# PBLD promotes IRF3 mediated the type I interferon (IFN-I) response and apoptosis to inhibit viral replication

**DOI:** 10.1038/s41419-024-07083-w

**Published:** 2024-10-03

**Authors:** Hongchao Zhu, Peili Hou, Fengyun Chu, Xingyu Li, Wenjia Zhang, Xiaonan Sun, Yu Liu, Guimin Zhao, Yuwei Gao, Daniel Chang He, Hongmei Wang, Hongbin He

**Affiliations:** 1 Ruminant Diseases Research Center, College of Life Sciences, Shandong Normal University, Jinan, Shandong China; 2 Department of Preventive Veterinary Medicine, College of Veterinary Medicine, Shandong Agricultural University, Taian, Shandong China; 3 Changchun Veterinary Research Institute, Chinese Academy of Agricultural Sciences, Changchun, Jilin China; 4 The College of Arts and Sciences, University of North Carolina at Chapel Hill, Chapel Hill, NC USA

**Keywords:** Cell death and immune response, Apoptosis, Drug delivery

## Abstract

Recent studies have implicated the phenazine biosynthesis-like domain-containing protein (PBLD) in the negative regulation of the development and progression of various cancers. However, its function in viral infection remains unknown. In this study, we found that PBLD plays important roles in multiple virus infections including BPIV3, SeV, VSV, and HSV-1. Our study revealed that PBLD enhances the expression of type I interferon (IFN-I) and ISGs through interferon regulatory factor 3 (IRF3). Further study indicated that PBLD promotes transcriptional phosphorylation of IRF3 (S385/386), thereby facilitating virus-induced IFN-I production. Interestingly, PBLD mediates virus-triggered mitochondrial apoptosis through its dependence on IRF3 (K313/315). Mechanistically, PBLD facilitated virus-induced apoptosis by recruiting the Puma protein to the mitochondria via IRF3. Additionally, we performed mutational analyses of IRF3, showing that its loss of either transcriptional or apoptotic function markedly increased viral replication. Moreover, macrophages with PBLD deficiency during viral infection exhibited decreased the IFN-I and ISGs expression, exacerbating viral infection. Importantly, mice deficient in PBLD exhibited increased viral replication and susceptibility to SeV infection, leading to decreased survival. Notably, Cedrelone, a chemical activator of PBLD, has the ability to reduce SeV replication. Collectively, we first discovered the new function of PBLD in viral infection, broadening our understanding of potential therapeutic targets and offering new insights for antiviral drug development.

## Introduction

Phenazine biosynthesis-like domain-containing protein (PBLD) also known as MAWBP, was initially isolated from a human liver cDNA library through the yeast two-hybrid system [1]. Recent studies have revealed a negative correlation between PBLD expression and the progression of various cancers, including gastric, hepatocellular, and breast cancer [2–4]. Currently, PBLD has been implicated in impeding disease progression through the NF-κB, Ras-Raf, and VEGF signaling pathways [5–7]. Despite its established role in cancer, the mechanism and function of PBLD in viral infections remain unknown.

The innate immune responses, which serve as the first line of defense against pathogen infection, have been well understood. The interaction between pathogen-associated molecular patterns (PAMPs) and pattern recognition receptors (PRRs) triggers multiple signaling pathways, leading to the production of pro-inflammatory cytokines, interferons, and IFN-stimulated genes (ISGs) to protect the host against pathogen infection [8]. Among those signaling pathways upon viral infection, type 1 interferon (IFN-I) signaling plays a central role in antiviral innate immune responses [9, 10]. Besides, IFN-I responses are tightly regulated to achieve protective immunity against microbial infection, involving a variety of intercellular pathogen and host-derived materials [11, 12]. Therefore, it is crucial to consider the roles of IFN regulators in viral replication and determine if they could serve as potential targets in combination with antivirus therapy.

Apoptosis is a programmed cell death process employed by multicellular organisms for maintaining cellular homeostasis and combating pathogens. There exists a number of subtypes of apoptosis, including the classic intrinsic mitochondrial apoptosis and extrinsic death-receptor superfamily signaling pathways [13, 14]. This process is triggered by intracellular stimuli, such as DNA damage, which modulate the expression of pro- and anti-apoptotic genes, ultimately leading to the release of cytochrome c and apoptosis-inducing factors from the mitochondria into the cytosol [15, 16]. Subsequently, activated caspase-9 and the effector caspases-3 and 7 play a major role in the execution of apoptosis [17, 18]. An increased number of evidence suggests that apoptosis plays a double-edged sword in virus replication. On one hand, apoptosis can limit viral infection by impairing cellular functions, thus serving as a protective mechanism [19–21]. Conversely, some viruses exploit apoptosis to enhance replication by facilitating cell killing and viral spread [22–24]. Notably, some viruses, including influenza A viruses (IAV), can manipulate host apoptosis at different stages of their lifecycle to their advantage [25]. Therefore, understanding these apoptotic pathways is important for the development of efficient antiviral therapeutics.

Both innate immunity and apoptosis play essential roles in maintaining host homeostasis. IRF3 is a well-documented cytoplasmic protein that is inactive in uninfected cells. Upon viral infection, IRF3 becomes activated by phosphorylation of its specific serine residues. This triggers its nuclear translocation, where it binds to the promoters of the target genes, thereby enhancing the production of interferon and interferon stimulating genes, contributing to antiviral innate immunity [26, 27]. Interestingly, IRF3 can also initiate an interferon-independent antiviral pathway, called RIG-I induced pathway of apoptosis (RIPA) [28]. To activate RIPA, IRF3 translocates from the cytoplasm to mitochondria [29, 30]. A non-destructive linear polyubiquitination on two lysine residues (193, 313/315) of human IRF3 is sufficient to confer RIPA activity [31]. Linear polyubiquitination of IRF3 is catalyzed by the linear ubiquitin chain assembly complex (LUBAC), enabling its interaction with pro-apoptotic proteins through their BH3 domain of C-terminus. The ubiquitinated IRF3/pro-apoptotic protein complex translocates to mitochondria, releasing cytochrome C and subsequently activating caspases, leading to apoptosis in infected cells [32, 33]. A growing number of RIPA-like pathways of IRF3 have been identified in various viral models including human T cell leukemia virus (HTLV1), vesicular stomatitis virus (VSV), and encephalomyelitis virus (EMCV), which protects the cells from productive viral replication [34, 35]. However, the specific pro-apoptotic protein involved in this process remains largely unclear.

In this study, we show that PBLD enhances IRF3-mediated innate immunity and apoptotic cell death, thereby leading to the suppression of viral replication in cell and mice models. Mechanistically, we uncover that PBLD upregulates IRF3 expression and promotes to activate specific serine residues (S385/S386) of IRF3, thus increasing antiviral immunity. Furthermore, we observe that PBLD facilitates IRF3-induced mitochondrial apoptosis by recruiting Puma to the mitochondria depending on IRF3 (K313/S315). In general, our findings unveil the novel role of PBLD in viral infection, thus providing a potential therapeutic target and contributing to our understanding of anti-viral strategies.

## Results

### PBLD positively regulates BPIV3 or SeV-induced type I IFN signaling pathway

**Fig. 1:**
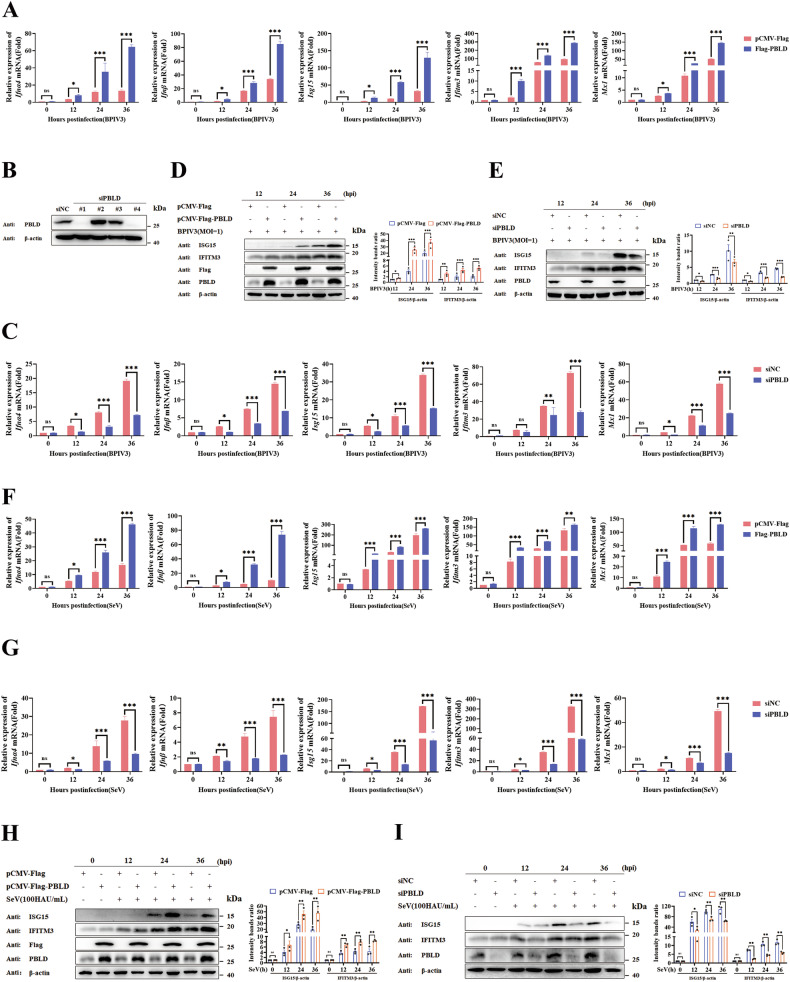
PBLD promotes the IFN-I signaling response induced by BPIV3 and SeV infection. **A** RT-qPCR analysis of *Ifnα4*, *Ifnβ*, and ISGs (*Isg15*, *Mx1*, and *Ifitm3*) in PBLD overexpressed HeLa cells and infected with BPIV3 (MOI = 1) for the indicated time. **B** Western blot analysis of the silencing effect of PBLD in HeLa cells treated with PBLD-specific siRNAs (siPBLD) or scrambled siRNA (siNC)for 36 h. **C** HeLa cells were transfected with PBLD-specific siRNA (siPBLD) or scrambled siRNA (siNC) for 36 h and then infected with BPIV3 (MOI = 1) for the indicated time. The mRNA levels of *Ifnα4*, *Ifnβ*, and ISGs (*Isg15*, *Mx1*, and *Ifitm3*) were analyzed by RT-qPCR. **D**, **E** Representative Western blot analysis of the ISGs (ISG15 and IFITM3) in HeLa cells with either overexpression or knockdown of PBLD followed by BPIV3 (MOI = 1) infection for the indicated time. **F**–**G** RT-qPCR analysis of *Ifnα4*, *Ifnβ*, and ISGs (*Isg15*, *Mx1*and *Ifitm3*) in HeLa cells with PBLD overexpression or knockdown in the content of SeV infection (100HAU/mL) for the indicated time. **H**, **I** Representative Western blot analysis of the ISGs (ISG15 and IFITM3) in HeLa cells with overexpression or knockdown of PBLD, infected with SeV infection (100HAU/mL) for the indicated hours. Data information: Data represent one of three independent experiments or collected from at least three. The gray intensity of the bands in (**D**, **E**, **H**, and **I**) from three independent experiments were analyzed using ImageJ software. Bars represent mean ± SEM of triplicate samples. Significance was determined by two-way ANOVA in (**A**, **C**, **F**, **G**, and **D**, **E**, **H**, and **I**) of gray intensity analysis of the bands. Ns, not significant; *P < 0.05; **P < 0.01; ***P < 0.001.

To investigate the potential role of PBLD in regulating the IFN-mediated antiviral response, we constructed a recombinant Flag-tagged PBLD eukaryotic expression vector. We then examined its ability to modulate the activation of the IFN-β and ISGs triggered by bovine parainfluenza virus 3 (BPIV3), a negative-strand RNA virus contributing to bovine respiratory disease complex (BRDC). Quantitative real-time PCR (RT-qPCR) analysis revealed that overexpression of PBLD significantly upregulated the transcription of *Ifnα4*, *Ifnβ*, and ISGs including *Isg15*, *Mx1*, and *Ifitm3* in BPIV3 infected HeLa cells (Fig. 1A) (SFig. 1A). We next investigated the contribution of PBLD to IFN-I response by siRNA-mediated knockdown in HeLa cells. Four pairs of PBLD siRNAs were designed and siPBLD No. 1, 4 were the best-performing, and siPBLD No. 1 was chosen for the subsequent experiments (Fig. 1B). After knockdown of endogenous PBLD, the results of RT-qPCR demonstrated that *Ifnα4, Ifnβ*, and ISGs transcription levels were reduced (Fig. 1C). Furthermore, western blot analysis revealed that PBLD overexpression increased the protein levels of ISG15 and IFITM3 (Fig. 1D), whereas PBLD knockdown reduced ISG15 and IFITM3 protein expression (Fig. 1E). Similar results were observed in Sendai virus (SeV) -infected HeLa cells upon PBLD overexpression (Fig. 1F) or knockdown (Fig. 1G). Moreover, western blot analysis showed that ISG15 and IFITM3 were markedly increased in SeV-infected cells overexpressing Flag-PBLD (Fig. 1H) but decreased after PBLD knockdown (Fig. 1I). Altogether, these data suggest that PBLD acts as a positive regulator of the IFN-I response during BPIV3 and SeV infections.

### PBLD facilitates the virus-induced type I IFN signaling pathway via IRF3

To delineate the mechanisms behind the above-mentioned effects during viral infection, we performed transcriptome analysis for the differentially expressed genes regulated by PBLD using gene microarray upon BPIV3 infection. The results provide clues for the following study of PBLD involved in IFN-related pathways, especially with upregulated IRF3 expression (SFig. 1B, C). Subsequent RT-qPCR and western blot assay confirmed that PBLD overexpression could increase the mRNA and protein levels of IRF3 during BPIV3 infection (SFig. 1D, E). Similar results were observed when HeLa cells were infected with model RNA viruses (SeV) (SFig. 1F, G) or DNA virus (Herpes simplex virus type 1, HSV-1) (SFig. 1H, I). Conversely, knockdown of PBLD in BPIV3, SeV or HSV-1 infected HeLa cells had the opposite effect (SFig. 1J–O). Thus, we speculated that PBLD promotes the virus-induced type I IFN signaling pathway by modulating IRF3.

**Fig. 2:**
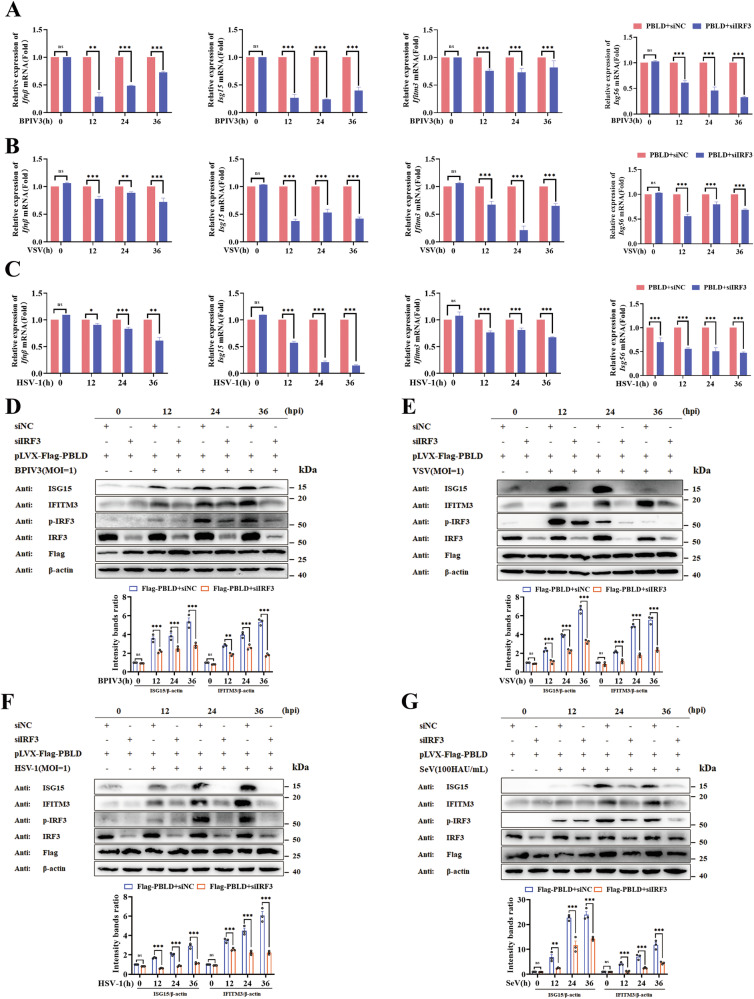
PBLD promotes the virus-induced IFN-I signaling pathway via IRF3. **A**–**C** HeLa cells with stable expression of PBLD were transfected with IRF3-specific siRNA (siIRF3) or siNC, and then infected with BPIV3, VSV and HSV-1 at an MOI of 1 for 0, 12, 24 and 36 h, respectively. The mRNA levels of *Ifnβ* and ISGs (*Isg15*, *Ifitm3* and *Isg56*) were then analyzed by RT-qPCR. **D**–**G** Representative Western blot analysis of the ISGs (ISG15 and IFITM3) in PBLD stably overexpressed HeLa cells with knockdown of IRF3 in the content of BPIV3, VSV and HSV-1 infection (MOI = 1) for 0, 12, 24 and 36 h, respectively. The gray intensity of the bands from three independent experiments were analyzed using ImageJ software. Data from three independent experiments and error bars are presented as the mean ± SEM. Significance was determined by two-way ANOVA. Ns not significant; *P < 0.05, **P < 0.01, ***P < 0.001.

Since IRF3 becomes activated upon viral infection and then translocates to the nucleus to induce antiviral innate immunity [36]. Accordingly, IRF3 knockdown inhibits the transcripts production of interferons *(Ifnβ)* and ISGs (*Isg15, Ifitm3* and *Isg56)* in the absence of PBLD upon BPIV3, (Vesicular stomatitis virus, VSV), SeV and HSV-1 infection (SFig. 2A–D). Western blot analysis showed that ISG15 and IFITM3 were markedly reduced in BPIV3, VSV, SeV and HSV-1 infected cells when IRF3 was knocked down (SFig. 2E–H). To further confirm the effect of IRF3 on PBLD-mediated antiviral immune responses, the HeLa cell lines that stably express PBLD were constructed (SFig. 3A). Then the IFN-I response promoted by PBLD after knockdown of IRF3 using siRNA during virus infection was assessed. As shown in Fig. 2A, IRF3 knockout markedly decreased the production of *Ifnβ, Isg15, Ifitm3* and *Isg56* genes promoted by PBLD in response to BPIV3 infection (SFig. 3B). This effect was also observed during VSV or HSV-1 infection (Fig. 2B, C and SFig. 3C, D). Consistently, the overexpression of PBLD but knockdown of IRF3 resulted in a decrease in ISG15 and IFITM3 proteins upon viral stimulation with BPIV3, VSV, HSV-1, and SeV (Fig. 2D–G). Collectively, these findings demonstrate that PBLD promotes the virus-induced type I IFN signaling pathway by upregulating the expression of IRF3.

### PBLD enhances the virus-induced type I IFN signaling pathway through exacerbating phosphorylation modification at the IRF3 (S385/386) site

**Fig. 3:**
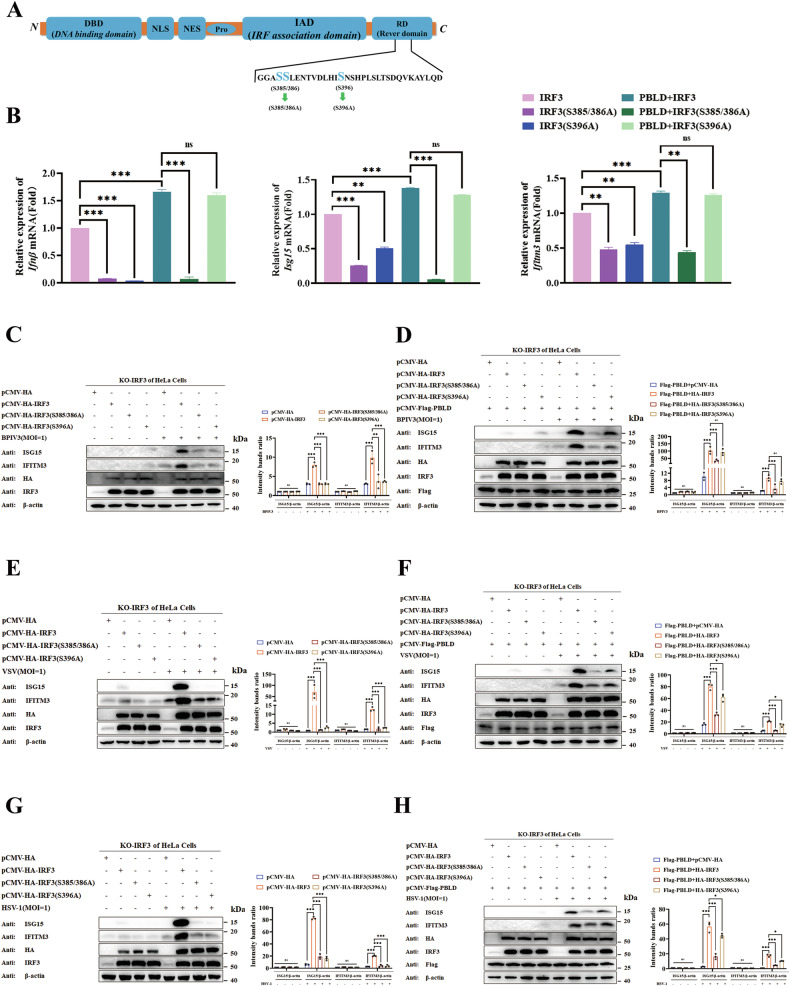
PBLD enhances virus-induced IFN-I signaling pathway through promoting phosphorylation modification at the IRF3 (S385/386) site. **A** Mapping the IRF3 phosphorylation sites. **B** Flag-PBLD construct, or vector were transfected together with wild-type IRF3 (HA-IRF3) and IRF3 mutants: HA-IRF3(S385/386A), HA-IRF3(396A) into IRF3 knockout HeLa cell lines for 24 h. Following BPIV3 infection for another 24 h, the mRNA of *Ifnβ* and ISGs (*Isg15* and *Ifitm3*) were determined by RT-qPCR analysis. Data from three independent experiments and error bars are presented as the mean ± SEM. **C**–**H** Representative Western blot analysis of the ISGs (ISG15 and IFITM3) in IRF3 knockout HeLa cell lines with wild-type IRF3 or IRF3 mutants in the absenence or presence of PBLD upon BPIV3 (MOI = 1), VSV (MOI = 1) and HSV-1 (MOI = 1) infection. The gray intensity of the bands from three independent experiments was analyzed using ImageJ software. Significance was determined by one-way ANOVA in (**B**–**H**). Ns, not significant, *P < 0.05, **P < 0.01, ***P < 0.001.

Phosphorylation of the carboxy-terminal serine clusters, such as IRF3 S385/S386 and IRF3 S396 are essential for the dimerization and activation of IRF3 [37]. To map the essential phosphorylation sites of IRF3 for downstream signaling, the serine residues of IRF3 S385/S386 and IRF3 S396, were mutated to alanine to create the mutants HA-IRF3 (S385/386A) and HA-IRF3 (S396A) (Fig. 3A). Specifically, to exclude the influence of endogenous IRF3, we constructed the IRF3 knockout HeLa cell lines (SFig. 3E). Subsequently, the site of IRF3 phosphorylation by which PBLD promoted the virus-induced type I IFN signaling pathway was identified by analyzing IRF3 mutations in the IRF3 knockout cell lines. The results of RT-qPCR demonstrated that, unlike wild-type IRF3, both individual mutations impaired *Ifnβ* and ISGs induction in the absence of PBLD during BPIV3 infection. Interestingly, in the presence of PBLD, IRF3 (S385/386A) abolished the promotion of *Ifnβ* as well as *Isg15* and *Ifitm3* expression. However, the mutation of Ser-396 in IRF3 moderately affected the PBLD-induced *Ifnβ*, *Isg15* and *Ifitm3* expression (Fig. 3B). Similarly, the western blot analysis revealed that both mutations of IRF3 strongly diminished ISG15 and IFITM3 protein expression during BPIV3 infection (Fig. 3C), indicating that these residues function cooperatively in the activation of IRF3 and the induction of innate immunity. Consistent with the results mentioned above, the mutation of Ser-385 and Ser-386 in IRF3 (S385/S386A) abolished the promotion of ISG15 and IFITM3 expression by PBLD, but the mutation of Ser-396 in IRF3 did not affect PBLD-induced activation of ISG15 and IFITM3 (Fig. 3D). Similar results were obtained in the context of VSV or HSV-1 infection (Fig. 3E–H), suggesting that PBLD promotes transcriptional activity of IRF3 by phosphorylating its Ser-385 and Ser-386 residues in virus-infected cells, thereby leading to nuclear translocation and the IFN-I response. Taken together, these results demonstrate that PBLD promotes the virus-induced IFN-I signaling pathway through promoting phosphorylation of IRF3 at the S385/386 site.

### PBLD promotes virus-induced apoptosis

**Fig. 4:**
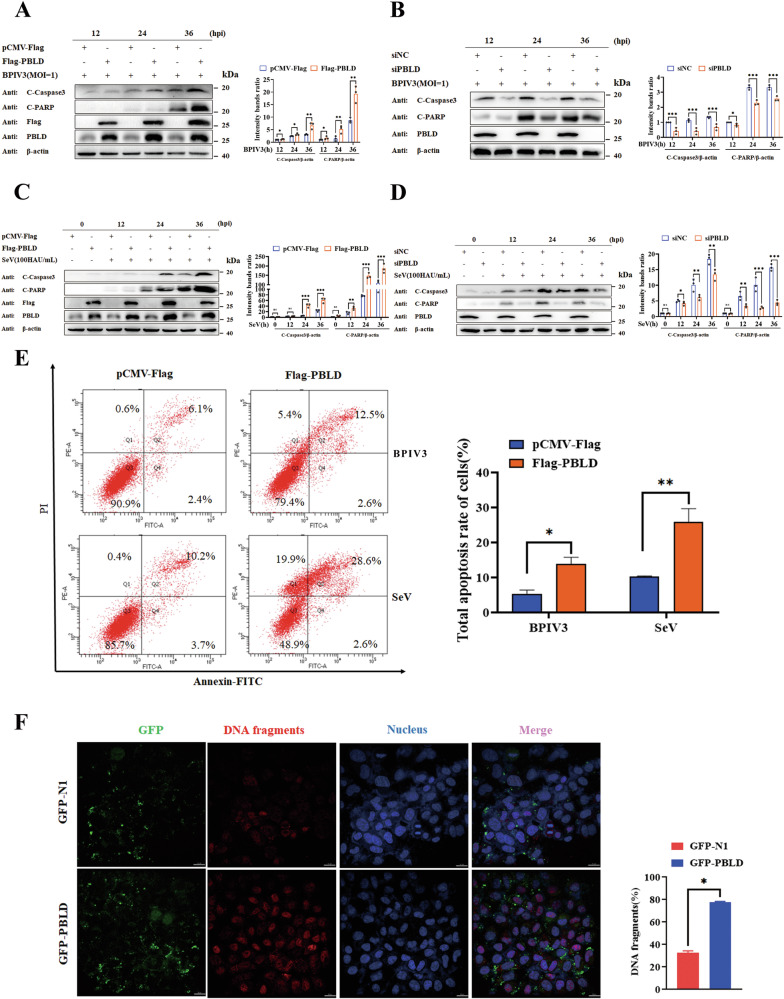
PBLD promotes virus-induced apoptosis. **A**, **B** HeLa cells were transfected with Flag-PBLD, pCMV-Flag, siPBLD, or scrambled siRNA (siNC) for 24 h, and then the cells were infected with BPIV3 (MOI = 1) for the indicated time. The expression of apoptosis-related proteins was determined by western blot analysis. The gray intensity of the bands from three independent experiments was analyzed using ImageJ software. **C**, **D** Representative Western blot analysis of the expression of apoptosis-related proteins in PBLD overexpressed or PBLD knockdown HeLa cells and infected with SeV (100HAU/mL) for the indicated time. The gray intensity of the bands from three independent experiments was analyzed using ImageJ software. **E** Flow cytometry analysis of PBLD-induced apoptosis in HeLa cells during BPIV3 or SeV infection. HeLa cells were transfected with the pCMV-Flag control or Flag-PBLD plasmid, and then infected with BPIV3 (MOI = 1) or SeV (100HAU/mL). After 36 h, the cells were collected, stained, and analyzed by flow cytometry. The total apoptosis rate of the cells was statistically analyzed. Images are representative of ≥3 experiments. **F** The evaluation of PBLD-induced cell apoptosis was observed by the TUNEL assay. HeLa cells were transfected with the pEGFP-N1 control vector or GFP-PBLD plamids. At 24 hours post-transfection (hpt), the cells were infected with BPIV3 for 24 h. Subsequently, the cells were fixed and stained with a TUNEL reaction mixture (red), and DAPI was used to stain the nuclei (blue). The stained cells were then visualized by fluorescence microscopy. Images are representative of ≥3 experiments. Scale bars, 10 μm. Data information: The results represent the means ± SEM from three independent experiments. Significance was determined by two-way ANOVA in (**A**, **B**, **C**, **D**, **E**) and Student’s t-test in (**F**). Ns not significant, *P < 0.05, **P < 0.01, ***P < 0.001.

Previous studies have established a connection between PBLD and cancer cell apoptosis [38]. However, the relationship between PBLD and apoptosis in viral infections remains unexplored. We next examined the apoptosis associated protein expression in BPIV3-infected HeLa cells overexpressing PBLD. Consistently, as shown in Fig. 4A, the levels of cleaved caspase-3 and cleaved PARP were significantly elevated after 24 h postinfection(hpi) of BPIV3 in PBLD overexpressing cells. In contrast, knockdown of PBLD significantly decreased cleaved caspase-3 and cleaved PARP expression (Fig. 4B), suggesting that PBLD can activate caspase-3 and PARP, thereby inducing apoptosis. Similar results were observed in SeV-infected HeLa cells upon PBLD overexpression (Fig. 4C) or knockdown (Fig. 4D). Furthermore, the effects of PBLD on the apoptosis in BPIV3 or SeV infected cells were detected by flow cytometry. The results showed that overexpression of PBLD significantly increased the apoptosis-positive cells compared to the vector control groups (Fig. 4E). Additionally, TUNEL staining, which is widely utilized as a readout for apoptosis, revealed that PBLD overexpression led to a higher intensity of staining and a larger population of apoptotic cells compared to the vector control cells at 24 hpi of BPIV3 (Fig. 4F). All in all, these findings strongly suggest that PBLD facilitates virus-induced apoptosis.

### PBLD promotes virus-induced intrinsic mitochondrial apoptosis signaling pathways via IRF3

**Fig. 5:**
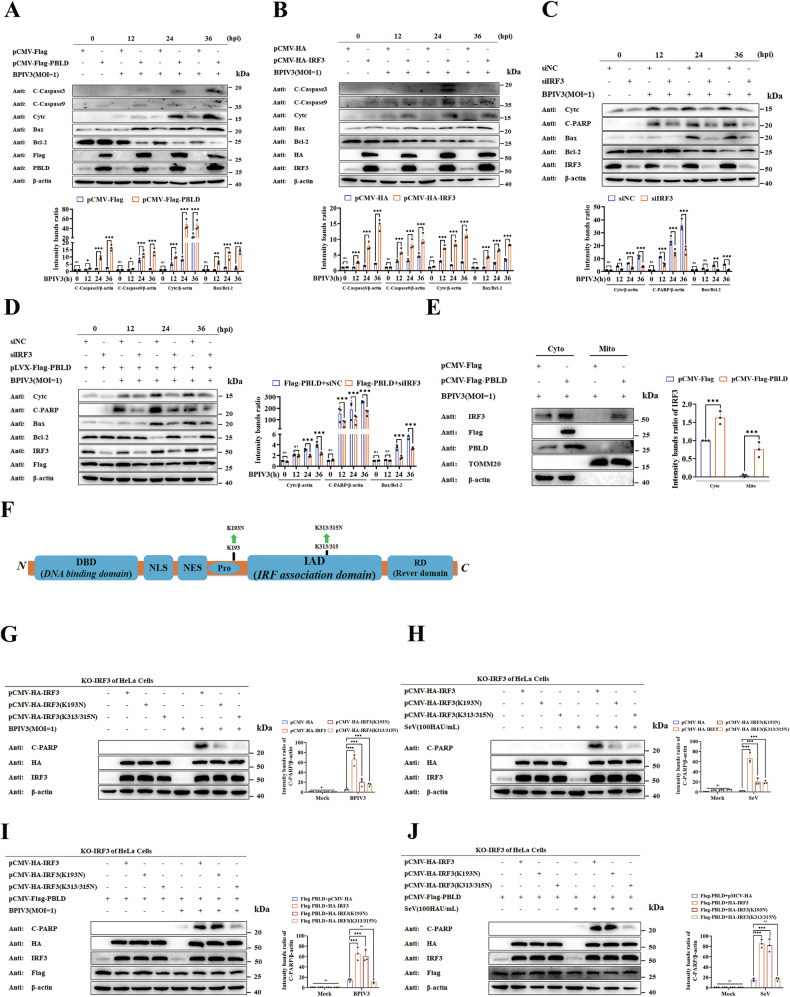
PBLD promotes virus-induced intrinsic mitochondrial apoptosis pathway via IRF3. **A** HeLa cells were transfected with Flag-PBLD or pCMV-Flag for 24 h, and then the cells were infected with BPIV3 (MOI = 1) for the indicated time. The expression of intrinsic mitochondrial apoptosis proteins was determined by western blot analysis. **B**, **C** HeLa cells were transfected with pCMV-Flag, pCMV-HA-IRF3, siIRF3, or siNC for 24 h, and then the cells were infected with BPIV3 (MOI = 1) for the indicated time. The expression of intrinsic mitochondrial apoptosis proteins was determined by western blot analysis. **D** Flag-PBLD HeLa cell lines were treated with siIRF3 or scrambled siRNA (siNC) for 24 h, and then infected with BPIV3 (MOI = 1) for the indicated time. The expression of Bax, Bcl_2_, Cytc, and cleaved PARP proteins were analyzed by western blotting. **E** Mitochondrial isolation and sub-cellular localization assay for IRF3 affected by PBLD during BPIV3 infection. The cells were transfected with Flag-PBLD or vector for 24 h. Following BPIV3 (MOI = 1) infection for 24 h, cytosolic and mitochondrial proteins were prepared, and the expression of IRF3 was determined by western blot analysis. β-actin and TOMM20 were used for loading control of cytosolic and mitochondrial protein, respectively. **F** Mapping the ubiquitination sites of IRF3. **G**–**J** pCMV-Flag or pCMV-Flag-PBLD in combination of pCMV-HA-IRF3, pCMV-HA-IRF3(S385/386 A) and pCMV-HA-IRF3(S396A) plasmids were transfected into IRF3 knockout HeLa cell lines and then infected with BPIV3 (MOI = 1) or SeV (100HAU/mL) for 24 h, respectively. The expression of cleaved PARP apoptosis protein was verified by western blotting. The gray intensity of the bands in (**A**–**E** and **G**–**J**) from three independent experiments were analyzed using ImageJ software. Significance was determined by two-way ANOVA in (**A**–**E**), one-way ANOVA in (**G**–**J**). Ns not significant, *p < 0.05, **p < 0.01, ***p < 0.001.

We further investigated the type of cell apoptosis induced by PBLD, and the results showed that both the extrinsic apoptosis-related protein (Cleaved caspase-8) (SFig. 4A), and the intrinsic mitochondrial apoptosis-related protein, cytochrome C (cyto c), cleaved caspase-9 and caspase-3 were markedly activated in PBLD-expressing HeLa cells compared to the control group (Fig. 5A). Moreover, the decreased Bcl-2/Bax index, which could lead to the loss of mitochondrial membrane potential (MMP) and improving the permeability of the mitochondrial membranes [39], was also observed (Fig. 5A), suggesting that PBLD promote BPIV3-induced the extrinsic apoptosis and the intrinsic mitochondrial apoptosis. Although it has been reported that IRF3 stimulates cell apoptosis independently of its DNA-binding domain [28], the functional implication in viral infection remains largely unclear. We first analyzed the role of IRF3 in cell apoptosis during BPIV3 infection using HeLa cells expressing HA-IRF3. The results of western blotting showed that Bax, cyto C, cleaved caspase-9 and caspase-3 were increased, whereas Bcl-2 was decreased (Fig. 5B). In contrast, the siRNA-mediated silence of IRF3 exhibited a comparatively reduced level of Bax, cyto C, cleaved PARP, promoted Bcl2 expression following BPIV3 infection (Fig. 5C). However, the cleaved caspase-8 was not detected in either IRF3 overexpression or knockdown cells (SFig. 4B, C), confirming that IRF3 contributes to intrinsic mitochondrial apoptosis in response to BPIV3 infection. To further confirm whether PBLD induced mitochondria apoptosis via IRF3 protein, cell apoptosis was detected in PBLD-expressing cells after knockdown of IRF3. As expected, western blot analysis indicated that the knockdown of IRF3 attenuated PBLD-induced apoptosis, as evidenced by weakened Bax, cleaved PARP formation and cyto C, but promoted Bcl2 expression compared with that of vector control (Fig. 5D), suggesting that PBLD facilitates BPIV3-induced endogenous mitochondrial apoptosis signaling pathways through IRF3.

Since PBLD induces intrinsic mitochondrial apoptosis via IRF3, one possibility is that PBLD escorts IRF3 to the mitochondria. To confirm this hypothesis, mitochondrial proteins from BPIV3-infected cells were purified, and the mitochondrial IRF3 was tested in PBLD overexpression or control cells. As shown in Fig. 5E, overexpression of PBLD promoted the translocation of IRF3 to the mitochondria. IRF3 is known to induce apoptosis through its key ubiquitylation sites (K193, K313, K315) [31]. To further characterize the role of IRF3, a series of ubiquitylation site mutant versions of IRF3 were generated (Fig. 5F). Then the IRF3 knockout (IRF3^−/−^) HeLa cell lines were transiently transfected with IRF3 mutants or wild-type IRF3. PBLD-mediated apoptosis was measured by immunoblotting. The results showed that BPIV3-induced apoptosis occurred with wild-type IRF3, but the IRF3 mutations that contained (K193N, K313N, K315N) indicated partially compromised apoptosis in the absence of PBLD (Fig. 5G). Similar results were observed in the process of SeV infection (Fig. 5H). Notably, in the presence of PBLD, wild-type IRF3, and IRF3 K193N could induce apoptosis, whereas the IRF3 proteins containing the K313N/K315N mutations partially enhanced PBLD-induced apoptosis in BPIV3 or SeV -infected cells (Fig. 5I, J), suggesting K313/K315 as potential ubiquitylation sites of IRF3, implicates PBLD-induced apoptosis as a consequence of viral infection.

### PBLD facilitates virus-induced apoptosis through an IRF3 recruited, Puma-dependent mitochondrial pathway

**Fig. 6:**
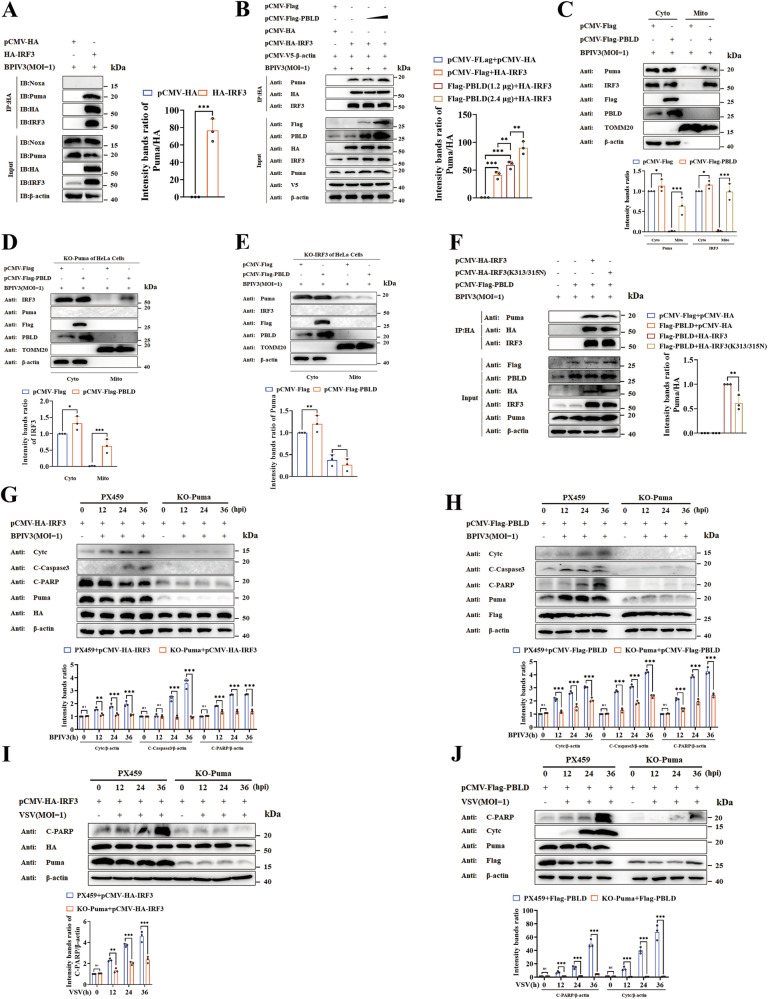
PBLD promotes virus-induced apoptosis through an IRF3-recruited PUMA-dependent mitochondrial pathway. **A** Interaction between IRF3 and Puma was analyzed by co-immunoprecipitation. HeLa cells expressing HA-IRF3 were exposed to BPIV3 infection for 24 h, and then immunoprecipitation of IRF3 was performed, and the presence of Puma and Noxa proteins was assessed by western blot analysis. **B** The effect of PBLD on the binding between IRF3 and Puma was examined by co-immunoprecipitation analysis. HeLa cells were cotransfected with HA-IRF3 and different doses (1.2 μg, 2.4 μg) of PBLD for 24 h, and then infected with BPIV3 for another 24 h. After that, the cell lysates were immunoprecipitated with HA antibody, and the presence of Puma was detected by western blot analysis. **C** PBLD facilitates the interaction between IRF3 and Puma within the mitochondria as demonstrated by western blot analysis. HeLa cells were transfected with Flag-PBLD and infected with BPIV3 for 24 h. The mitochondrial and cytosolic fractions were isolated and analyzed by western blot for the indicated proteins. β-actin and TOMM20 function as the loading control for cytosolic proteins and mitochondrial proteins, respectively. **D** PBLD promoted the localization of IRF3 at the mitochondria and was examined in Puma knockout cell lines. **E** The effect of PBLD on the mitochondrial localization of Puma was detected in IRF3 knockout cell lines. **F** Wild-type IRF3 (HA-IRF3) and IRF3 mutants: HA-IRF3(K313/315 N) interacted with Puma was analyzed by co-immunoprecipitation in the context of PBLD. **G**–**J** Knockout of Puma attenuated IRF3- or PBLD-induced apoptosis during BPIV3 or VSV infection. Puma knockout cell lines were transfected with HA-IRF3 or Flag-PBLD, and infected with BPIV3 at a MOI of 1 for the indicated time. Then, apoptosis-associated proteins in these cells were determined by western blotting. The gray intensity of the bands in (**A**–**J**) from three independent experiments was analyzed using ImageJ software. Significance was determined by Student’s *t*-test in (**A**), one-way ANOVA in (**B**), two-way ANOVA in (**C**–**J**). Ns, not significant, *p < 0.05, **p < 0.01, ***p < 0.001.

To investigate the role of IRF3 in PBLD function, we employed an immunoprecipitation assay to determine whether IRF3 interacts with proapoptotic proteins. Interestingly, we found IRF3 interacted with p53 upregulated modulator of apoptosis (Puma), but not Noxa (Fig. 6A). Furthermore, we observed that PBLD enhanced IRF3’s ability to interact with Puma in a dose-dependent way (Fig. 6B). To validate the physiological significance of this interaction, mitochondrial proteins were isolated, and a higher level of IRF3 and Puma were detected at mitochondria in PBLD-expressed cells (Fig. 6C). Additionally, to confirm whether the localization of Puma to mitochondria is dependent on IRF3, and vice versa. HeLa cell lines were developed to knock out the expression of Puma (Puma^−/−^) (SFig. 4D). As shown in Fig. 6D, E, PBLD-induced mitochondrial localization of IRF3 in Puma KO cell lines but PBLD had no effect on mitochondrial localization of Puma in IRF3 KO cells. Moreover, the interaction between IRF3 (K313/315N) and Puma was reduced in PBLD-expressed cells (Fig. 6F), indicating that PBLD-induced mitochondria localization of Puma is dependent on IRF3.

Furthermore, to identify IRF3-mediated apoptosis is Puma-dependent, Puma^−/−^ cell lines were transfected with IRF3 or PBLD, and western blotting analysis was conducted to assess their ability to induce apoptosis during BPIV3 infection. As displayed in Fig. 6G and H, the knockout of Puma resulted in a significant decrease in apoptosis levels upon expression of IRF3 or PBLD compared to control cells, raising a possibility that PBLD or IRF3-mediated apoptosis is dependent on Puma. Consistently, PBLD or IRF3-mediated apoptosis was reduced after Puma was knockout during VSV infection (Fig. 6I, J). Altogether, the above results confirmed that PBLD plays an important role in facilitating virus-induced apoptosis in a Puma-dependent manner that involves the recruitment of IRF3.

### PBLD promotes IRF3-mediated the IFN-I response and apoptosis against virus replication

**Fig. 7:**
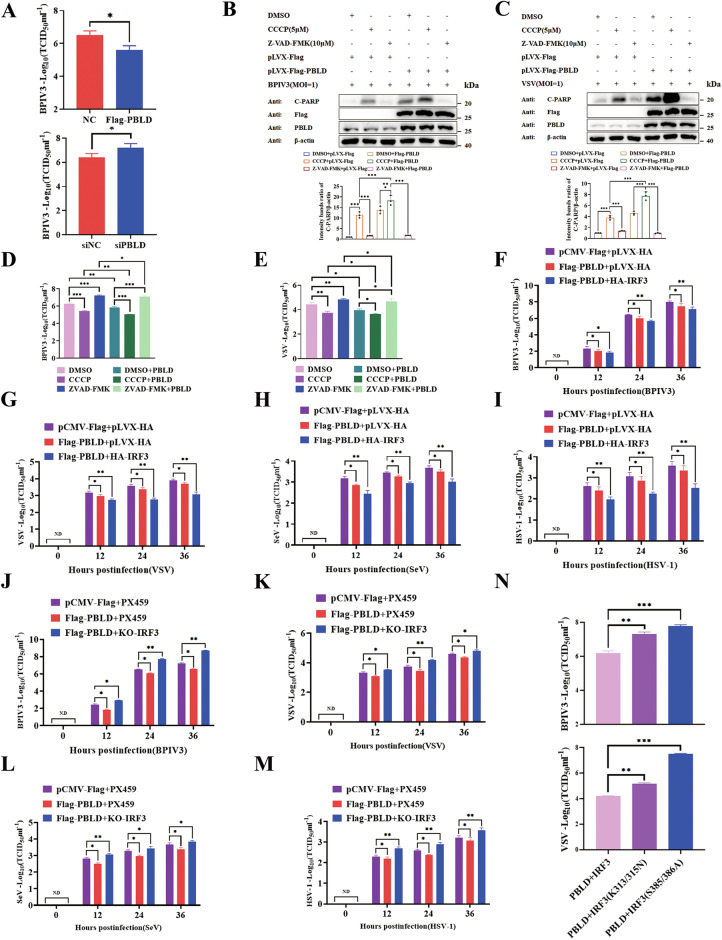
PBLD promotes IRF3-mediated the IFN-I response and apoptosis against virus replication. **A** The inhibitory effect of PBLD on BPIV3 replication was determined by TCID_50_ assay. HeLa cells were transfected with pCMV-Flag, Flag-PBLD, siNC or siPBLD for 24 h, respectively, and then infected with BPIV3. After 24 h, the viral titers were determined by TCID_50_ assay. **B**, **C** Western blotting analysis of the apoptosis proteins in BPIV3 or VSV-infected HeLa cells treated with DMSO, CCCP (5 μM) or Z-VAD-FMK (10 μM) with and without the presence of PBLD protein at 24 hpi. **D**, **E** The viral titer of BPIV3 or VSV was determined by TCID_50_ assay in HeLa cells transfected with Flag-PBLD or vector control and treated with DMSO, CCCP (5 μM) or Z-VAD-FMK (10 μM) at 24 hpi. **F**–**I** The viral titer of BPIV3, VSV, SeV, or HSV-1 was determined by TCID_50_ assay in Flag-PBLD and HA-IRF3 co-expressed HeLa cell at 24 hpi. **J**–**M** The viral titer of BPIV3, VSV, SeV, or HSV-1 was determined by TCID_50_ assay in Flag-PBLD expressed HeLa cell after IRF3 knockout at 24 hpi. **N** The viral titer of BPIV3 was determined by TCID_50_ assay in IRF3 knockout cells with co-expressed Flag-PBLD and WT-IRF3 as well as IRF3 mutants. Data are the mean ± SEM from three independent experiments. The gray intensity of the bands in (**B** and **C**) from three independent experiments were analyzed using ImageJ software. Significance was determined by Student’s *t*-test in (**A**), one-way ANOVA in (**B**–**N**). Ns, not significant, *P < 0.05, **P < 0.01, ***P < 0.001.

Since PBLD promotes IRF3-mediated the IFN-I response and apoptosis, we proceeded to investigate the effect of PBLD or IRF3 on viral replication. The overexpression of PBLD or IRF3 effectively impaired BPIV3 replication as indicated by decreased virus titer of tissue culture infectious dose 50 (TCID_50_), viral HN mRNA, and protein, while knockdown of PBLD had the opposite effect (Fig. 7A, SFig. 5A–H), suggesting that PBLD or IRF3 inhibits BPIV3 infection.

Given that RIG-I-like receptor-induced IRF3-mediated pathway of apoptosis (RIPA) contributes to its antiviral function [28], we hypothesized that PBLD-promoted apoptosis through IRF3 would inhibit viral replication. To investigate this, we treated cells with the apoptosis inducer CCCP or the inhibitor Z-VAD-FMK following BPIV3 or VSV infection, in the presence or absence of PBLD. The effect of CCCP and Z-VAD-FMK on apoptosis was validated by immunoblot analyses (Fig. 7B, C). The results demonstrated that CCCP treatment reduced the titer of BPIV3, whereas Z-VAD-FMK treatment increased the titer of BPIV3. Importantly, CCCP treatment augmented the inhibitory effect of PBLD on the titer of BPIV3, while Z-VAD-FMK treatment had the opposite effect on the titer of BPIV3 (Fig. 7D). Similar results were obtained in PBLD-expressing cells treated with CCCP or Z-VAD-FMK after VSV infection (Fig. 7E). Furthermore, when PBLD or IRF3 was overexpressed, knockout of Puma promoted BPIV3 and VSV replication as demonstrated by increased BPIV3 HN and VSV G mRNA and protein expression as well as raised virus titer of BPIV3 and VSV compared to control group, respectively (SFig. 6A-L), suggesting that knockout of Puma reduces PBLD or IRF3 triggered apoptosis and leads to an increase in viral replication after BPIV3 and VSV infection.

To further determine whether the antiviral activity of PBLD is dependent on IRF3, we transfected PBLD into IRF3-overexpressed or IRF3-knockout cells upon virus infection. The results of virus titer showed that the co-expression of IRF3 and PBLD significantly attenuated BPIV3, VSV, SeV or HSV-1 replication compared with that in individual expression of PBLD alone (Fig. 7F–I). Conversely, the opposite effect of PBLD on virus titers was observed after IRF3 was knockout during BPIV3, VSV, SeV or HSV-1 infection (Fig. 7J–M). Consistently, the expression of BPIV3 HN and SeV NP mRNA and protein was increased compared to the control group (SFig. 7A–D). These data demonstrate that IRF3 play an important role in PBLD-suppressed virus replication.

Additionally, our findings reveal the crucial role of both the IFN-I response and apoptotic activities of IRF3 in its overall antiviral function. To assess the relative importance of these two antiviral branches in the terms of their contributions to viral replication, we used two distinct IRF3 mutants: IRF3-S385/S386A, which is functional only in the apoptotic activities, and IRF3-K313/K315N, which exclusively exhibits transcriptional activity of IFN-I response. Upon expression of IRF3 mutants in PBLD expressing cells, our results demonstrate that PBLD showed a strong promotion of BPIV3 or VSV replication compared with that of the wild-type IRF3 (Fig. 7N). Collectively, PBLD facilitates IRF3-mediated the IFN-I response and apoptosis as defense mechanisms against viral replication.

### PBLD enhances the virus-induced IFN-I response in macrophages

**Fig. 8:**
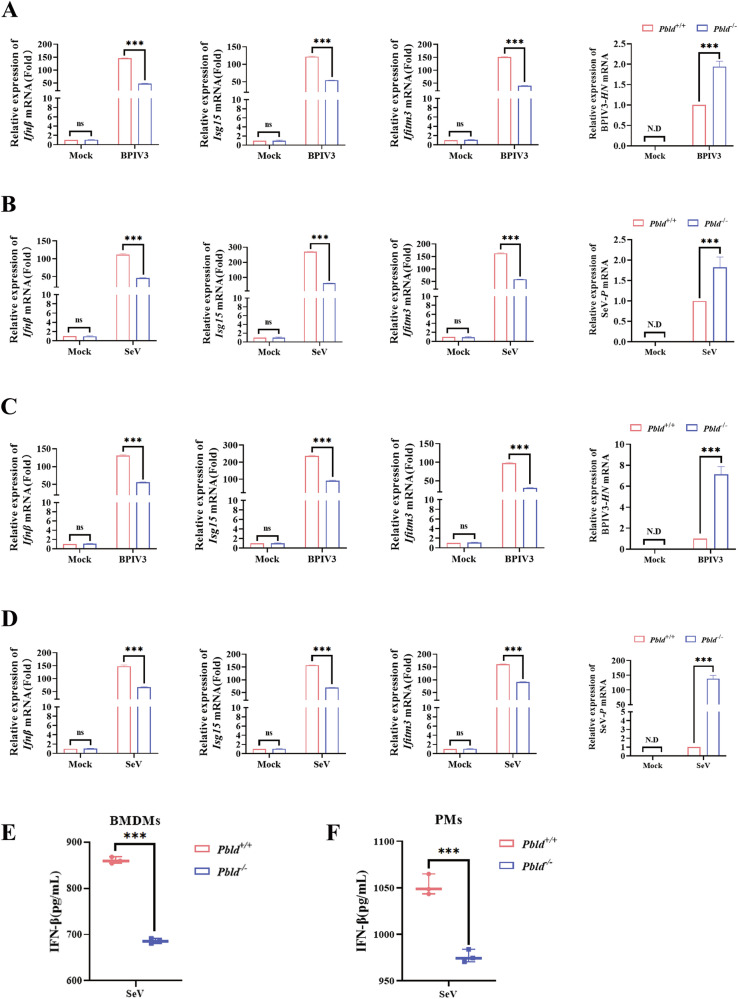
PBLD promotes virus-induced IFN-I response in macrophages. **A**, **B** BMDMs from *Pbld*^+/+^ and *Pbld*^−/−^ mice were infected with BPIV3 or SeV. The mRNA expression of *Ifnβ* and the ISGs (*Isg15* and *Ifitm3*) as well as BPIV3 *HN* or SeV-*P* were measured by RT-qPCR analysis. **C**, **D** PMs from *Pbld*^+/+^ and *Pbld*^−/−^ mice were infected with BPIV3 or SeV. The mRNA expression of *Ifnβ* and the ISGs (*Isg15* and *Ifitm3*) as well as BPIV3 *HN* or SeV-*P* were measured by RT-qPCR analysis. **E**, **F** The ELISA assay of IFN-β production in *Pbld*^+/+^ and *Pbld*^−/−^ BMDCs differentiated with GM-CSF (20 ng/mL), and PMs infected with SeV for 24 h. Data are the mean ± SEM from three independent experiments. Significance was determined by two-way ANOVA in (**A**–**D**), Student’s *t*-test in (**E** and **F**), ns, not significant, ***P < 0.001.

To further confirm the effect of PBLD deficiency on antiviral immune responses, we prepared *Pbld* gene knockout mice, the absence of PBLD expression in tissues, including liver, spleen, and lung was determined by western blotting (SFig. 8A–D). Subsequently, the production of IFN-I and ISGs in bone marrow-derived macrophages (BMDMs) and peritoneal macrophages (PMs) obtained from *Pbld* deficiency (*Pbld*^−/−^) mice following BPIV3 or SeV infection was investigated. As expected, *Pbld* knockout markedly decreased the production of the *Ifnβ*, *Isg15* and *Ifitm3* genes in both BMDMs (Fig. 8A, B) and PMs (Fig. 8C, D) in response to BPIV3 or SeV infection. Moreover, *Pbld* deficiency markedly reduced secretion of IFN-β protein in SeV-infected BMDMs (Fig. 8E) and PMs (Fig. 8F). Furthermore, the effect of *Pbld* deficiency on viral infection was accompanied by an increase of BPIV3 or SeV viral gene (Fig. 8A–D). Taken together, these data demonstrate that PBLD potentiates innate immune responses against virus infection in murine primary macrophages.

### *Pbld* deficiency mice are more susceptible to viral infection

**Fig. 9:**
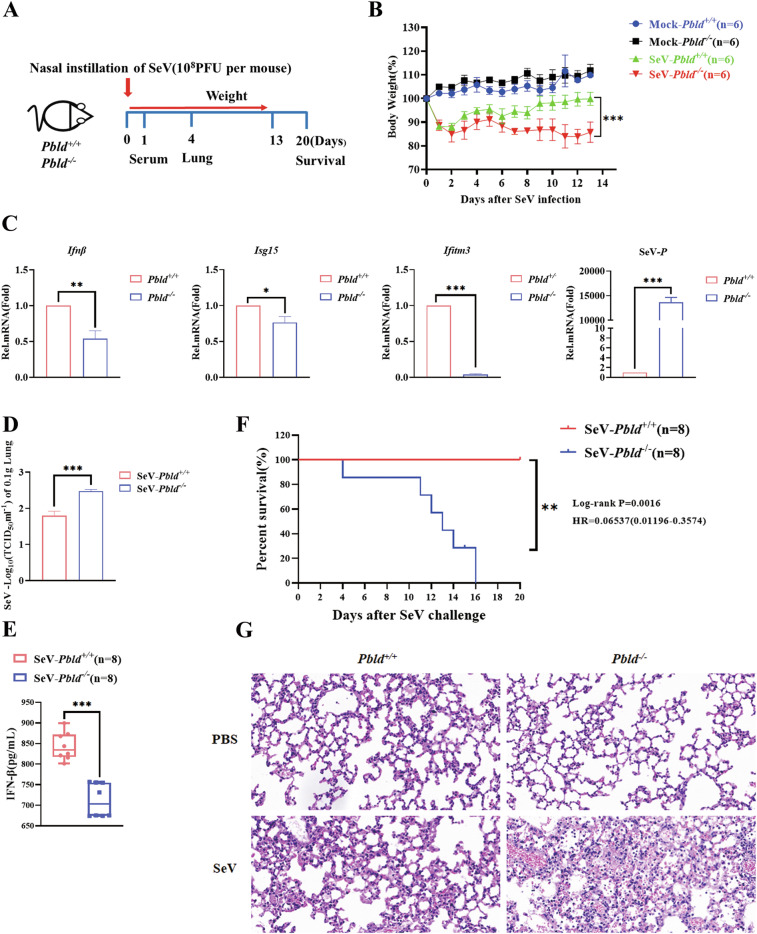
Mice with *Pbld* deficiency are more susceptible to virus infection. **A** A working model was proposed to illustrate the experimental workflow, where *Pbld*^+/+^ and *Pbld*^−/−^ mice were intravenously inoculated with SeV (10^8^PFU per mouse). **B** The body weights of *Pbld*^+/+^ and *Pbld*^−/−^ mice (*n* = 6 mice per group), inoculated intravenously with SeV (10^8^PFU per mouse) or mock infection, were recorded. **C** *Pbld*^+/+^ and *Pbld*^−/−^ mice (*n* = 6 mice per group) were intravenously inoculated with SeV (10^8^PFU per mouse). RT-qPCR analysis of the mRNA expression of *Ifnβ* and ISGs (*Isg15* and *Ifitm3*) and the viral load of *P* gene in the lung at 4 dpi of SeV infection. **D** Virus titer of lung was determined by TCID_50_ assay, and the samples were retrieved from *Pbld*^+/+^ and *Pbld*^−/−^ mice (*n* = 6 mice per group) that were inoculated intravenously with SeV at 4 dpi. **E** ELISA assay of IFN-β in serum of control *Pbld*^+/+^ and *Pbld*^−/−^ mice intraperitoneally infected with SeV (10^8^PFU per mouse) for 24 h (*n* = 8 per group). **F** Kaplan–Meier survival curve of *Pbld*^+/+^ and *Pbld*^−/−^ mice (*n* = 8 mice per group) challenged with SeV (10^8^PFU per mouse). **G** The representative H&E staining images of the lungs from *Pbld*^+/+^ and *Pbld*^−/−^ mice inoculated intranasally with SeV (10^8^PFU per mouse) or mock infection at 4 dpi. Data from three independent experiments and error bars are presented as the mean ± SEM. Significance was determined by two-way ANOVA in (**B**), Student’s *t*-test in (**C**, **D**, and **E**), and the log-rank (Mantel-Cox) test for (**F**), *P < 0.05, **P < 0.01, ***P < 0.001.

To further elucidate the functional significance of PBLD in the host’s antiviral response, we intranasally infected *Pbld*-deficient mice with SeV. Work model to illustrate how *Pbld* deficiency mice are more susceptible to virus infection (Fig. 9A). Consistently, *Pbld*-deficient mice displayed more body weight loss following SeV infection (Fig. 9B). Subsequently, we sacrificed the infected mice to measure the levels of antiviral cytokines and viral load in the lung. Consistent with these results, the induction of *Ifnβ*, *Isg15*, and *Ifitm3* production was significantly attenuated, while viral load and TCID_50_ were much higher in the lungs of *Pbld*^−/−^ mice than in control *Pbld*^+/+^ (wild-type, WT) mice (Fig. 9C-D). Similarly, the induction of IFN-β in the serum from *Pbld*^−/−^ mice was severely impaired (Fig. 9E). Furthermore, *Pbld*-deficient mice exhibited significantly higher susceptibility to SeV-induced lethality compared to control mice (Fig. 9F), further supporting the function of PBLD in host defense against viruses in vivo. Interestingly, the pathological analysis of Hematoxylin-eosin (H&E) stain revealed that *Pbld*-deficient mice displayed increased alveolar wall thickening, edema, and infiltration of inflammatory cells in their lungs, in response to SeV infection when compared to WT mice (Fig. 9G). Overall, these findings demonstrate that mice with *Pbld* deficiency are more susceptible to viral infection.

### PBLD-specific activator Cedrelone reduces SeV replication

**Fig. 10:**
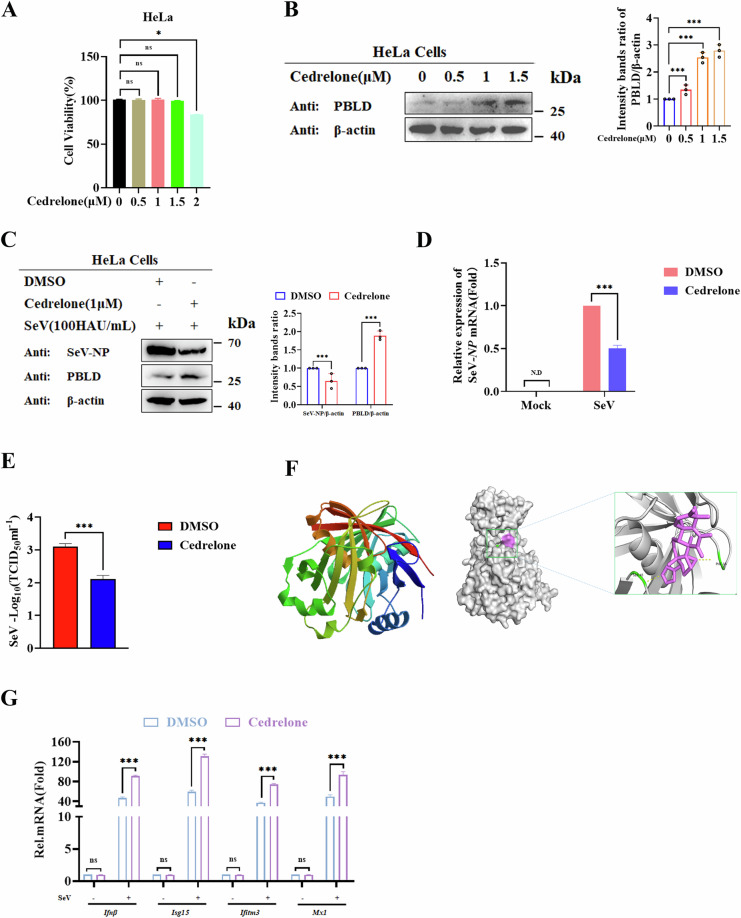
PBLD-specific activator Cedrelone has the ability to reduce SeV replication. **A** The different concentrations of Cedrelone (0 μM, 0.5 μM, 1 μM, 1.5 μM, and 2 μM) on HeLa cell viability were assessed by using the CCK-8 assay kit. **B** The different concentrations of Cedrelone (0 μM, 0.5 μM, 1 μM, 1.5 μM) on PBLD expression were detected through Western blot analysis. **C** HeLa cells were infected with SeV and treated with Cedrelone (1.0 μM) for 12 h, then the expression of PBLD and NP proteins was detected by Western blot analysis. **D** HeLa cells were infected with SeV and treated with Cedrelone (1.0 μM) for 12 h, then the mRNA expression of *NP* gene was detected by RT-qPCR analysis. **E** Virus titer of SeV was determined by TCID_50_ after challenge with DMSO and Cedrelone (1.0 μM) for 12 h. **F** The three-dimensional structures of PBLD were predicted by AlphaFold software. In silico docking of ligands with the PBLD protein was conducted. The PBLD protein is depicted in gray to highlight the binding cavity, Cedrelone is shown as sticks in pink color, and the yellow dotted line represents the coordination interactions between Cedrelone and protein atoms. **G** Real-time PCR analysis of the expression of IFNs and ISGs gene in SeV- infected HeLa cells upon challenge with DMSO and Cedrelone (1 μM), respectively. Data from three independent experiments and error bars are presented as the mean ± SEM. Significance was determined by one-way ANOVA in (**A** and **B**), two-way ANOVA in (**C**, **D**, and **G**), and Student’s *t*-test in (**E**). Ns, not significant, *P < 0.05, ***P < 0.001.

Cedrelone is a chemical activator of PBLD [6], however, its role in viral infection is yet unclear. Firstly, the effects of Cedrelone on cell viability were analyzed at concentrations ranging from 1 to 2 μM. The results showed that Cedrelone at a concentration of 1.5 μM did not show cytotoxic effects but 2 μM Cedrelone slightly affects cell viability (Fig. 10A). We treated HeLa cells with Cedrelone and found that it can enhance PBLD protein expression in dosage-dependent way (Fig. 10B). Subsequently, we assessed the antiviral and PBLD-activatory effects of Cedrelone after SeV infection. As a result, Cedrelone (1 μM) activated the expression of PBLD compared to the DMSO control but reduced the expression of SeV-NP protein (Fig. 10C). Furthermore, the viral gene copies were reduced by Cedrelone at a concentration of 1 μM, (Fig. 10D). Correspondingly, we found that Cedrelone (1 μM) markedly reduced the virus titer of SeV compared to DMSO control (Fig. 10E). These data consistently suggest that Cedrelone has the capacity to attenuate SeV productive infection. To investigate the potential interaction between PBLD and Cedrelone, a molecular docking study was conducted using AutoDock Vina software and visualized by PyMOL software. We found that PBLD docked to Cedrelone and formed hydrogen bonds with Cedrelone (Fig. 10F). Furthermore, we investigated the activating effects of Cedrelone on IFN-I response following stimulation with SeV. As expected, Cedrelone increased the expression of INFs *(Ifnβ)* and ISGs (*Isg15, Ifitm3*, *and Mx1*) in response to SeV infection, respectively (Fig. 10G). Taken together, these data suggest that Cedrelone specifically targeted PBLD and exerted an antiviral IFN-I immune response effect.

## Discussion

In this study, we first discovered the relationship between PBLD and the IFN-I signaling pathway and identified the activation of the transcription factor IRF3 is crucial for the PBLD-enhanced the production of type I IFNs. The IRF3 is a multifunctional and well-studied protein, but its role in the function of PBLD remains poorly understood. Furthermore, phosphorylation of IRF3 at S385/386 plays an important role in the PBLD-triggered IFN-I cascade. This finding is consistent with a previous study demonstrating that phosphorylation of IRF3 at Ser-385/386 and Ser-396, which are critical sites for virus-activated phosphorylation and IFN-I induction [37], indicating that PBLD partially influences IRF3 at the Ser residues 385 and 386. However, PBLD is unlikely a protein kinase. Instead, PBLD might be involved in recruiting IRF3 kinase(s) to increase IRF3 phosphorylation. Moreover, we found that PBLD significantly regulated IRF3 expression at the transcriptional level. However, as an important antiviral key signaling molecule, viral infection may keep modest IRF3 protein expression through protein translation and post-translational modifications such as the ubiquitin-proteasome pathway and caspase activity cleavage to avoid excessive immunity [40–42]. We used the online database to predict the transcription factors for IRF3. Unfortunately, no transcription factor has been found that effectively regulates IRF3 transcriptional expression by PBLD at present, and further research is needed to clarify this mechanism. Furthermore, we observed that *Pbld* deficiency reduced antiviral innate immunity in BMDMs, and PMs obtained from *Pbld*^*−/−*^ mice, facilitating the proliferation of BPIV3 and SeV. Remarkably, *Pbld*-deficient mice challenged with SeV infection showed a diminished innate immune response, rendering them more susceptible to viral infection. Therefore, our study provides a mechanism by which PBLD functions as a positive regulator in maintaining the antiviral immune response.

Besides the virus-induced type I IFN signaling pathway, apoptosis is a cellular mechanism that effectively sacrifices virus-infected cells to maintain homeostasis between the virus and host. In our study, we identified PBLD as a novel activator of the apoptosis branch of IRF3. Unlike its transcriptional function, IRF3 exhibits chaperone-like properties in virus-infected cells by facilitating the translocation of pro-apoptotic proteins to the mitochondria, ultimately triggering apoptosis. It has been reported that Tom70 induces the accumulation of IRF3 on mitochondria, and apoptosis-related protein Bax specifically interacted with IRF3 on mitochondria, thus inducing cell apoptosis [43, 44]. Surprisingly, we discovered that IRF3 interacts with the region the BH3 domain-containing Puma, (also known as BCL-2 binding component 3 (BBC3)), an apoptosis-related protein [45]. Our results demonstrated that PBLD facilitates the interaction between IRF3 and Puma, leading to their mitochondrial localization and subsequent apoptosis.

A growing body of research shows that Puma plays an important in triggering mitochondrial apoptosis [46]. PBLD facilitates the interplay between IRF3 and direct activators like Puma, forming oligomer complexes that might insert into mitochondrial outer membrane pores. This process leads to changes in mitochondrial osmotic pressure, loss of transmembrane potential, and induction of cell apoptosis. Furthermore, an increasing number of studies indicate that linear polyubiquitination of specific lysine residues (K193, K313/315) of IRF3 could induce apoptosis [31]. Indeed, RBCK1 could undergo ubiquitination modification at K313/315 of IRF3, disrupting its stable state [47]. This may also explain why the upregulation of IRF3 protein level is relatively modest, although PBLD significantly upregulates the transcription level of IRF3. Notably, our study highlighted that PBLD primarily contributes to apoptosis through K313/315 of IRF3, which provides a deeper understanding of the specific mechanism by which PBLD induces cell apoptosis through IRF3. However, whether PBLD promotes IRF3 interaction with E3 ligases needs further studies. Besides, we investigated PBLD could also induce the extrinsic apoptosis signaling pathways, but the specific mechanism is still further investigated.

Most importantly, our results have proved the role and specific molecular mechanism of PBLD in its novel function on virus replication via activating antiviral IFN-I responses and inducing apoptosis by regulating IRF3. This critical discovery is supported by several scenarios. Firstly, IRF3, upon activation, stimulates the expression of antiviral genes in innate immunity, which plays an important role in defending against viral infections [27]. Our study revealed that PBLD facilitates IFN-I response by activating IRF3, thereby limiting viral replication. Additionally, we uncovered that PBLD triggers apoptosis in an IRF3-mediated, transcription-independent pathway. Previous study has identified an IRF3-dependent apoptotic cell death called RIG-I induced apoptosis pathway (RIPA) which functions in antiviral defense [28, 31, 48]. This pathway, unrelated to interferon, has been implicated in combating various viruses, including RNA and DNA viruses [34, 49–52]. Furthermore, our mutational analyses demonstrated that both the transcriptional and apoptotic functions of IRF3 are crucial for PBLD’s antiviral effects on BPIV3 and VSV. Further research should explore PBLD’s role in combating other viral infections. Furthermore, the PBLD and IRF3 double knock out (PBLD/IRF3-KO) HeLa cell lines were successfully constructed (SFig. 9A). Transfection of PBLD or IRF3 in genetic knockout of both PBLD and IRF3 cell lines indicate that the expression of PBLD could attenuate viral replication compared with that of vector expression in the PBLD/IRF3-DKO HeLa cell lines (SFig. 9B), while cotransfection of PBLD with IRF3 significantly reduces the titer of BPIV3, VSV, SeV or HSV-1, demonstrating that PBLD suppresses virus replication partially depends on the IRF3. PBLD exerting antiviral effects through other pathways or molecules other than IRF3 need further study. Most importantly, Cedrelone, known for its involvement in various biological processes including colony formation, cell adhesion, migration, invasion, apoptosis, cell cycle arrest, and antitumor effects [6, 53, 54], also exhibits antiviral properties against SeV. Our study suggests that Cedrelone’s antiviral activity may be linked to the upregulation of PBLD and its facilitation of innate immune responses. This highlights PBLD as a promising target for antiviral drug development.

**Fig. 11:**
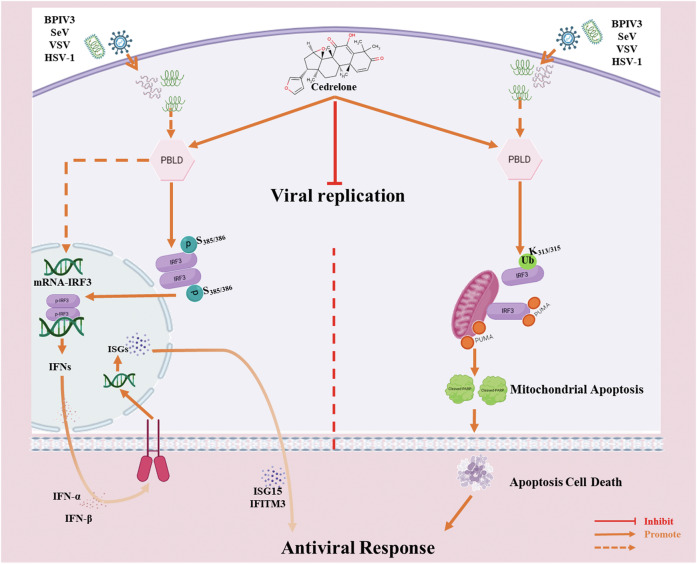
Schematic diagram showing that PBLD promotes IRF3-mediated the IFN-I response and apoptosis to inhibit viral replication, and Cedrelone has the ability to reduce SeV replication.

In summary, our findings, for the first time, demonstrate that PBLD acts as a positive regulator of the IFN-I response and apoptosis by upregulating IRF3 expression. Moreover, we have discovered that PBLD promotes IFN-I production by specifically phosphorylating IRF3 at the S385/386 residues. Furthermore, PBLD contributes to apoptosis by targeting the K313/315 residues of IRF3, which in turn strengthens the interaction between IRF3 and Puma and leads to their translocation to the mitochondria, resulting in mitochondrial apoptosis. Importantly, our study highlights the significance of PBLD in the overall antiviral defense by modulating the transcriptional and apoptotic activities of IRF3. Notably, Cedrelone, a chemical activator of PBLD, has the ability to reduce viral replication (Fig. 11). These findings provide valuable insights for the development of innovative therapeutic strategies and the design of targeted, broad-spectrum antiviral agents.

## Methods and materials

### Cell culture

Human 293T cells (ATCC, #CRL-3216), HeLa cells (ATCC, #CCL-2) were purchased from American Type Culture Collection and stored in our laboratory. Bone marrow-derived macrophages (BMDMs) were isolated from the femurs and tibias of 7–8-week-old male *Pbld*^+/+^ or *Pbld*^−^^/−^ mice. These cells were stimulated with 20 ng/ml of macrophage colony-stimulating factor (GM-CSF), (Novoprotein, CJ46, Suzhou, China) for 7 days. Peritoneal macrophages (PMs) were harvested from 7–8-week-old male *Pbld* ^+/+^ or *Pbld*^−/−^ mice 4 days post- the intraperitoneal injection of starch solution (Sigma Aldrich, 232-679-6) in sterile PBS. All cells were cultured in Dulbecco’s modified Eagle’s medium (DMEM; VivaCell, C3110-0500) supplemented with 10% fetal bovine serum (FBS; TransGen Biotech, FS301-02), 100 IU/ml penicillin, and 100 μg/ml streptomycin (Procell, PB180120) in a humidified incubator at 37 °C with 5% CO_2_.

### Mice

Both the *Pbld*2 and *Pbld*1 genes encode PBLD proteins in mice, as reported previously [55]. A murine deficient in *Pbld2* and *Pbld1* (*Pbld*^+/−^) with a C57BL/6 background was successfully generated using the CRISPR/Cas9 gene editing system with the help of Guangzhou Cyagen Biosciences Inc. To obtain *Pbld*^*+/+*^ and *Pbld*^−/−^ mice, *Pbld*^+/−^ mice were crossed. The genotype of the *Pbld*^*+/+*^ mice was confirmed by sequencing of PCR fragments amplified from the genomic DNA isolated from tails and western blotting analysis of PBLD protein expression in tissues. The primers used for this analysis are listed in Supplementary Table 1. 7–8-week-old C57BL/6 (B6) (*Pbld*^+/+^) and PBLD knock-out (*Pbld*^−/−^) mice used in the experiments were randomly grouped. All mice were housed under specific-pathogen-free conditions at Shandong Normal University, and all animal studies conformed to the Institutional Animal Care and Use Committee of Shandong Normal University.

### Viruses

Bovine parainfluenza virus type 3 (BPIV3) was isolated and stored in the Ruminant Disease Research Center, Shandong Normal University. Vesicular stomatitis virus (VSV), Sendai virus (SeV), Herpes simplex virus (HSV-1) were kindly provided by Prof. Yuwei Gao (Changchun Veterinary Research Institute) and Prof. Xuexing Zheng (Shandong University).

### Antibodies and reagents

Monoclonal antibodies against IRF3 (CY5779), phospho-IRF3 (S386) (CY6575), β-actin (AY0573), ISG15 (CY9357), Fragilis (CY7091), Puma (CY5460), Noxa (CY6774), Bcl2 (CY6717), Bax (CY5059), Cytochrome C (CY5734) and cleaved PARP (CY5035) were obtained from Abways Biotechnology Co., Ltd. Rabbit anti-DYKDDDDK Tag (D6W5B) (#14793), rabbit anti-HA Tag (C29F4) (#3724), Caspase-3 (#9662), Cleaved Caspase-9 (Asp315) (#9505), Caspase-8(D35G2) (#4790), Cleaved Caspase-8 (Asp374) (18C8) (#9496) antibody were purchased from Cell Signaling Technology. Rabbit polyclonal antibody to TOMM20 (#AF5206) was obtained from Affinity Biosciences. The PBLD polyclonal antibody (27891-1-AP) was purchased Proteintech. SeV-NP antibody was prepared and stored in the Ruminant Disease Research Center, Shandong Normal University. The secondary antibody goat anti-rabbit IgG-horseradish peroxidase (HRP) (#111-005-003) and Goat anti-mouse IgG-HRP secondary antibody (#115-035-003) were purchased from Jackson Immunoresearch. Cedrelone (1254-85-9) was purchased from TargetMOI. Dimethyl sulfoxide (DMSO, #67-68-5) was purchased from Sigma-Aldrich. Z-VAD-FMK (pan-caspase inhibitor, HY-16658B) and CCCP (Carbonyl cyanide 3-chlorophenylhydrazone, HY-100941) were purchased from Med Chem Express (MCE). Puromycin (#P8230) was purchased from Solarbio. Halt™ Protease Inhibitor Cocktail, EDTA-Free (100×, P002), and RIPA lysis buffer (WB3100) were purchased from New Cell and Molecular Biotech.

### Plasmids construction and small interfering RNAs

Recombinant plasmids expressing Flag-tagged PBLD, HA-tagged IRF3, and its mutants IRF3 (K193, K313/315 and S385/386, S396) were constructed by PCR-based amplification of 293T cell cDNA using various primer sets. The amplified products were then cloned into the pCMV-Flag or pCMV-HA eukaryotic expression vector (Clontech). The lentivector-expressing PBLD was constructed by subcloning the PCR product of PBLD into the pLVX-IRES-Puro construct. All PCR primers used are provided in Table 1. All plasmids were confirmed by DNA sequencing (Sangon Biotech). The small interfering RNAs (siRNAs) targeting PBLD, IRF3 and scrambled control (siNC) were produced from GenePharma and the sequences are listed in Table 2.

**Table 1:** The primers sequences for amplifying genes used in this study.

Name	Sequences (5′−3′)
PBLD-Forward Primer-*Hind*III	aaagacgatgacgacaagctt *gccacc* ATGAAGCTTCCTATTTTCATAGCAGA
PBLD-Reverse Primer- *Hind*III	tgaattcgcggccgcaagctt CTAGGCTGTCAGTGTGCCCTCT
IRF3-Forward-Primer-*EcoR*I	*CCGGAATTC gccacc* ATGGGAACCCCAAAGCCACGGATCC
IRF3-Reverse-Primer-*Xho*I	*CCGCTCGAG* GGCTCTCCCCAGGGCCCTGGAA
IRF3-Reverse Primer-mutation(K193)	CAACAGCCG**g**TTCAGTGGGT
IRF3-Forward Primer-mutation(K193)	ACCCACTGAA**c**CGGCTGTTG
IRF3-Reverse Primer-mutation(K313/315)	CACGCCTCCTTC**g**TTGTCgTTGGGGACCTC
IRF3-Forward Primer-mutation(K313/315)	GAGGTCCCCAA**c**GACAA**c**GAAGGAGGCGTG
IRF3-Reverse Primer-mutation(S385/386)	AGTATTCTCCAGGG**c**GG**c**GGCACCCCCTAC
IRF3-Forward Primer-mutation(S385/386)	GTAGGGGGTGCC**g**CC**g**CCCTGGAGAATACT
IRF3-Reverse Primer-mutation(S396)	TGGGTGGCTGTTGG**c**AATGTGCAGGTC
IRF3-Forward Primer-mutation(S396)	GACCTGCACATT**g**CCAACAGCCACCCA
PBLD-Forward Primer-*EcoR*I	ggatctatttccggtgaattc *gccacc* ATG GACTACAAGGACGACGATGACAAG AAGCTTCCTATTTTCATAGCAGA
PBLD-Reverse Primer- *EcoR*I	agaactagtctcgaggaattcc CTAGGCTGTCAGTGTGCCCTCT

Lowercase English letters are homologous arm sequences, lowercase italicized letters are Kozak sequences, lowercase bold letters are mutant amino acid bases, and uppercase italicized letters are protective bases and cleavage sites.

**Table 2:** The Oligo sequences used in this study.

Name	No.		Sequences (5′−3′)
sgIRF3	1#	Oligo1	CACCGTTGGAAGCACGGCCTACGGC
		Oligo2	AAACGCCGTAGGCCGTGCTTCCAAC
	2#	Oligo1	CACCGGCCACGGATCCTGCCCTGGC
		Oligo2	AAACGCCAGGGCAGGATCCGTGGCC
	3#	Oligo1	CACCGCGGATCCTGCCCTGGCTGGT
		Oligo2	AAACACCAGCCAGGGCAGGATCCGC
	4#	Oligo1	CACCGTGGCTGGTGTCGCAGCTGGA
		Oligo2	AAACTCCAGCTGCGACACCAGCCAC
sgPuma	1#	Oligo1	CACCGCAGGGCTGCTTCCACGACGT
		Oligo2	AAACACGTCGTGGAAGCAGCCCTGC
	2#	Oligo1	CACCGAGGACACTGCCGAGGGCACC
		Oligo2	AAACGGTGCCCTCGGCAGTGTCCTC
	3#	Oligo1	CACCGCTGGCAGGGGACCCACGTCG
		Oligo2	AAACCGACGTGGGTCCCCTGCCAGC
	4#	Oligo1	CACCGCACGTCGTGGAAGCAGCCCT
		Oligo2	AAACAGGGCTGCTTCCACGACGTGC
	5#	Oligo1	CACCGCCAGGGCTGCTTCCACGACG
		Oligo2	AAACCGTCGTGGAAGCAGCCCTGGC
	6#	Oligo1	CACCGATGTCCATGCCAGGTGCCCA
		Oligo2	AAACTGGGCACCTGGCATGGACATC
siPBLD	1#		CCGACAGACAACUUUGCACAA
	2#		GACGUUUACAACAGGUCGUUU
	3#		CCGACAGACAACUUUGCACAA
	4#		GACUUGAUAAAGACUGCCAUA
siIRF3			AGGCCUACCUGCAGGACUUTT
siNC			UUCUCCGAACGUGUCACGUTT

### Generation of IRF3 or Puma knockout (KO) HeLa cell lines through CRISPR‒Cas9 system

HeLa cell lines harboring IRF3 or Puma gene knockouts were established using the lenti-CRISPR/Cas9-V2 system as previously reported [56]. The specific guide RNA sequences for targeting IRF3 and Puma are listed in Table 2. After treatment with puromycin, single clonal knockout cells were isolated and confirmed by immunoblot analysis.

### Real-time PCR assay

Total cellular and tissue RNA was isolated and purified using Total RNA Isolation Kit (FOREGENE, RE-03111), followed by reverse-transcribed with the SuperScript ^III^ first-strand synthesis kit (Accurate, AG11706) according to the manufacturer’s protocol. Real-time PCR (RT-qPCR) was performed with SYBR Green PCR Master Mix (Accurate, AG11739) on a Roche 480 Detection System (Roche), with primer sequences listed in Table 3. The 2^−ΔΔCt^ method was used to calculate relative gene expression changes. The data were normalized to the expression level of β-actin in each sample.

**Table 3:** The primers sequences for RT-qPCR used in this study.

Name	Sequences (5′−3′)
BPIV3-HN-F	AGGAAGTGTTGTGTCATCA
BPIV3-HN-R	TGTGTCTGTTGCGTAAGT
VSV-G-F	CAAGTCAAAATGCCCAAGAGTCACA
VSV-G-R	TTTCCTTGCATTGTTCTACAGATGG
SeV-P-F	TCAGGAGGAGGTGCTGTTATC
SeV-P-R	TTGGGCCTAGTACAACACTG
SeV-NP-F	CCAAGTGTGACTGATGATG
SeV-NP-R	GCTTATCTGTGTCCAATGAG
IRF3-F(Human)	AAATCTACGAGTTTGTGAA
IRF3-R(Human)	CTCATCCAGAATGTCTTC
IFN-α-F(Human)	CAAGCCCAGAAGTATCTGCAATATC
IFN-α-R(Human)	GCACCACCAGGACCATCAGT
IFN-β-F(Human)	GGACAGGATGAACTTTGACA
IFN-β-R(Human)	AGACATTAGCCAGGAGGTT
β-actin-F(Human)	GGAAATCGTGCGTGACATTAA
β-actin-R(Human)	AGGAAGGAAGGCTGGAAGAG
ISG56-F(Human)	TCAGGTCAAGGATAGTCTGGAG
ISG56-R(Human)	AGGTTGTGTATTCCCACACTGTA
IFITM3-F(Human)	AATCACACTGTCCAAACCT
IFITM3-R(Human)	CTCCTCCTTGAGCATCTC
Mx1-F(Human)	GGCCAGCAAGCGCATCT
Mx1-R(Human)	TGGAGCAAGCGCATCT
ISG15-F(Human)	TCTGAGCATCCTGGTGAG
ISG15-R(Human)	GAAGGTCAGCCAGAACAG
IFN-β-F(Mouse)	CAGCTCCAAGAAAGGACGAAC
IFN-β-R(Mouse)	GGCAGTGTAACTCTTCTGCAT
β-actin-F(Mouse)	CCACACCCGCCACCAGTTCG
β-actin-R(Mouse)	TACAGCCCGGGGAGCATCGT
ISG15-F(Mouse)	AGCCTCTGAGCATCCTGGTGAG
ISG15-R(Mouse)	AGCGTGTCTACAGTCTGCGTCA
IFITM3-F(Mouse)	TGATCAACATGCCCAGAGATG
IFITM3-R(Mouse)	AGCCCAGGCAGCAGAAGTT

*F* forward primer, *R* reverse primer.

### Immunoprecipitation (IP) and immunoblotting

The cells were transfected with the indicated plasmids as described and then infected with BPIV3 for 24 h. Following three washes with ice-cold PBS (Shandong Sparkjade Biotechnology, CR0014), the cells were lysed at 4 °C for 30 min using nondenature lysis buffer (Solarbio, R0030) containing the protease inhibitor cocktail (New cell & Molecular Biotech, P002). The cell lysates were then centrifuged at 12,000 × *g* at 4 °C for 15 min. The supernatant of cell lysates was incubated with HA antibodies for 12 h at 4 °C, followed by further incubation with protein A/G resin for 4 h. After washing the beads with lysis buffer, the bound proteins were suspended in 1x sample buffer and boiled for 5 min. Then the proteins were separated by SDS-PAGE gel (Epizyme, PG113) and transferred to a polyvinylidene difluoride (PVDF) (Millipore, IPVH00010) membrane. After blocking with 5% skimmed dried milk (BioFroxx, 1172) in TBST (Epizyme Biotech, PS103), the membranes were incubated overnight with the desired primary antibodies at 4 °C. Subsequently, HRP-conjugated secondary antibody (Jackson immunoresearch, 115–035-003 or 111–005-003) were probed for 1 h at room temperature, and the immunoreactive signals were visualized using enhanced chemiluminescence (ECL) detection (Shandong Sparkjade Biotechnology, ED0015-C).

### Separation of mitochondria

HeLa cells were transfected with either the pCMV control vector or Flag-PBLD for 24 h. After that, the cells were infected with BPIV3 (MOI = 1) and incubated for an additional 24 h. The cells were then rinsed with ice-cold PBS, and mitochondria were isolated using the Thermo Scientific kit (#89874) as per the manufacturer’s instructions. Subsequently, the resulting cytosolic and mitochondrial fractions were subjected to SDS-PAGE and immunoblot analysis. β-actin and TOMM20 were utilized as markers for the cytosolic and mitochondrial fractions, respectively.

### Flow cytometry

HeLa cells were transfected with either the pCMV control vector or Flag-PBLD for 24 h using attractene transfection reagent (Qiagen, Germany, 301007). Subsequently, the cells were infected with BPIV3 (MOI = 1) or SeV (100HAU/mL), respectively. After 24 h of infection, the cells were washed with cold PBS, and trypsinized. Cell apoptosis was detected with the Annexin V-PE/7-AAD Apoptosis Detection Kit (Vazyme, Nanjing, China, A213-02) according to the manufacturer’s instructions. Fluorescence-activated cell sorting (FACS) data were subsequently analyzed using CellQuest software (BD Biosciences, San Jose, CA, USA).

### TUNEL assay

Apoptosis in PBLD-expressing HeLa cells was detected using a terminal deoxynucleotidyl transferase (TdT) dUTP nick-end labeling (TUNEL) assay kit (Boster Biological Technology (MK1016) according to the manufacturer’s instructions. Briefly, HeLa cells were transfected with GFP-PBLD or a vector control plasmid for 24 h and then infected with BPIV3 for another 24 h. After that, 4% paraformaldehyde was added to fix the cells. The samples were permeabilized with 0.25% Triton™ X-100 for 20 min at room temperature. Then, the TdT reaction mixture containing TdT and EdUTP was added to label fragmented DNA in the cells, followed by the addition of 50 μL TUNEL detection solution and DAPI staining (Beyotime, China, C1005). The samples were then analyzed using a confocal microscope (Leica DMIRBE inverted microscope).

### RNA-Seq based transcriptome analysis

HeLa cells were transfected with either the pCMV control vector or Flag-PBLD for 24 h. Then, the cells were infected with BPIV3 (MOI = 1) for another 24 h. Total RNA of cell samples was extracted using TRIzol Reagent. The RNA sequencing and analysis were conducted by NovoMagic. The differential expressed genes (DEGs) were defined with |log_2_FC| ≥ 1 and FDR < 0.1 (false discovery rate (FDR)). Pathway enrichment analyses of DEGs were conducted using the KEGG (https://www.genome.jp/kegg/).

### Analysis of apoptosis by western blotting

HeLa cells were seeded in six-well plates and transfected with the PBLD-expressing plasmid or corresponding control plasmids using Attractene transfection reagent (Qiagen, Germany, 301007) for the indicated time. Subsequently, the transfected cells were collected, lysed, and processed for SDS-PAGE analysis and western blotting with an apoptosis-associated antibody. The immunoreactive signals were probed using enhanced chemiluminescence (ECL) detection (Shandong Sparkjade Biotechnology Co., Ltd.).

### Enzyme-linked immunosorbent assay (ELISA)

Mouse IFN-β ELISA Kits (EK2236) provided by Multisciences (Lianke) Biotech were used to measure the concentrations of IFN-β in both cell culture supernatants and sera following the manufacturer’s instructions.

### Histopathology

7–8-week-old wild-type *Pbld*^+/+^ and *Pbld* -deficient (*Pbld*^−/−^) mice were challenge with SeV (10^8^PFU per mouse) intranasally for 4 d. For histological analysis, lung samples were fixed in 4% paraformaldehyde solution, embedded in paraffin, and sectioned according to standard procedures. 3 µm-thick sections were stained with hematoxylin and eosin (H&E) staining kit (Yeasen, 60524ES60) according to the manufacturer’s instructions. The extent of infiltration in the SeV-affected lung was evaluated by Caseviewer software for histological analysis.

### Statistical analysis

All data were processed using Microsoft Excel or GraphPad Prism 8.0. Results are displayed as the mean ± standard error of the mean (SEM) for three biological replicates. Statistical analyses, including the student’s *t*-test or one-way ANOVA or two-way ANOVA, as well as the log-rank (Mantel-Cox) test for mouse survival curve analysis, were conducted with GraphPad Prism 8.0 software (GraphPad Software, La Jolla, California, USA). The gray intensity of the bands in western blot from at least three independent experiments were analyzed using ImageJ software. Significance levels were set as follows: Ns: no significant difference, *p* > 0.05; *p < 0.05; **p < 0.01; ***p < 0.001.

## Supplementary information

Supplementary Figure 1

Supplementary Figure 2

Supplementary Figure 3

Supplementary Figure 4

Supplementary Figure 5

Supplementary Figure 6

Supplementary Figure 7

Supplementary Figure 8

Supplementary Figure 9

Supplementary Figure legends

original data

Supplementary Table 1

## Data Availability

The published article and its supplementary information files include all data generated or analyzed during this study. The RNA-seq data for this study have been deposited in the NCBI Sequenced Read Archive (SRA) under accession number SUB14496458.

## Materials availability

The materials supporting the current study are available from the corresponding author on request.
