# Supplementary figures and images for: PBLD promotes IRF3 mediated the type I interferon (IFN-I) response and apoptosis to inhibit viral replication
Source: Cell Death Dis. 2024 Oct 3;15(10):727. doi: 10.1038/s41419-024-07083-w (PMC11450232; doi:10.1038/s41419-024-07083-w)

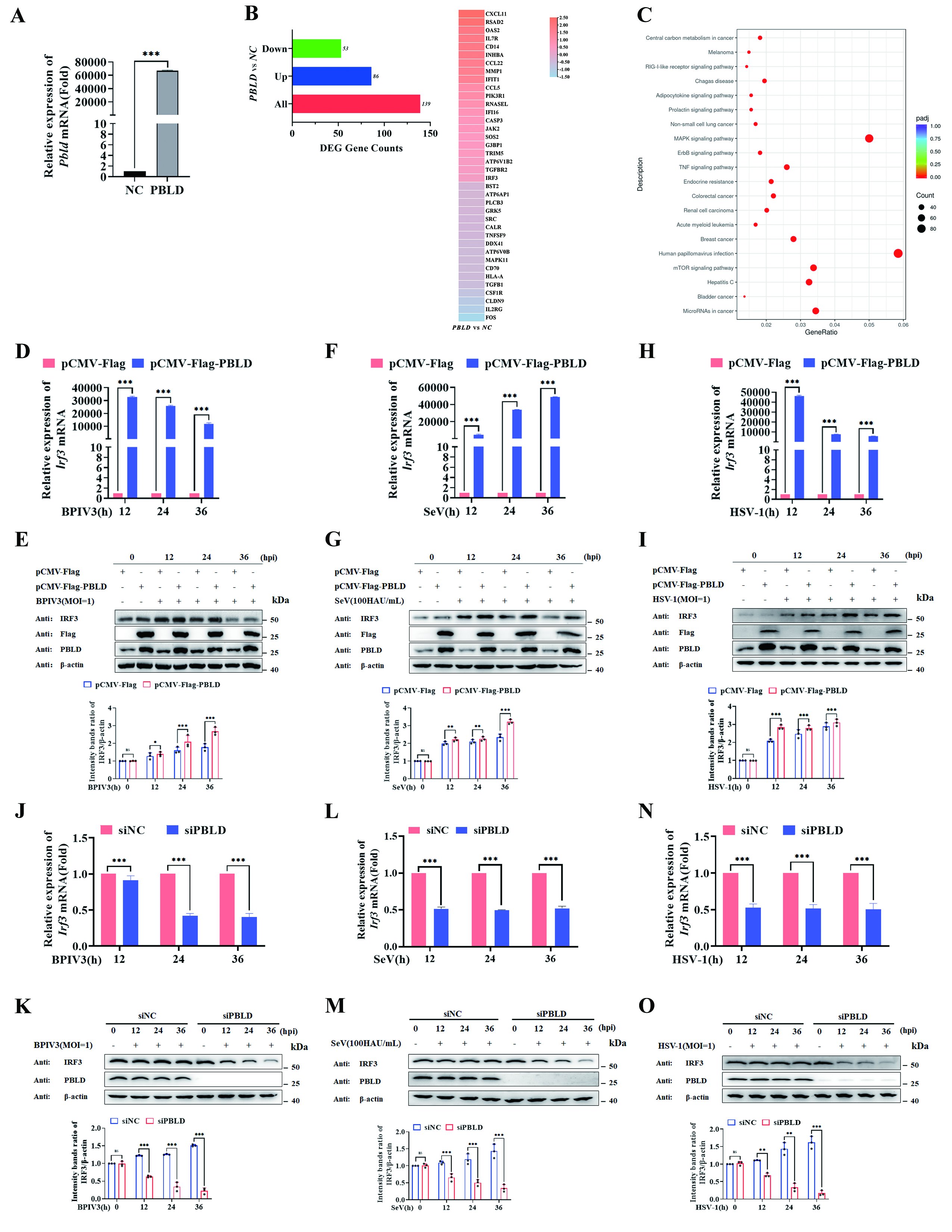

Supplement: Supplementary file 1 — Supplementary Figure 1 [file 41419_2024_7083_MOESM1_ESM.tif]

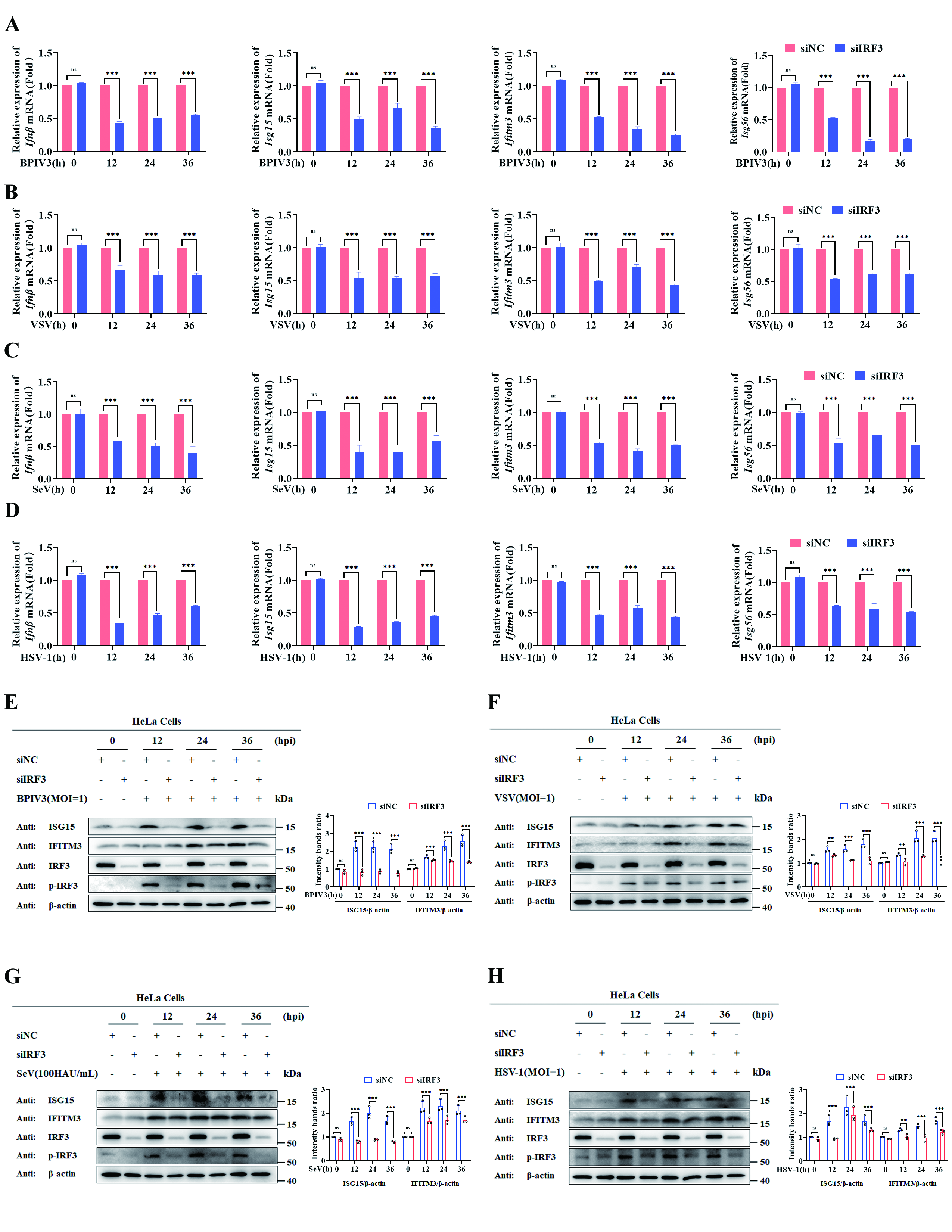

Supplement: Supplementary file 2 — Supplementary Figure 2 [file 41419_2024_7083_MOESM2_ESM.tif]

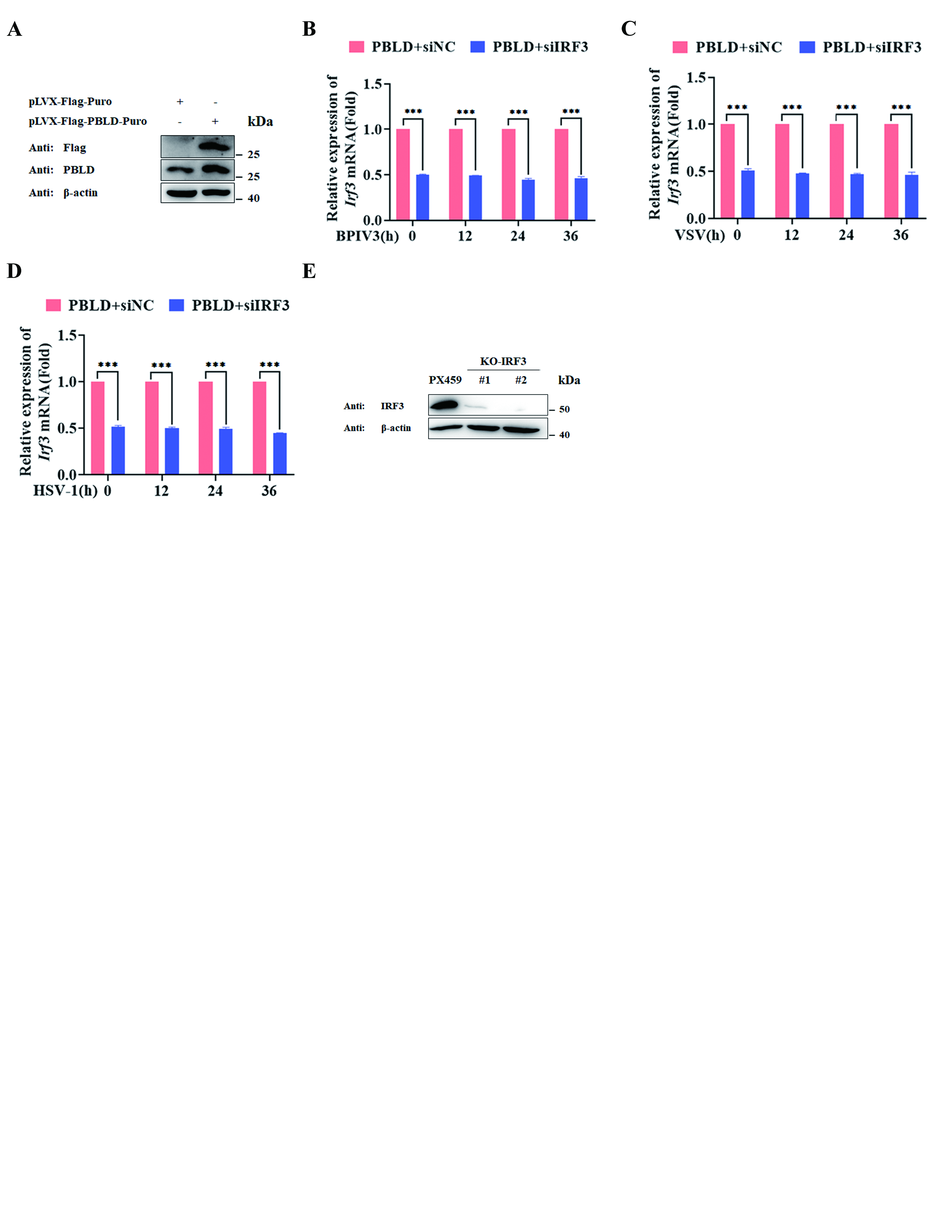

Supplement: Supplementary file 3 — Supplementary Figure 3 [file 41419_2024_7083_MOESM3_ESM.tif]

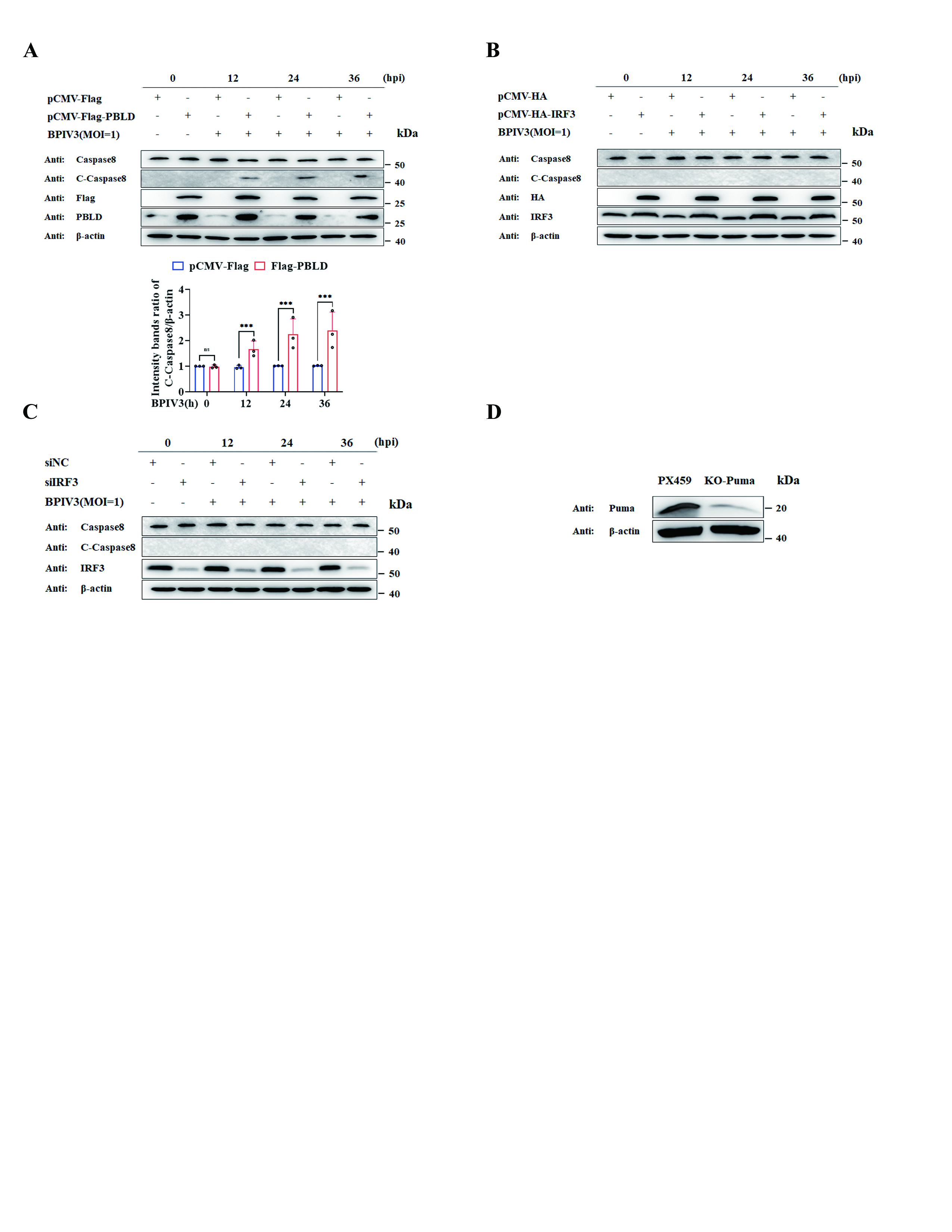

Supplement: Supplementary file 4 — Supplementary Figure 4 [file 41419_2024_7083_MOESM4_ESM.tif]

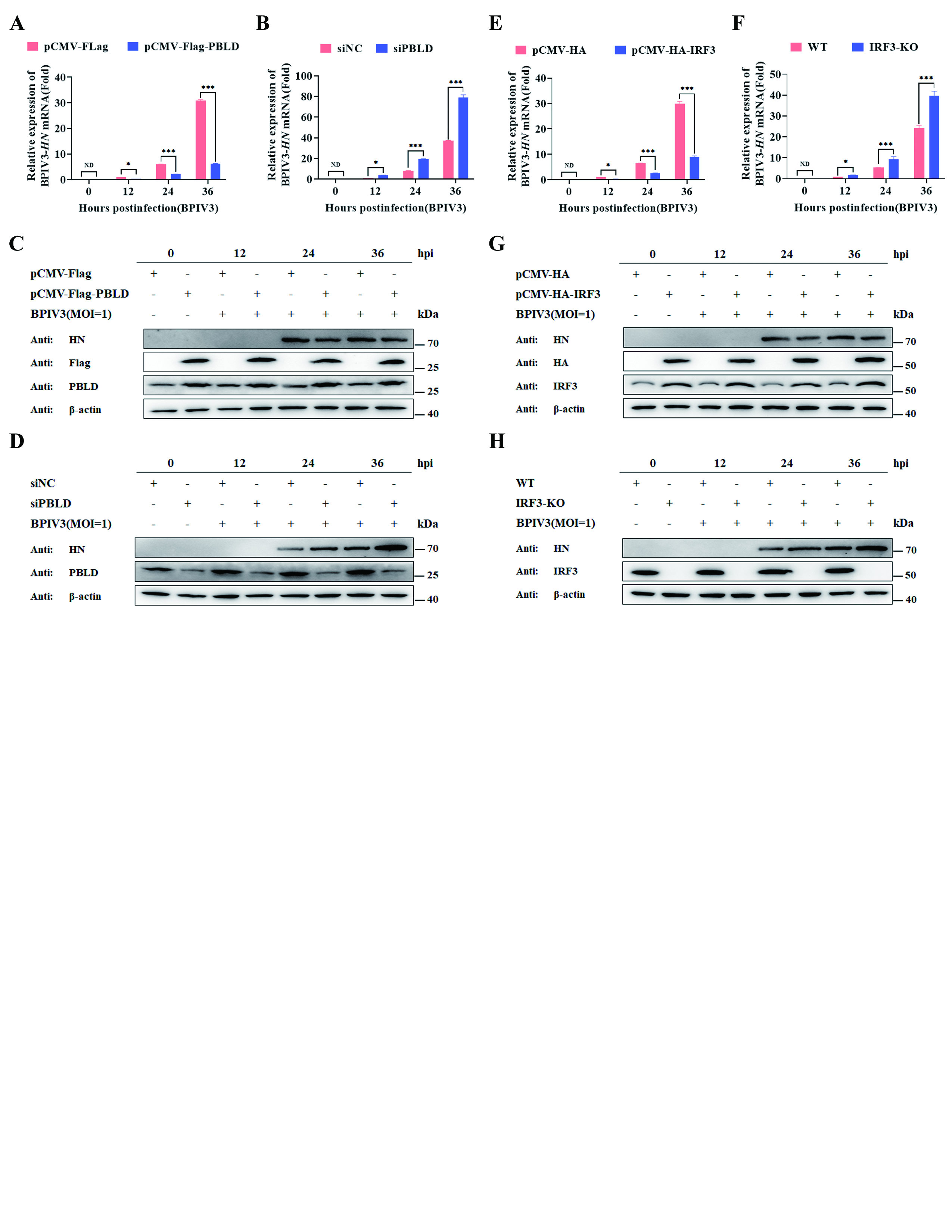

Supplement: Supplementary file 5 — Supplementary Figure 5 [file 41419_2024_7083_MOESM5_ESM.tif]

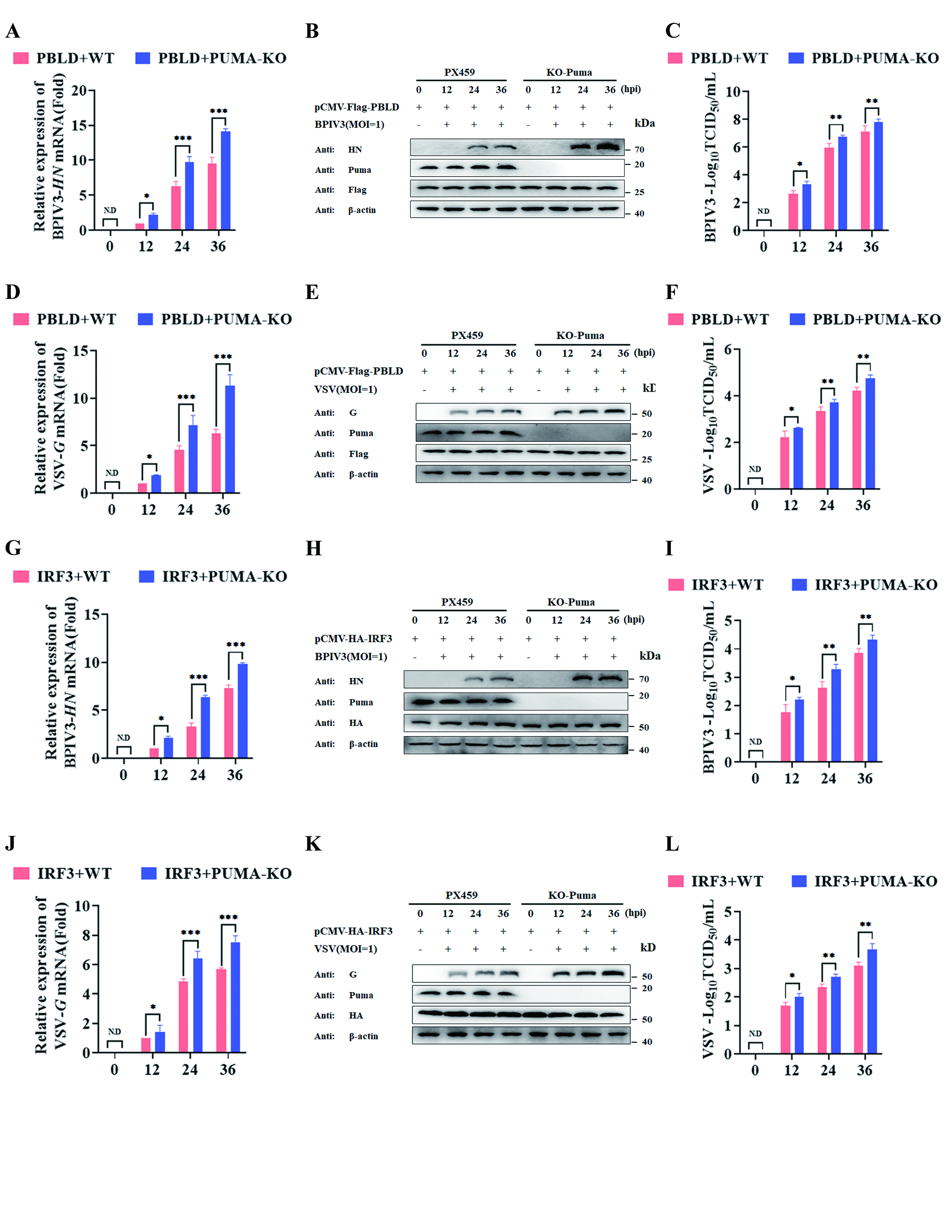

Supplement: Supplementary file 6 — Supplementary Figure 6 [file 41419_2024_7083_MOESM6_ESM.tif]

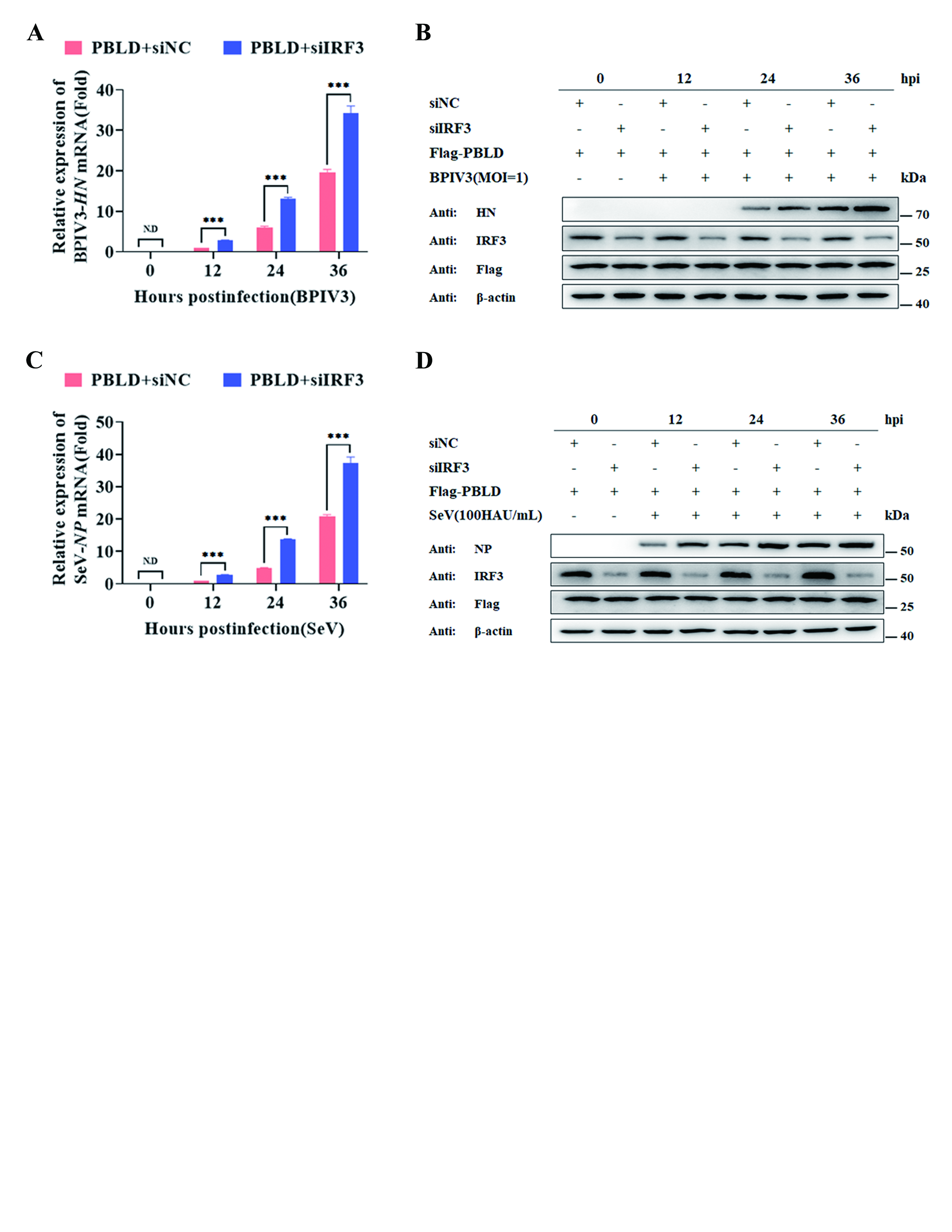

Supplement: Supplementary file 7 — Supplementary Figure 7 [file 41419_2024_7083_MOESM7_ESM.tif]

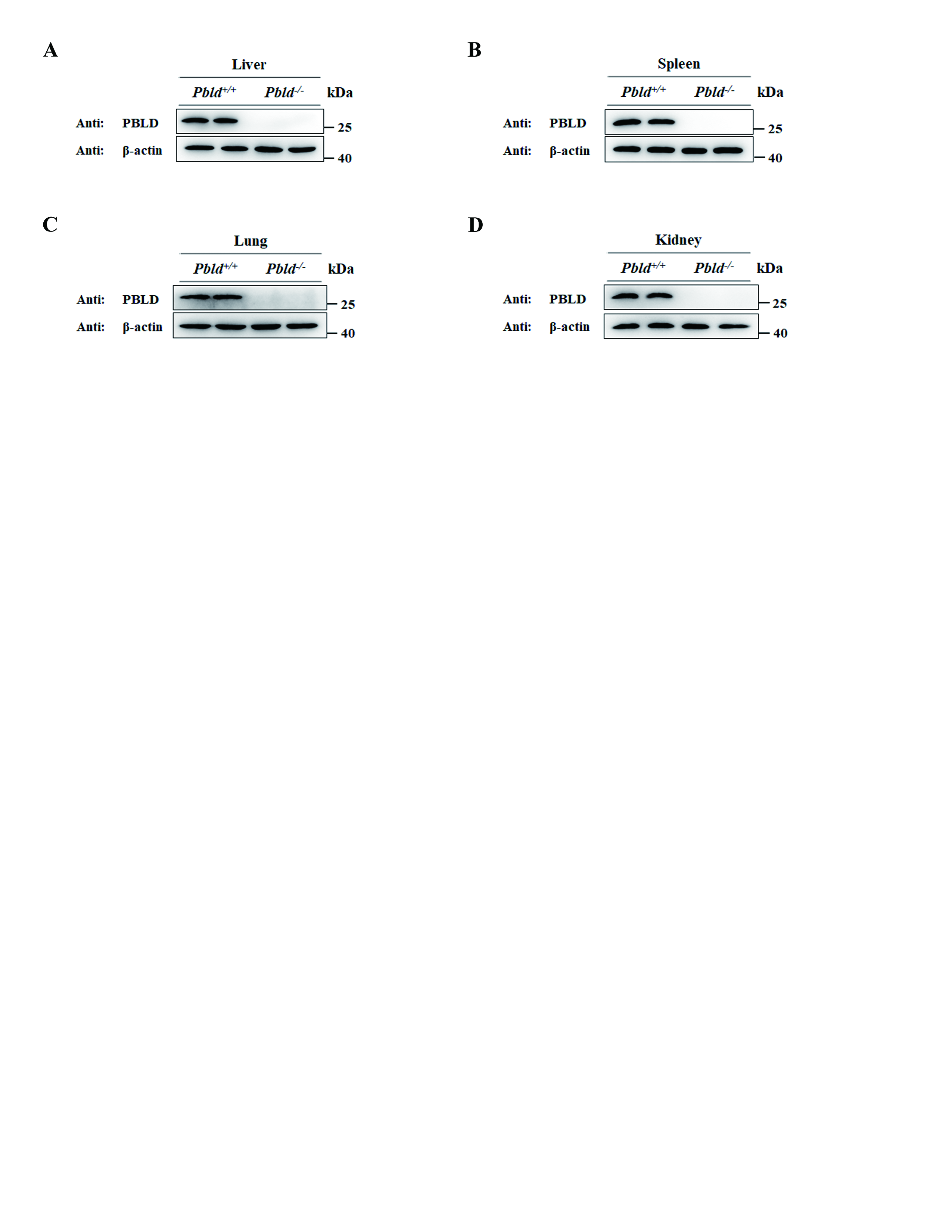

Supplement: Supplementary file 8 — Supplementary Figure 8 [file 41419_2024_7083_MOESM8_ESM.tif]

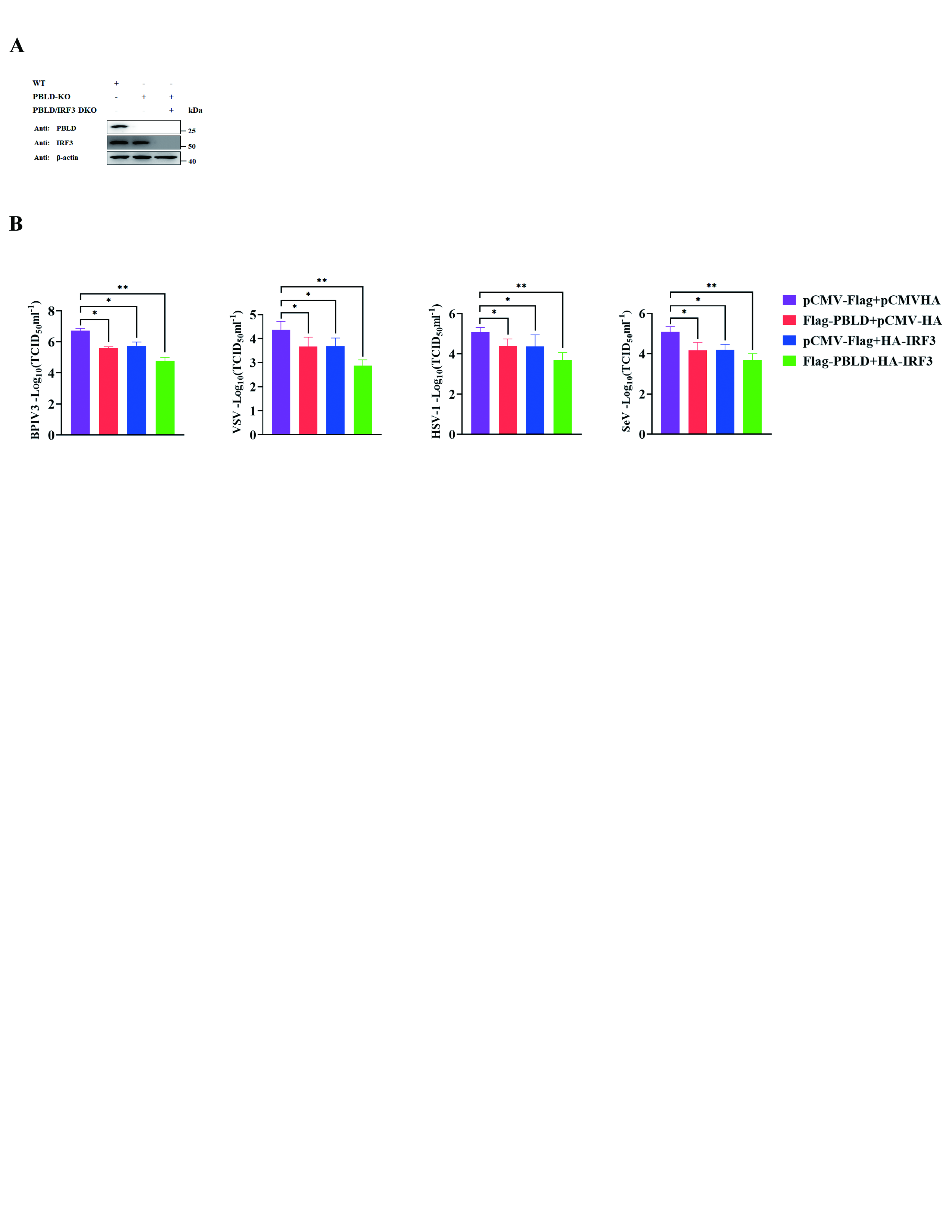

Supplement: Supplementary file 9 — Supplementary Figure 9 [file 41419_2024_7083_MOESM9_ESM.tif]
