# Supplementary material for: PBLD promotes IRF3 mediated the type I interferon (IFN-I) response and apoptosis to inhibit viral replication
Source: Cell Death Dis. 2024 Oct 3;15(10):727. doi: 10.1038/s41419-024-07083-w (PMC11450232; doi:10.1038/s41419-024-07083-w)
Supplement: Supplementary file 10 — Supplementary Figure legends [file 41419_2024_7083_MOESM10_ESM.docx]

**Supplementary Figure 1.** **Analyzing differentially expressed genes of PBLD-expressing HeLa cell lines after BPIV3 infection through RNA-Seq based transcriptome sequencing analysis.**

**A** Validation of PBLD overexpression in HeLa cell lines by RT-qPCR. Data information: The bars represent the mean±SEM of triplicate samples. Significance was determined by Student’s t-test, ***P < 0.001.

**B** The number of differentially expressed genes between vector control (NC) or PBLD expressing (PBLD) HeLa cell lines stimulated with BPIV3 (MOI=1) for 24 h. The heatmap showing the main upregulated and downregulated DEGs in immunity-related signaling pathways.

**C** Significantly enriched Kyoto Encyclopedia of Genes and Genomes (KEGG) pathway of upregulated and downregulated DEGs (FDR<0.01).

**D-O PBLD upregulates the IRF3 expression.**

**D-H** RT-qPCR analysis of the mRNA expression of IRF3 in Flag-PBLD expressed cells upon BPIV3, SeV and HSV-1 infection for the indicated hours.

**E-I** Representative Western blot analysis of the IRF3 protein expression in Flag-PBLD expressed cells after infection with BPIV3, SeV and HSV-1 for the indicated hours.

**J-N** RT-qPCR analysis of the mRNA expression of IRF3 in HeLa cells treated with PBLD-specific siRNA or scrambled siRNA (siNC) following BPIV3, SeV and HSV-1 infection for the indicated hours.

**K-O** Representative Western blot analysis of the IRF3 protein expression in HeLa cells treated with PBLD-specific siRNA or scrambled siRNA (siNC), followed by infection with BPIV3, SeV and HSV-1 for the indicated hours.

Data information: The bars represent the mean±SEM of triplicate samples. Significance was determined by two-way ANOVA. Ns, not significant; *P < 0.05; **P < 0.01; ***P < 0.001.

**Supplementary Figure 2. Knockdown of IRF3 reduces the virus-induced IFN-I signaling pathway.**

**A-D** HeLa cells were transfected with IRF3-specific siRNA (siIRF3) or siNC, and then infected with BPIV3, VSV, SeV and HSV-1 at an MOI of 1 for 0, 12, 24 and 36 h, respectively. The mRNA levels of *Ifnβ* and ISGs (*Isg15*, *Ifitm3* and *Isg56*) were then analyzed by RT-qPCR.

**E-H** Representative Western blot analysis of the ISGs (ISG15 and IFITM3) in HeLa cells with IRF3 knockdown in the content of BPIV3, VSV, SeV and HSV-1 infection for 0, 12, 24 and 36 h, respectively.

Data from three independent experiments and error bars are presented as the mean ± SEM. Significance was determined by two-way ANOVA. Ns, not significant; *P < 0.05; **P < 0.01; ***P < 0.001.

**Supplementary Figure 3. The expression level of IRF3 after supplementing the overexpressed PBLD protein in siIRF3 cells.**

**A** Identification of PBLD overexpression HeLa cell lines by immunoblot analysis.

**B-D** RT-qPCR analysis of the mRNA expression of IRF3 in HeLa cells with stable expression of PBLD after transfection with IRF3-specific siRNA (siIRF3) or siNC, and then infected with BPIV3, VSV and HSV-1 at an MOI of 1 for 0, 12, 24 and 36 h, respectively.

**E** Identification of IRF3 knockout HeLa cell lines by immunoblot analysis.

Data information: The bars represent the mean±SEM of triplicate samples. Significance was determined by two-way ANOVA. ***P < 0.001.

**Supplementary Figure 4. PBLD promotes virus-induced extrinsic apoptosis pathway in an IRF3 independent manner.**

**A** HeLa cells were transfected with Flag-PBLD or pCMV-Flag for 24 h, and then the cells were infected with BPIV3 (MOI=1) for the indicated time. The expression of extrinsic apoptotic proteins was determined by western blot analysis.

**B** Western blotting analysis of the expression of extrinsic apoptotic proteins in IRF3-overexpressed HeLa cells and infected with BPIV3 (MOI=1) at 0, 12, 24 and 36 hpi.

**C** HeLa cells were transfected with siIRF3 or scrambled siRNA (siNC) for 24 h, and then the cells were infected with BPIV3 (MOI=1) for the indicated time. The expression of extrinsic apoptotic proteins was determined by western blot analysis.

**D** Identification of PUMA knockout HeLa cell lines by immunoblot analysis.

Data represent one of three independent experiments or collected from at least three. The gray intensity of the bands in western blot from three independent experiments were analyzed using ImageJ software. Significance was determined by two-way ANOVA. Ns, not significant, *** P < 0.001.

**Supplementary Figure 5. The impact of PBLD and IRF3 on BPIV3 replication.**

**A, B** RT-qPCR analysis of the mRNA expression of BPIV3*-HN* in Flag-PBLD expressed HeLa cells or siPBLD HeLa cells and infected with BPIV3 (MOI=1) at 0, 12, 24 and 36 hpi.

**C, D** Western blotting analysis of the expression of BPIV3-HN proteins in Flag-PBLD expressed HeLa cells or siPBLD HeLa cells and infected with BPIV3 (MOI=1) at 0, 12, 24 and 36 hpi.

**E, F** RT-qPCR analysis of the mRNA expression of BPIV3*-HN* in HA-IRF3 expressed HeLa cells or IRF3-KO HeLa cells and infected with BPIV3 (MOI=1) at 0, 12, 24 and 36 hpi.

**G** Western blotting analysis of the expression of BPIV3-HN proteins in HA-IRF3 expressed HeLa cells or IRF3-KO HeLa cells and infected with BPIV3 (MOI=1) at 0, 12, 24 and 36 hpi.

Data information: The bars represent the mean±SEM of triplicate samples. Significance was determined by two-way ANOVA. * P <0.05; ***P < 0.001.

**Supplementary Figure 6. The inhibition of virus replication by PBLD or IRF3 is mediated through PUMA.**

**A-L** Puma knockout cell lines were transfected with Flag-PBLD or HA-IRF3, and infected with BPIV3 or VSV at a MOI of 1 for the indicated time. **A**, **D**, **G**, **J** RT-qPCR analysis of the mRNA expression of BPIV3*-HN* or VSV*-G*. **B**, **E**, **H**, **K** Western blotting analysis of the protein expression of BPIV3-HN or VSV-G. **C**, **F**, **I**, **L** The viral titer of BPIV3 or VSV was determined by TCID_50_ assay.

Data information: The bars represent the mean±SEM of triplicate samples. Significance was determined by two-way ANOVA. *P < 0.05; **P < 0.01; ***P < 0.001.

**Supplementary Figure 7. The inhibition of virus replication by PBLD is mediated through IRF3.**

**A-D** HeLa cells with stable expression of PBLD were transfected with IRF3-specific siRNA (siIRF3) or siNC, and then infected with BPIV3 and SeV at an MOI of 1 for 0, 12, 24 and 36 h, respectively. **A** and **C** RT-qPCR analysis of the mRNA expression of BPIV3-*HN* or SeV*-NP*. **B** and **D** Western blotting analysis of the protein expression of BPIV3-HN or SeV-NP.

Data information: The bars represent the mean±SEM of triplicate samples. Significance was determined by two-way ANOVA. ***P < 0.001.

**Supplementary Figure 8. Validation of PBLD** **deficiency expression in tissues from PBLD knockout mice.**

**A-D** The PBLD expression in tissues, including liver (**A**), spleen (**B**), lung (**C**) and kidney (**D**) from wild-type (*Pbld^+/+^*) and PBLD (*Pbld^-/-^*) knockout mice was determined by western blotting.

**Supplementary Figure 9. PBLD promotes virus replication via IRF3.**

**A** Identification of PBLD and IRF3 double knockout (DKO) HeLa cell lines by immunoblot analysis.

**B** The viral titer of BPIV3, VSV, HSV-1 and SeV was determined by TCID_50_ assay in PBLD/IRF3- DKO cell lines with transfected with Flag-PBLD, HA-IRF3 or Flag-PBLD and HA-IRF3.

The bars in **B** represent the mean±SEM of triplicate samples. Significance was determined by one-way ANOVA. *P < 0.05; **P < 0.01.
