# Supplementary material for: PBLD promotes IRF3 mediated the type I interferon (IFN-I) response and apoptosis to inhibit viral replication
Source: Cell Death Dis. 2024 Oct 3;15(10):727. doi: 10.1038/s41419-024-07083-w (PMC11450232; doi:10.1038/s41419-024-07083-w)

FIG1

Figure 1

B

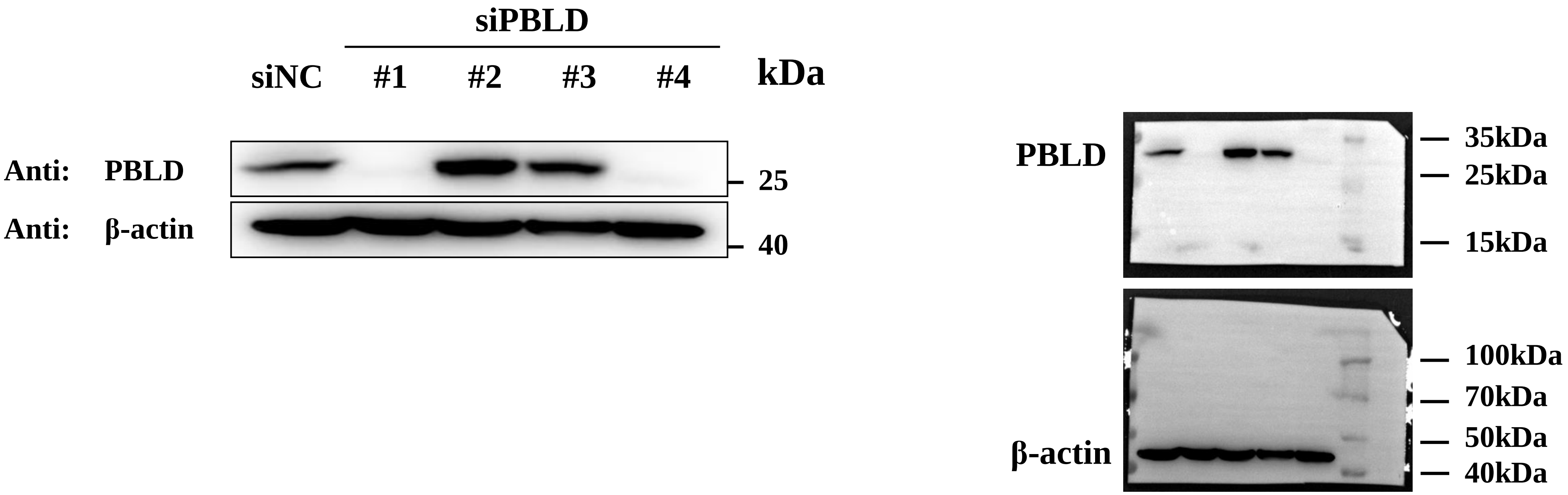
siPBLD
siNC
#1
#2
#3
#4
kDa
Anti: PBLD
Anti: β-actin
25
40
PBLD
β-actin
35kDa
25kDa
15kDa
100kDa
70kDa
50kDa
40kDa

Figure 1

D

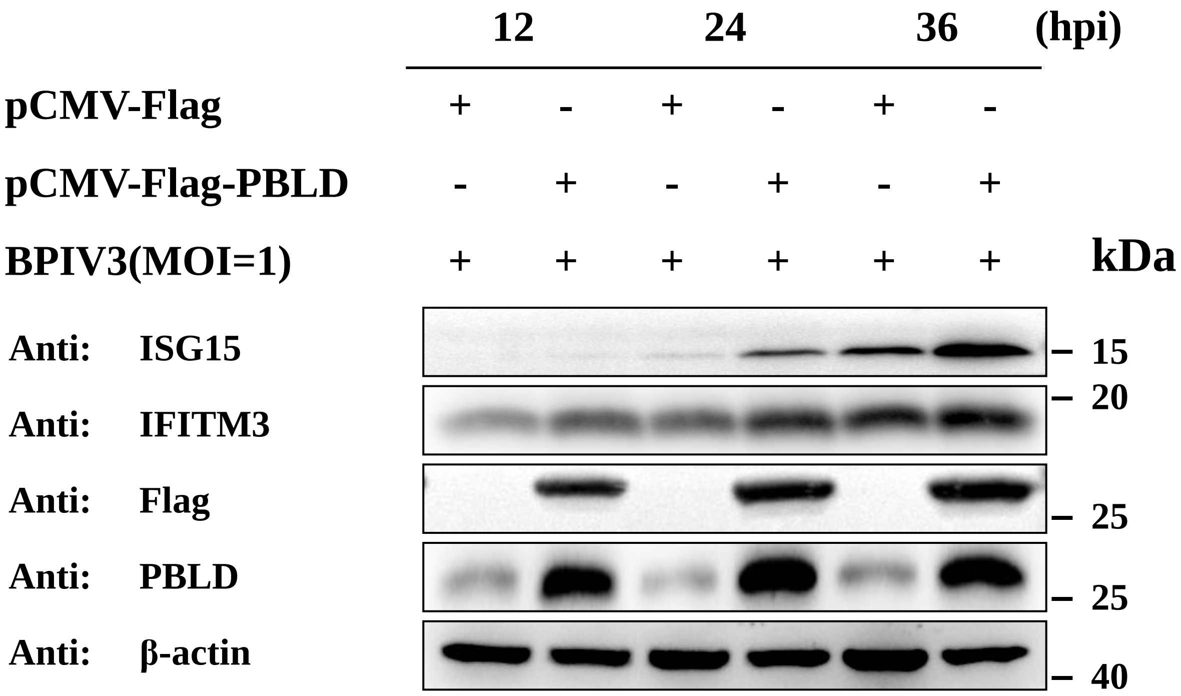

12
24
36
(hpi)
pCMV-Flag + - + - + -
pCMV-Flag-PBLD - + - + - +
BPIV3(MOI=1) + + + + + +
kDa
Anti: ISG15
Anti: IFITM3
Anti: Flag
Anti: PBLD
Anti: β-actin
15
20
25
25
40

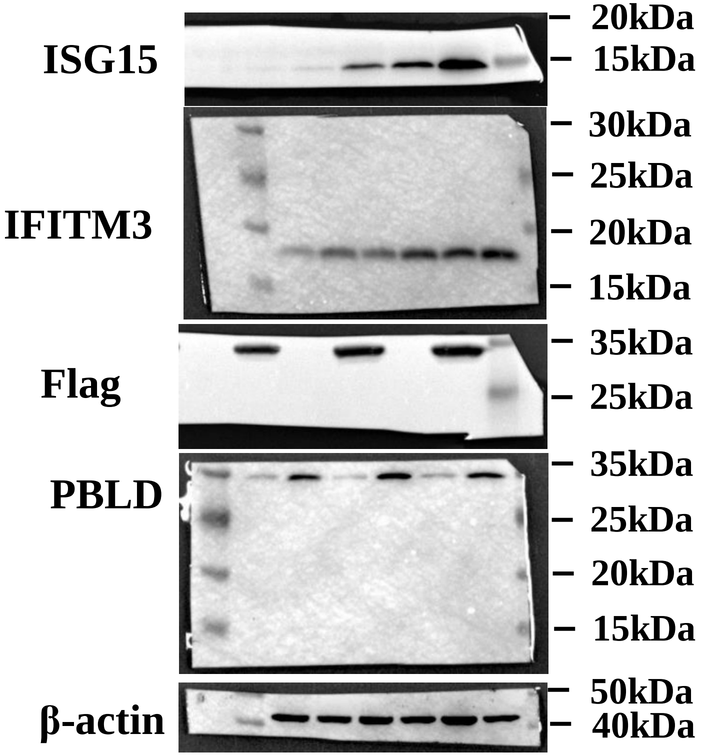

ISG15
20kDa
15kDa
IFITM3
30kDa
25kDa
20kDa
15kDa
Flag
35kDa
25kDa
PBLD
35kDa
25kDa
20kDa
15kDa
β-actin
50kDa
40kDa

Figure 1

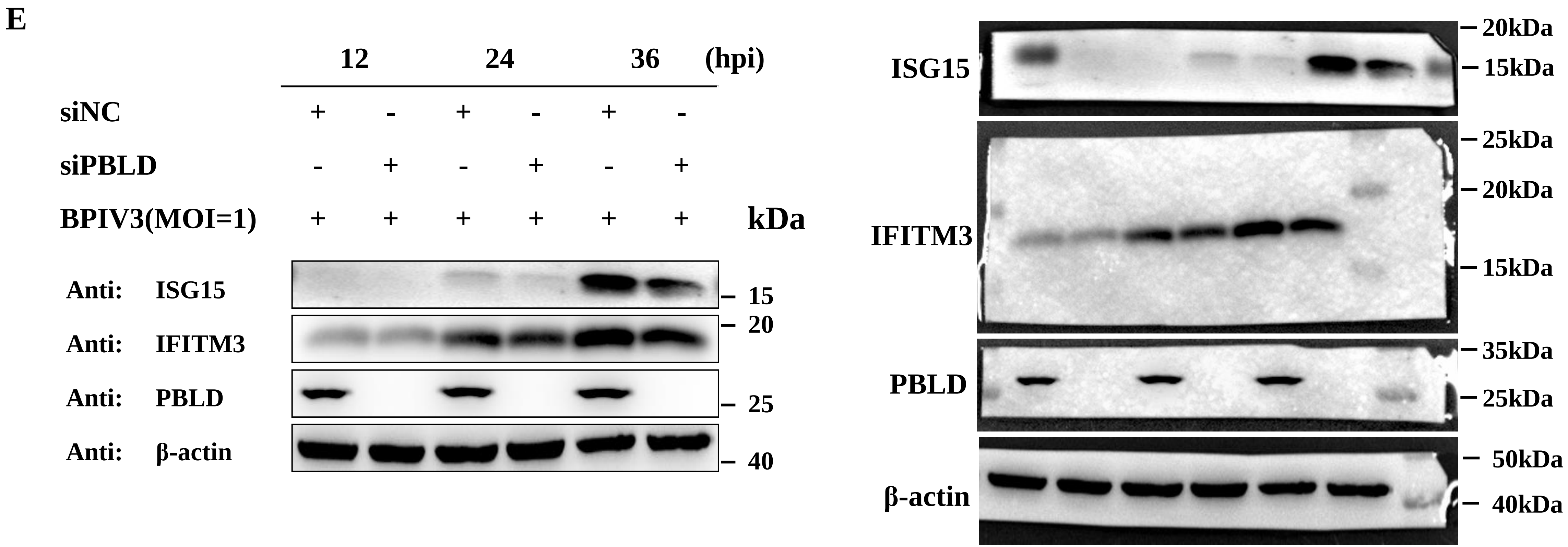
E
12
24
36
(hpi)
siNC
+ - + - + -
siPBLD
- + - + - +
BPIV3(MOI=1)
+ + + + + +
kDa
Anti: ISG15
Anti: IFITM3
Anti: PBLD
Anti: β-actin
15
20
25
40
ISG15
IFITM3
PBLD
β-actin
20kDa
15kDa
25kDa
20kDa
15kDa
35kDa
25kDa
50kDa
40kDa

Figure 1

H

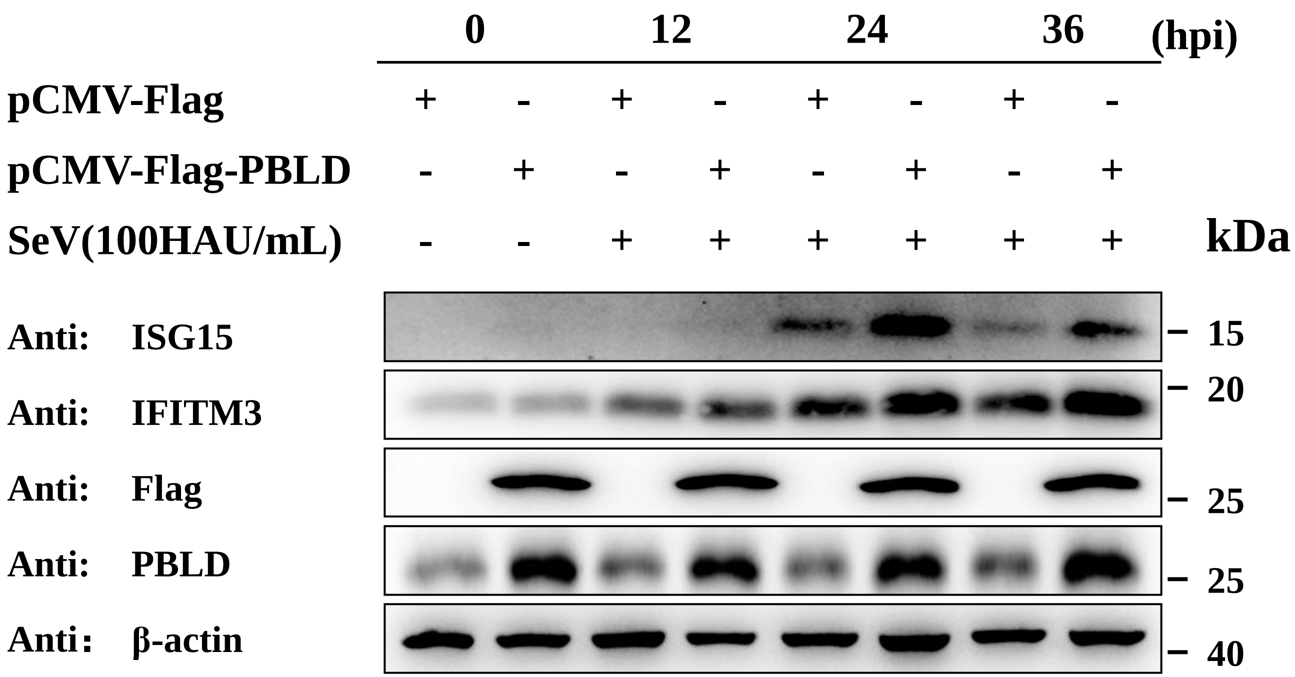
0
12
24
36
(hpi)
pCMV-Flag + - + - + - + -
pCMV-Flag-PBLD - + - + - + - +
SeV(100HAU/mL) - - + + + + + +
kDa
Anti: ISG15
Anti: IFITM3
Anti: Flag
Anti: PBLD
Anti: β-actin
15
20
25
25
40

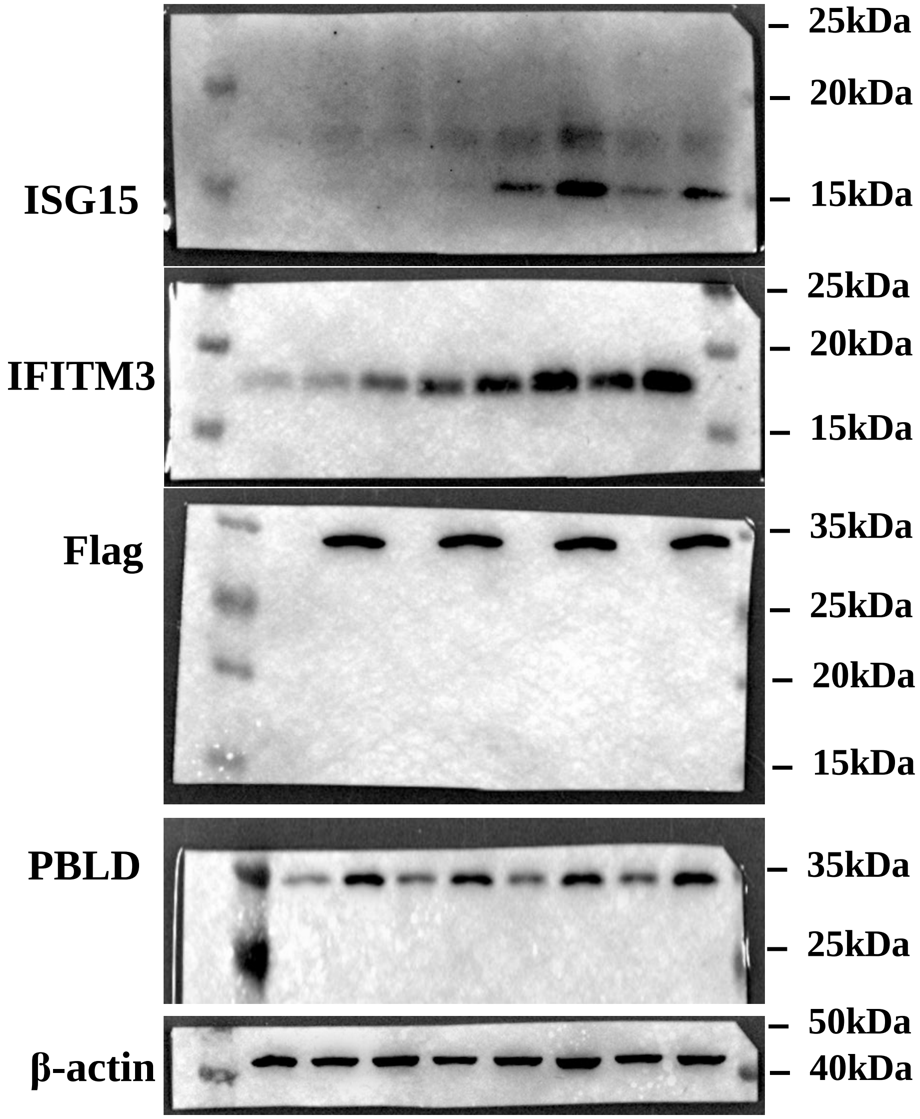
ISG15
IFITM3
Flag
PBLD
β-actin
25kDa
20kDa
15kDa
25kDa
20kDa
15kDa
35kDa
25kDa
20kDa
15kDa
35kDa
25kDa
50kDa
40kDa

Figure 1

I

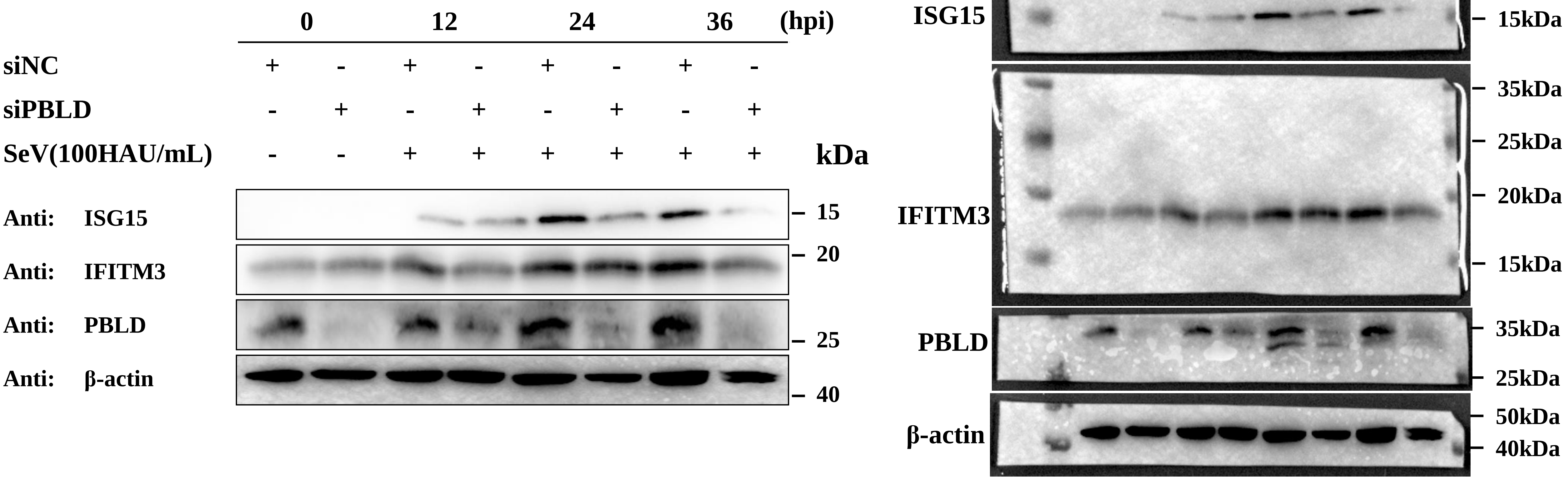
0
12
24
36
(hpi)
siNC
siPBLD
SeV(100HAU/mL)
kDa
Anti: ISG15
Anti: IFITM3
Anti: PBLD
Anti: β-actin
15
20
25
40
ISG15
IFITM3
PBLD
β-actin
35kDa
25kDa
20kDa
15kDa
50kDa
40kDa

FIG2

Figure 2

D

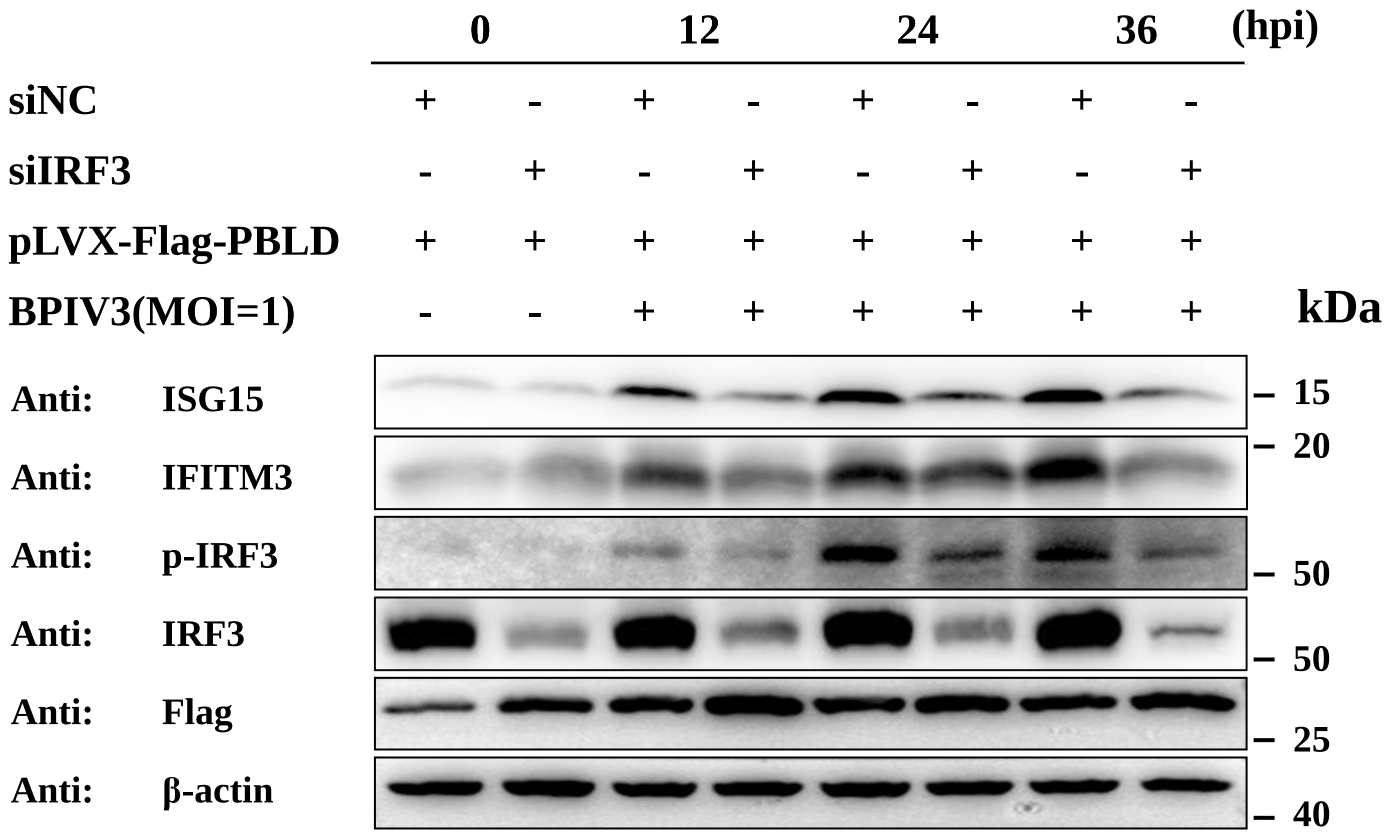

0
12
24
36
(hpi)
siNC + - + - + - + -
siIRF3 - + - + - + - +
pLVX-Flag-PBLD + + + + + + + +
BPIV3(MOI=1) - - + + + + + +
kDa
Anti: ISG15
Anti: IFITM3
Anti: p-IRF3
Anti: IRF3
Anti: Flag
Anti: β-actin
15
20
50
50
25
40

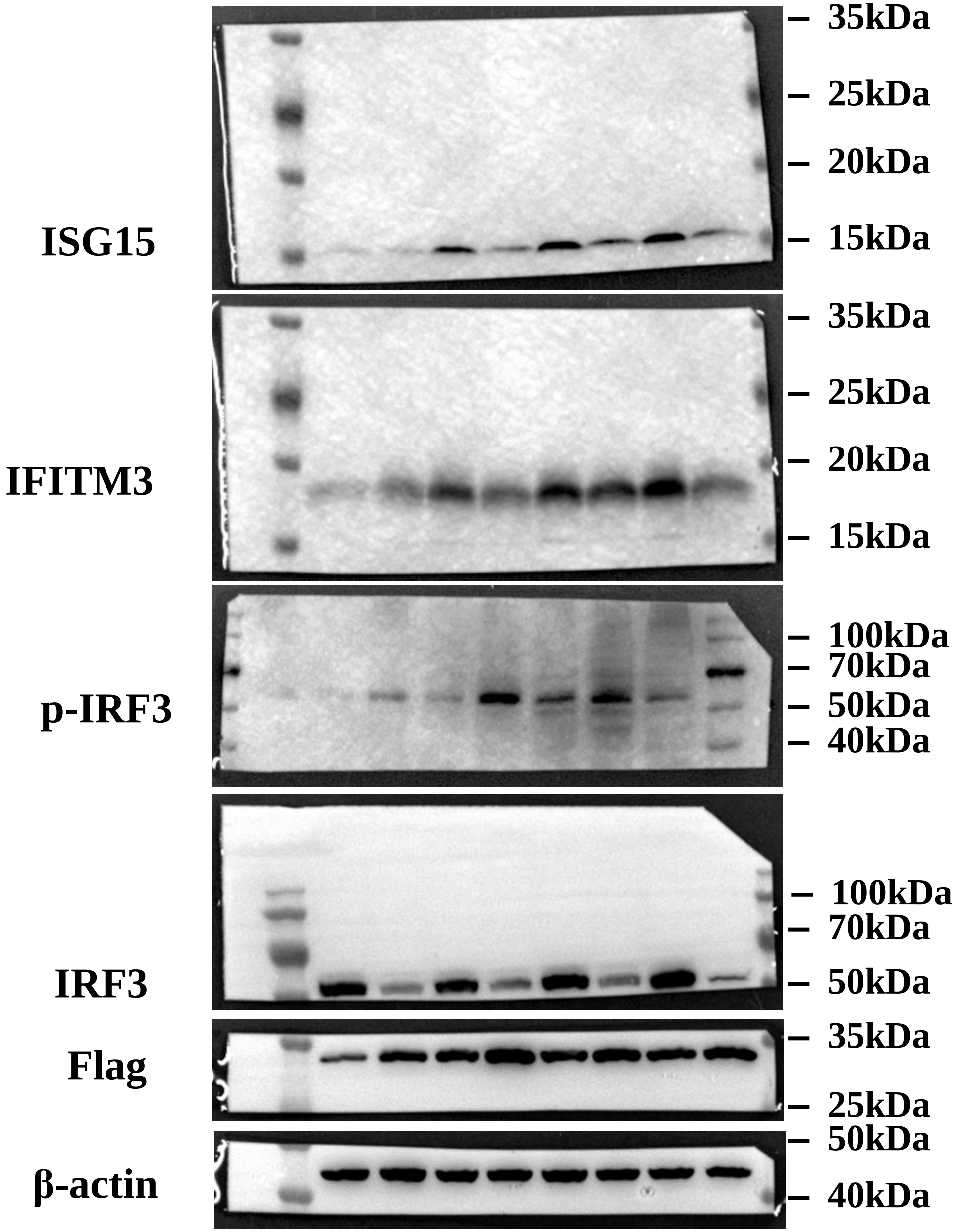

ISG15
IFITM3
p-IRF3
IRF3
Flag
β-actin
35kDa
25kDa
20kDa
15kDa
35kDa
25kDa
20kDa
15kDa
100kDa
70kDa
50kDa
40kDa
100kDa
70kDa
50kDa
35kDa
25kDa
50kDa
40kDa

Figure 2

E

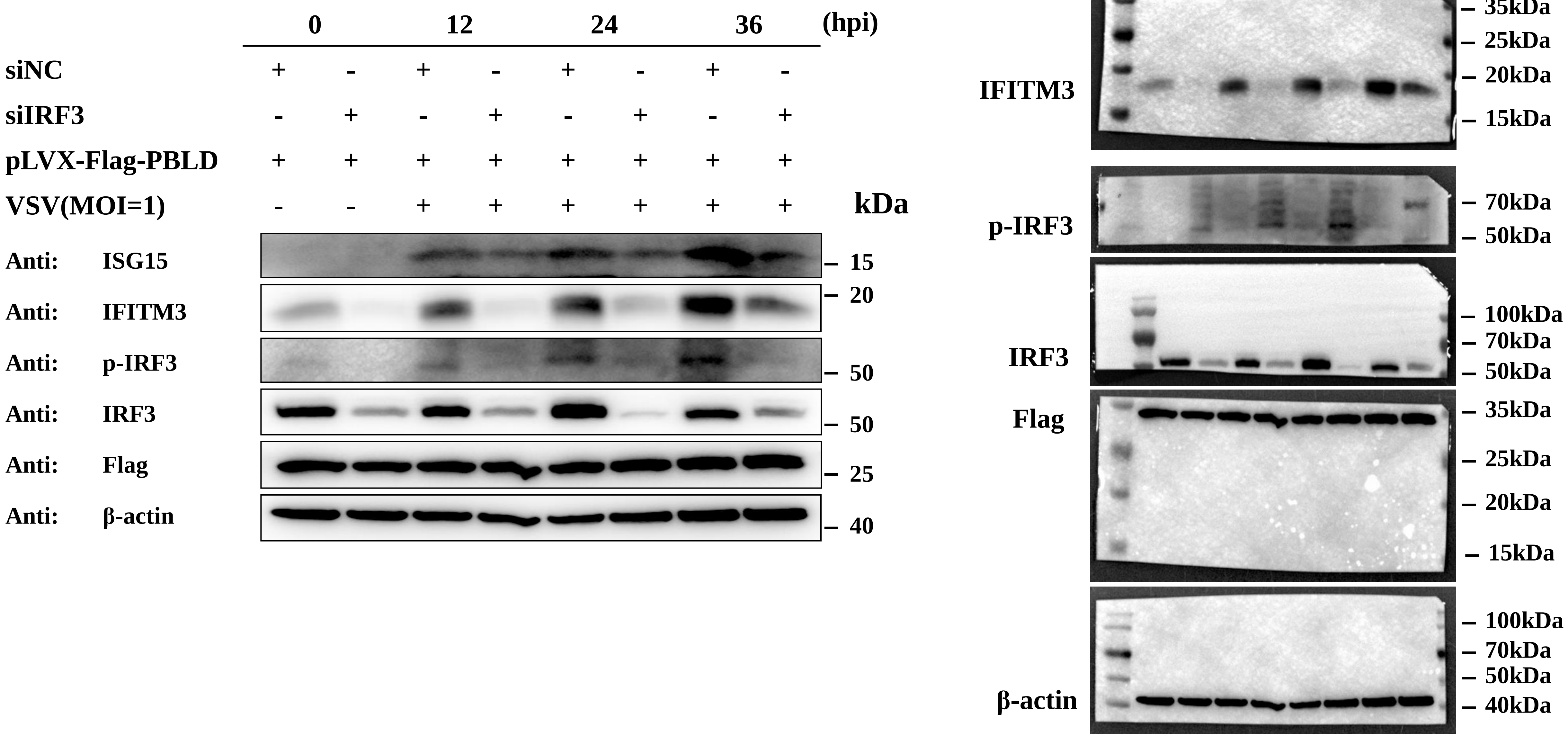

0
12
24
36
(hpi)
siNC
siIRF3
pLVX-Flag-PBLD
VSV(MOI=1)
kDa
Anti: ISG15
Anti: IFITM3
Anti: p-IRF3
Anti: IRF3
Anti: Flag
Anti: β-actin
15
20
50
50
25
40
ISG15
35kDa
25kDa
20kDa
15kDa
IFITM3
35kDa
25kDa
20kDa
15kDa
p-IRF3
70kDa
50kDa
IRF3
100kDa
70kDa
50kDa
Flag
35kDa
25kDa
20kDa
15kDa
β-actin
100kDa
70kDa
50kDa
40kDa

Figure 2

F

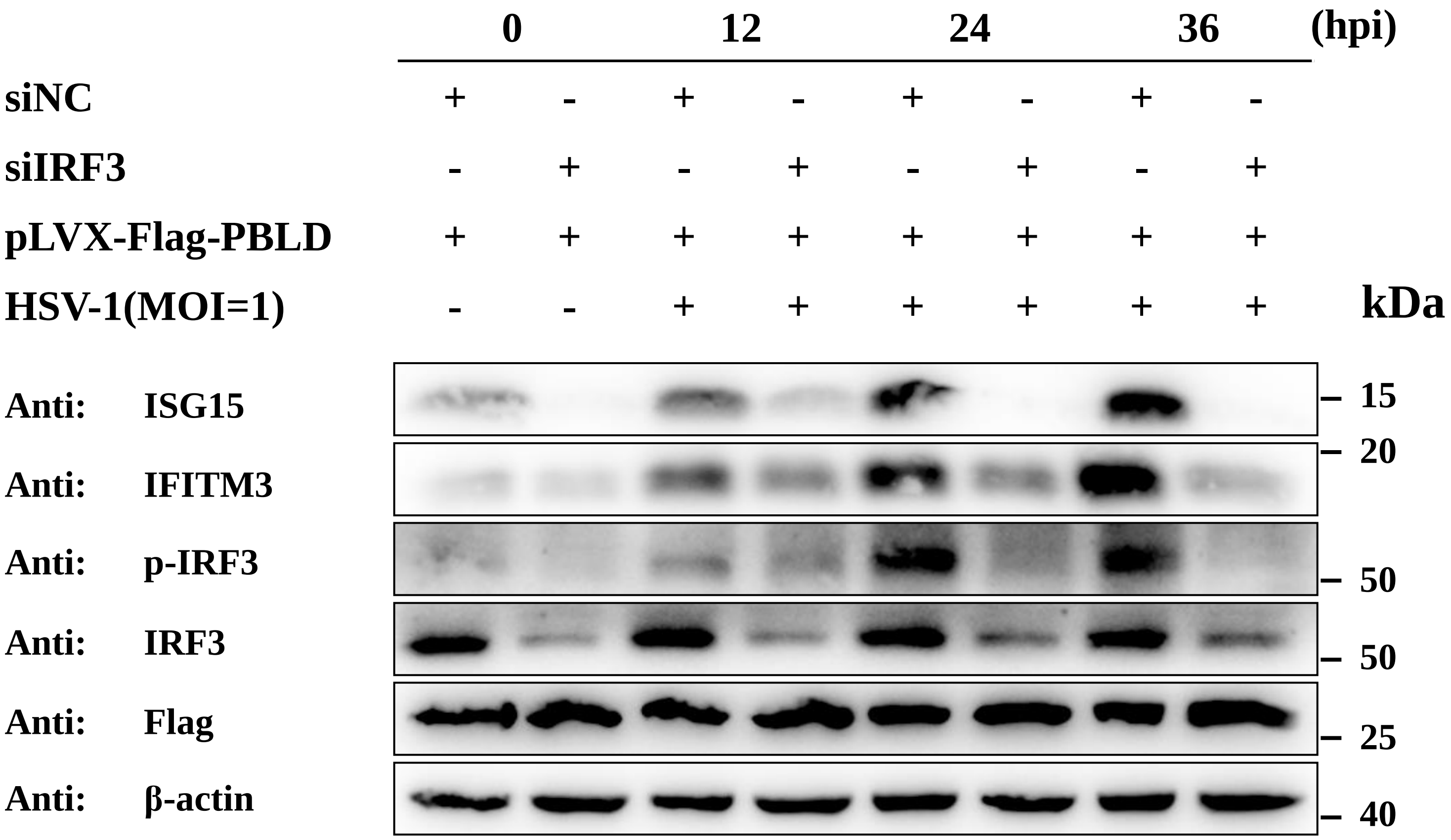

0
12
24
36
(hpi)
siNC + - + - + - + -
siIRF3 - + - + - + - +
pLVX-Flag-PBLD + + + + + + + +
HSV-1(MOI=1) - - + + + + + +
kDa
Anti: ISG15
Anti: IFITM3
Anti: p-IRF3
Anti: IRF3
Anti: Flag
Anti: β-actin
15
20
50
50
25
40

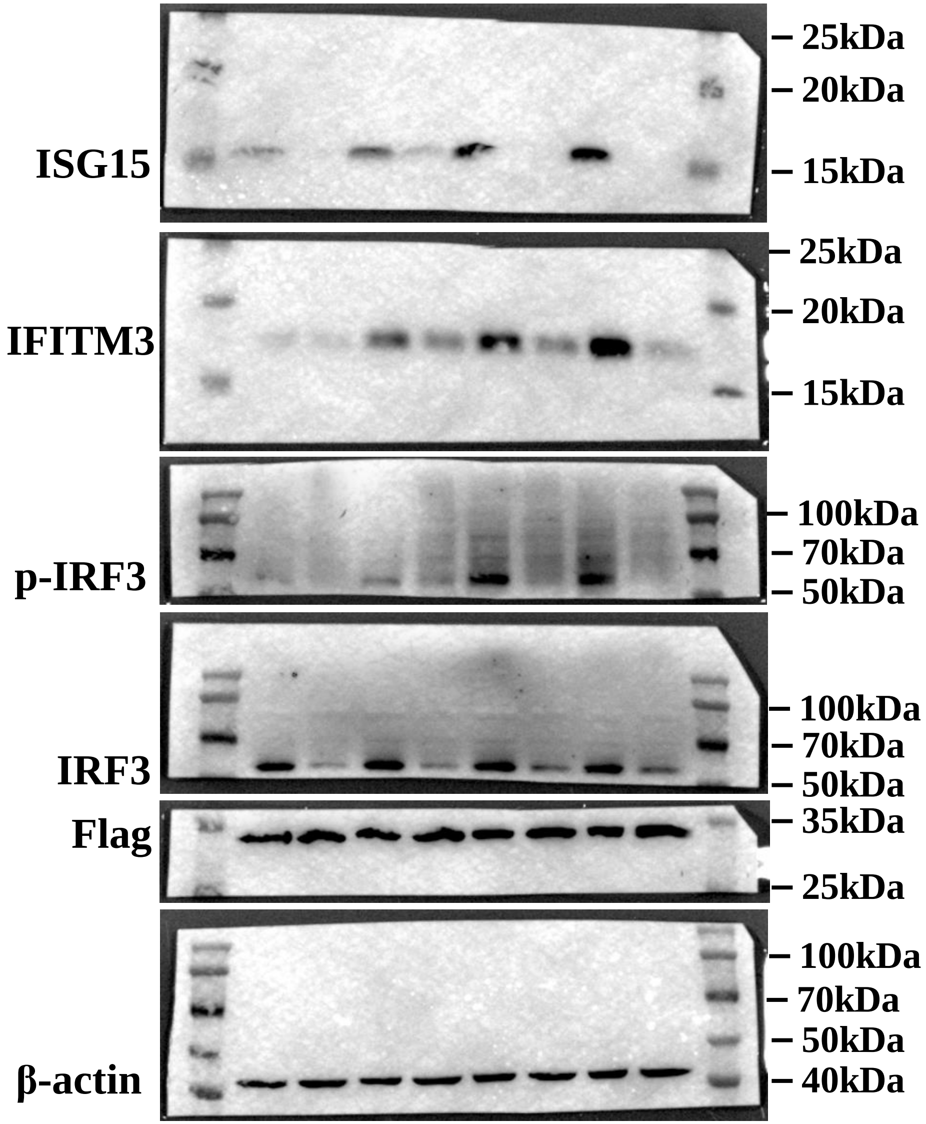

ISG15
IFITM3
p-IRF3
IRF3
Flag
β-actin
25kDa
20kDa
15kDa
25kDa
20kDa
15kDa
100kDa
70kDa
50kDa
100kDa
70kDa
50kDa
35kDa
25kDa
100kDa
70kDa
50kDa
40kDa

Figure 2

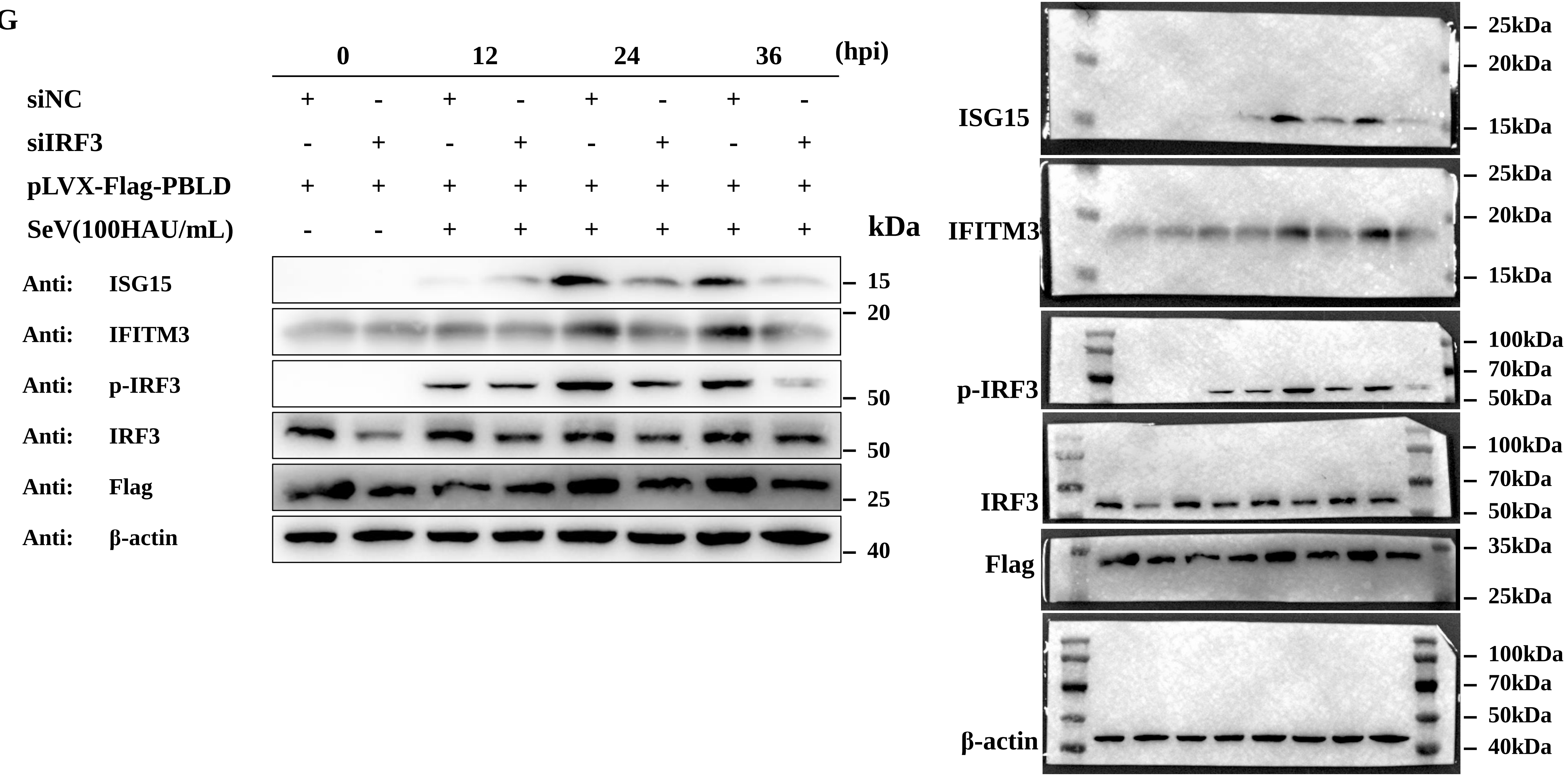
G
0
12
24
36
(hpi)
siNC + - + - + - + -
siIRF3 - + - + - + - +
pLVX-Flag-PBLD + + + + + + + +
SeV(100HAU/mL) - - + + + + + +
kDa
Anti: ISG15
Anti: IFITM3
Anti: p-IRF3
Anti: IRF3
Anti: Flag
Anti: β-actin
15
20
50
50
25
40
ISG15
IFITM3
p-IRF3
IRF3
Flag
β-actin
25kDa
20kDa
15kDa
25kDa
20kDa
15kDa
100kDa
70kDa
50kDa
100kDa
70kDa
50kDa
35kDa
25kDa
100kDa
70kDa
50kDa
40kDa

FIG3

Figure 3

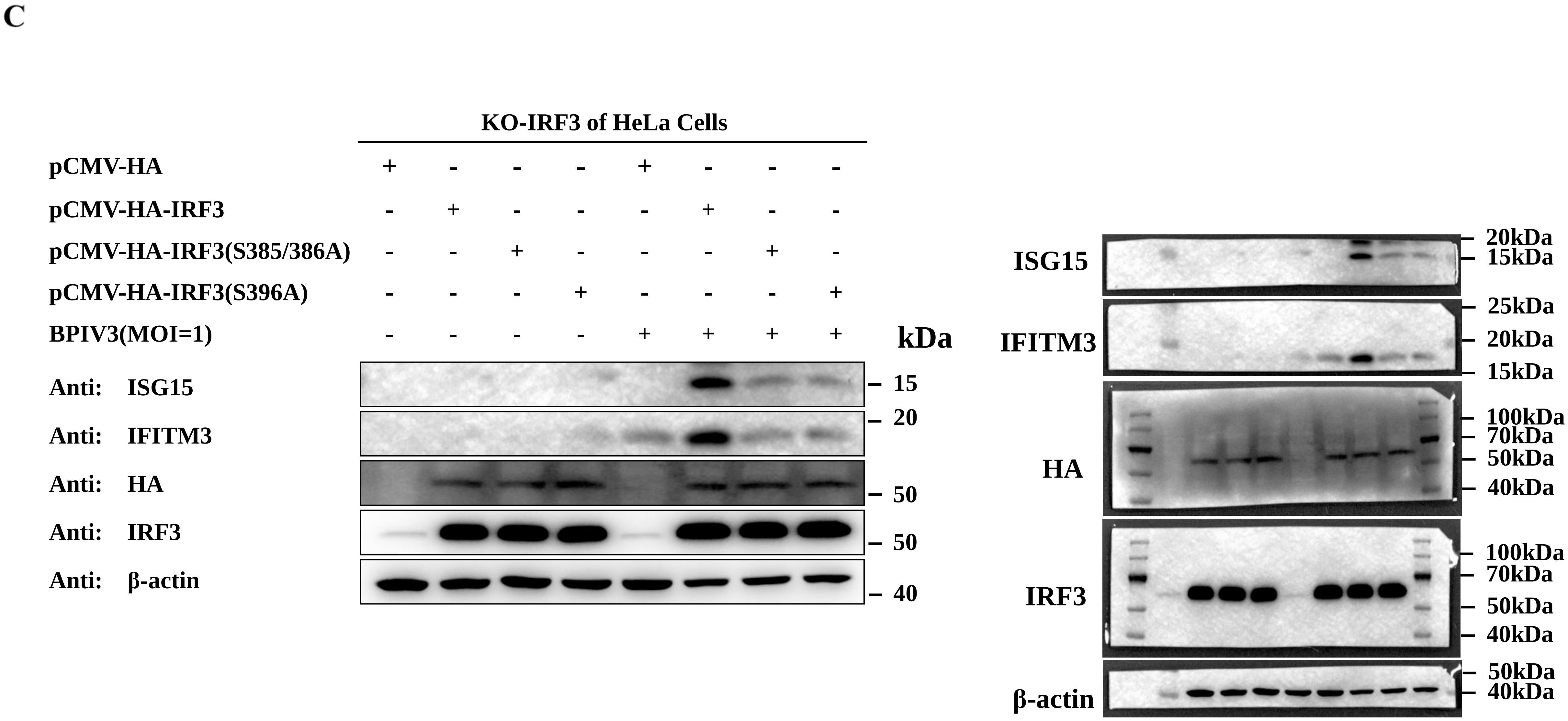
C
KO-IRF3 of HeLa Cells
pCMV-HA + - - - + - - -
pCMV-HA-IRF3 - + - - - + - -
pCMV-HA-IRF3(S385/386A) - - + - - - + -
pCMV-HA-IRF3(S396A) - - - + - - - +
BPIV3(MOI=1) - - - - + + + +
kDa
Anti: ISG15
Anti: IFITM3
Anti: HA
Anti: IRF3
Anti: β-actin
15
20
50
50
40
ISG15
IFITM3
HA
IRF3
β-actin
20kDa
15kDa
25kDa
20kDa
15kDa
100kDa
70kDa
50kDa
40kDa
100kDa
70kDa
50kDa
40kDa
50kDa
40kDa

Figure 3

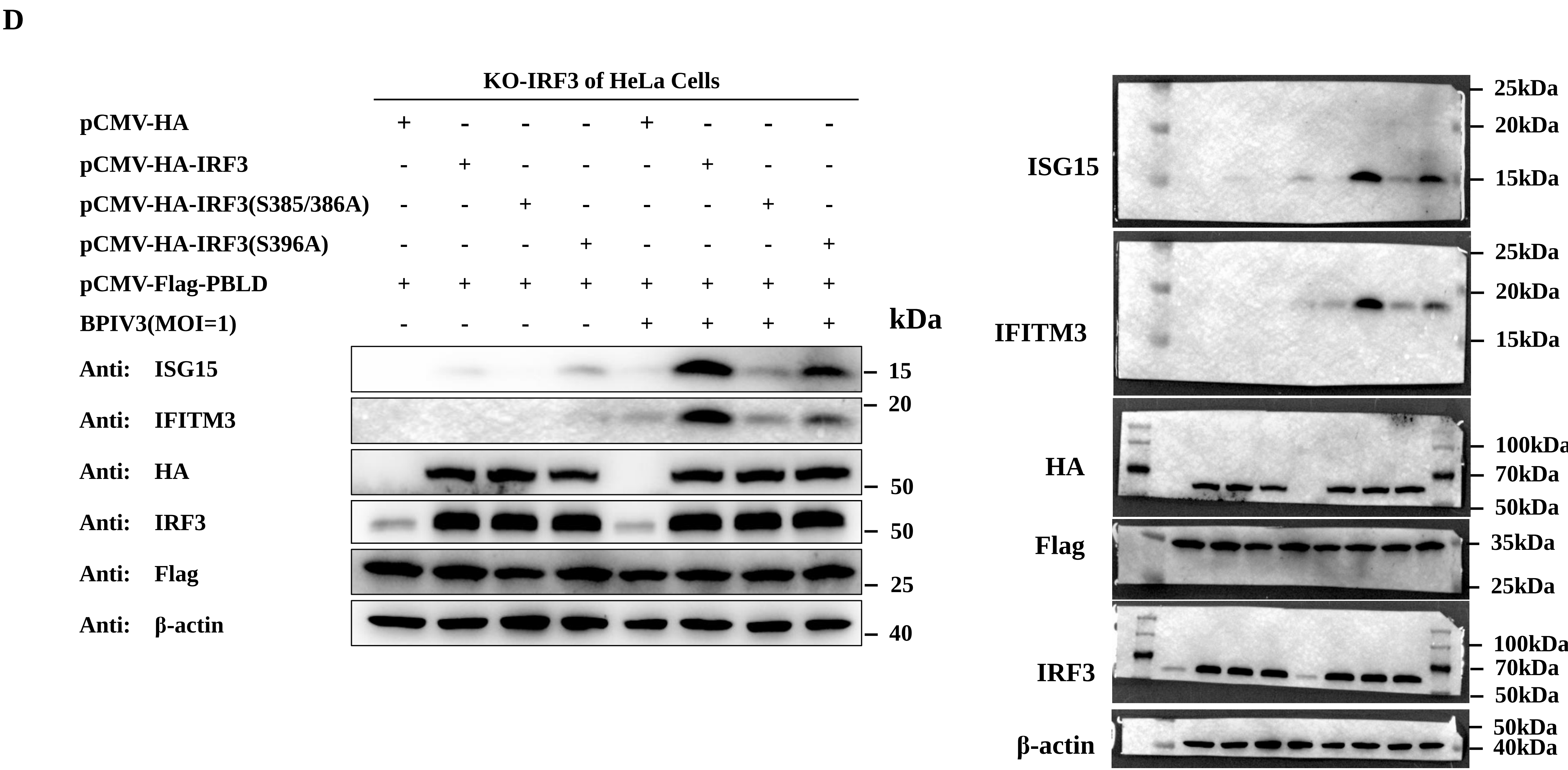
D
KO-IRF3 of HeLa Cells
pCMV-HA + - - - + - - -
pCMV-HA-IRF3 - + - - - + - -
pCMV-HA-IRF3(S385/386A) - - + - - - + -
pCMV-HA-IRF3(S396A) - - - + - - - +
pCMV-Flag-PBLD + + + + + + + +
BPIV3(MOI=1) - - - - + + + +
kDa
Anti: ISG15
15
Anti: IFITM3
20
Anti: HA
50
Anti: IRF3
50
Anti: Flag
25
Anti: β-actin
40
ISG15
25kDa
20kDa
15kDa
IFITM3
25kDa
20kDa
15kDa
HA
100kDa
70kDa
50kDa
Flag
35kDa
25kDa
IRF3
100kDa
70kDa
50kDa
β-actin
50kDa
40kDa

Figure 3

E

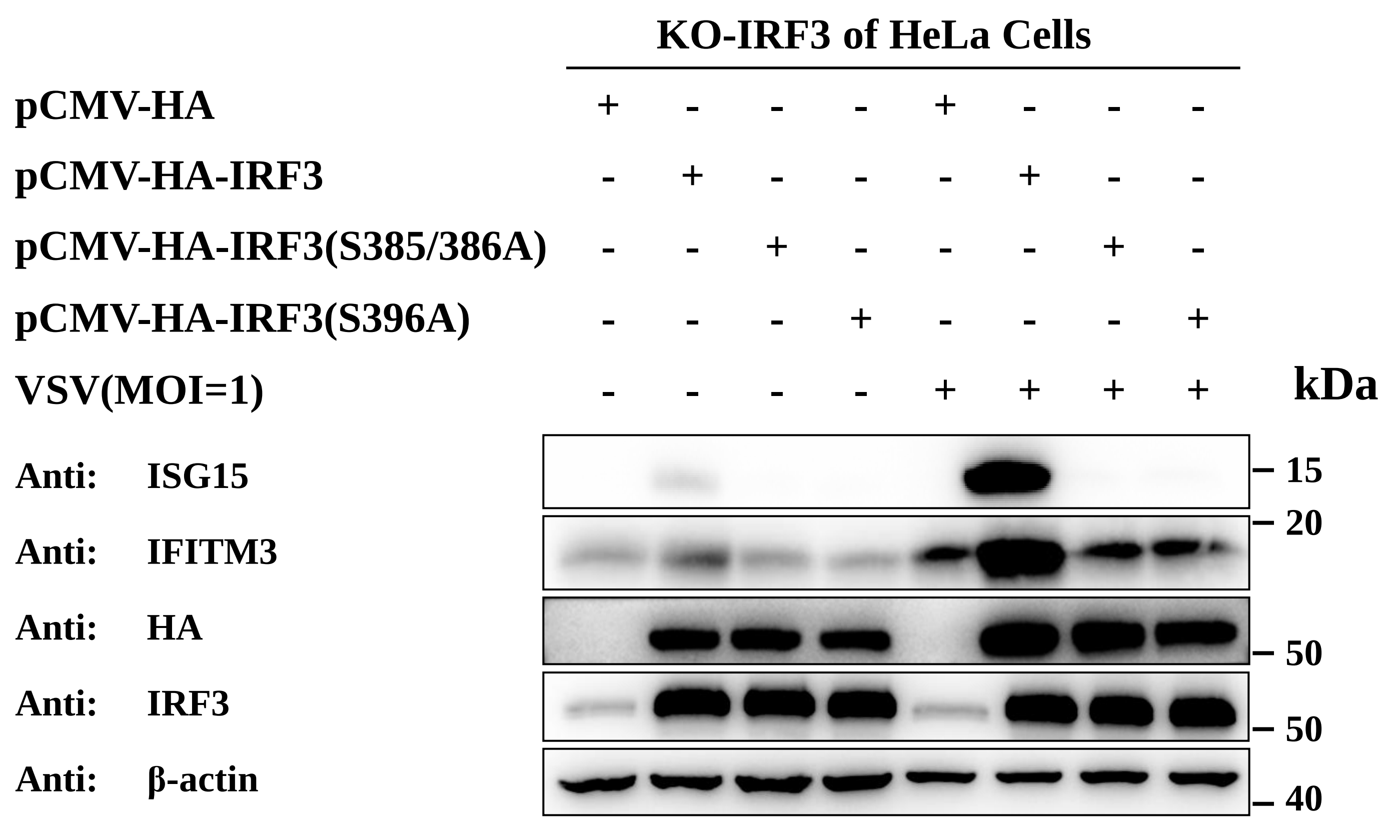

KO-IRF3 of HeLa Cells
pCMV-HA + - - - + - - -
pCMV-HA-IRF3 - + - - - + - -
pCMV-HA-IRF3(S385/386A) - - + - - - + -
pCMV-HA-IRF3(S396A) - - - + - - - +
VSV(MOI=1) - - - - + + + +
kDa
Anti: ISG15
Anti: IFITM3
Anti: HA
Anti: IRF3
Anti: β-actin
15
20
50
50
40

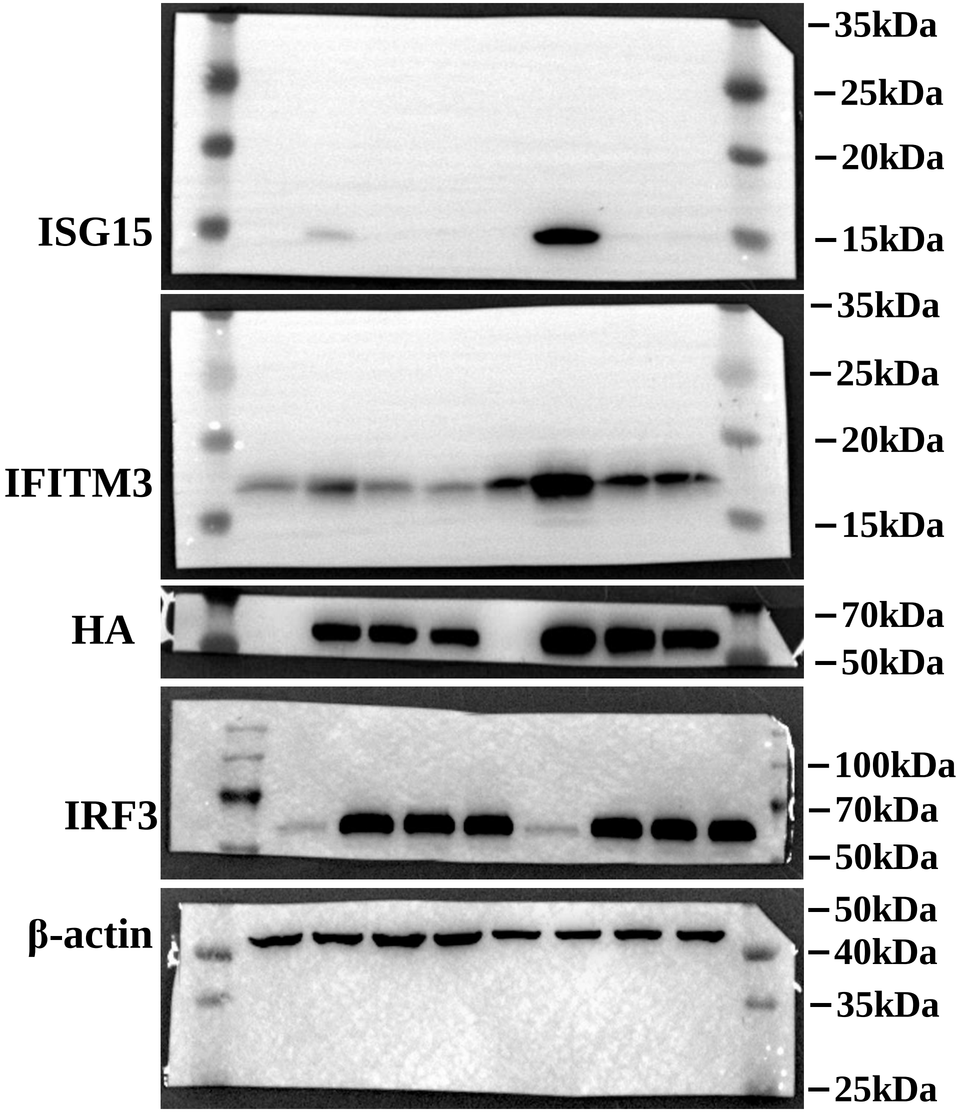

ISG15
IFITM3
HA
IRF3
β-actin
35kDa
25kDa
20kDa
15kDa
35kDa
25kDa
20kDa
15kDa
70kDa
50kDa
100kDa
70kDa
50kDa
50kDa
40kDa
35kDa
25kDa

Figure 3

F

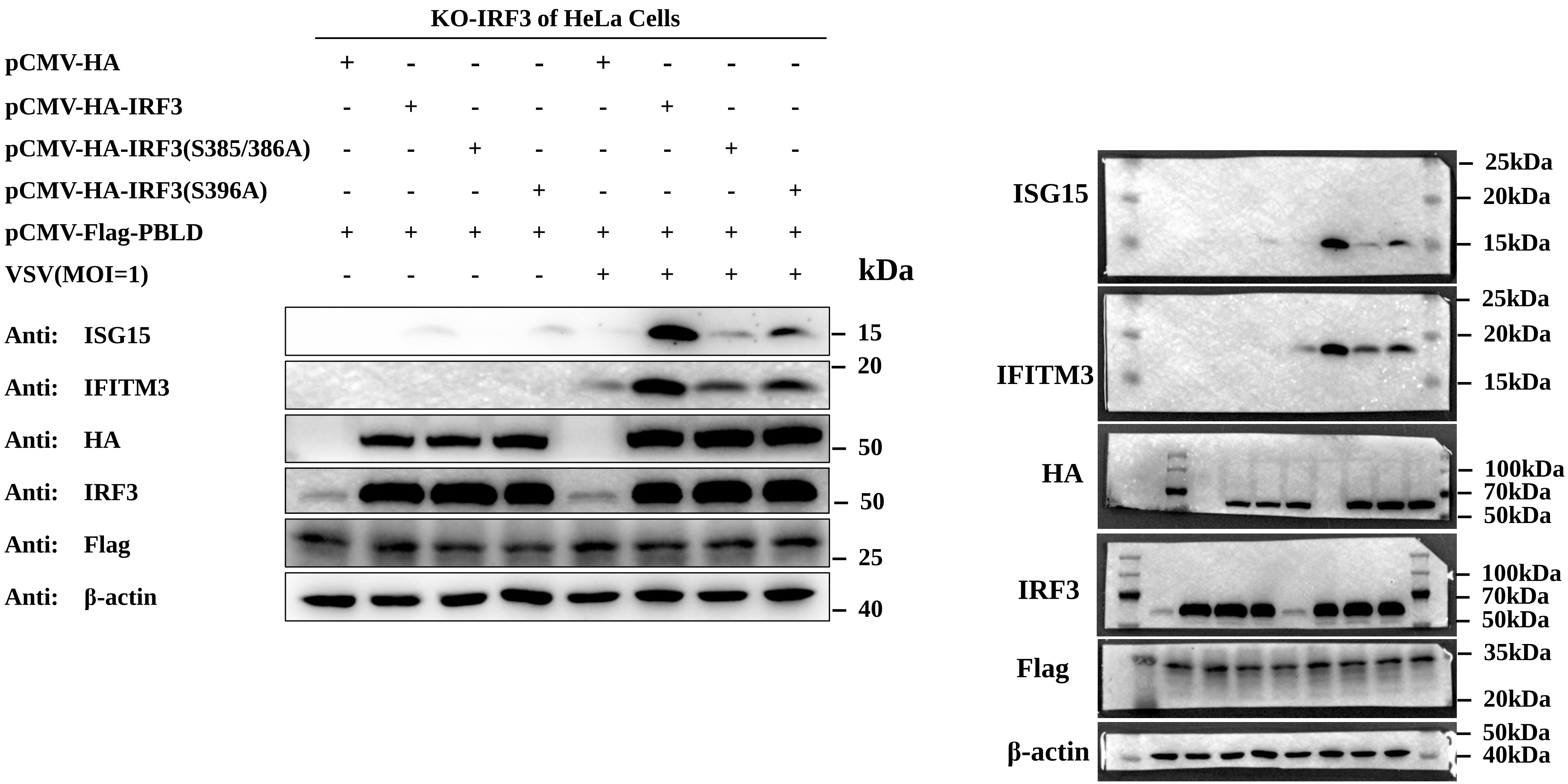
KO-IRF3 of HeLa Cells
pCMV-HA + - - - + - - -
pCMV-HA-IRF3 - + - - - + - -
pCMV-HA-IRF3(S385/386A) - - + - - - + -
pCMV-HA-IRF3(S396A) - - - + - - - +
pCMV-Flag-PBLD + + + + + + + +
VSV(MOI=1) - - - - + + + +
kDa
Anti: ISG15
Anti: IFITM3
Anti: HA
Anti: IRF3
Anti: Flag
Anti: β-actin
15
20
50
50
25
40
ISG15
IFITM3
HA
IRF3
Flag
β-actin
25kDa
20kDa
15kDa
25kDa
20kDa
15kDa
100kDa
70kDa
50kDa
100kDa
70kDa
50kDa
35kDa
20kDa
50kDa
40kDa

Figure 3

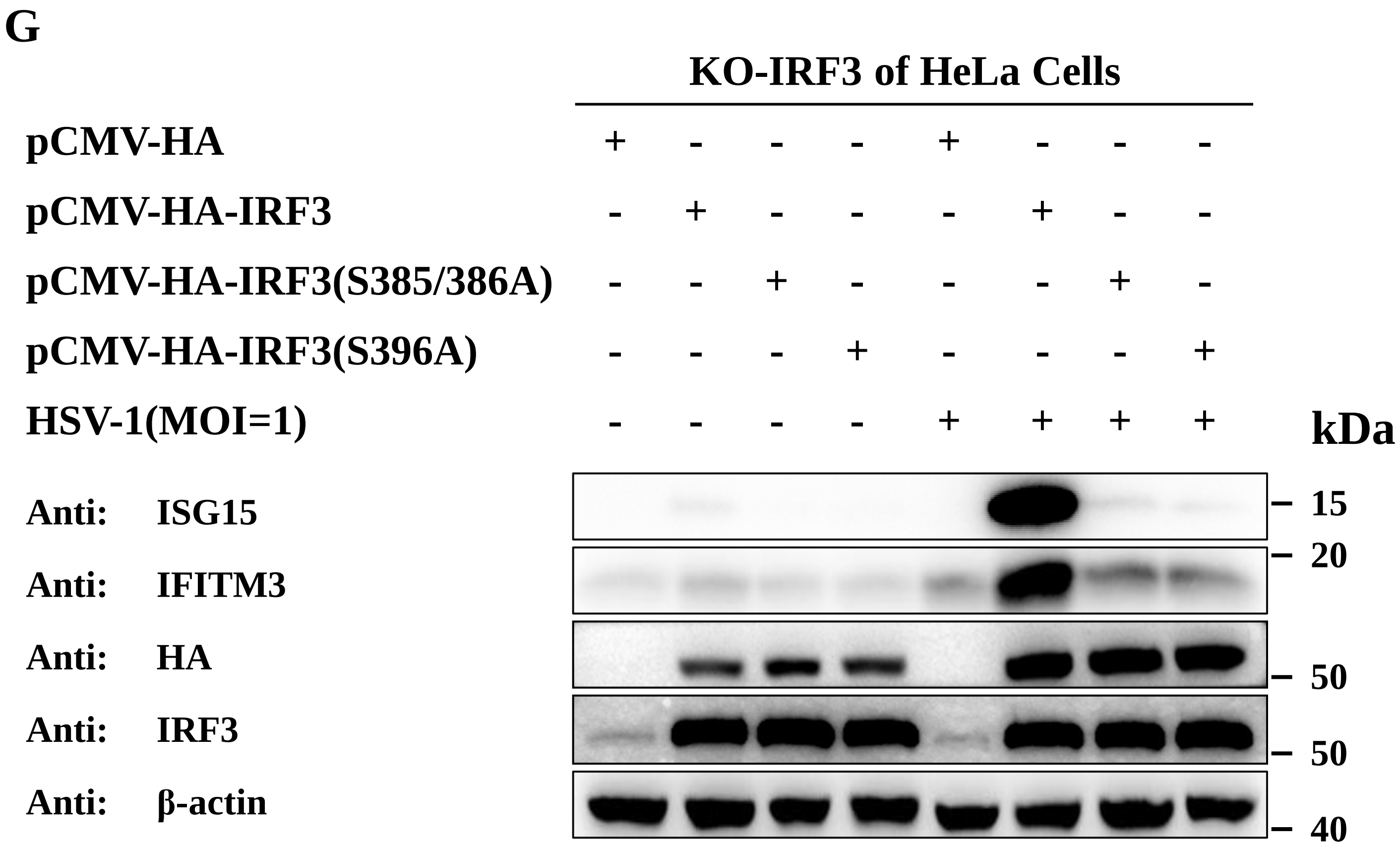
G
KO-IRF3 of HeLa Cells
pCMV-HA + - - - + - - -
pCMV-HA-IRF3 - + - - - + - -
pCMV-HA-IRF3(S385/386A) - - + - - - + -
pCMV-HA-IRF3(S396A) - - - + - - - +
HSV-1(MOI=1) - - - - + + + +
kDa
Anti: ISG15
Anti: IFITM3
Anti: HA
Anti: IRF3
Anti: β-actin
15
20
50
50
40

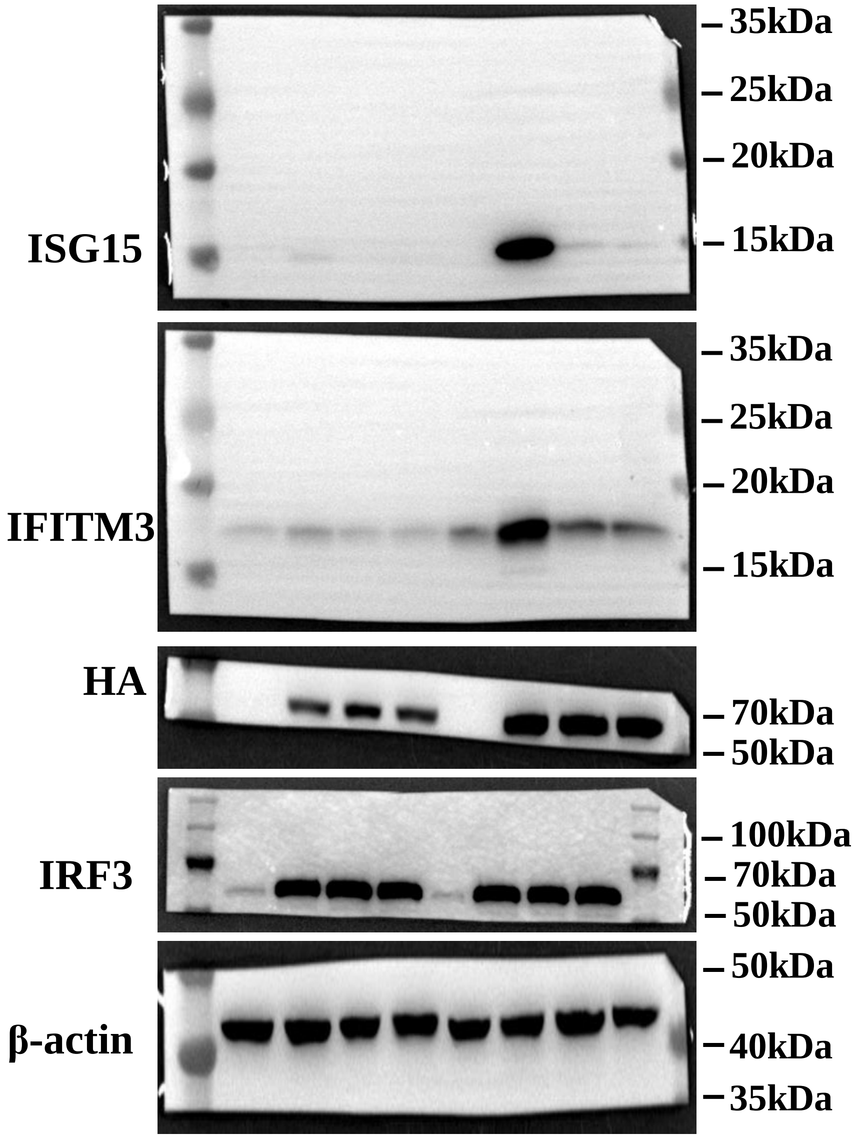
ISG15
35kDa
25kDa
20kDa
15kDa
IFITM3
35kDa
25kDa
20kDa
15kDa
HA
70kDa
50kDa
IRF3
100kDa
70kDa
50kDa
β-actin
50kDa
40kDa
35kDa

Figure 3

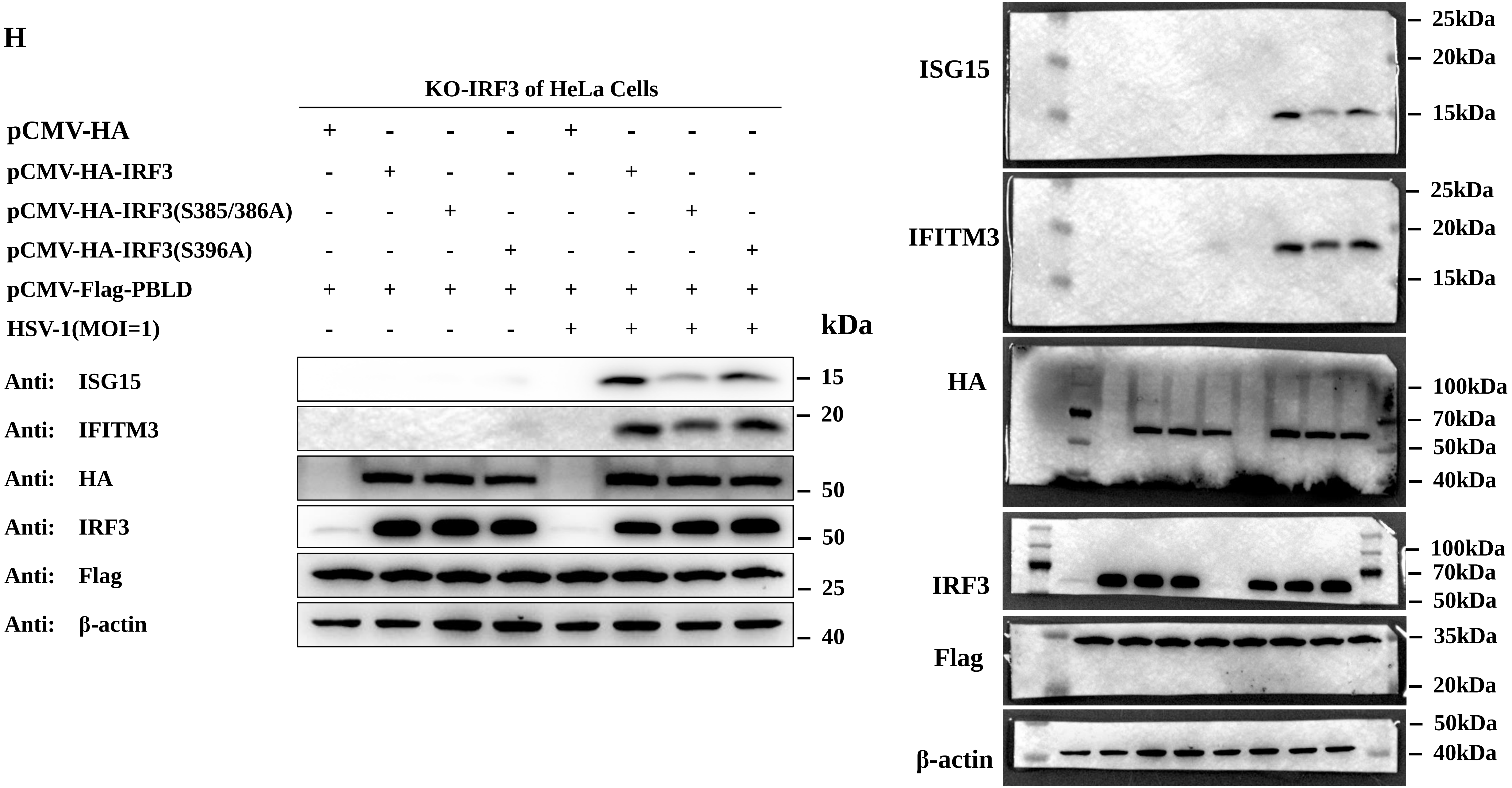
H
KO-IRF3 of HeLa Cells
pCMV-HA + - - - + - - -
pCMV-HA-IRF3 - + - - - + - -
pCMV-HA-IRF3(S385/386A) - - + - - - + -
pCMV-HA-IRF3(S396A) - - - + - - - +
pCMV-Flag-PBLD + + + + + + + +
HSV-1(MOI=1) - - - - + + + +
kDa
Anti: ISG15
Anti: IFITM3
Anti: HA
Anti: IRF3
Anti: Flag
Anti: β-actin
15
20
50
50
25
40
ISG15
IFITM3
HA
IRF3
Flag
β-actin
25kDa
20kDa
15kDa
25kDa
20kDa
15kDa
100kDa
70kDa
50kDa
40kDa
100kDa
70kDa
50kDa
35kDa
20kDa
50kDa
40kDa

FIG4

Figure 4

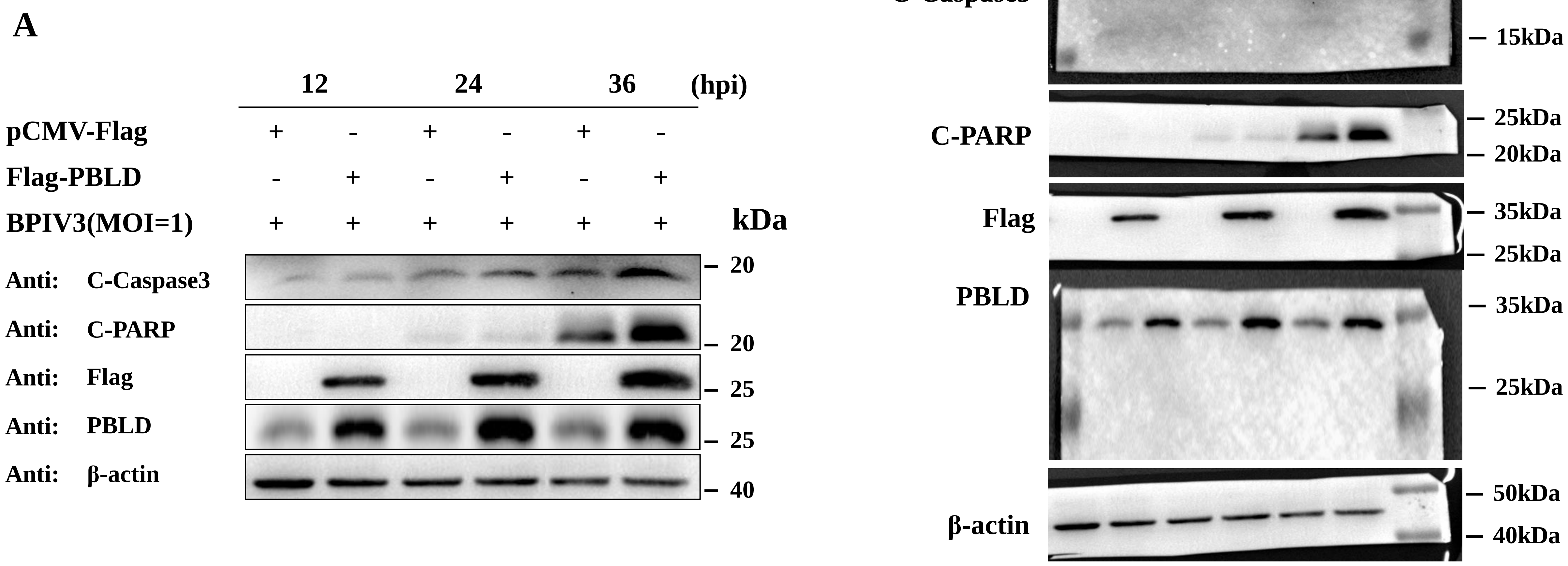
A
12
24
36
(hpi)
pCMV-Flag + - + - + -
Flag-PBLD - + - + - +
BPIV3(MOI=1) + + + + + +
kDa
Anti: C-Caspase3
Anti: C-PARP
Anti: Flag
Anti: PBLD
Anti: β-actin
20
20
25
25
40
C-Caspase3
C-PARP
Flag
PBLD
β-actin
40kDa
35kDa
20kDa
15kDa
25kDa
20kDa
35kDa
25kDa
35kDa
25kDa
50kDa
40kDa

Figure 4

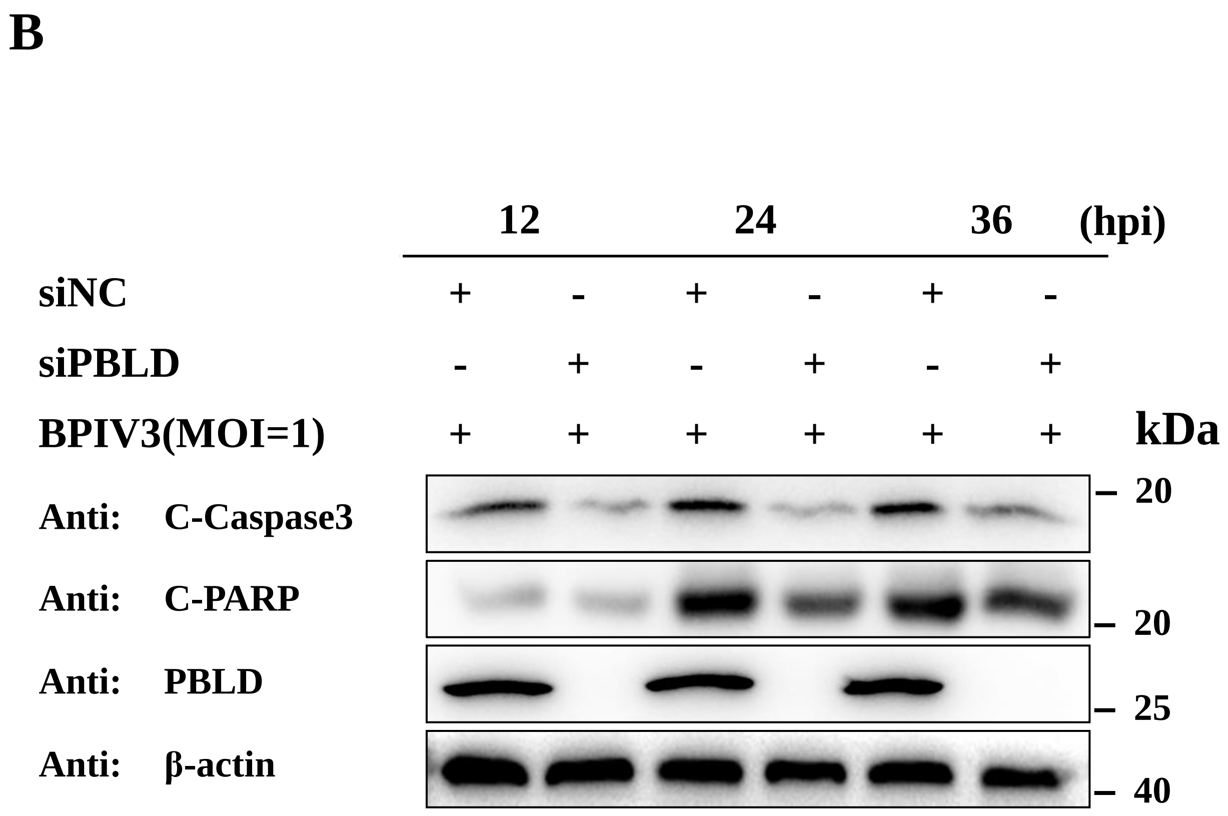
B
12
24
36
(hpi)
siNC + - + - + -
siPBLD - + - + - +
BPIV3(MOI=1) + + + + + +
kDa
Anti: C-Caspase3
Anti: C-PARP
Anti: PBLD
Anti: β-actin
20
20
25
40

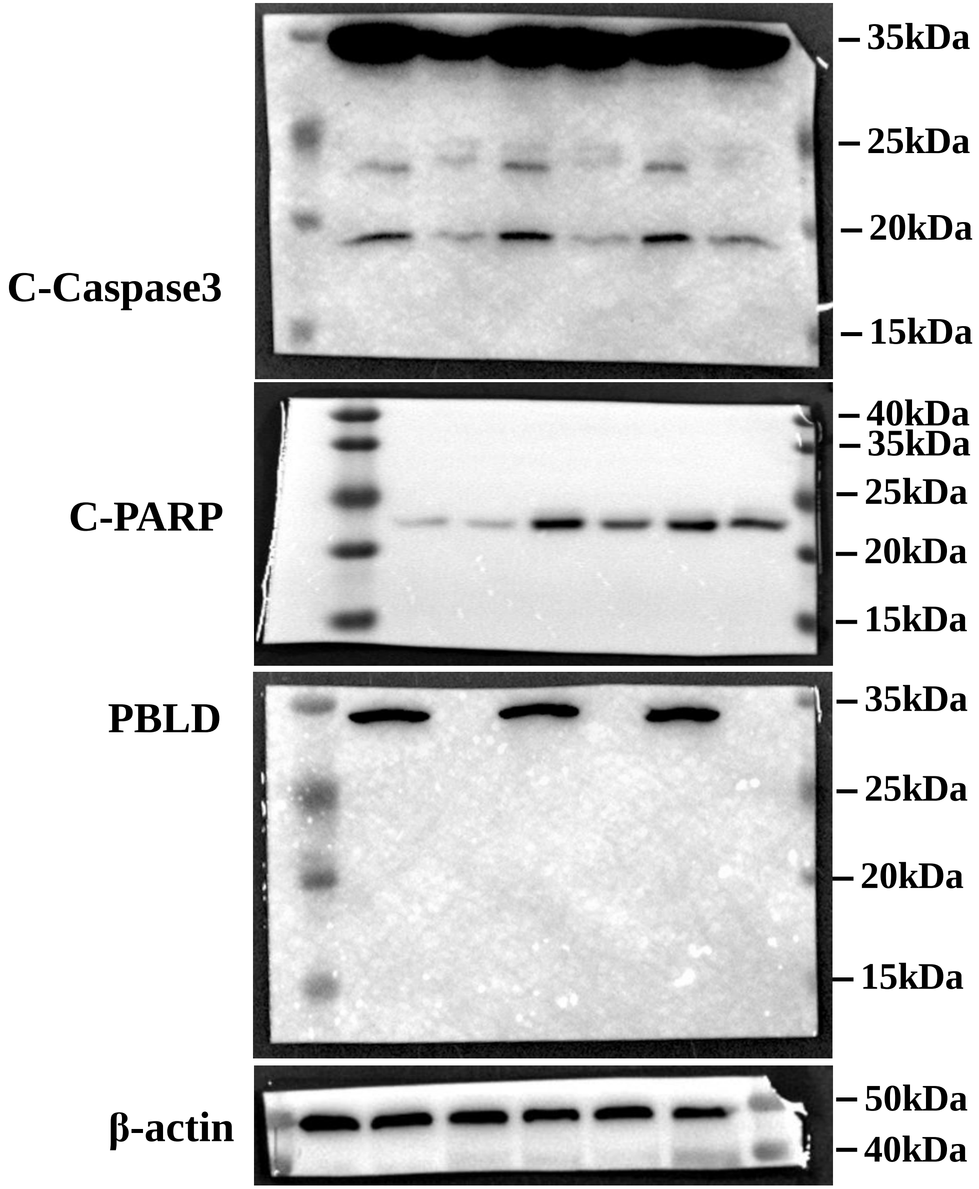
C-Caspase3
C-PARP
PBLD
β-actin
35kDa
25kDa
20kDa
15kDa
40kDa
35kDa
25kDa
20kDa
15kDa
35kDa
25kDa
20kDa
15kDa
50kDa
40kDa

Figure 4

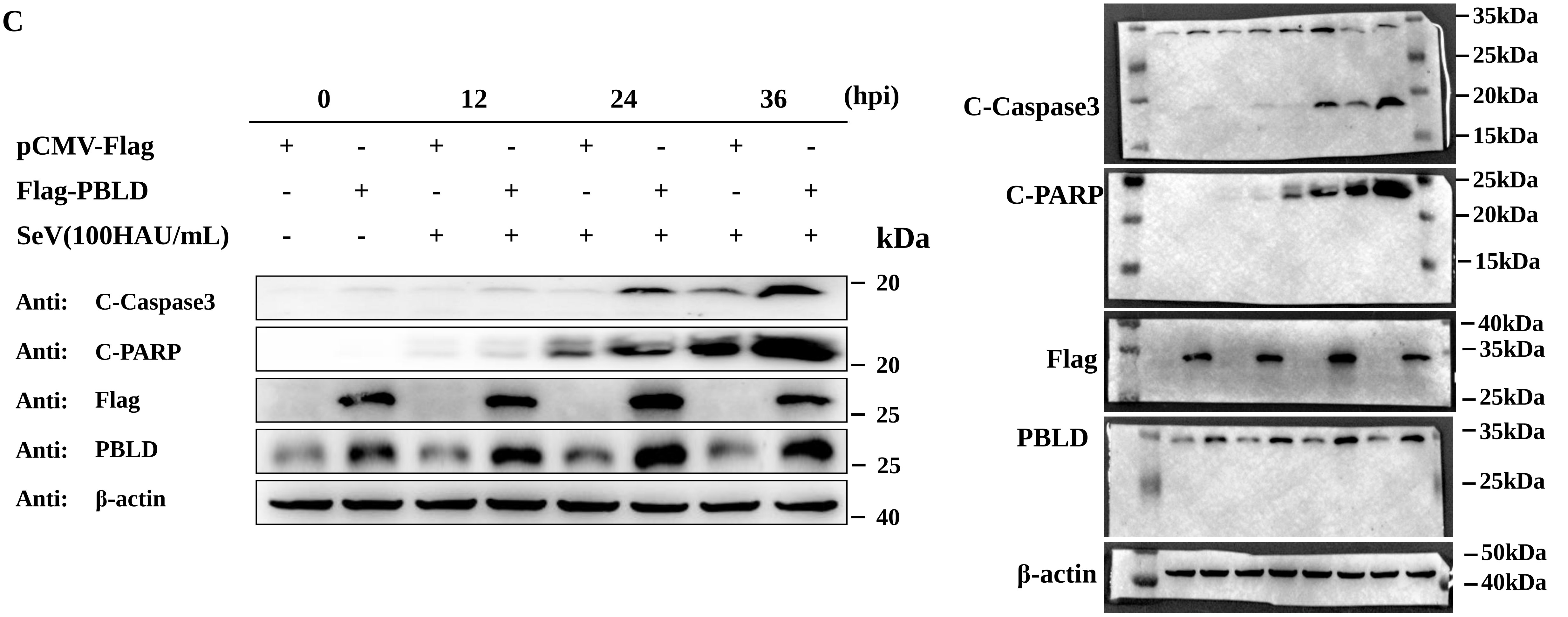
C
0
12
24
36
(hpi)
pCMV-Flag + - + - + - + -
Flag-PBLD - + - + - + - +
SeV(100HAU/mL) - - + + + + + +
kDa
Anti: C-Caspase3
Anti: C-PARP
Anti: Flag
Anti: PBLD
Anti: β-actin
20
20
25
25
40
C-Caspase3
35kDa
25kDa
20kDa
15kDa
C-PARP
25kDa
20kDa
15kDa
Flag
40kDa
35kDa
25kDa
PBLD
35kDa
25kDa
β-actin
50kDa
40kDa

Figure 4

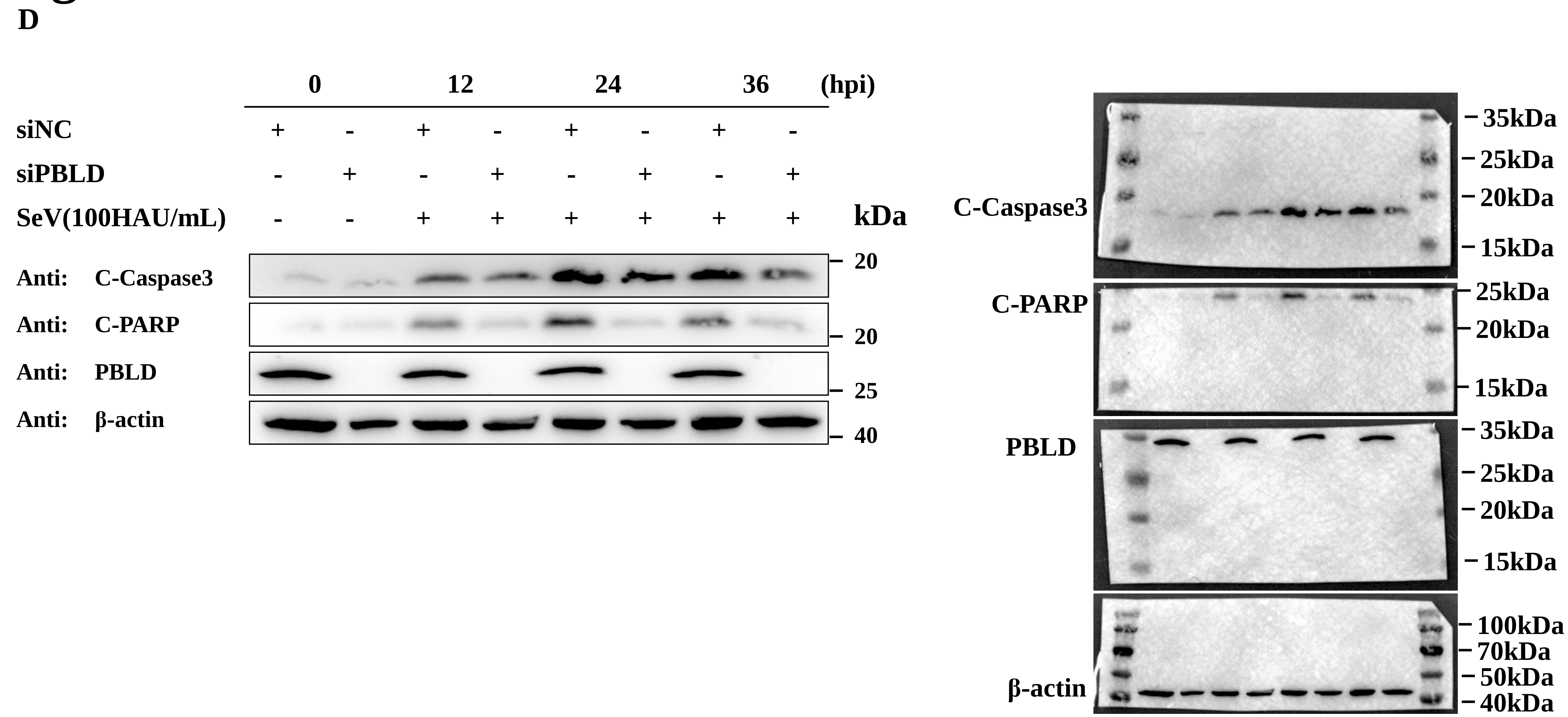

D
0
12
24
36
(hpi)
siNC
siPBLD
SeV(100HAU/mL)
kDa
Anti: C-Caspase3
Anti: C-PARP
Anti: PBLD
Anti: β-actin
20
20
25
40
C-Caspase3
C-PARP
PBLD
β-actin
35kDa
25kDa
20kDa
15kDa
25kDa
20kDa
15kDa
35kDa
25kDa
20kDa
15kDa
100kDa
70kDa
50kDa
40kDa

FIG5

Figure 5

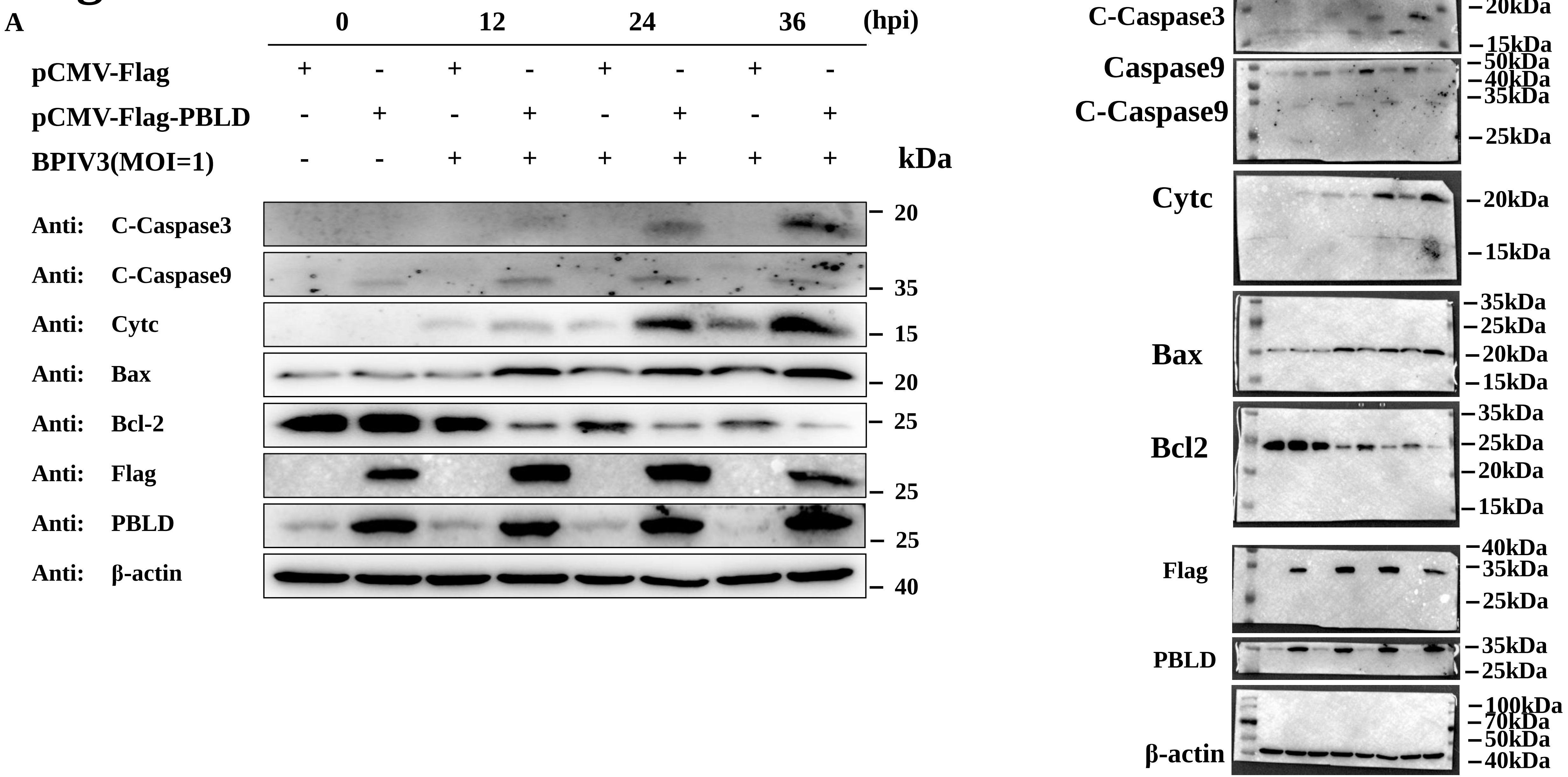
A
0
12
24
36
(hpi)
pCMV-Flag + - + - + - + -
pCMV-Flag-PBLD - + - + - + - +
BPIV3(MOI=1) - - + + + + + +
kDa
Anti: C-Caspase3
Anti: C-Caspase9
Anti: Cytc
Anti: Bax
Anti: Bcl-2
Anti: Flag
Anti: PBLD
Anti: β-actin
20
35
15
20
25
25
25
40
C-Caspase3
35kDa
20kDa
15kDa
Caspase9
C-Caspase9
50kDa
40kDa
35kDa
25kDa
Cytc
20kDa
15kDa
Bax
35kDa
25kDa
20kDa
15kDa
Bcl2
35kDa
25kDa
20kDa
15kDa
Flag
40kDa
35kDa
25kDa
PBLD
35kDa
25kDa
β-actin
100kDa
70kDa
50kDa
40kDa

Figure 5

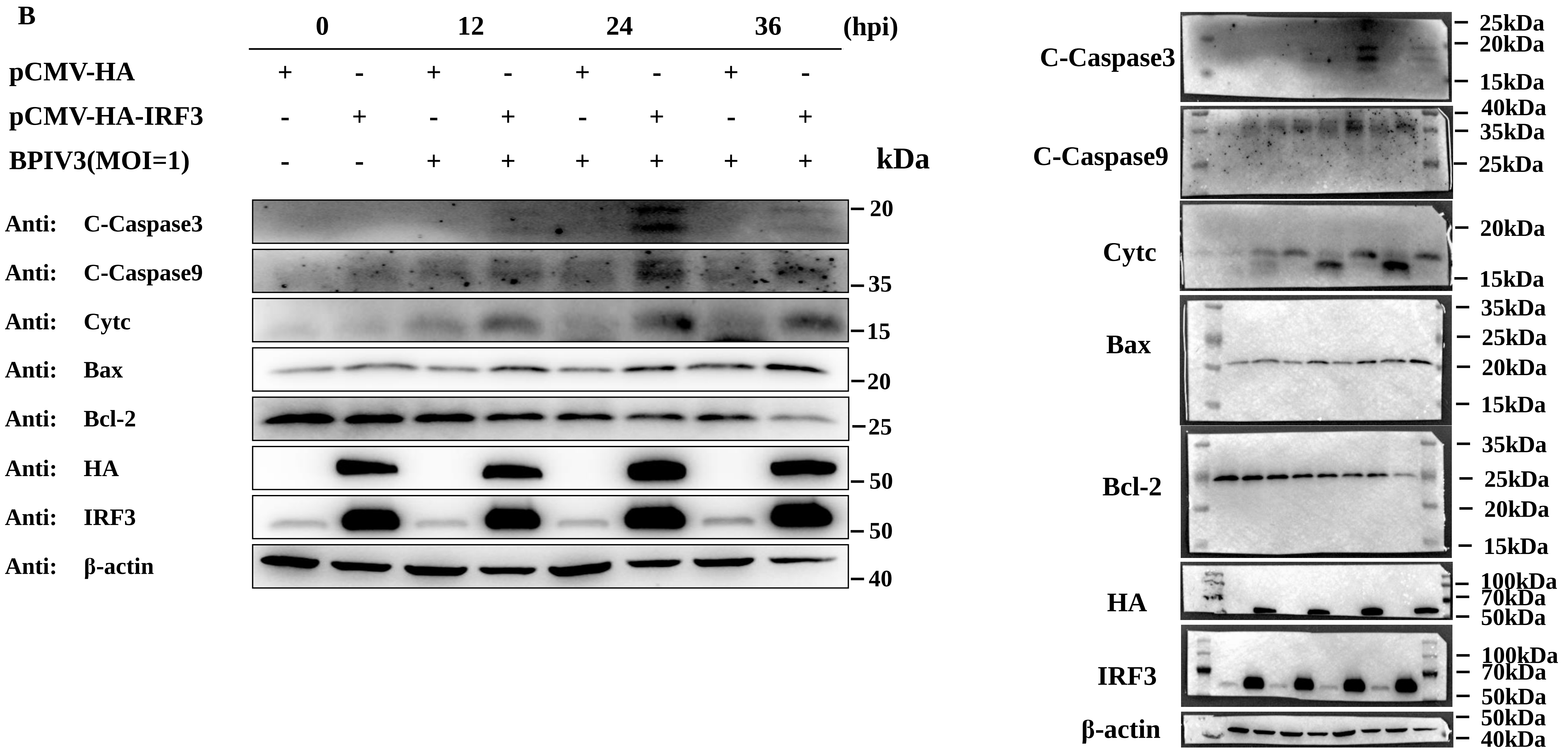
B
0
12
24
36
(hpi)
pCMV-HA + - + - + - + -
pCMV-HA-IRF3 - + - + - + - +
BPIV3(MOI=1) - - + + + + + +
kDa
Anti: C-Caspase3
Anti: C-Caspase9
Anti: Cytc
Anti: Bax
Anti: Bcl-2
Anti: HA
Anti: IRF3
Anti: β-actin
20
35
15
20
25
50
50
40
C-Caspase3
25kDa
20kDa
15kDa
C-Caspase9
40kDa
35kDa
25kDa
Cytc
20kDa
15kDa
Bax
35kDa
25kDa
20kDa
15kDa
Bcl-2
35kDa
25kDa
20kDa
15kDa
HA
100kDa
70kDa
50kDa
IRF3
100kDa
70kDa
50kDa
β-actin
50kDa
40kDa

Figure 5

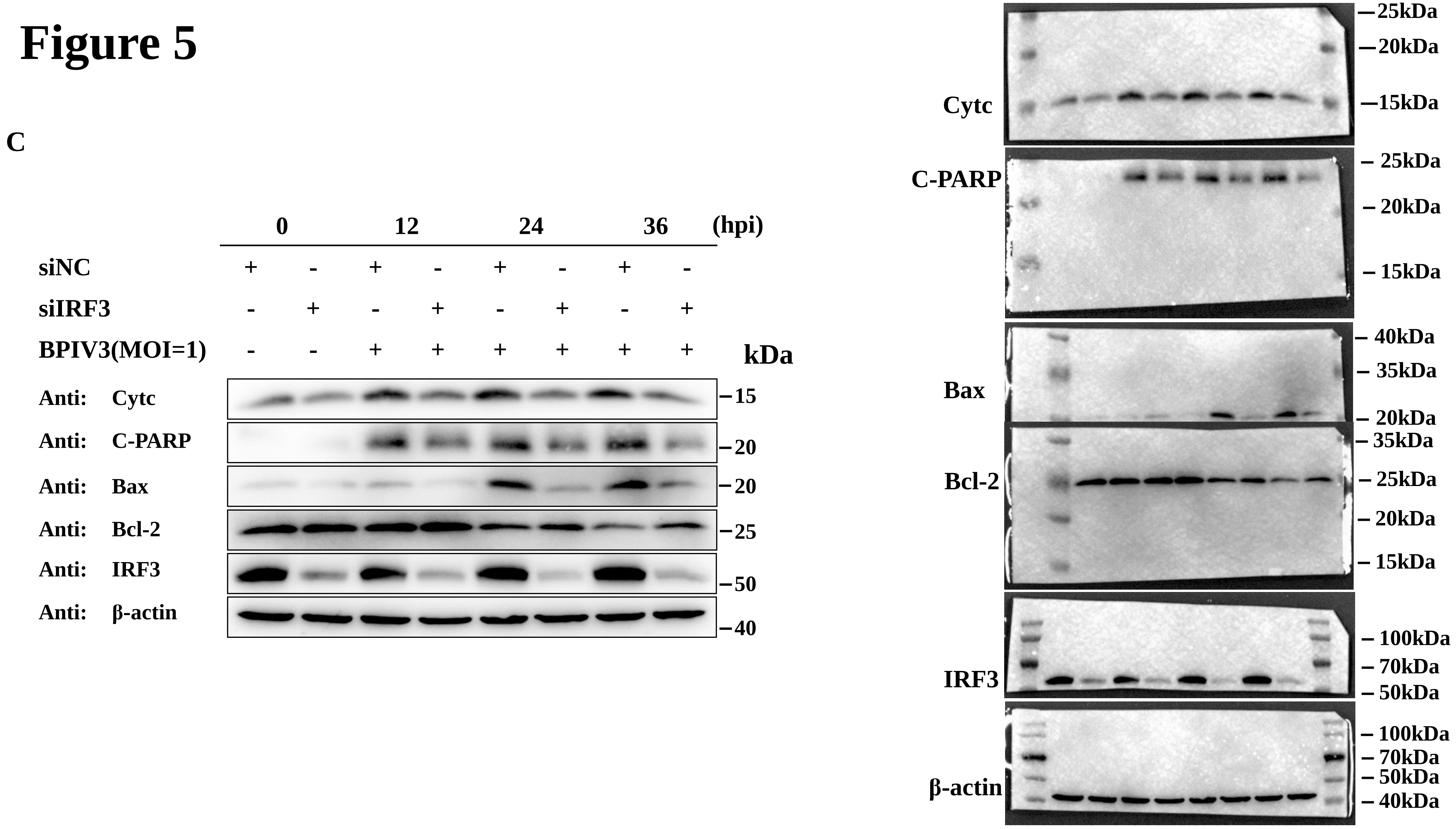
C
0
12
24
36
(hpi)
siNC + - + - + - + -
siIRF3 - + - + - + - +
BPIV3(MOI=1) - - + + + + + +
kDa
Anti: Cytc
Anti: C-PARP
Anti: Bax
Anti: Bcl-2
Anti: IRF3
Anti: β-actin
15
20
20
25
50
40
Cytc
25kDa
20kDa
15kDa
C-PARP
25kDa
20kDa
15kDa
Bax
40kDa
35kDa
20kDa
Bcl-2
35kDa
25kDa
20kDa
15kDa
IRF3
100kDa
70kDa
50kDa
β-actin
100kDa
70kDa
50kDa
40kDa

Figure 5

D

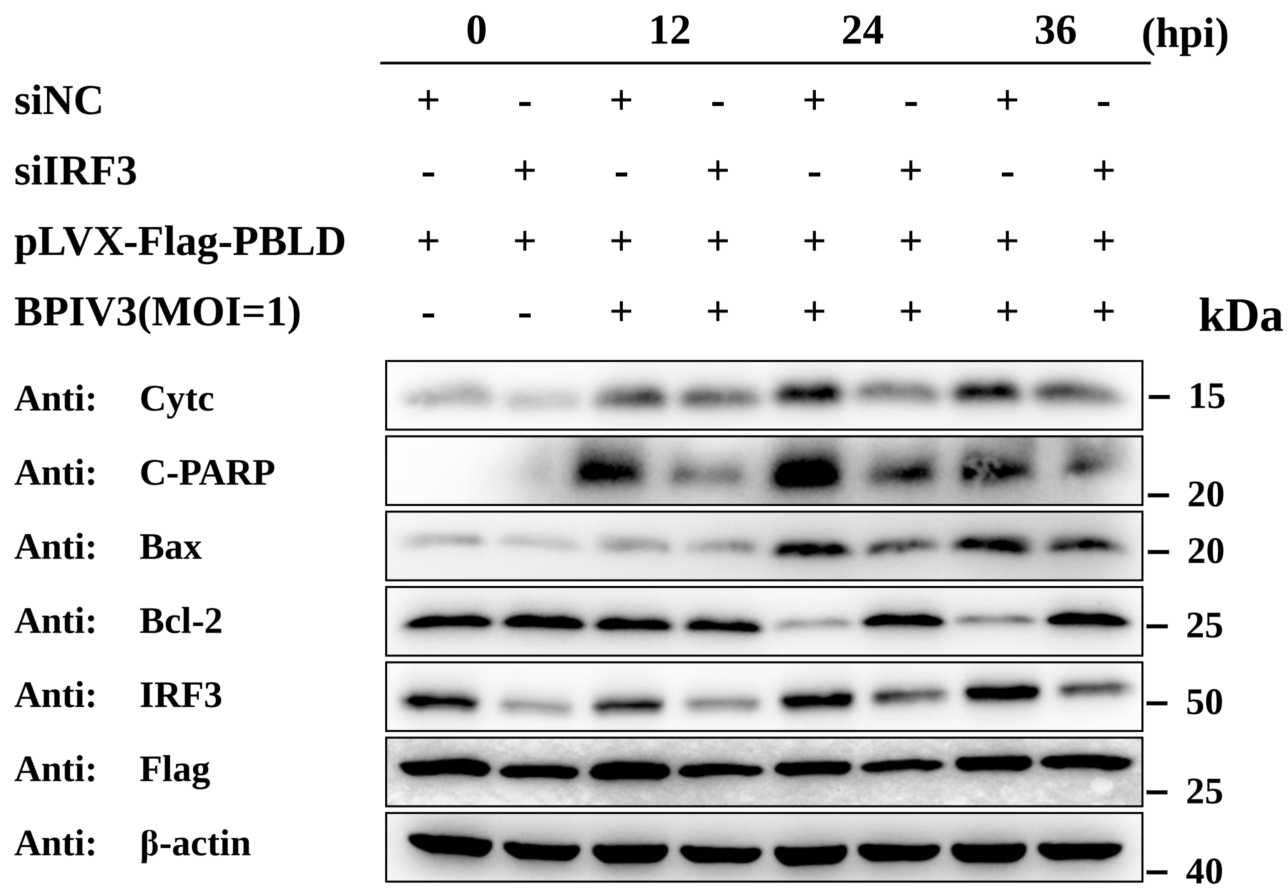

0
12
24
36
(hpi)
siNC
siIRF3
pLVX-Flag-PBLD
BPIV3(MOI=1)
kDa
Anti: Cytc
Anti: C-PARP
Anti: Bax
Anti: Bcl-2
Anti: IRF3
Anti: Flag
Anti: β-actin
15
20
20
25
50
25
40

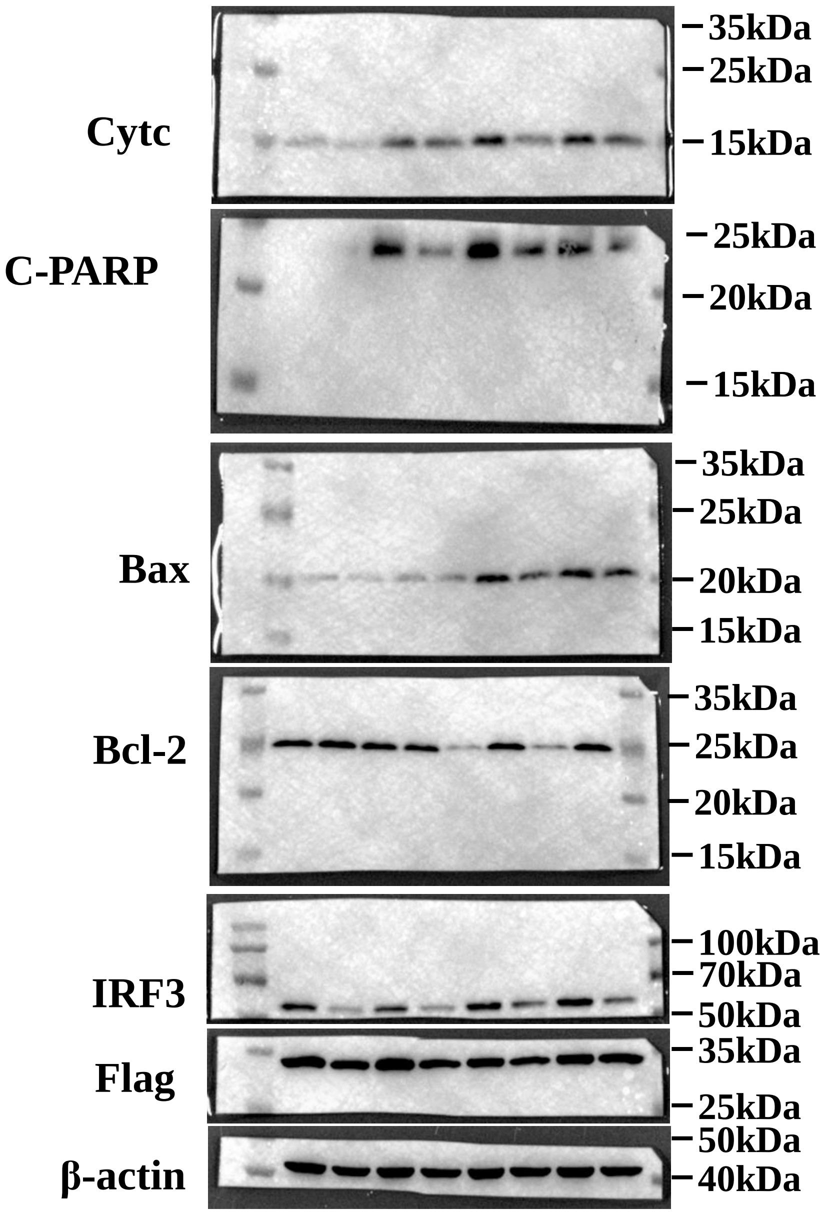

Cytc
C-PARP
Bax
Bcl-2
IRF3
Flag
β-actin
35kDa
25kDa
15kDa
25kDa
20kDa
15kDa
35kDa
25kDa
20kDa
15kDa
35kDa
25kDa
20kDa
15kDa
100kDa
70kDa
50kDa
35kDa
25kDa
50kDa
40kDa

Figure 5

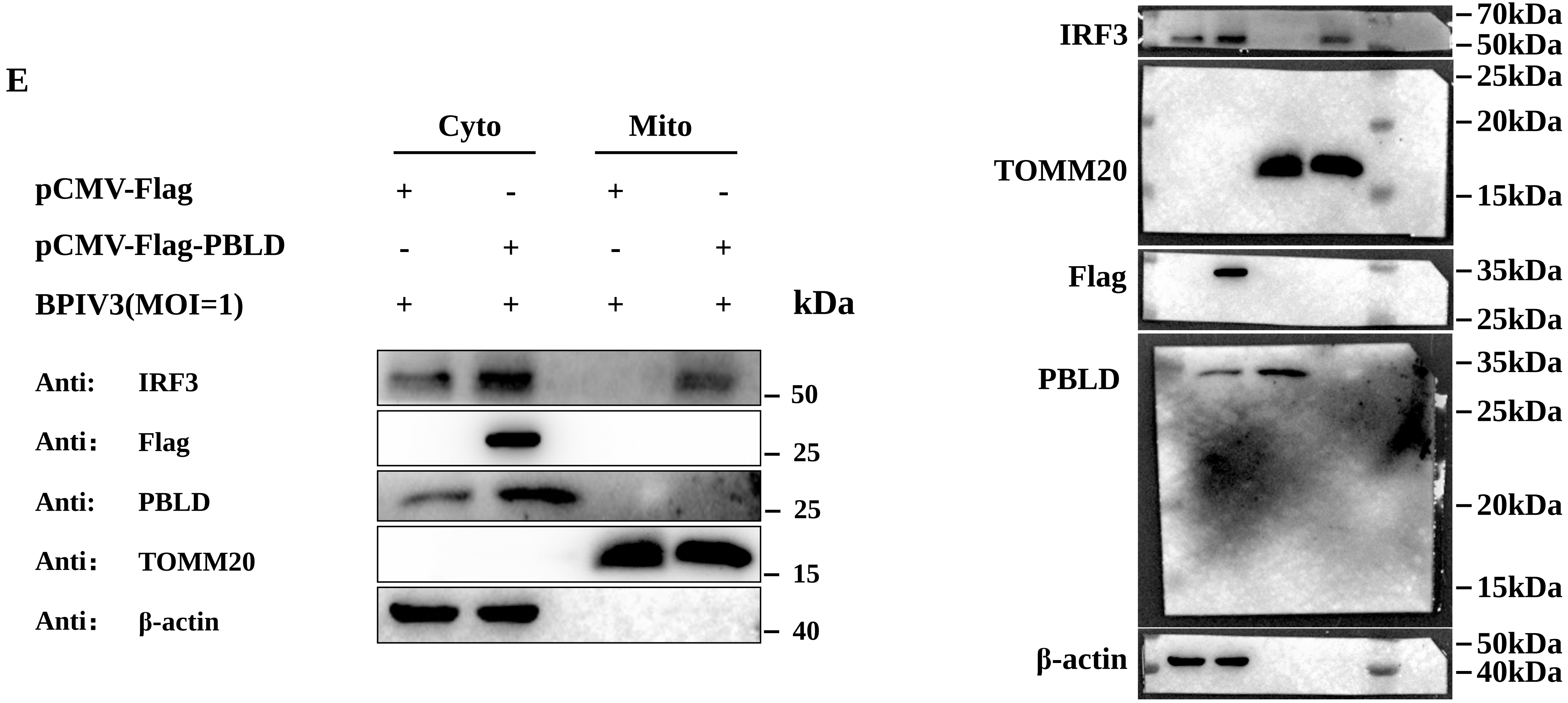
E
Cyto
Mito
pCMV-Flag
pCMV-Flag-PBLD
BPIV3(MOI=1)
+
-
+
-
-
+
-
+
+
+
+
+
kDa
Anti: IRF3
Anti: Flag
Anti: PBLD
Anti: TOMM20
Anti: β-actin
50
25
25
15
40
IRF3
TOMM20
Flag
PBLD
β-actin
70kDa
50kDa
25kDa
20kDa
15kDa
35kDa
25kDa
35kDa
25kDa
20kDa
15kDa
50kDa
40kDa

Figure 5

G

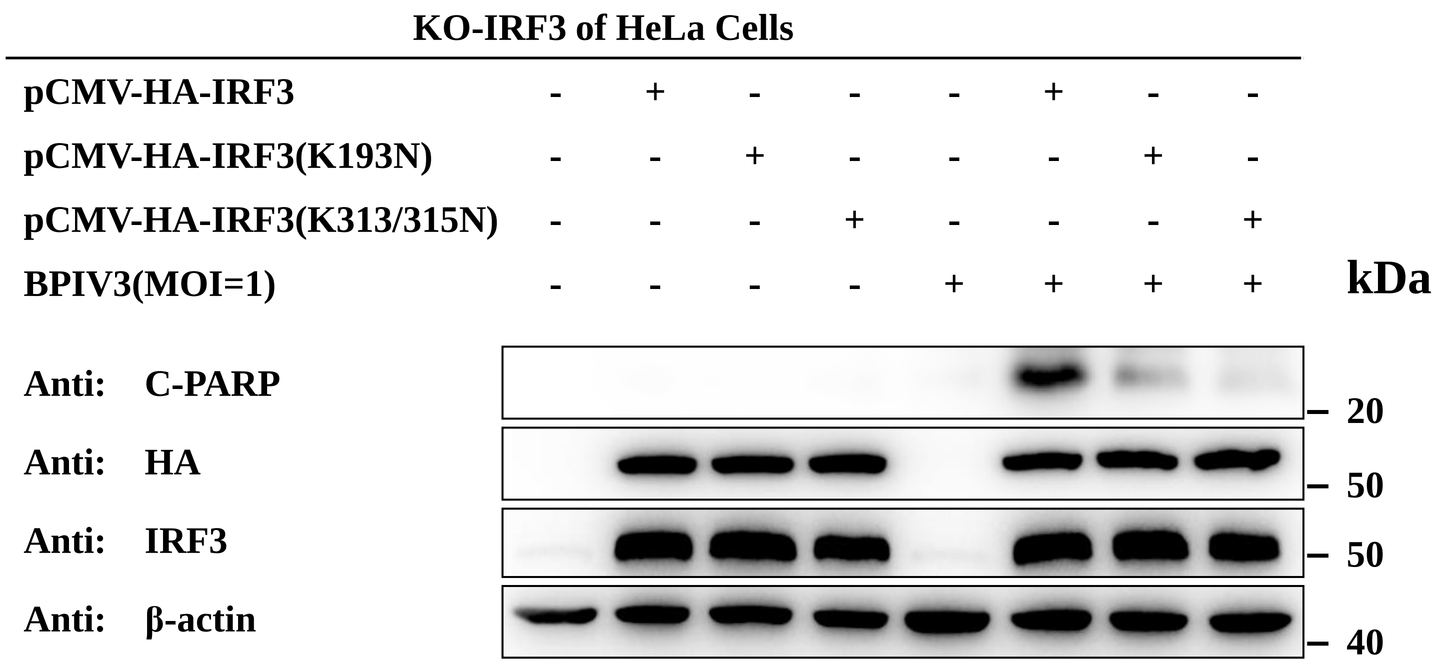

KO-IRF3 of HeLa Cells
pCMV-HA-IRF3 - + - - - + - -
pCMV-HA-IRF3(K193N) - - + - - - + -
pCMV-HA-IRF3(K313/315N) - - - + - - - +
BPIV3(MOI=1) - - - - + + + +
kDa
Anti: C-PARP
Anti: HA
Anti: IRF3
Anti: β-actin
20
50
50
40

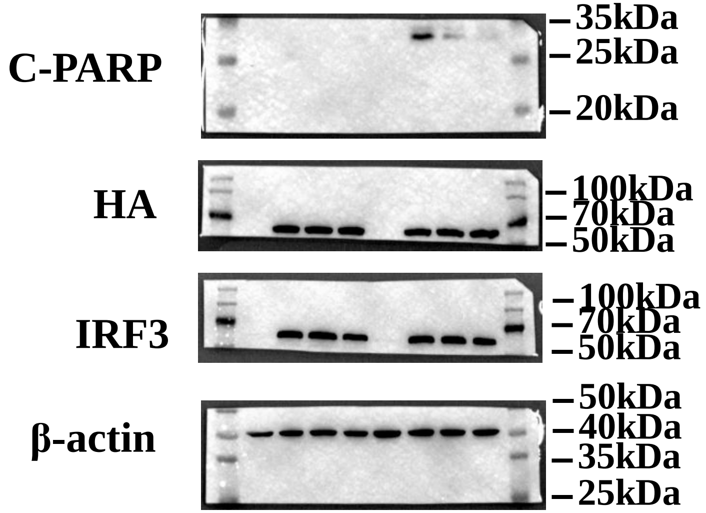

C-PARP
35kDa
25kDa
20kDa
HA
100kDa
70kDa
50kDa
IRF3
100kDa
70kDa
50kDa
β-actin
50kDa
40kDa
35kDa
25kDa

Figure 5

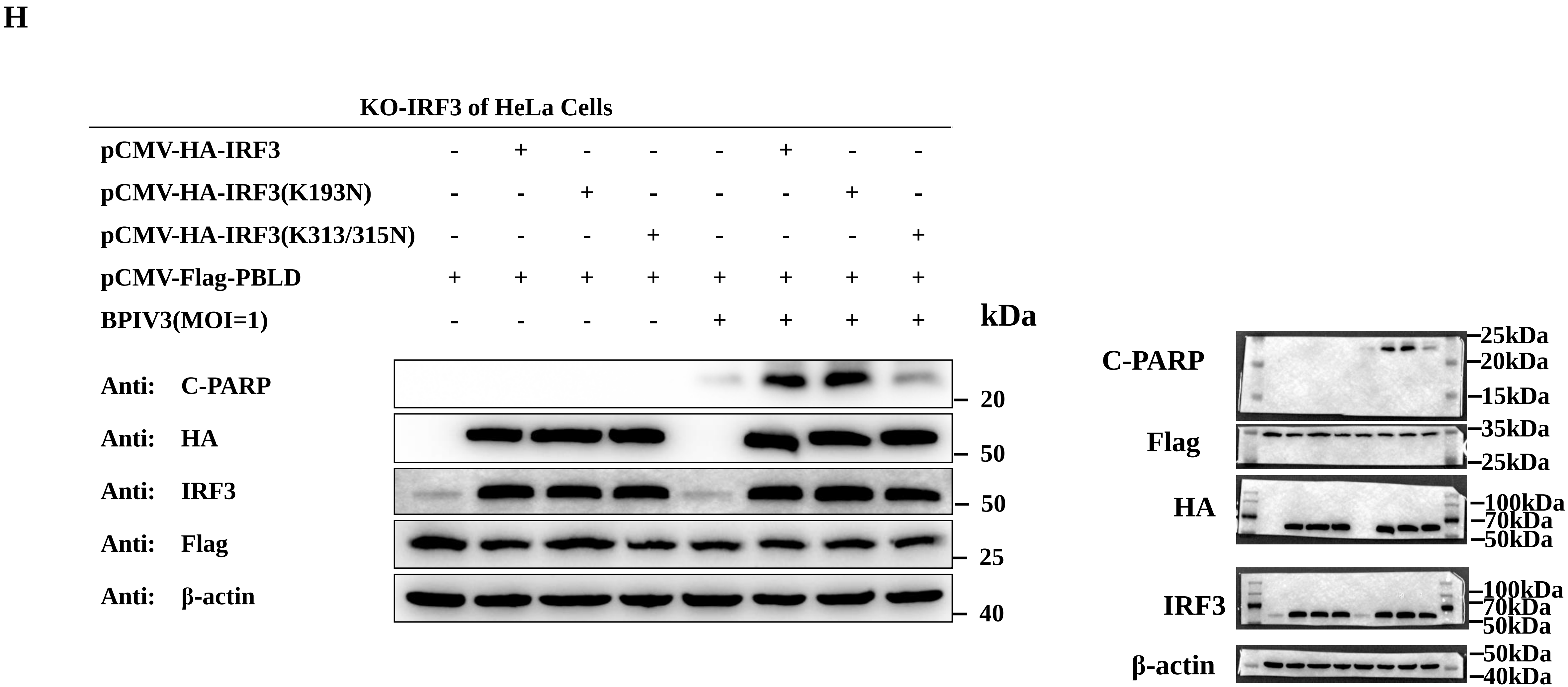
H
KO-IRF3 of HeLa Cells
pCMV-HA-IRF3 - + - - - + - -
pCMV-HA-IRF3(K193N) - - + - - - + -
pCMV-HA-IRF3(K313/315N) - - - + - - - +
pCMV-Flag-PBLD + + + + + + + +
BPIV3(MOI=1) - - - - + + + +
kDa
Anti: C-PARP
20
Anti: HA
50
Anti: IRF3
50
Anti: Flag
25
Anti: β-actin
40
C-PARP
25kDa
20kDa
15kDa
Flag
35kDa
25kDa
HA
100kDa
70kDa
50kDa
IRF3
100kDa
70kDa
50kDa
β-actin
50kDa
40kDa

Figure 5

I

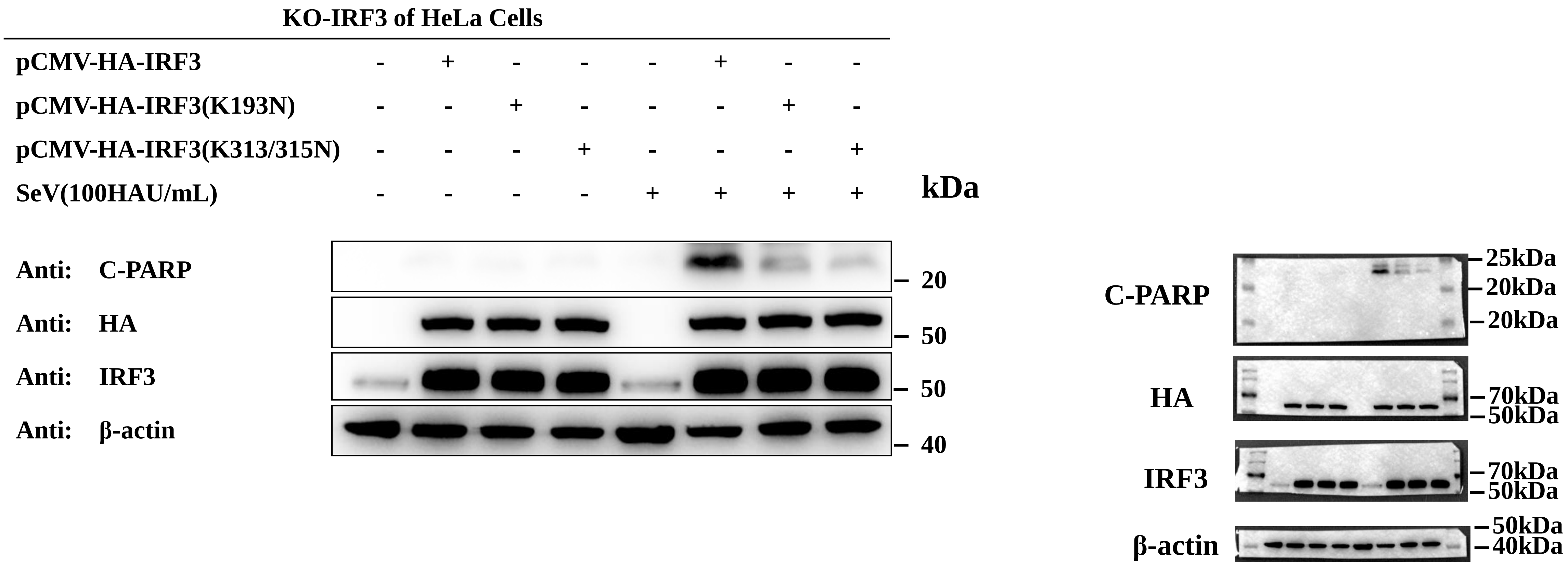
KO-IRF3 of HeLa Cells
pCMV-HA-IRF3 - + - - - + - -
pCMV-HA-IRF3(K193N) - - + - - - + -
pCMV-HA-IRF3(K313/315N) - - - + - - - +
SeV(100HAU/mL) - - - - + + + +
kDa
Anti: C-PARP
Anti: HA
Anti: IRF3
Anti: β-actin
20
50
50
40
C-PARP
HA
IRF3
β-actin
25kDa
20kDa
20kDa
70kDa
50kDa
70kDa
50kDa
50kDa
40kDa

Figure 5

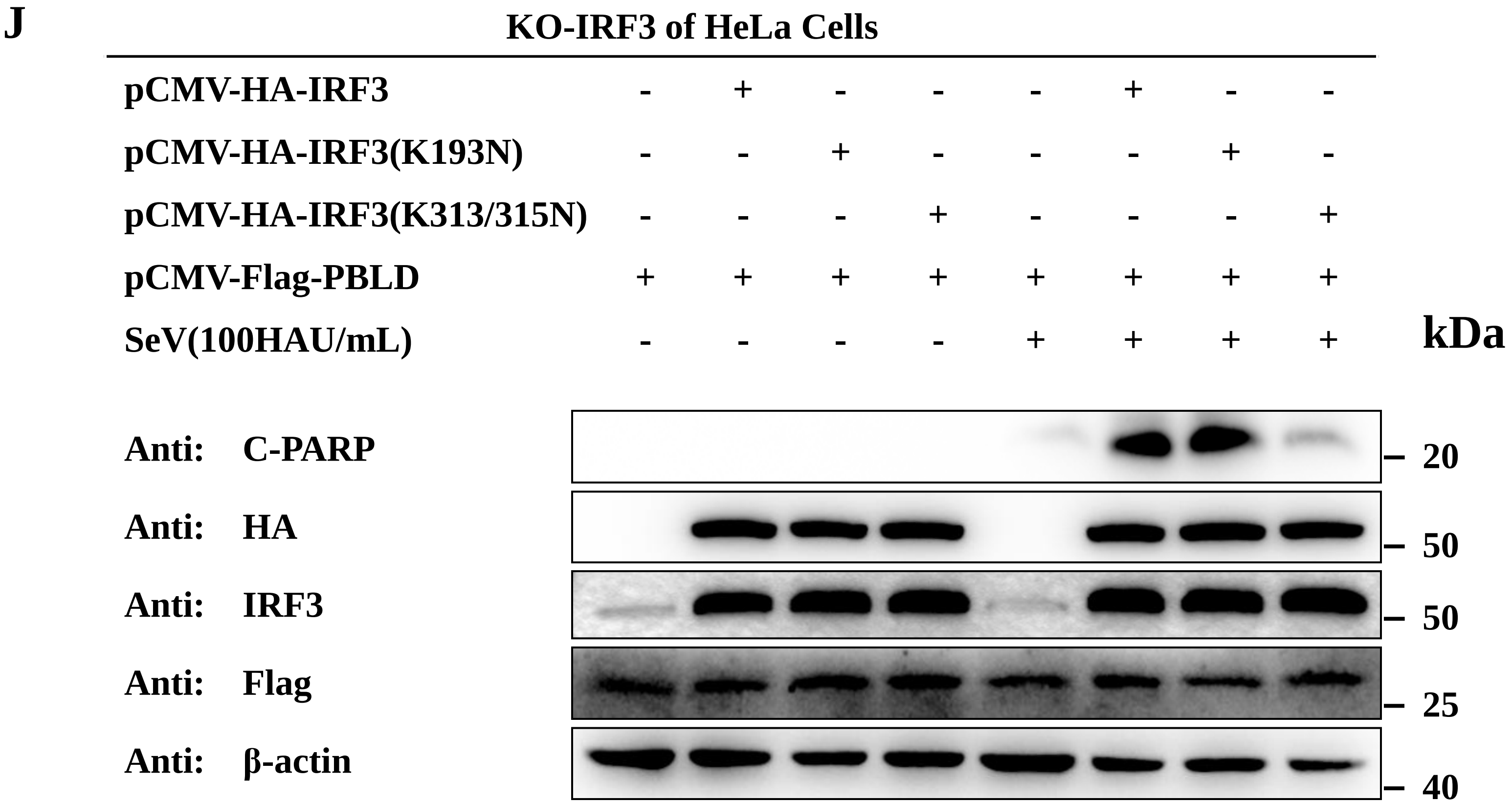
J
KO-IRF3 of HeLa Cells
pCMV-HA-IRF3 - + - - - + - -
pCMV-HA-IRF3(K193N) - - + - - - + -
pCMV-HA-IRF3(K313/315N) - - - + - - - +
pCMV-Flag-PBLD + + + + + + + +
SeV(100HAU/mL) - - - - + + + +
kDa
Anti: C-PARP
Anti: HA
Anti: IRF3
Anti: Flag
Anti: β-actin
20
50
50
25
40

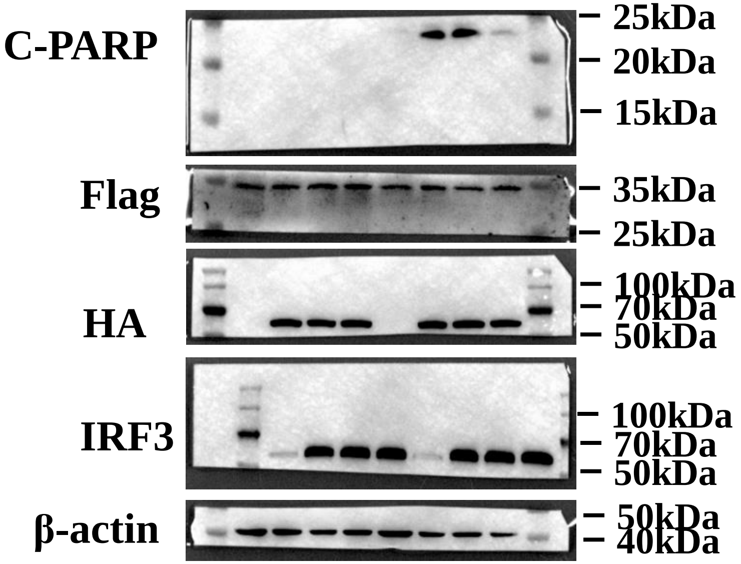
C-PARP
Flag
HA
IRF3
β-actin
25kDa
20kDa
15kDa
35kDa
25kDa
100kDa
70kDa
50kDa
100kDa
70kDa
50kDa
50kDa
40kDa

FIG6

Figure 6

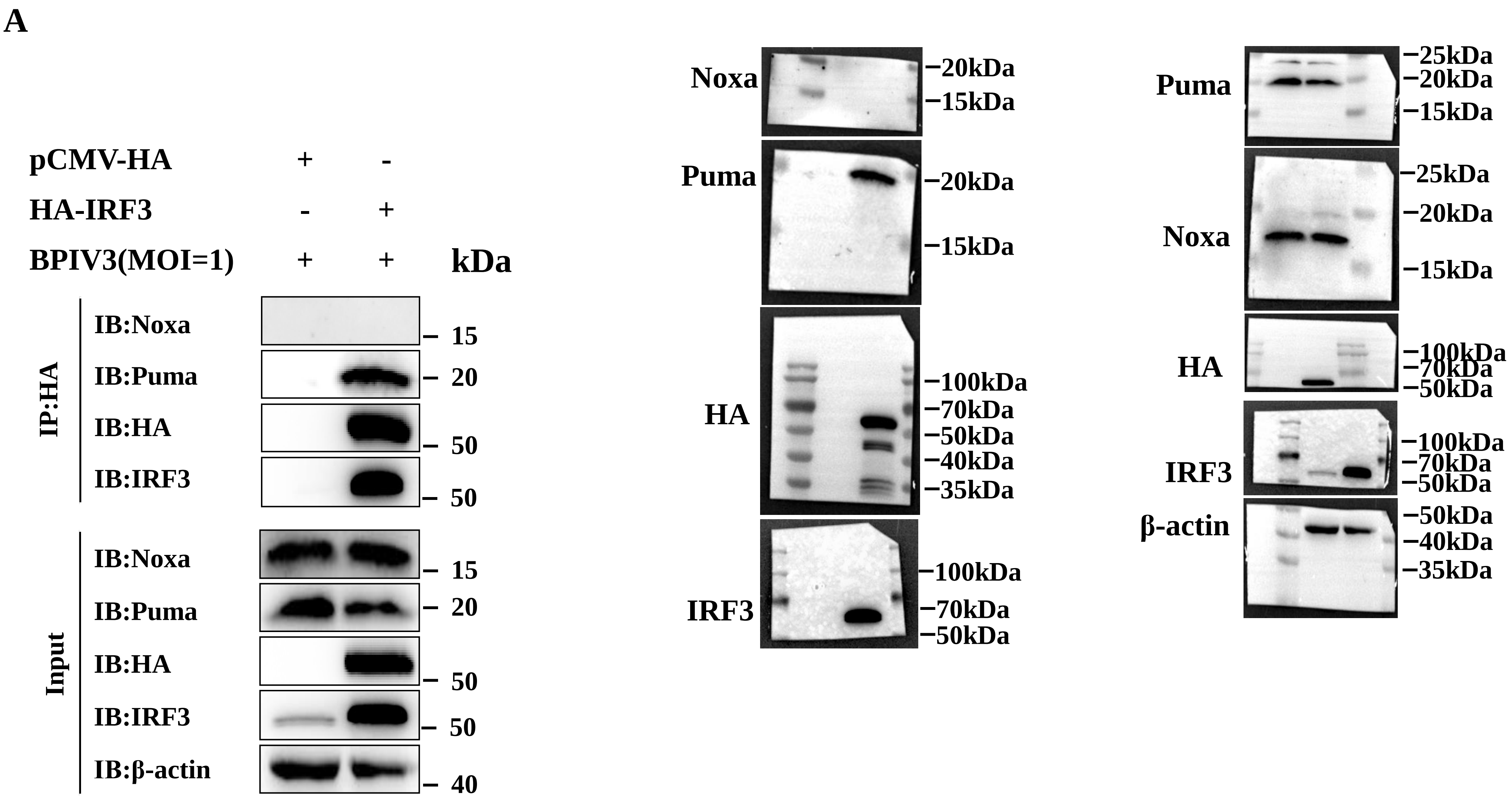

A
pCMV-HA + -
HA-IRF3 - +
BPIV3(MOI=1) + +
kDa
IP:HA
IB:Noxa
IB:Puma
IB:HA
IB:IRF3
Input
IB:Noxa
IB:Puma
IB:HA
IB:IRF3
IB:β-actin
15
20
50
50
15
20
50
50
40
Noxa
Puma
HA
IRF3
20kDa
15kDa
20kDa
15kDa
100kDa
70kDa
50kDa
40kDa
35kDa
100kDa
70kDa
50kDa
Puma
Noxa
HA
IRF3
β-actin
25kDa
20kDa
15kDa
25kDa
20kDa
15kDa
100kDa
70kDa
50kDa
100kDa
70kDa
50kDa
50kDa
40kDa
35kDa

Figure 6

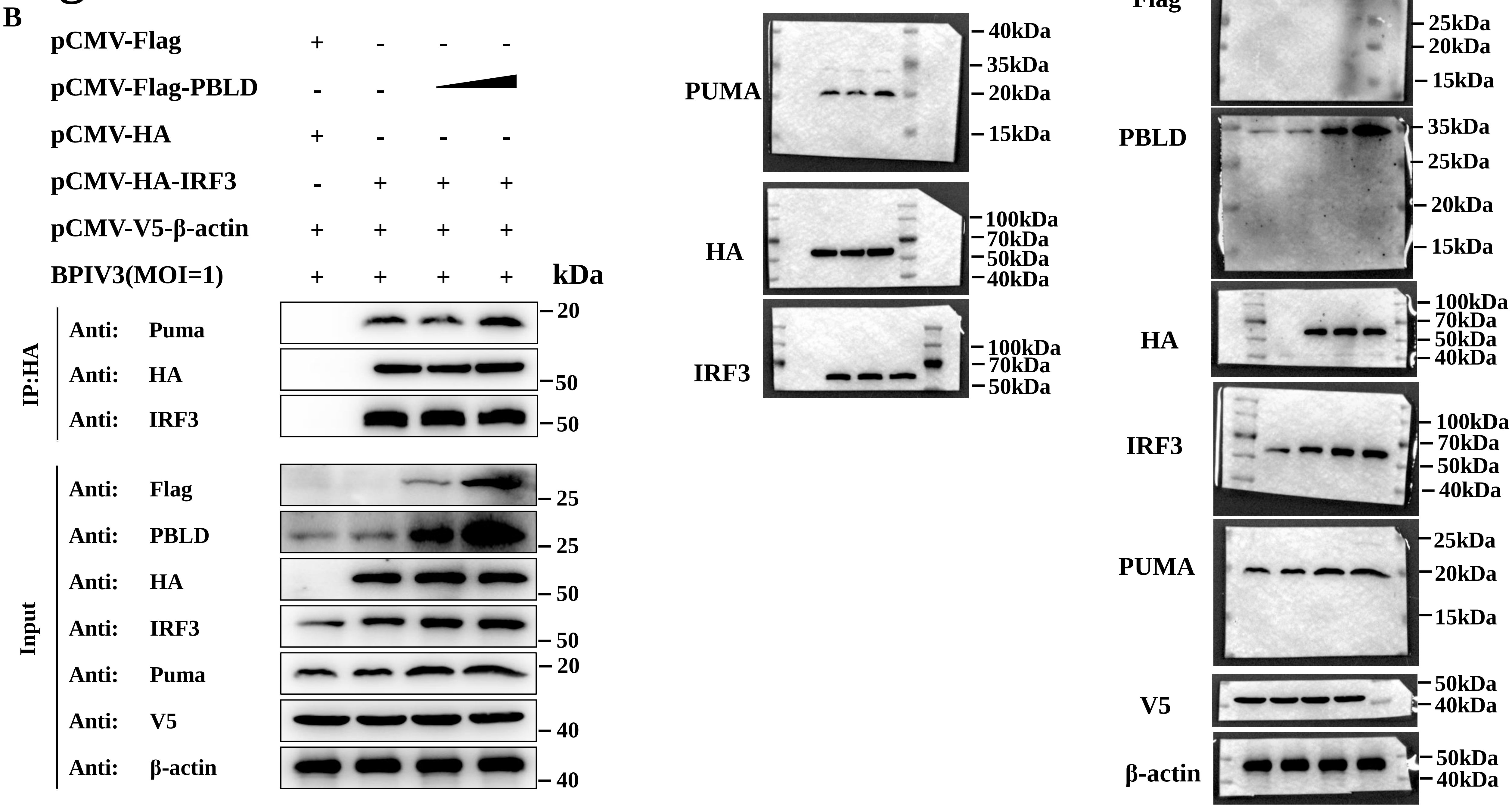
B
pCMV-Flag + - - -
pCMV-Flag-PBLD - -
pCMV-HA + - - -
pCMV-HA-IRF3 - + + +
pCMV-V5-β-actin + + + +
BPIV3(MOI=1) + + + +
kDa
IP:HA
Anti: Puma
Anti: HA
Anti: IRF3
Input
Anti: Flag
Anti: PBLD
Anti: HA
Anti: IRF3
Anti: Puma
Anti: V5
Anti: β-actin
20
50
50
25
25
50
50
20
40
40
PUMA
HA
IRF3
40kDa
35kDa
20kDa
15kDa
100kDa
70kDa
50kDa
40kDa
Flag
PBLD
HA
IRF3
PUMA
V5
β-actin
25kDa

Figure 6

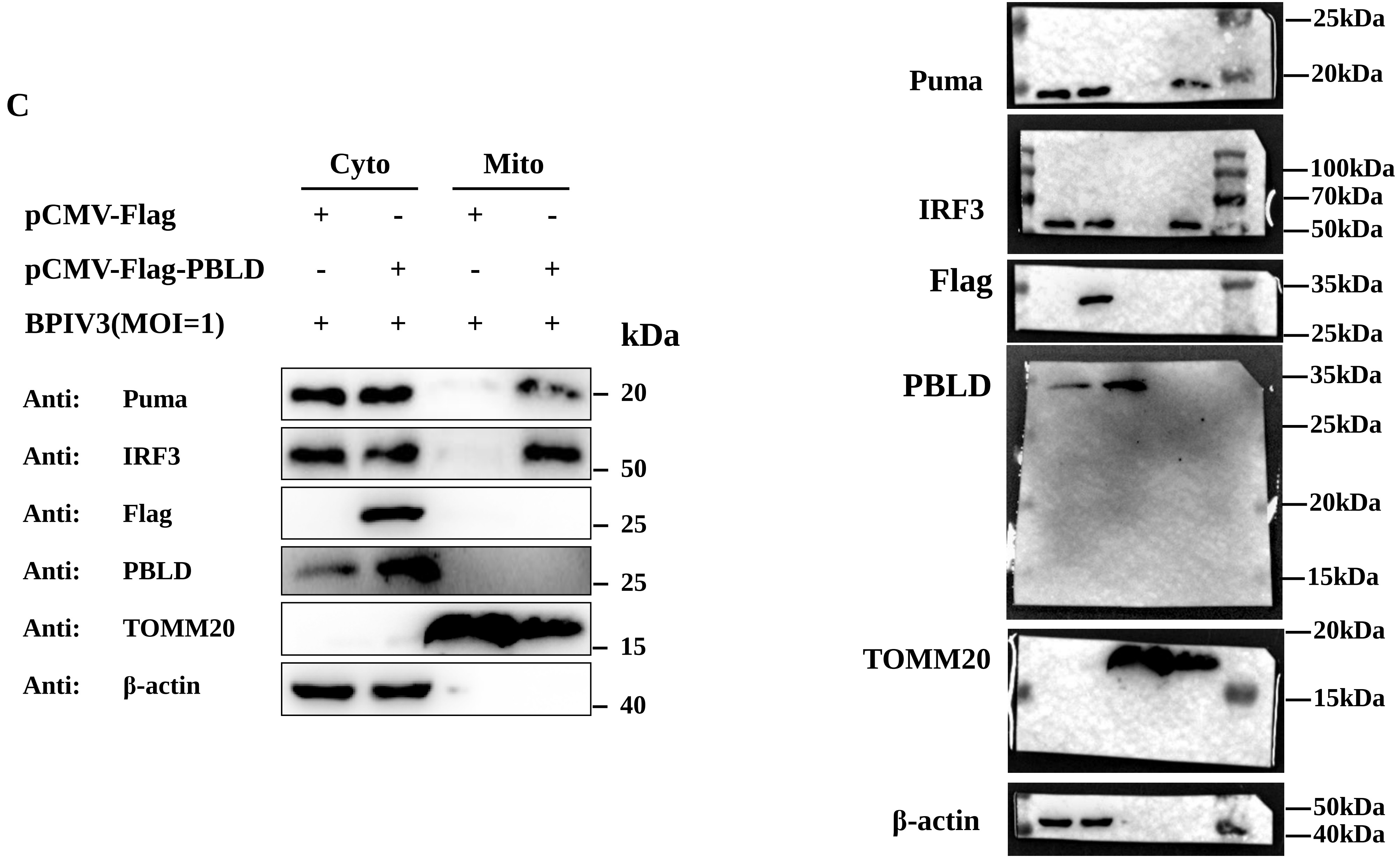
C
Cyto
Mito
pCMV-Flag + - + -
pCMV-Flag-PBLD - + - +
BPIV3(MOI=1) + + + +
kDa
Anti: Puma
20
Anti: IRF3
50
Anti: Flag
25
Anti: PBLD
25
Anti: TOMM20
15
Anti: β-actin
40
Puma
25kDa
20kDa
IRF3
100kDa
70kDa
50kDa
Flag
35kDa
25kDa
PBLD
35kDa
25kDa
20kDa
15kDa
TOMM20
20kDa
15kDa
β-actin
50kDa
40kDa

Figure 6

D

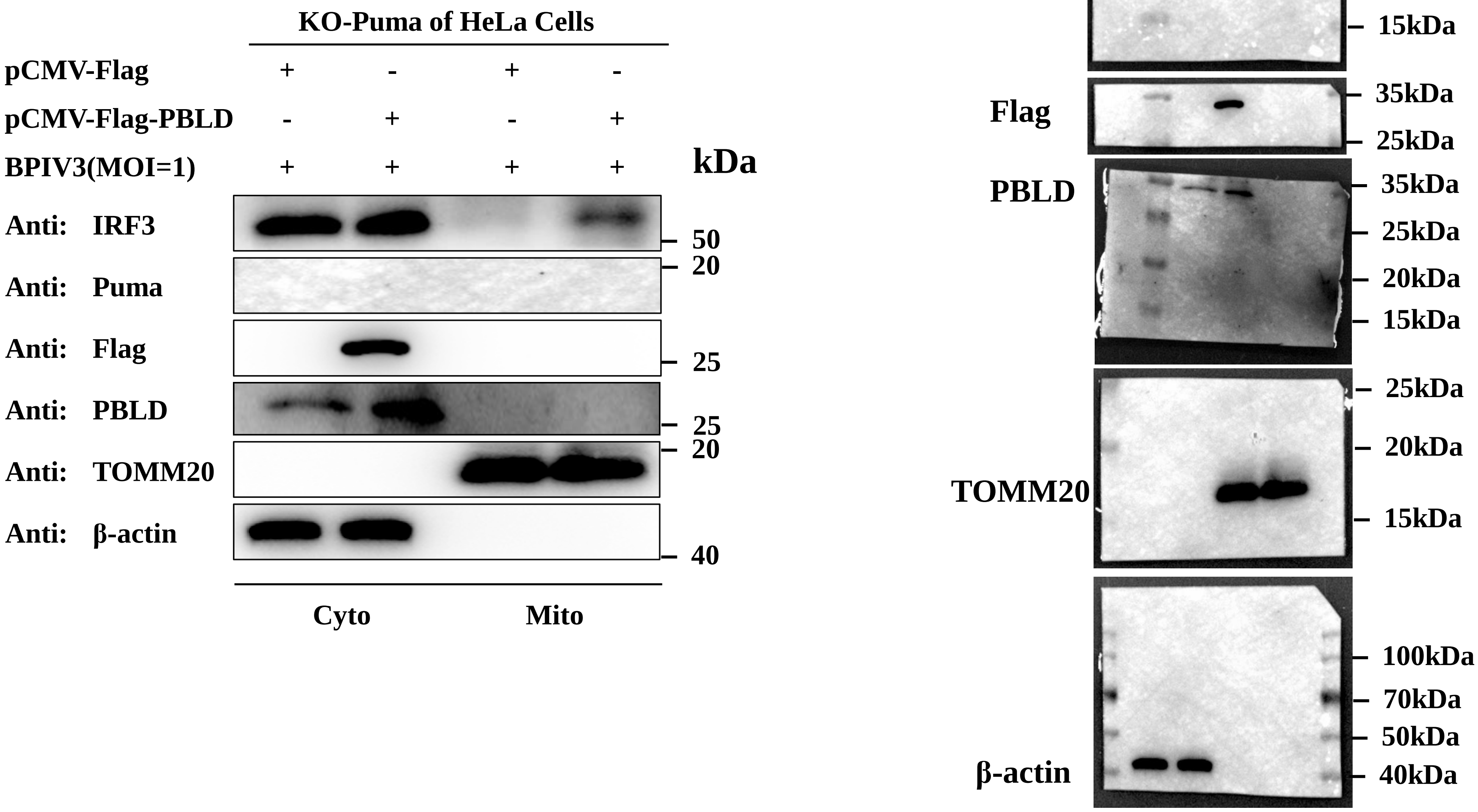
KO-Puma of HeLa Cells
pCMV-Flag + - + -
pCMV-Flag-PBLD - + - +
BPIV3(MOI=1) + + + +
kDa
Anti: IRF3
50
20
Anti: Puma
Anti: Flag
25
Anti: PBLD
25
20
Anti: TOMM20
Anti: β-actin
40
Cyto
Mito
IRF3
100kDa
70kDa
50kDa
Puma
25kDa
20kDa
15kDa
Flag
35kDa
25kDa
PBLD
35kDa
25kDa
20kDa
15kDa
TOMM20
25kDa
20kDa
15kDa
β-actin
100kDa
70kDa
50kDa
40kDa

Figure 6

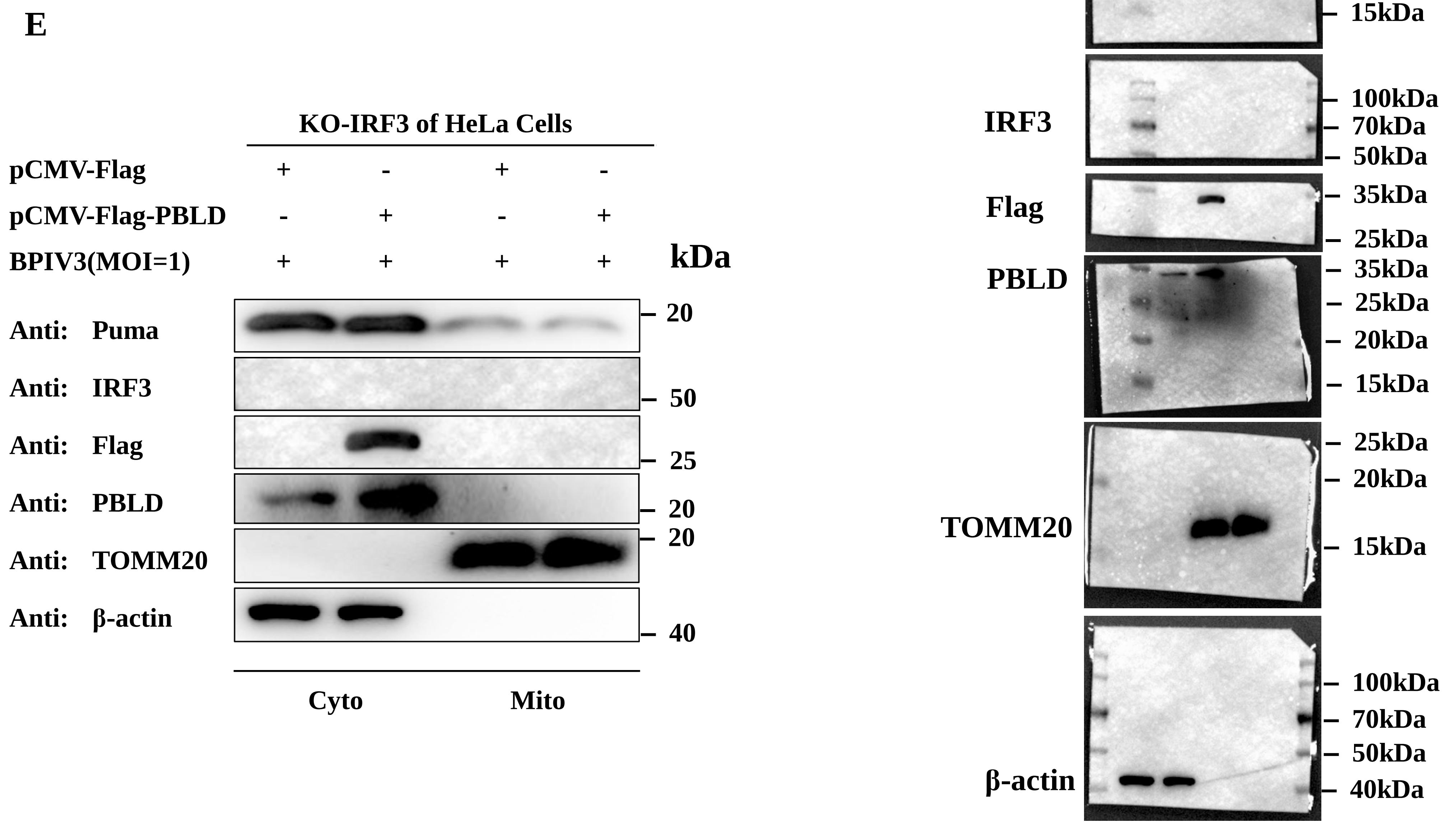
E
KO-IRF3 of HeLa Cells
pCMV-Flag + - + -
pCMV-Flag-PBLD - + - +
BPIV3(MOI=1) + + + +
kDa
Anti: Puma
Anti: IRF3
Anti: Flag
Anti: PBLD
Anti: TOMM20
Anti: β-actin
20
50
25
20
20
40
Cyto
Mito
Puma
25kDa
20kDa
15kDa
IRF3
100kDa
70kDa
50kDa
Flag
35kDa
25kDa
PBLD
35kDa
25kDa
20kDa
15kDa
TOMM20
25kDa
20kDa
15kDa
β-actin
100kDa
70kDa
50kDa
40kDa

Figure 6

F

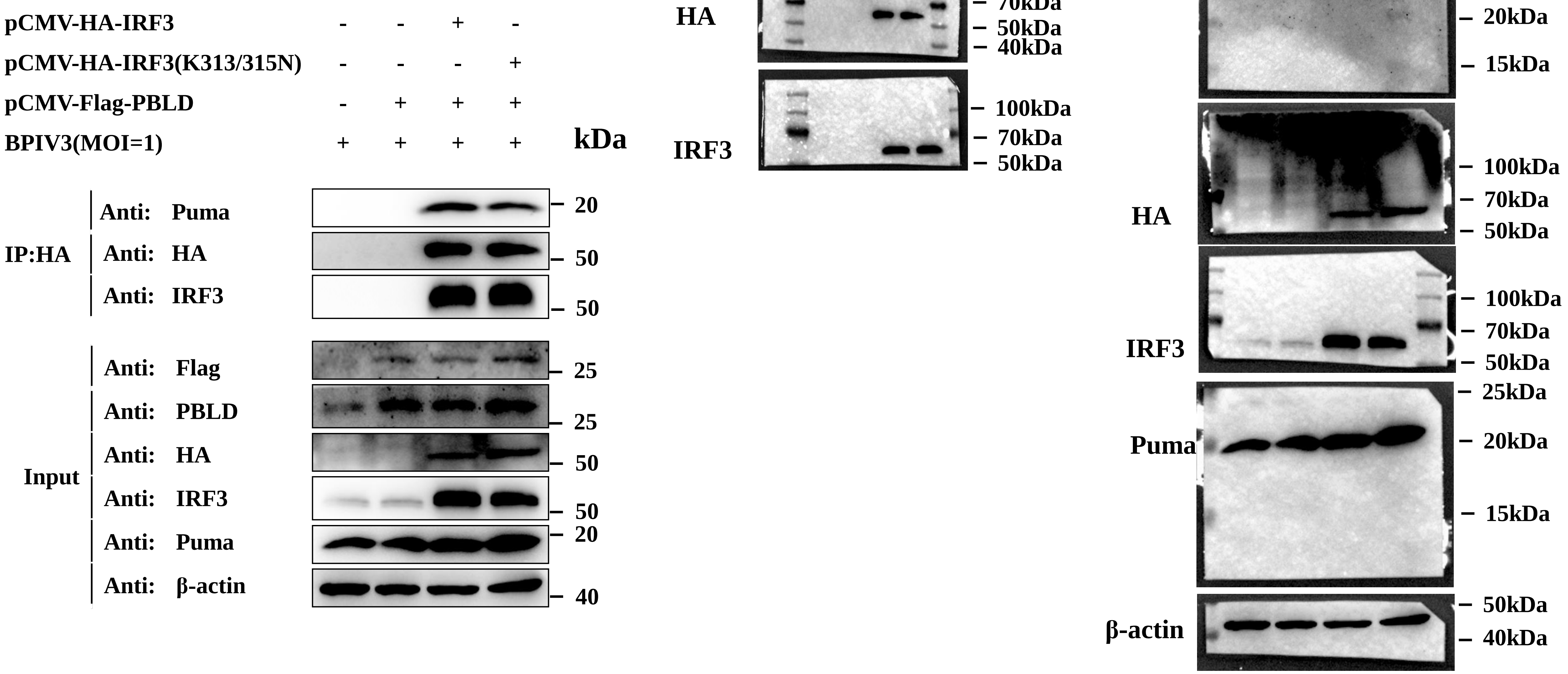
pCMV-HA-IRF3 - - + -
pCMV-HA-IRF3(K313/315N) - - - +
pCMV-Flag-PBLD - + + +
BPIV3(MOI=1) + + + +
kDa
IP:HA
Anti: Puma 20
Anti: HA 50
Anti: IRF3 50
Input
Anti: Flag 25
Anti: PBLD 25
Anti: HA 50
Anti: IRF3 50
Anti: Puma 20
Anti: β-actin 40
Puma 25kDa 20kDa 15kDa
HA 100kDa 70kDa 50kDa 40kDa
IRF3 100kDa 70kDa 50kDa
Flag 35kDa 25kDa
PBLD 35kDa 25kDa 20kDa 15kDa
HA 100kDa 70kDa 50kDa
IRF3 100kDa 70kDa 50kDa
Puma 25kDa 20kDa 15kDa
β-actin 50kDa 40kDa

Figure 6

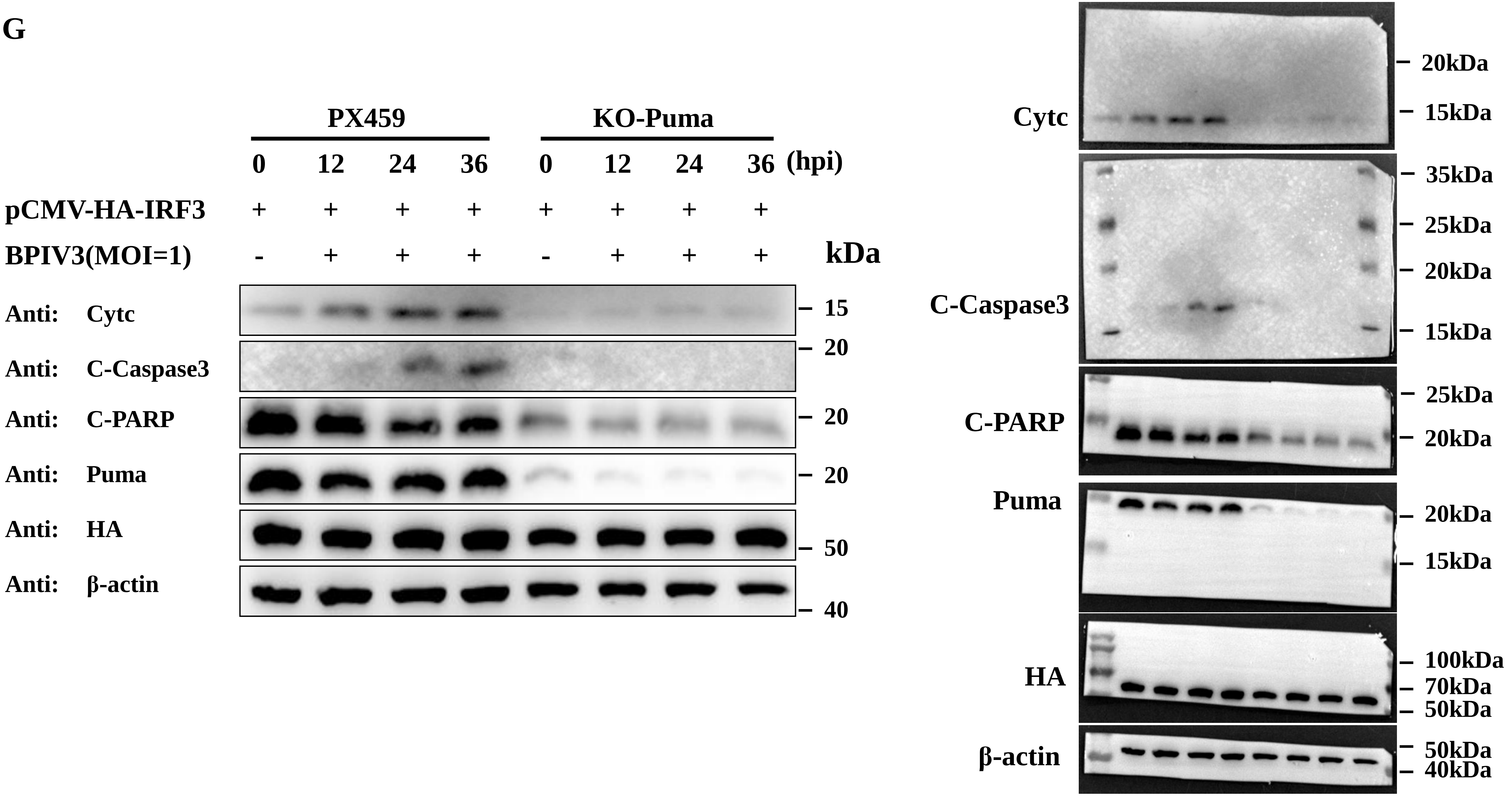
G
PX459
KO-Puma
0
12
24
36
0
12
24
36
(hpi)
pCMV-HA-IRF3
+ + + + + + + +
BPIV3(MOI=1)
- + + + - + + +
kDa
Anti: Cytc
Anti: C-Caspase3
Anti: C-PARP
Anti: Puma
Anti: HA
Anti: β-actin
15
20
20
20
50
40
Cytc
20kDa
15kDa
C-Caspase3
35kDa
25kDa
20kDa
15kDa
C-PARP
25kDa
20kDa
Puma
20kDa
15kDa
HA
100kDa
70kDa
50kDa
β-actin
50kDa
40kDa

Figure 6

H

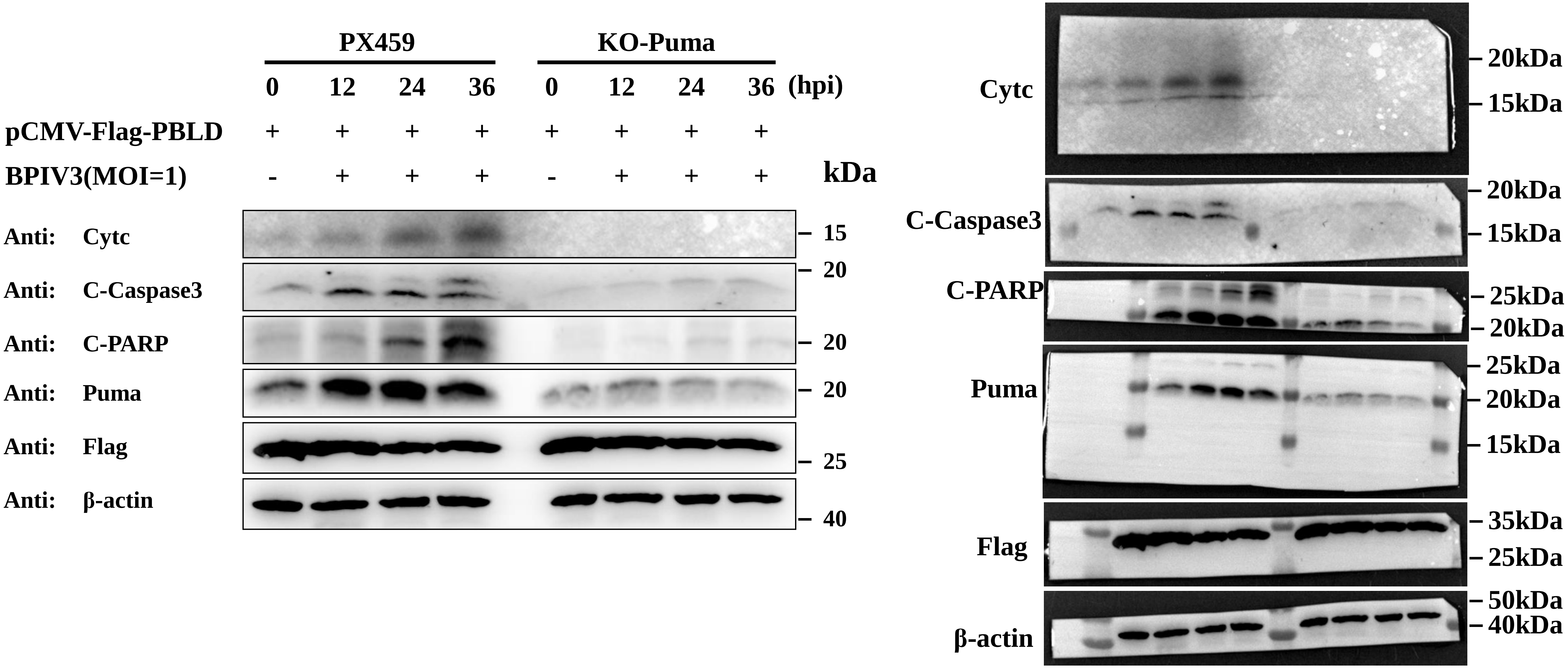
PX459
KO-Puma
0 12 24 36 0 12 24 36 (hpi)
pCMV-Flag-PBLD + + + + + + + +
BPIV3(MOI=1) - + + + - + + +
kDa
Anti: Cytc
Anti: C-Caspase3
Anti: C-PARP
Anti: Puma
Anti: Flag
Anti: β-actin
15
20
20
20
25
40
Cytc
C-Caspase3
C-PARP
Puma
Flag
β-actin
20kDa
15kDa
20kDa
15kDa
25kDa
20kDa
25kDa
20kDa
15kDa
35kDa
25kDa
50kDa
40kDa

Figure 6

I

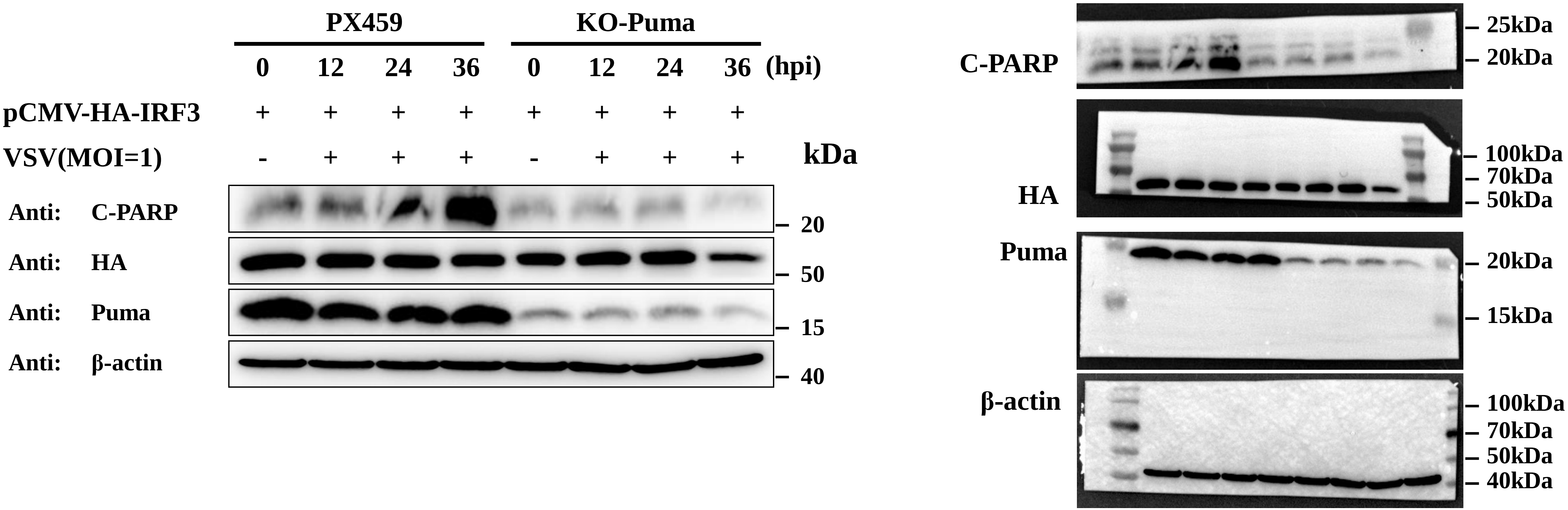
PX459
KO-Puma
0 12 24 36 0 12 24 36 (hpi)
pCMV-HA-IRF3 + + + + + + + +
VSV(MOI=1) - + + + - + + +
kDa
Anti: C-PARP
Anti: HA
Anti: Puma
Anti: β-actin
20
50
15
40
C-PARP
HA
Puma
β-actin
25kDa
20kDa
100kDa
70kDa
50kDa
20kDa
15kDa
100kDa
70kDa
50kDa
40kDa

Figure 6

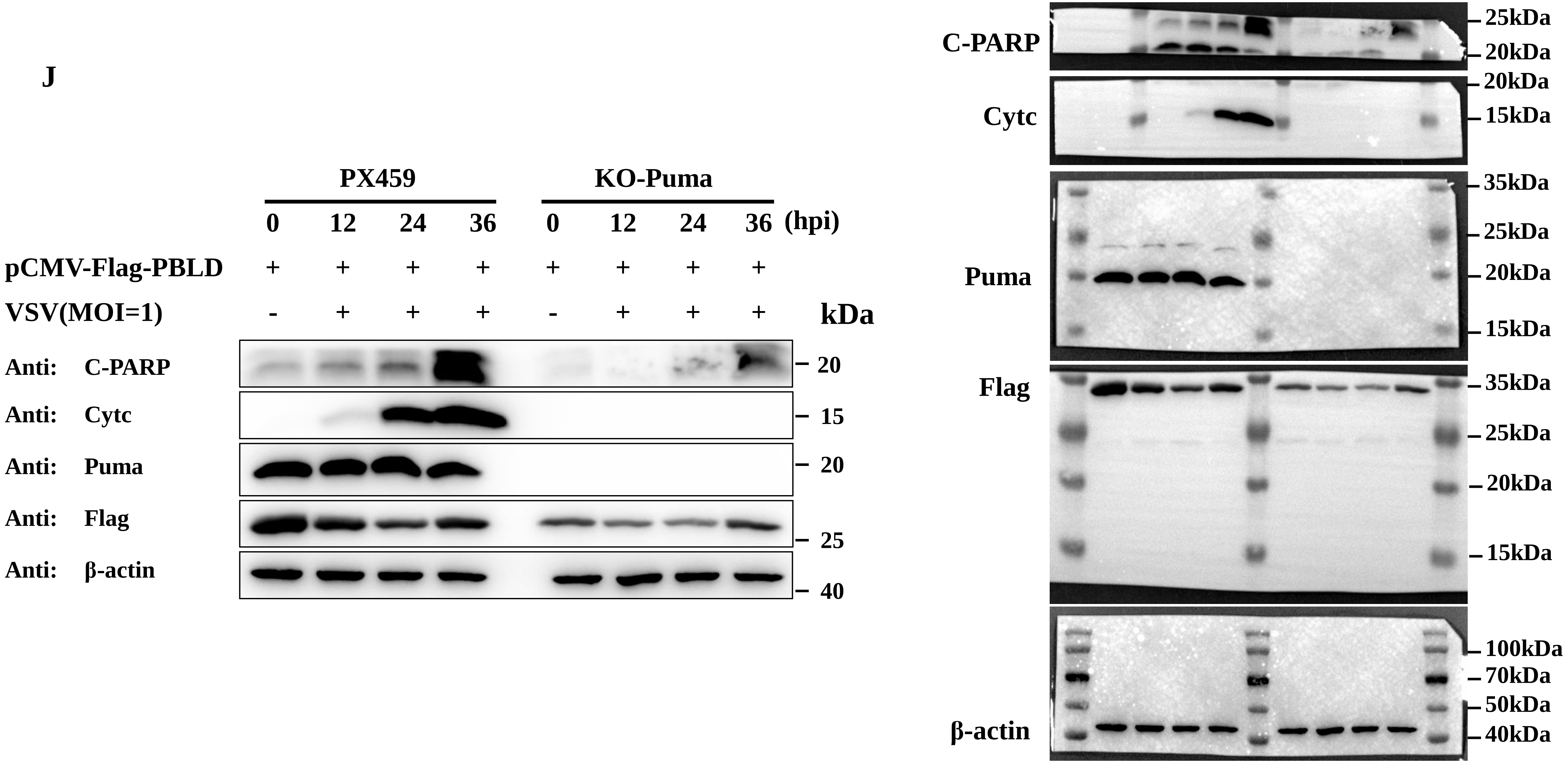
J
PX459
KO-Puma
0 12 24 36 0 12 24 36 (hpi)
pCMV-Flag-PBLD + + + + + + + +
VSV(MOI=1) - + + + - + + +
kDa
Anti: C-PARP
Anti: Cytc
Anti: Puma
Anti: Flag
Anti: β-actin
20
15
20
25
40
C-PARP
Cytc
Puma
Flag
β-actin
25kDa
20kDa
20kDa
15kDa
35kDa
25kDa
20kDa
15kDa
35kDa
25kDa
20kDa
15kDa
100kDa
70kDa
50kDa
40kDa

FIG7

Figure 7

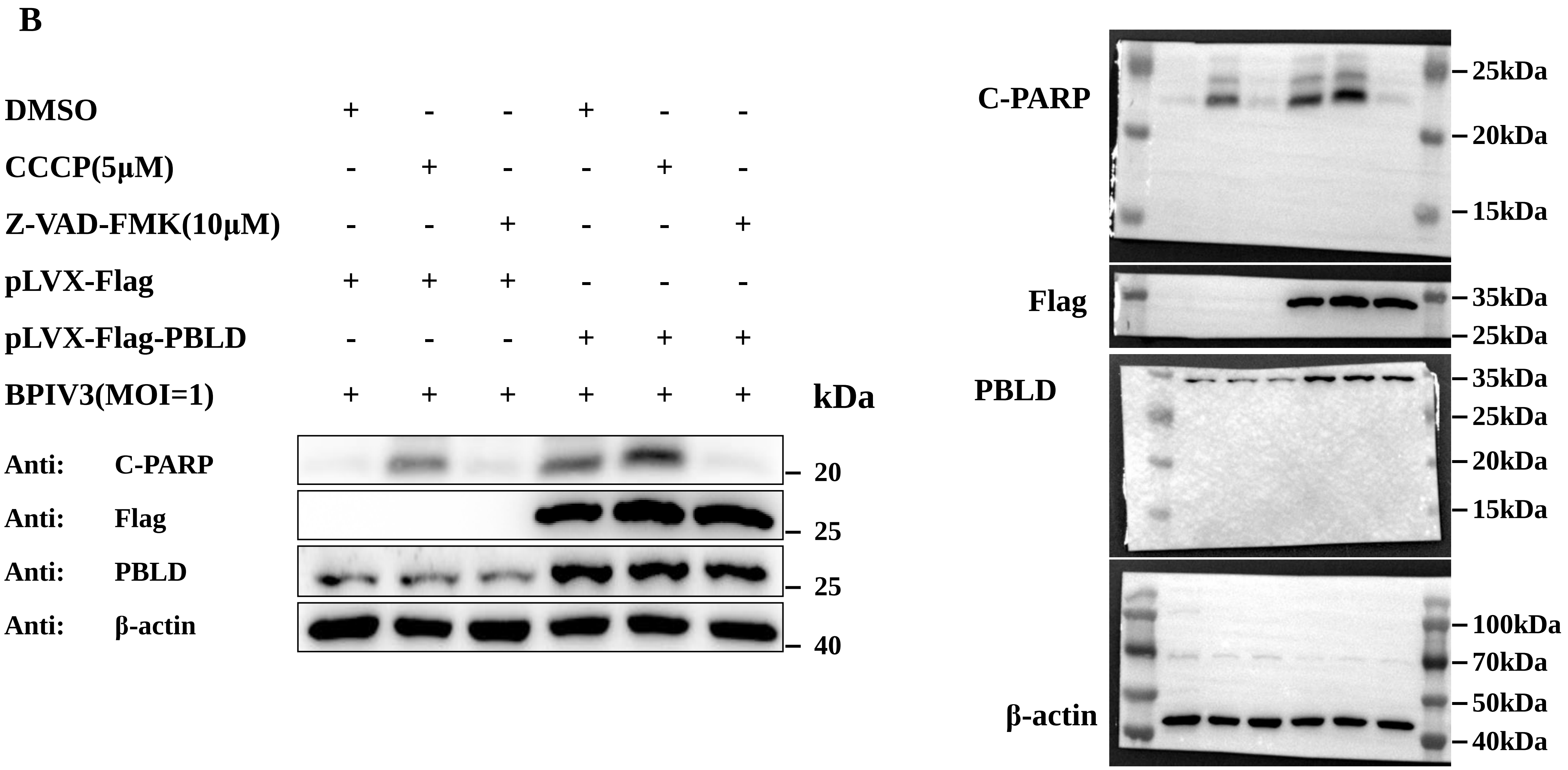
B
DMSO + - - + - -
CCCP(5μM) - + - - + -
Z-VAD-FMK(10μM) - - + - - +
pLVX-Flag + + + - - -
pLVX-Flag-PBLD - - - + + +
BPIV3(MOI=1) + + + + + +
kDa
Anti: C-PARP
20
Anti: Flag
25
Anti: PBLD
25
Anti: β-actin
40
C-PARP
25kDa
20kDa
15kDa
Flag
35kDa
25kDa
PBLD
35kDa
25kDa
20kDa
15kDa
β-actin
100kDa
70kDa
50kDa
40kDa

Figure 7

C

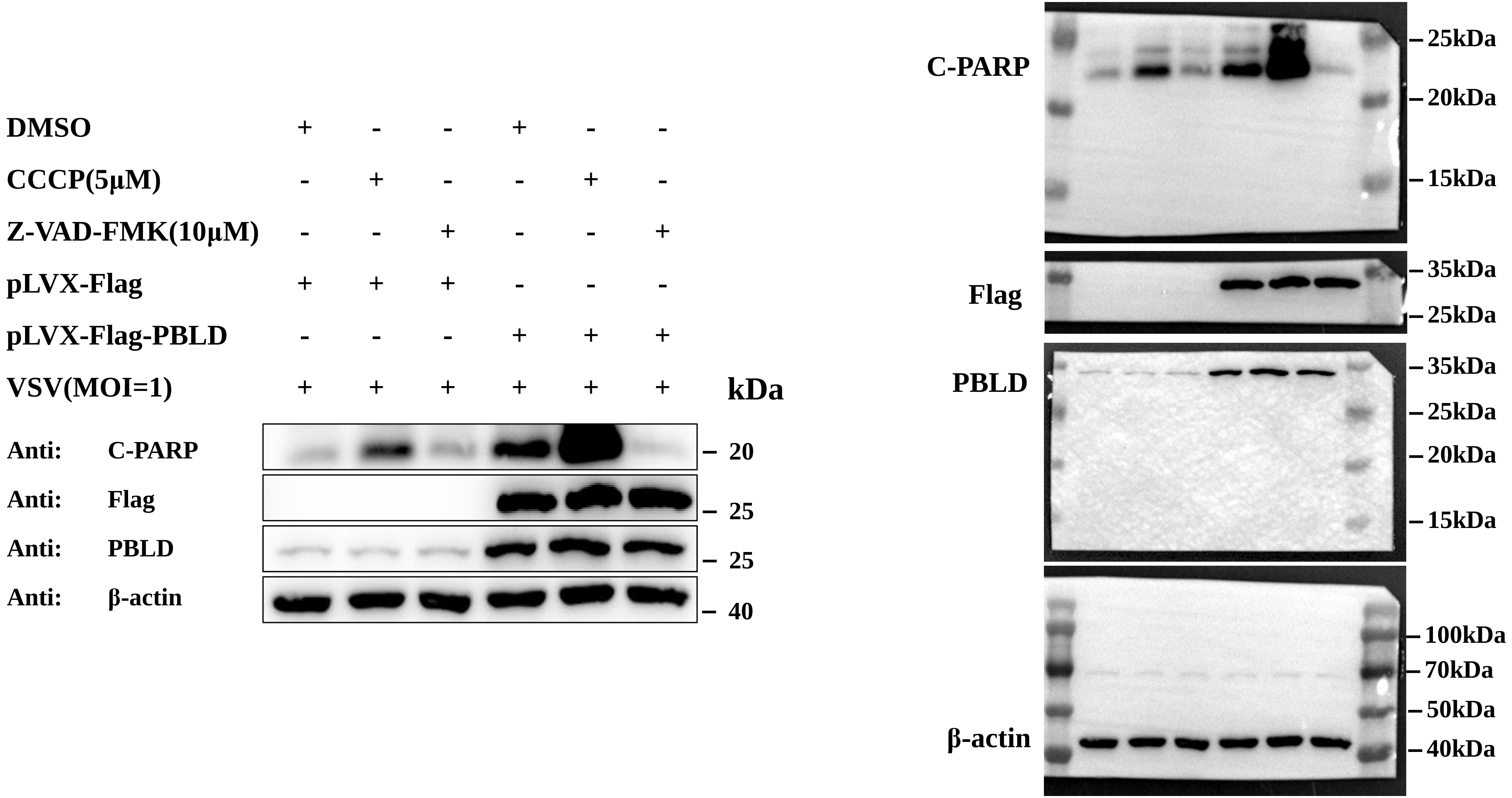
DMSO + - - + - -
CCCP(5μM) - + - - + -
Z-VAD-FMK(10μM) - - + - - +
pLVX-Flag + + + - - -
pLVX-Flag-PBLD - - - + + +
VSV(MOI=1) + + + + + +
kDa
Anti: C-PARP
Anti: Flag
Anti: PBLD
Anti: β-actin
20
25
25
40
C-PARP
25kDa
20kDa
15kDa
Flag
35kDa
25kDa
PBLD
35kDa
25kDa
20kDa
15kDa
β-actin
100kDa
70kDa
50kDa
40kDa

FIG10

Figure 10

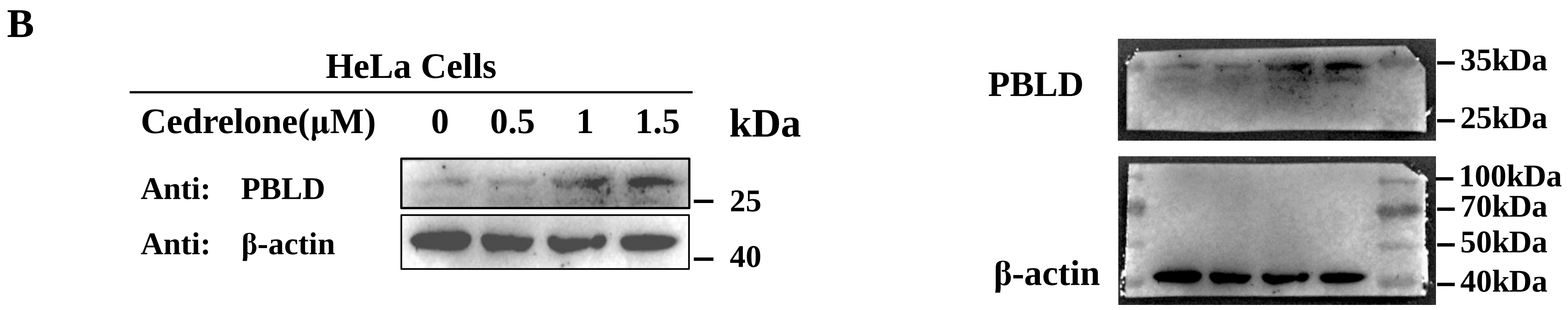
B
HeLa Cells
Cedrelone(μM)
0
0.5
1
1.5
kDa
Anti: PBLD
25
Anti: β-actin
40
PBLD
35kDa
25kDa
100kDa
70kDa
50kDa
β-actin
40kDa

Figure 10

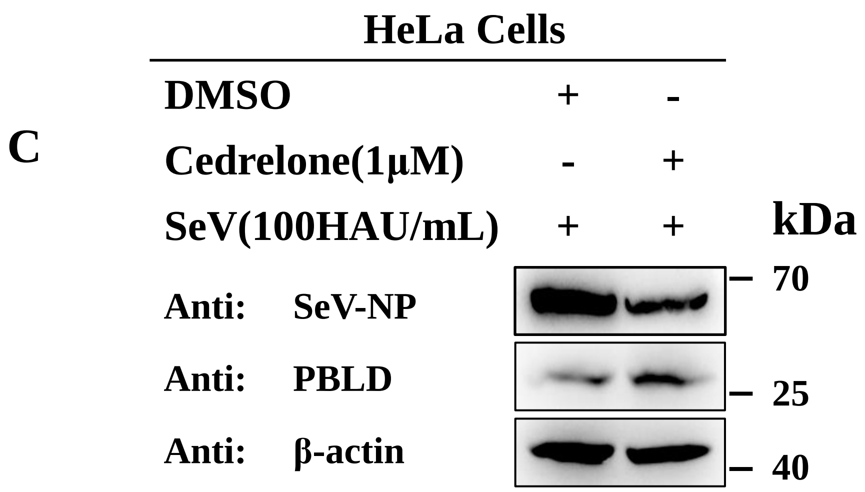
C
HeLa Cells
DMSO + -
Cedrelone(1μM) - +
SeV(100HAU/mL) + +
kDa
Anti: SeV-NP
Anti: PBLD
Anti: β-actin
70
25
40

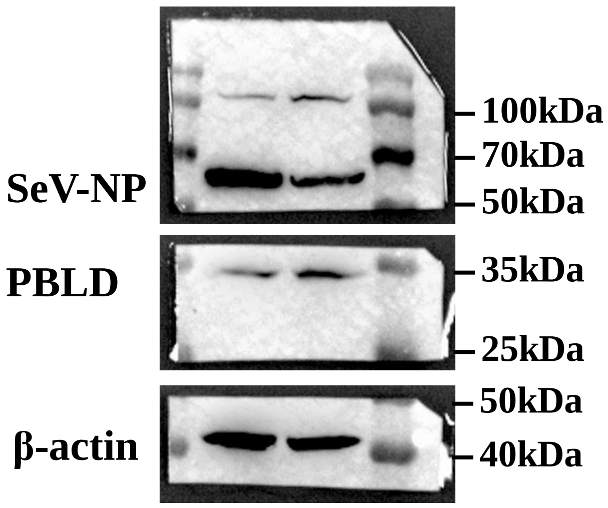
SeV-NP
PBLD
β-actin
100kDa
70kDa
50kDa
35kDa
25kDa
50kDa
40kDa

Supplementary Figure 1

Supplementary Figure 1

E

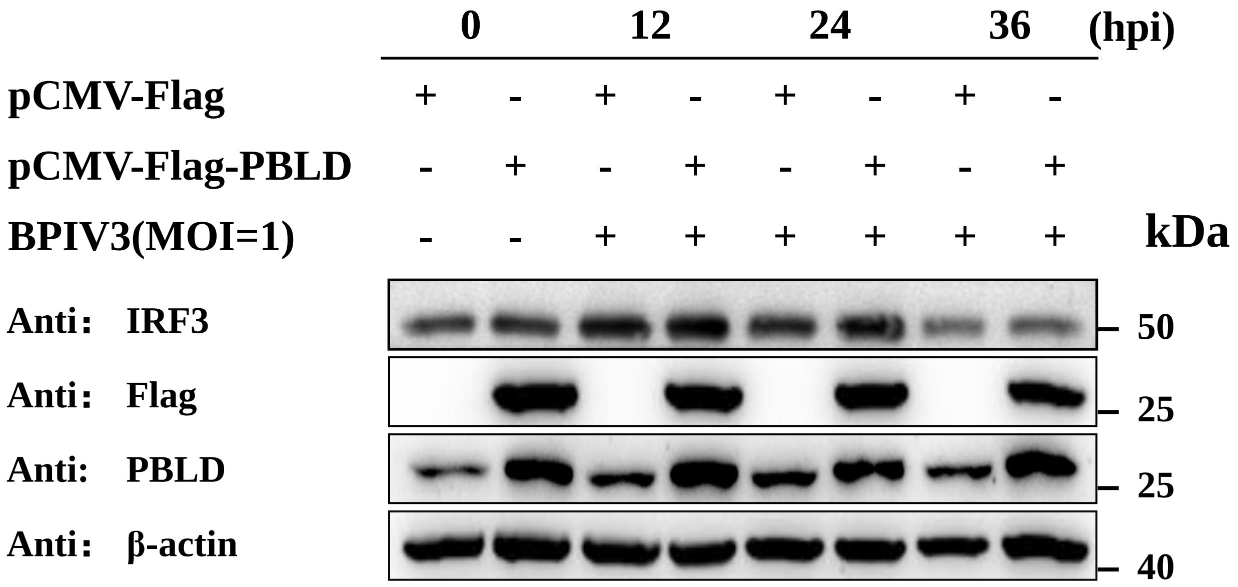

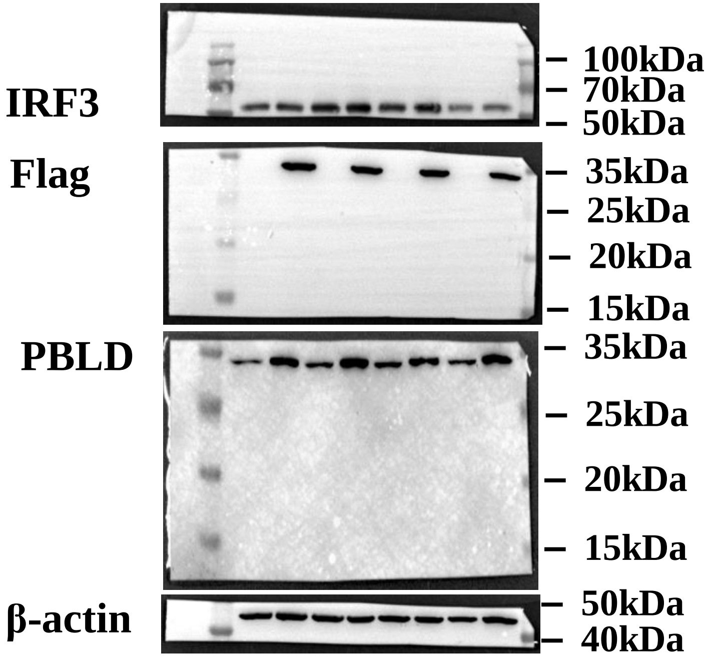

Supplementary Figure 1

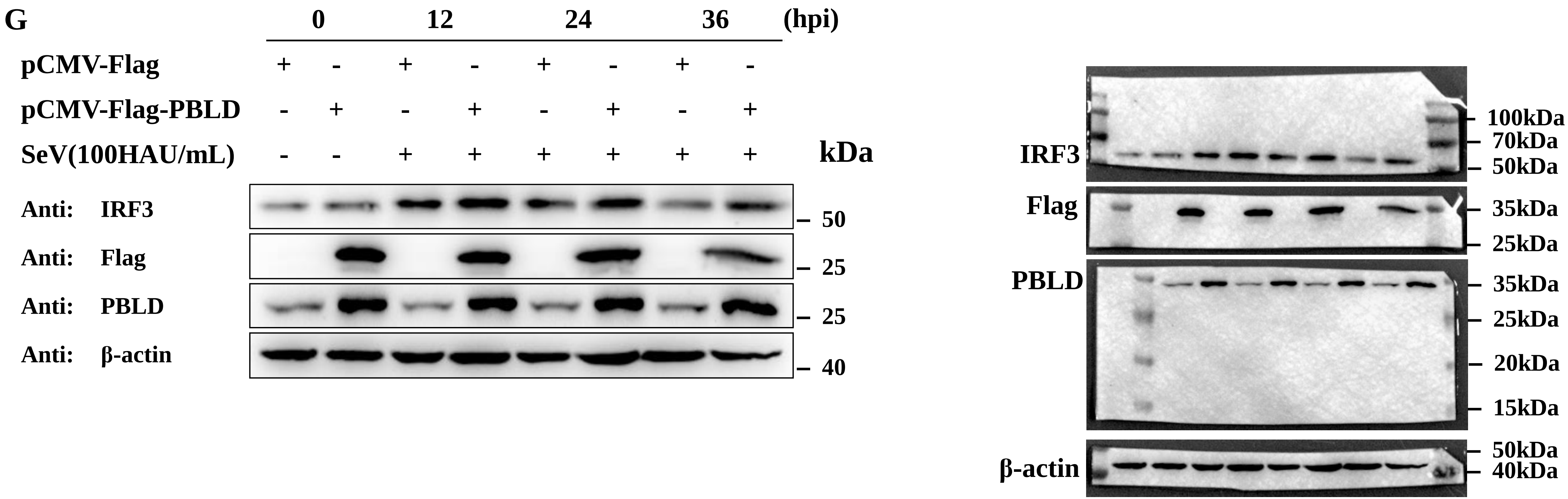

Supplementary Figure 1

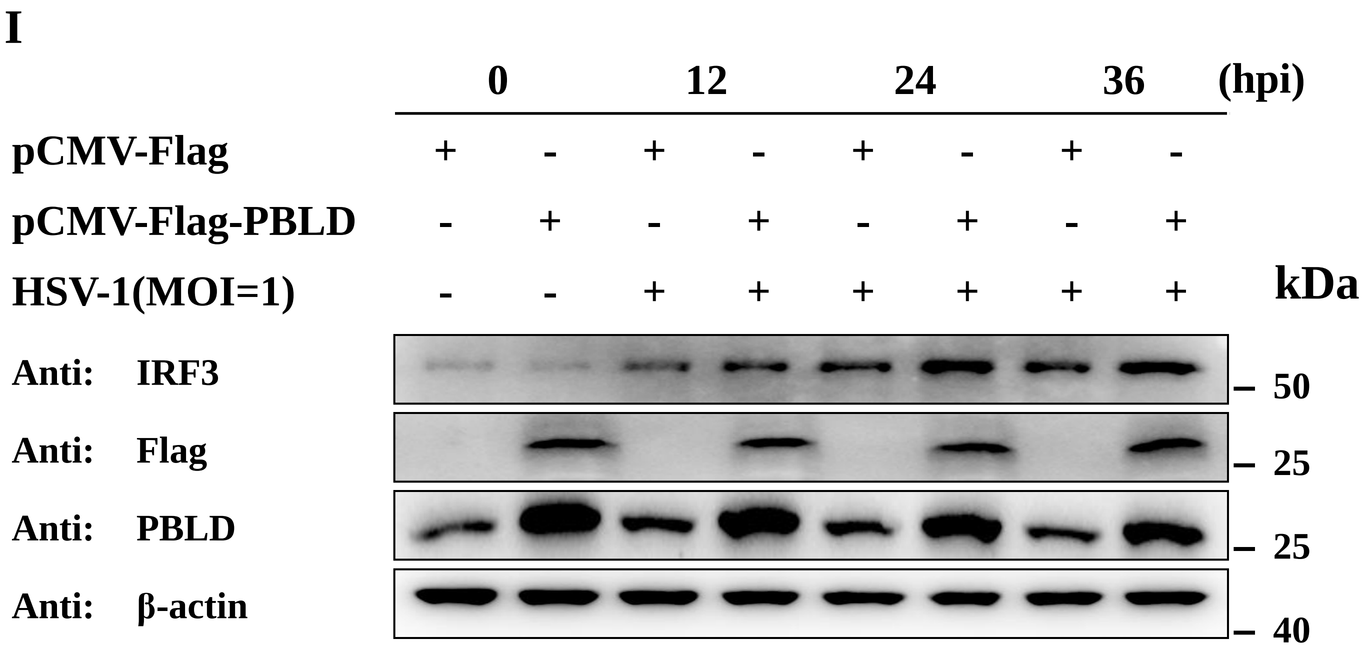

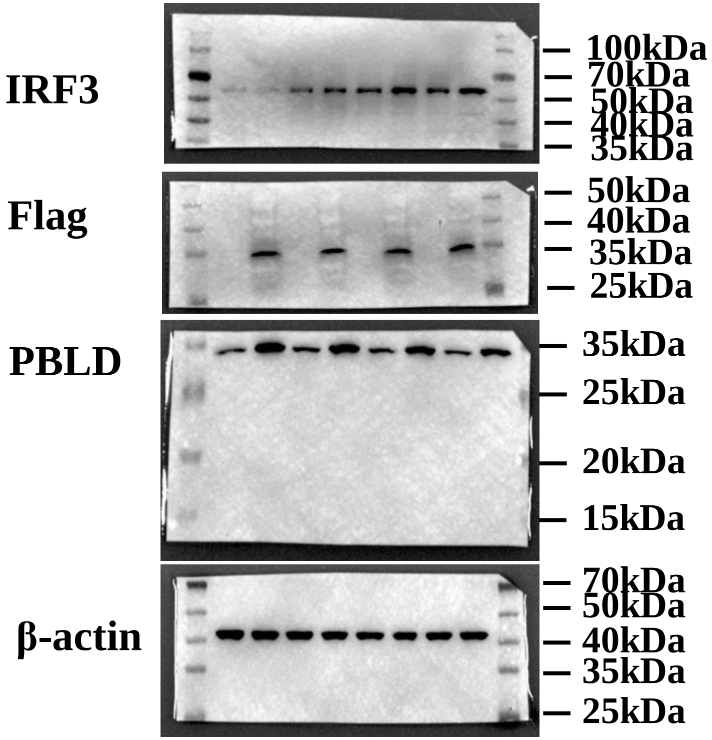

Supplementary Figure 1

K

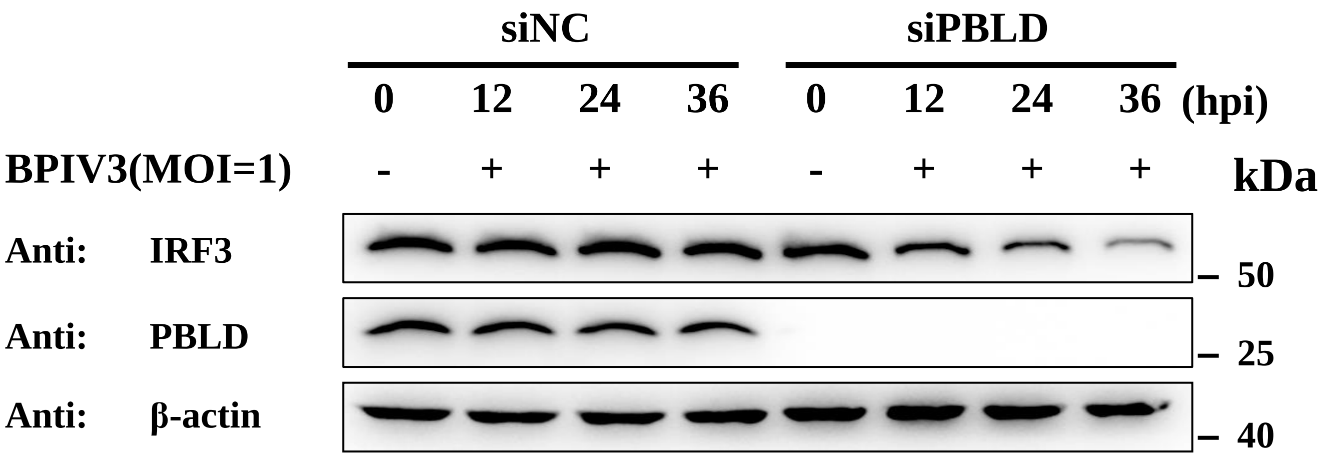

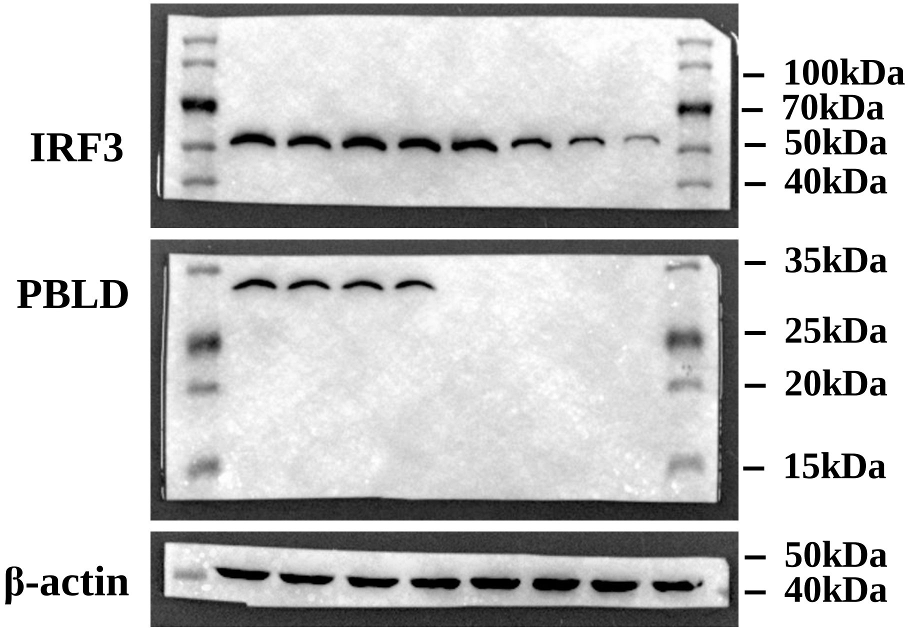

Supplementary Figure 1

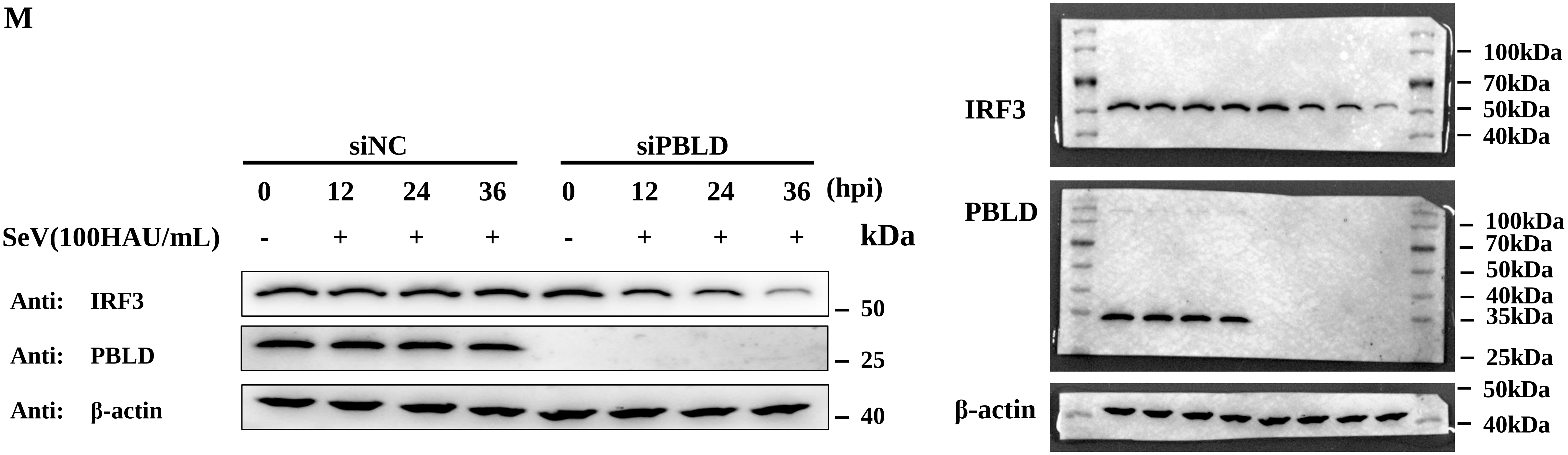

Supplementary Figure 1

O

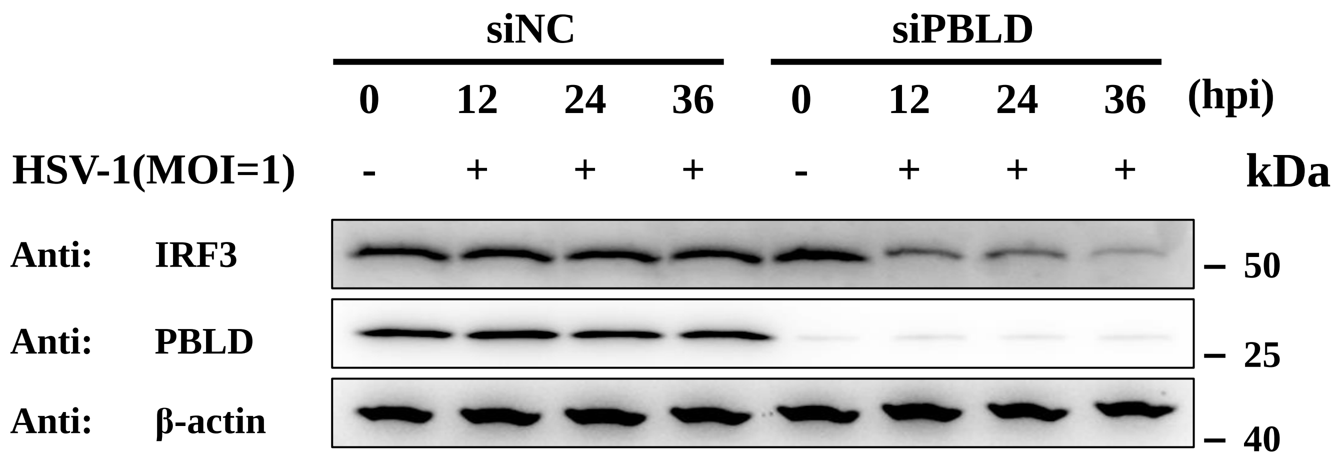

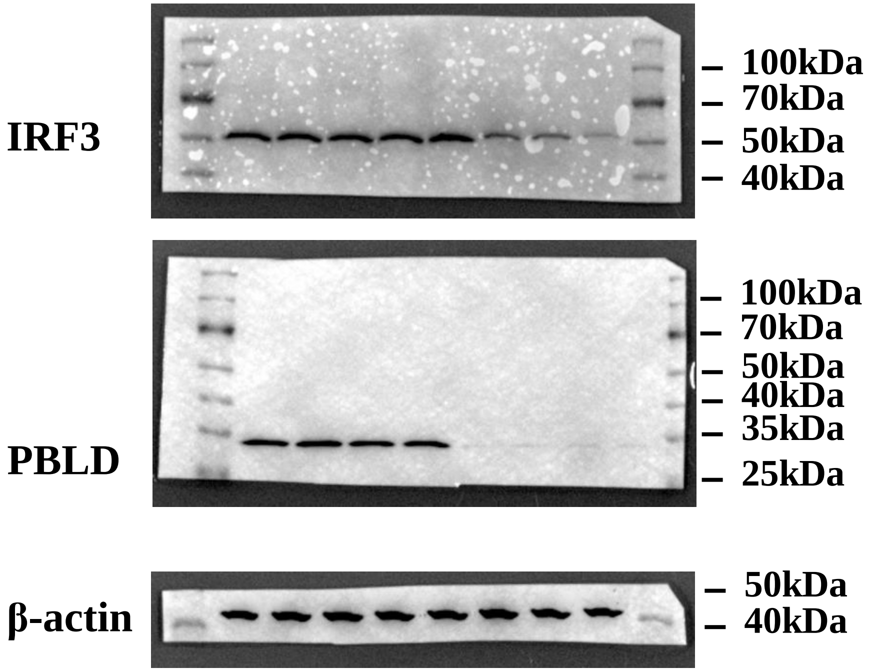

Supplementary Figure 2

Supplementary Figure 2

E

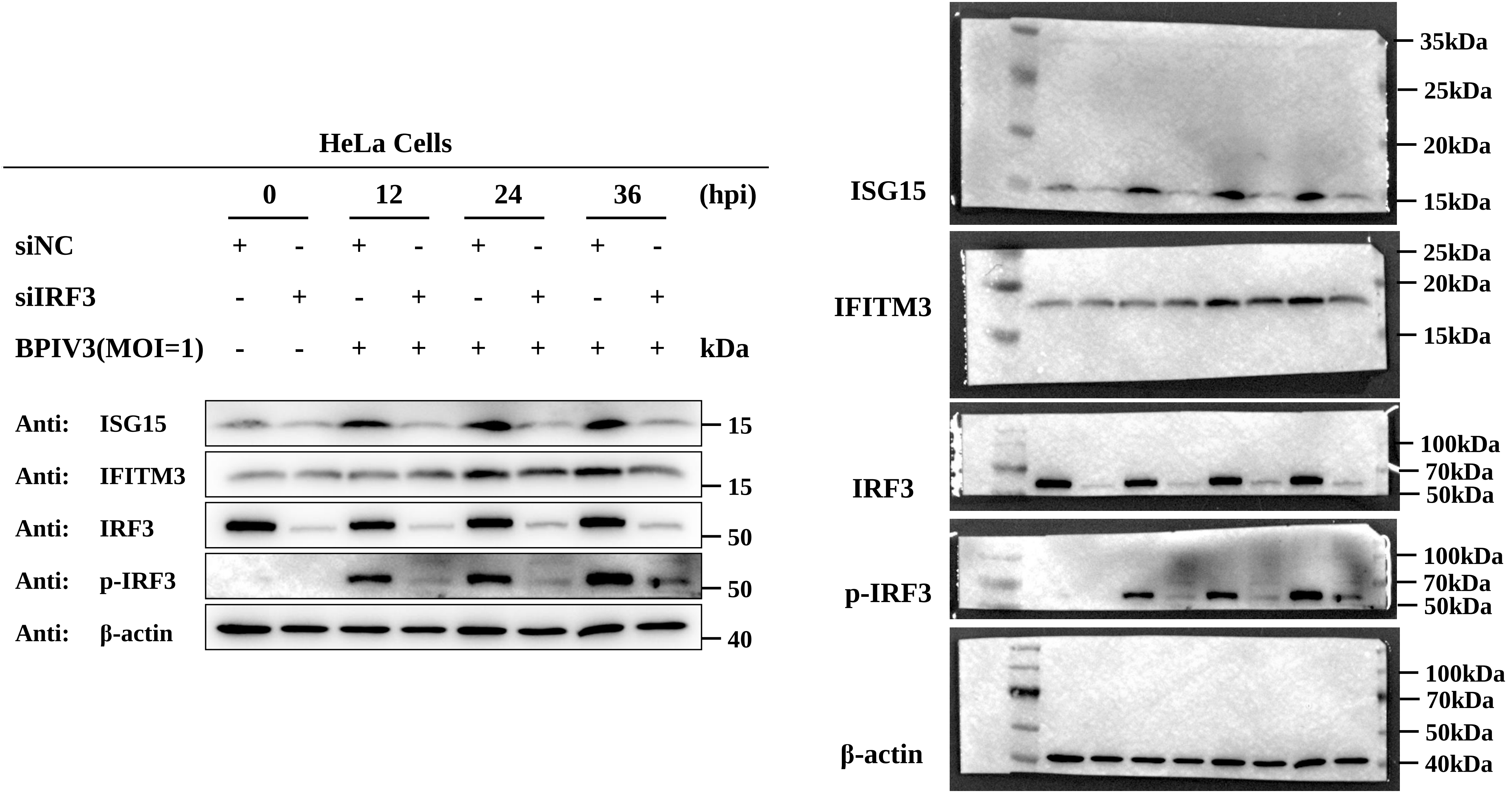

Supplementary Figure 2

F

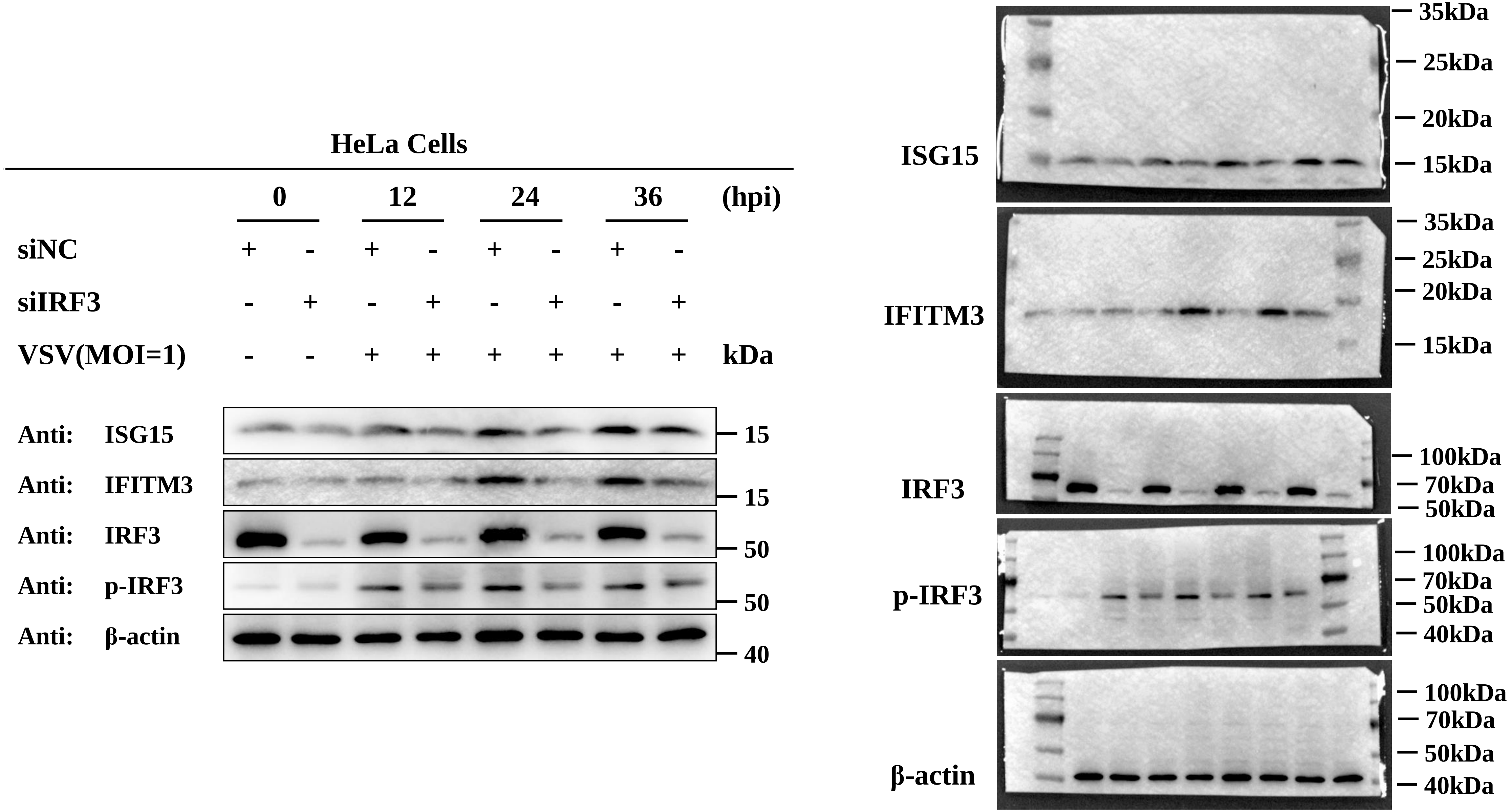

Supplementary Figure 2

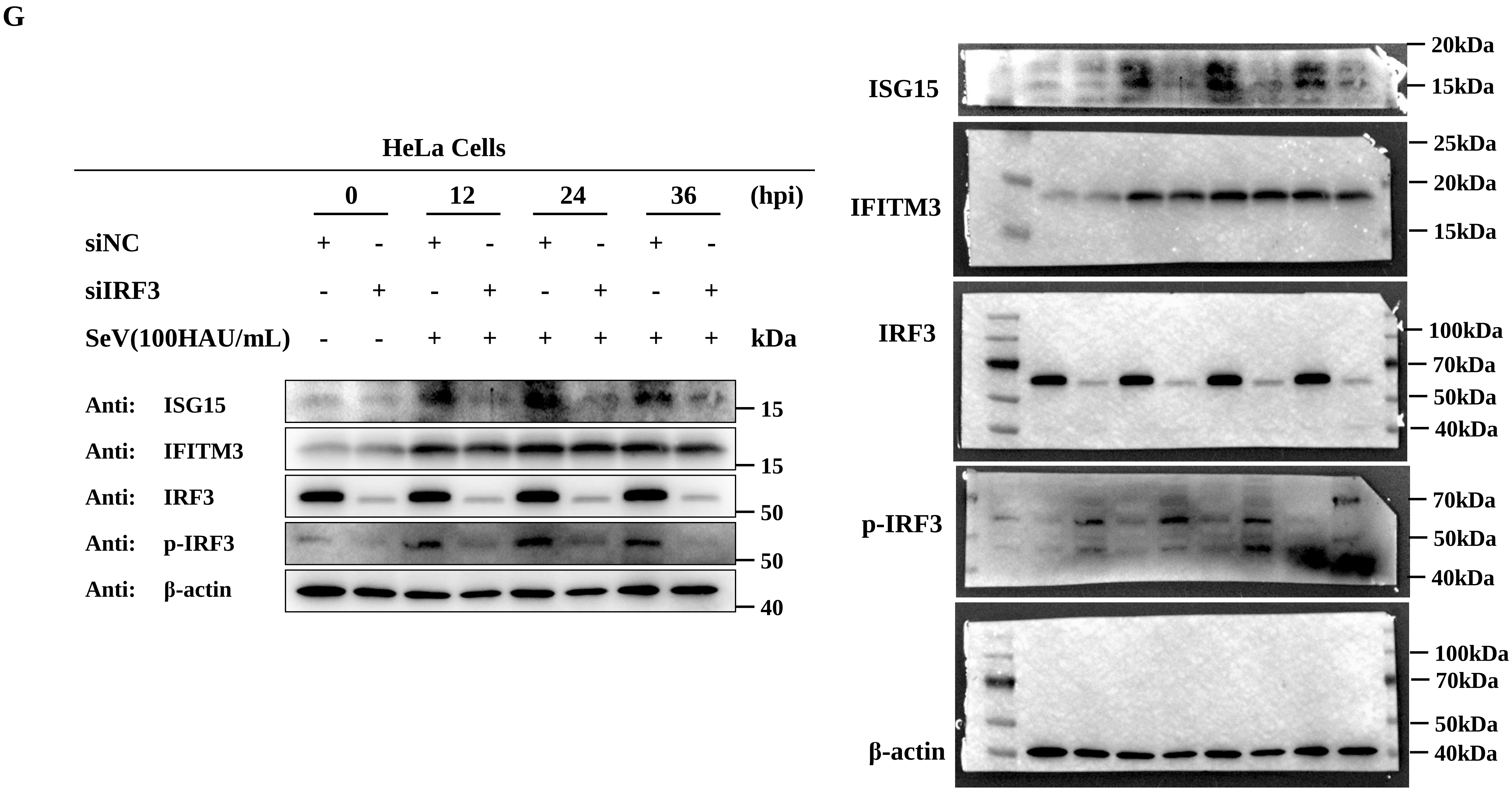

Supplementary Figure 2

H

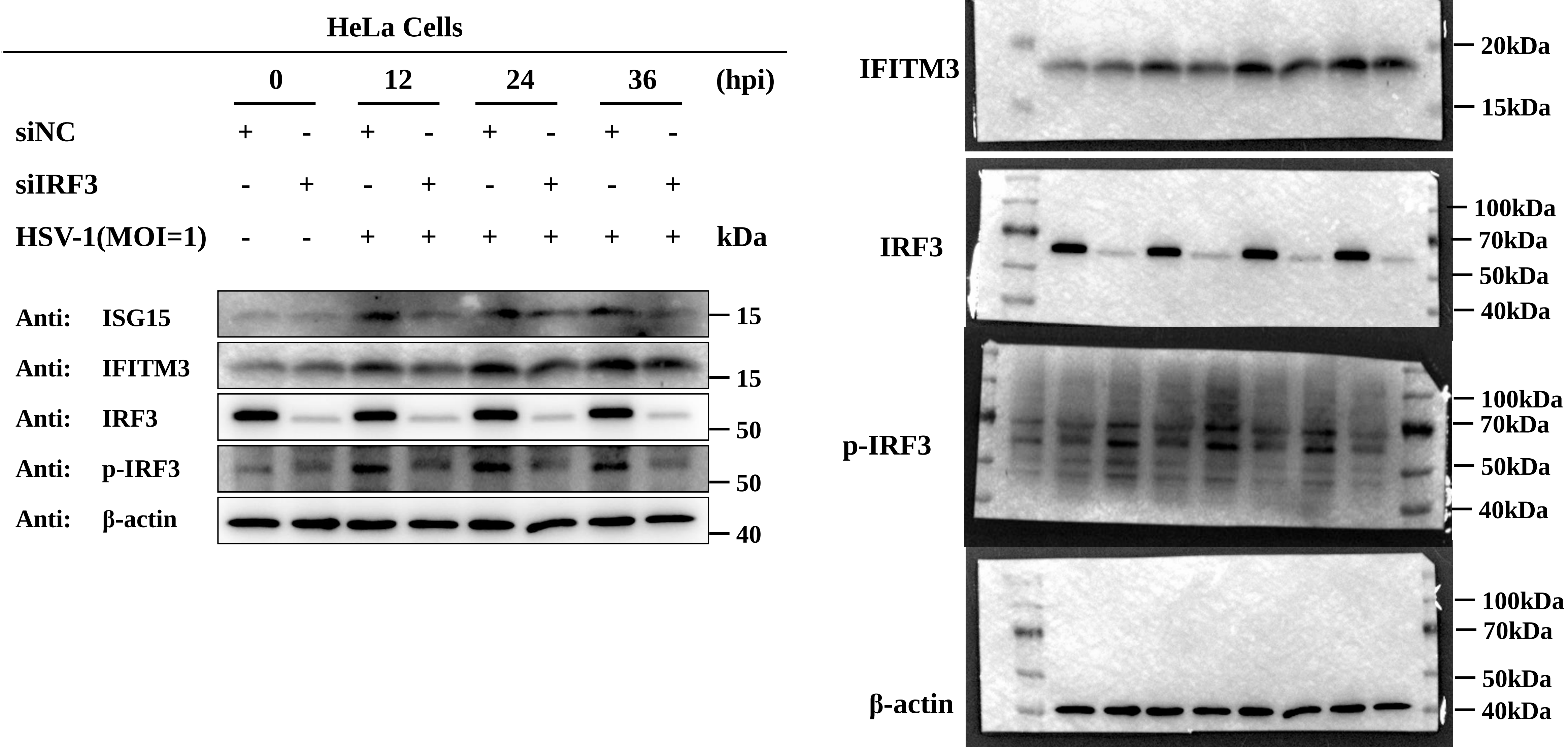

Supplementary Figure 3

Supplementary Figure 3

A

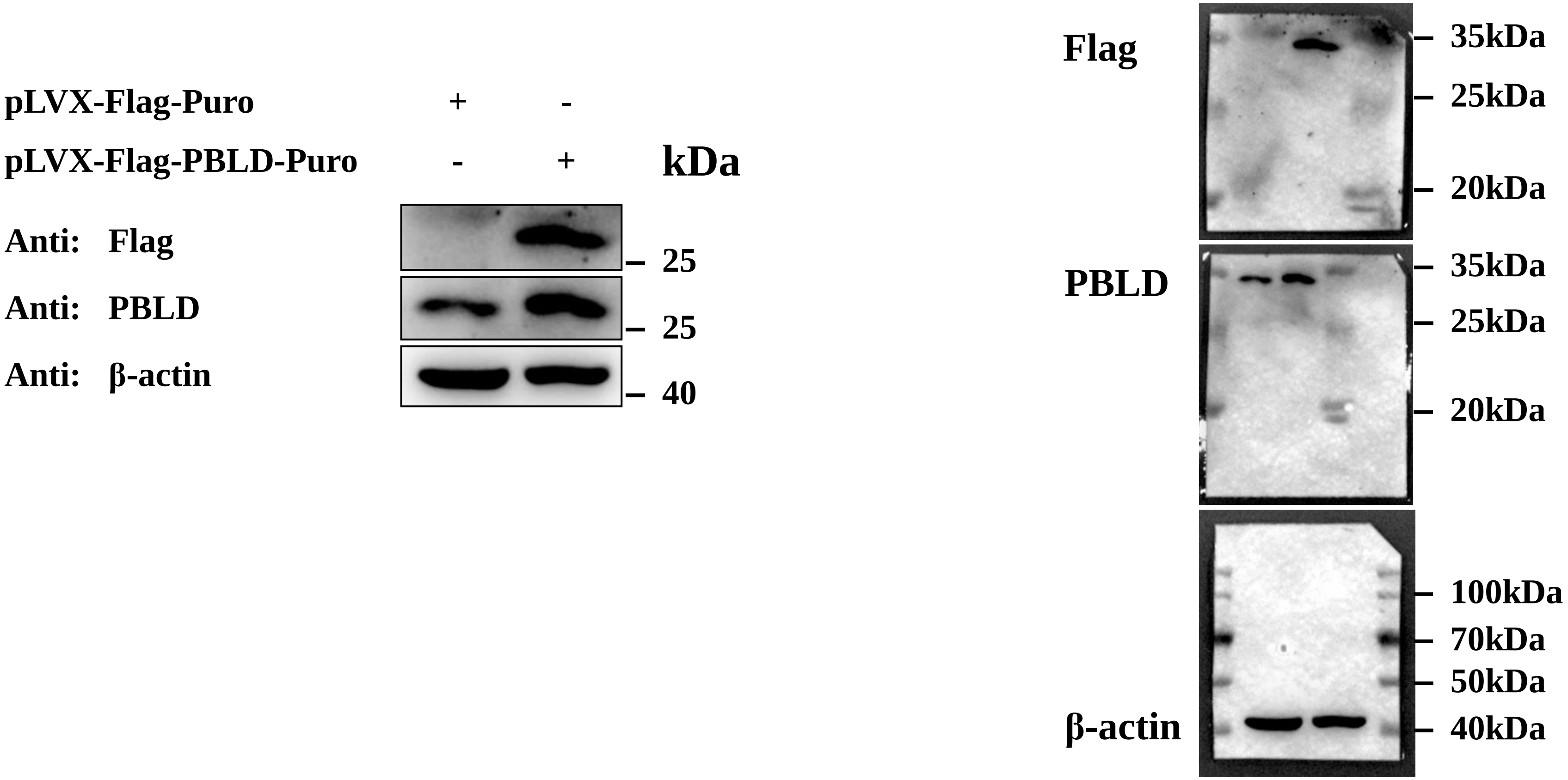

Supplementary Figure 3

E

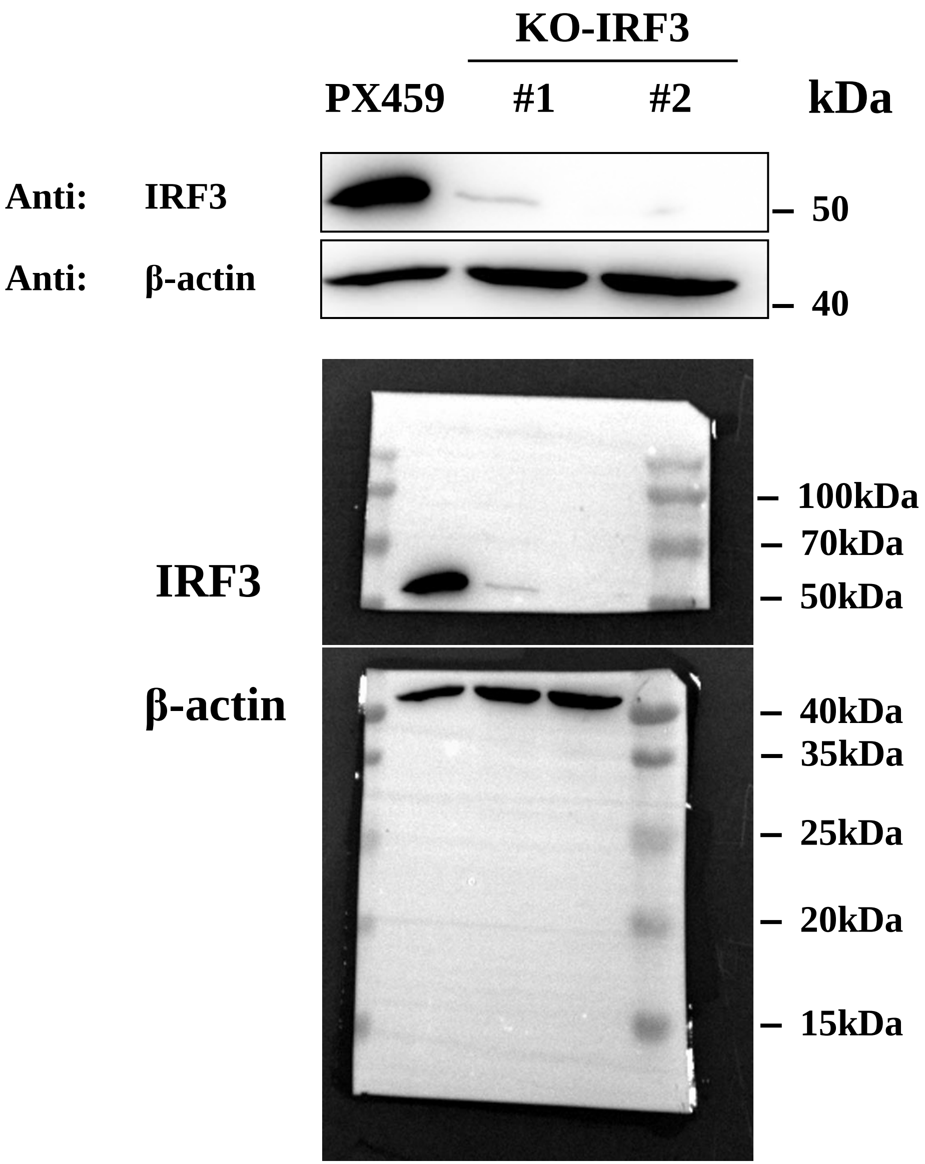

Supplementary Figure 4

Supplementary Figure 4

A

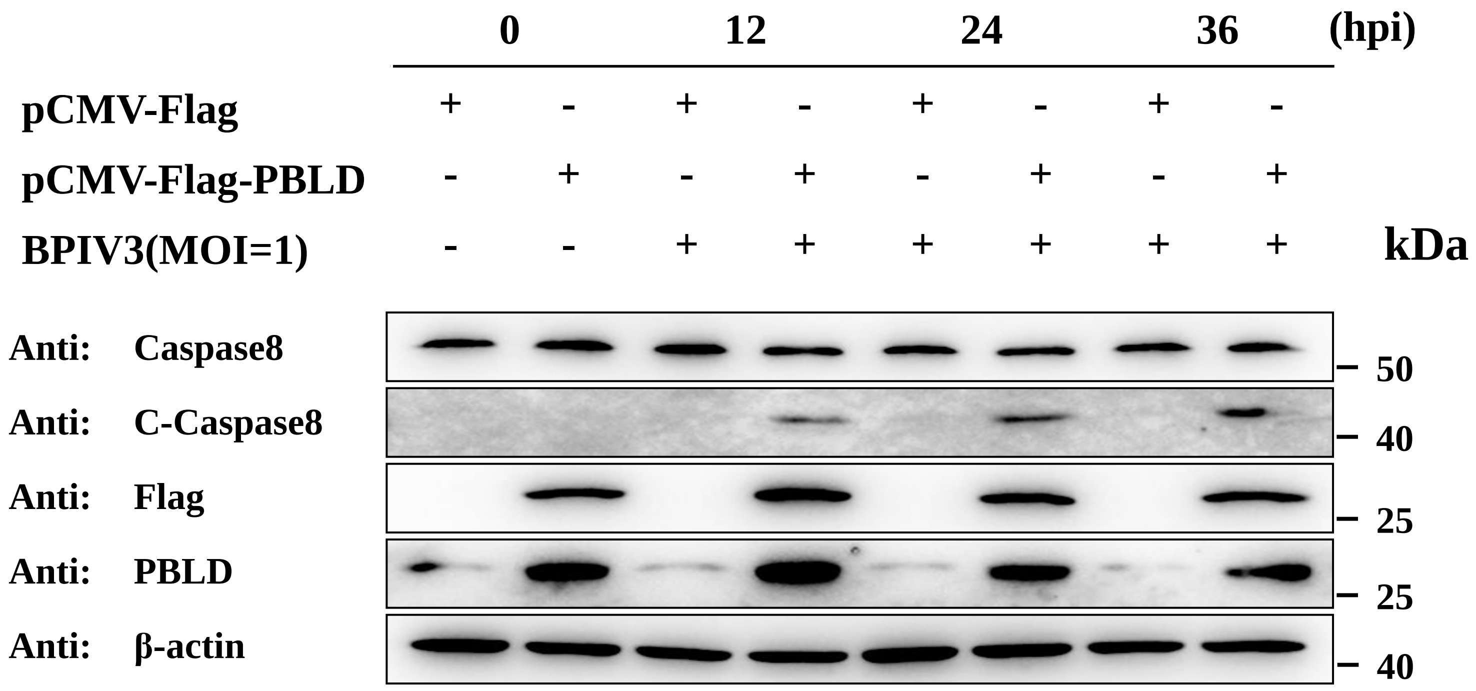

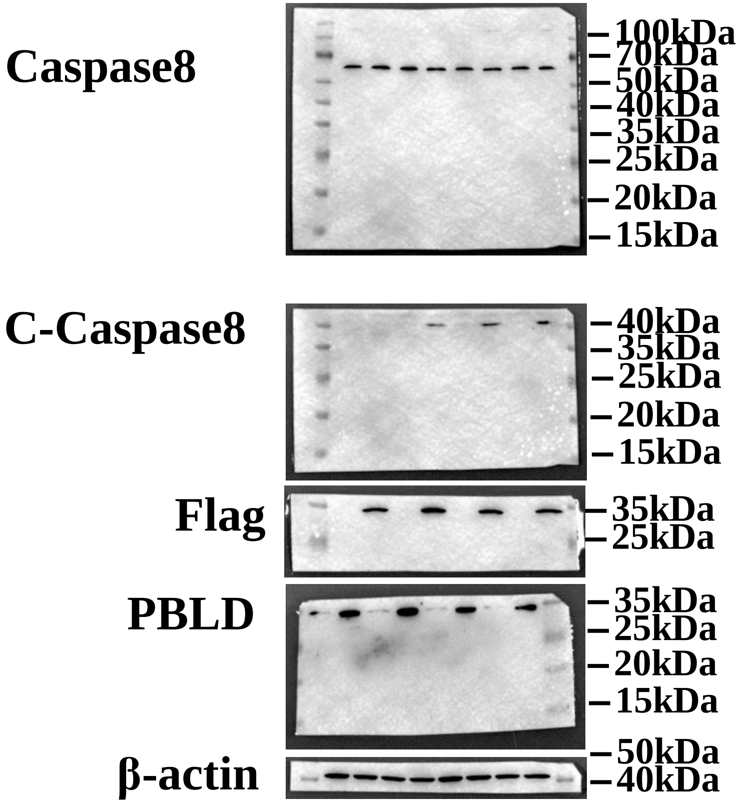

Supplementary Figure 4

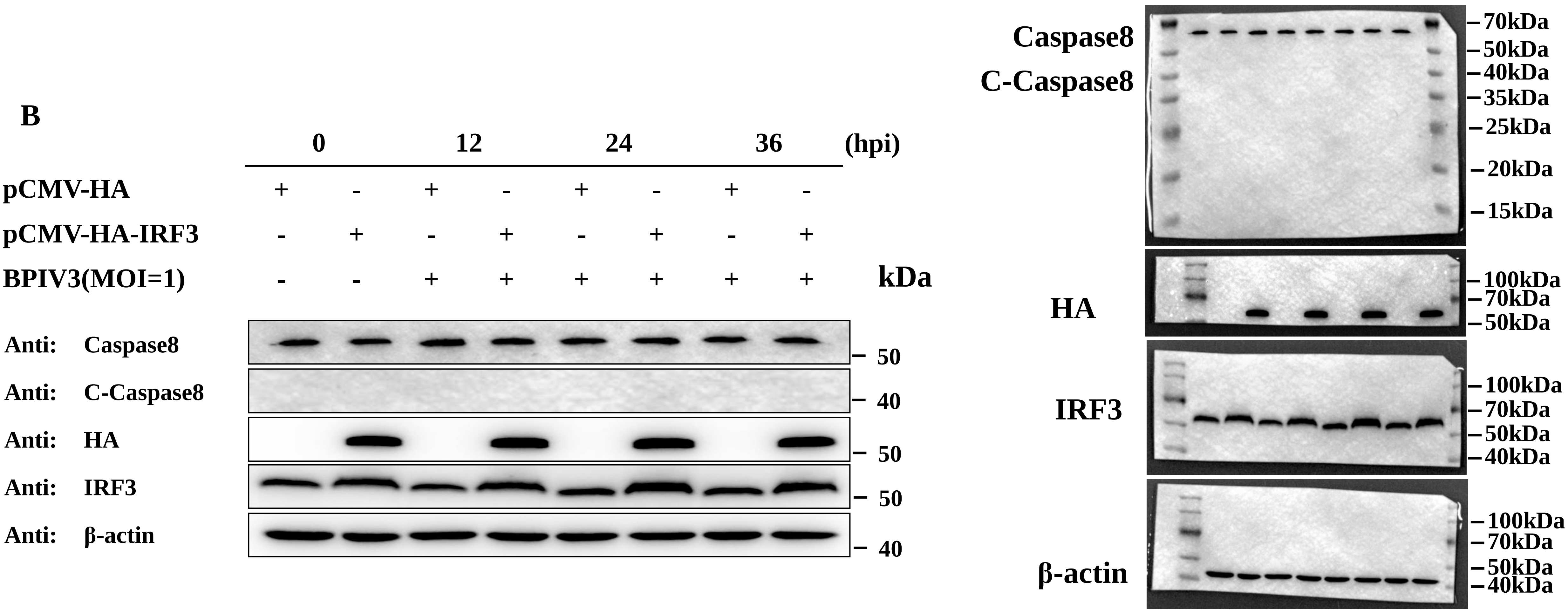

Supplementary Figure 4

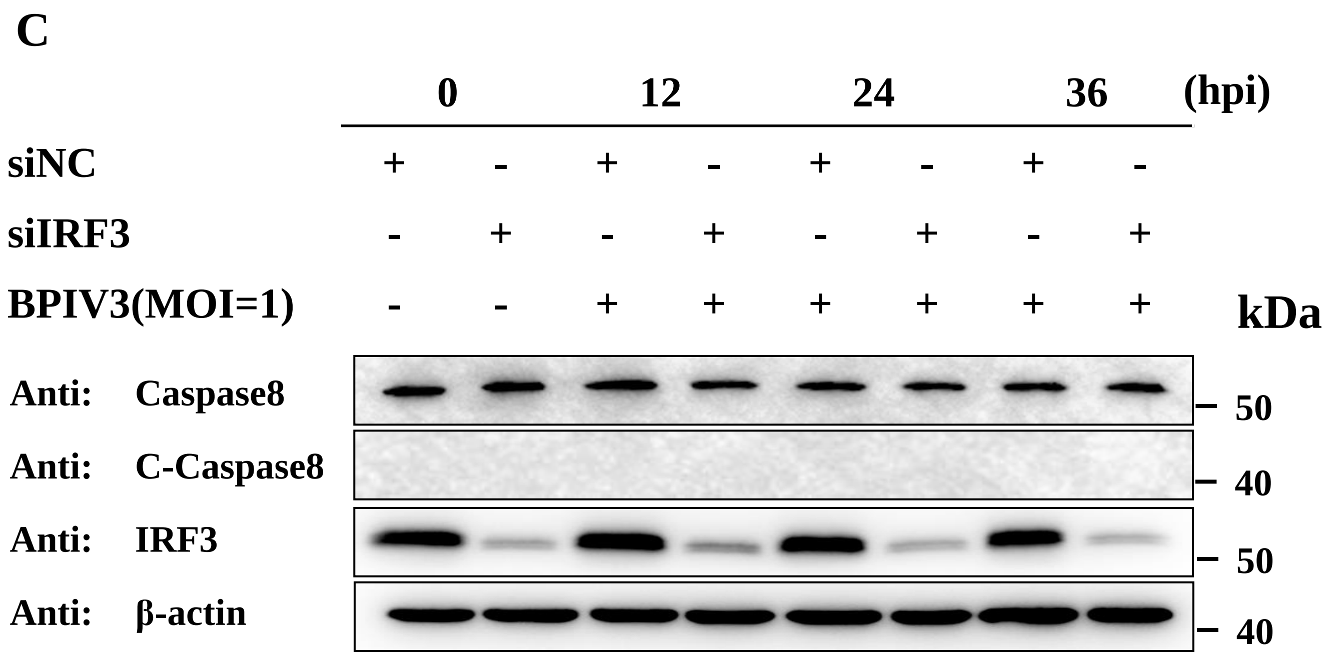

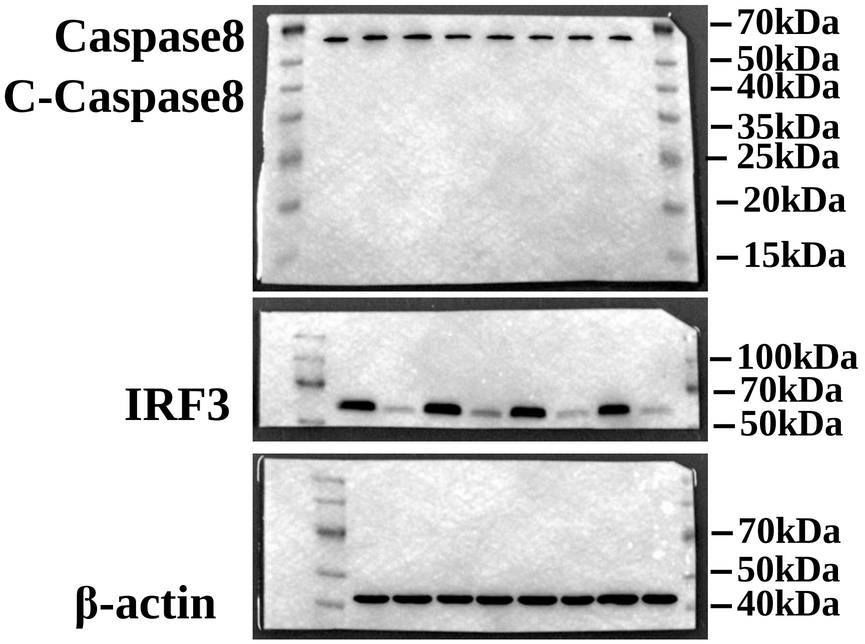

Supplementary Figure 4

D

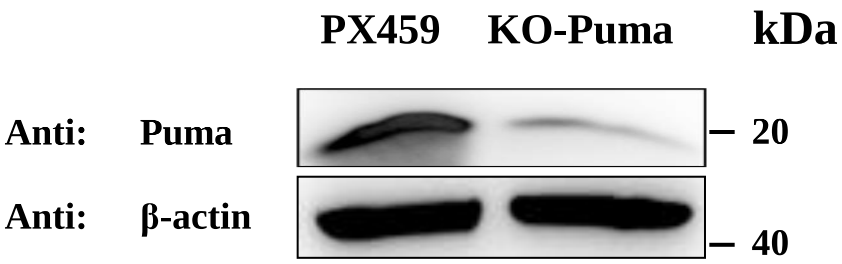

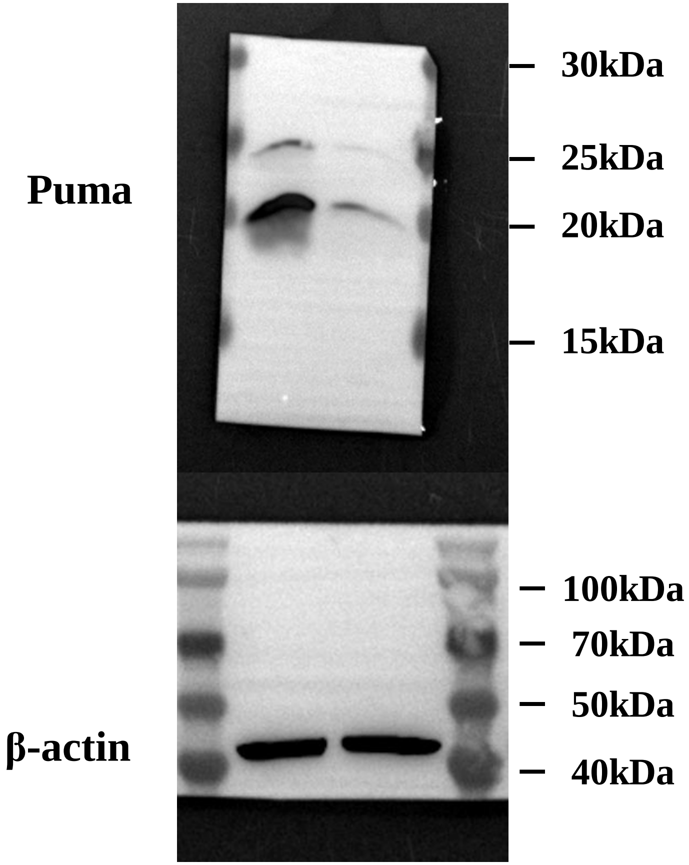

Supplementary Figure 5

Supplementary Figure 5

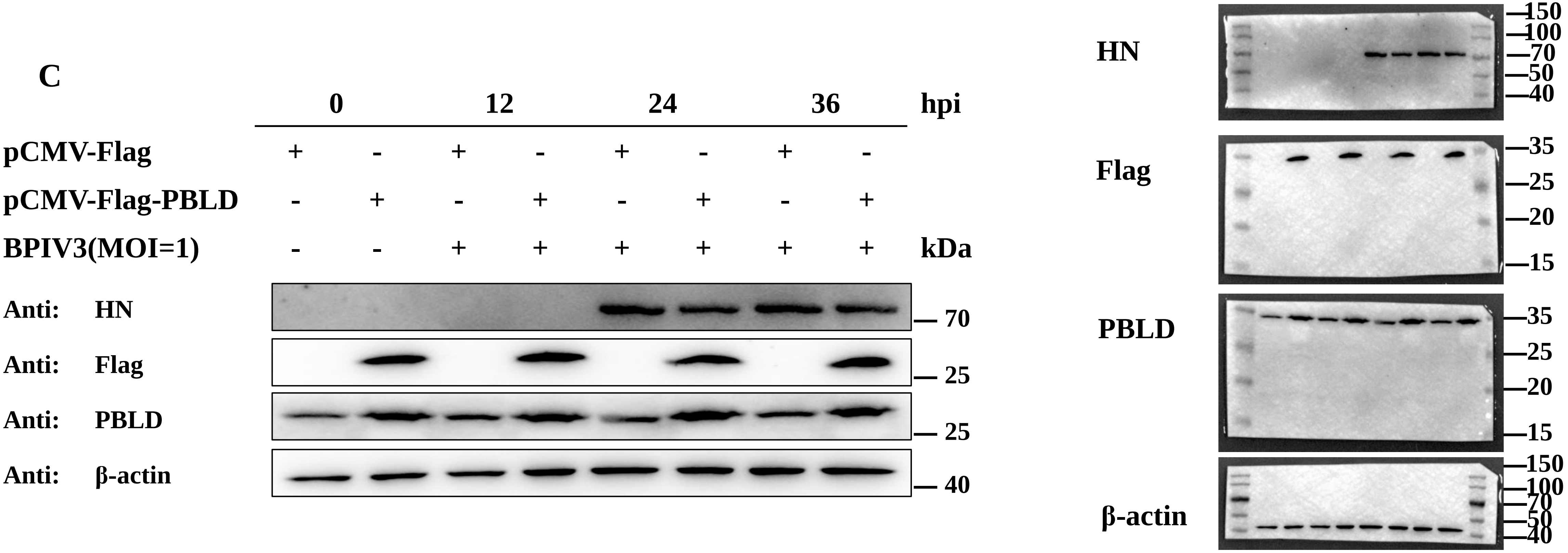

Supplementary Figure 5

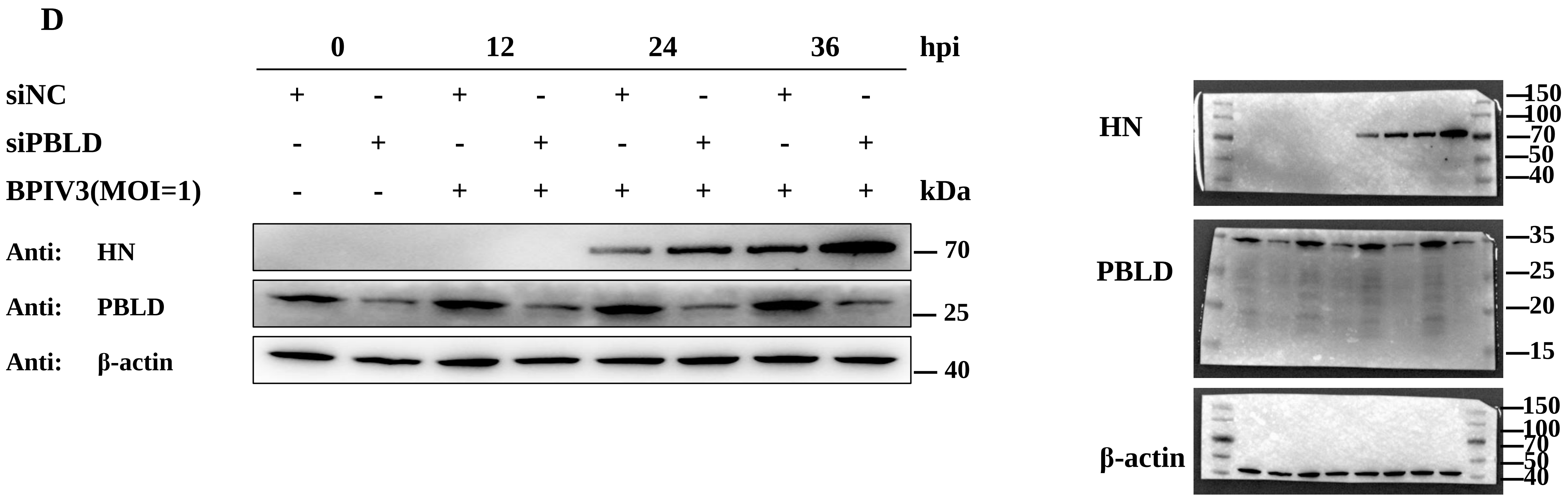

Supplementary Figure 5

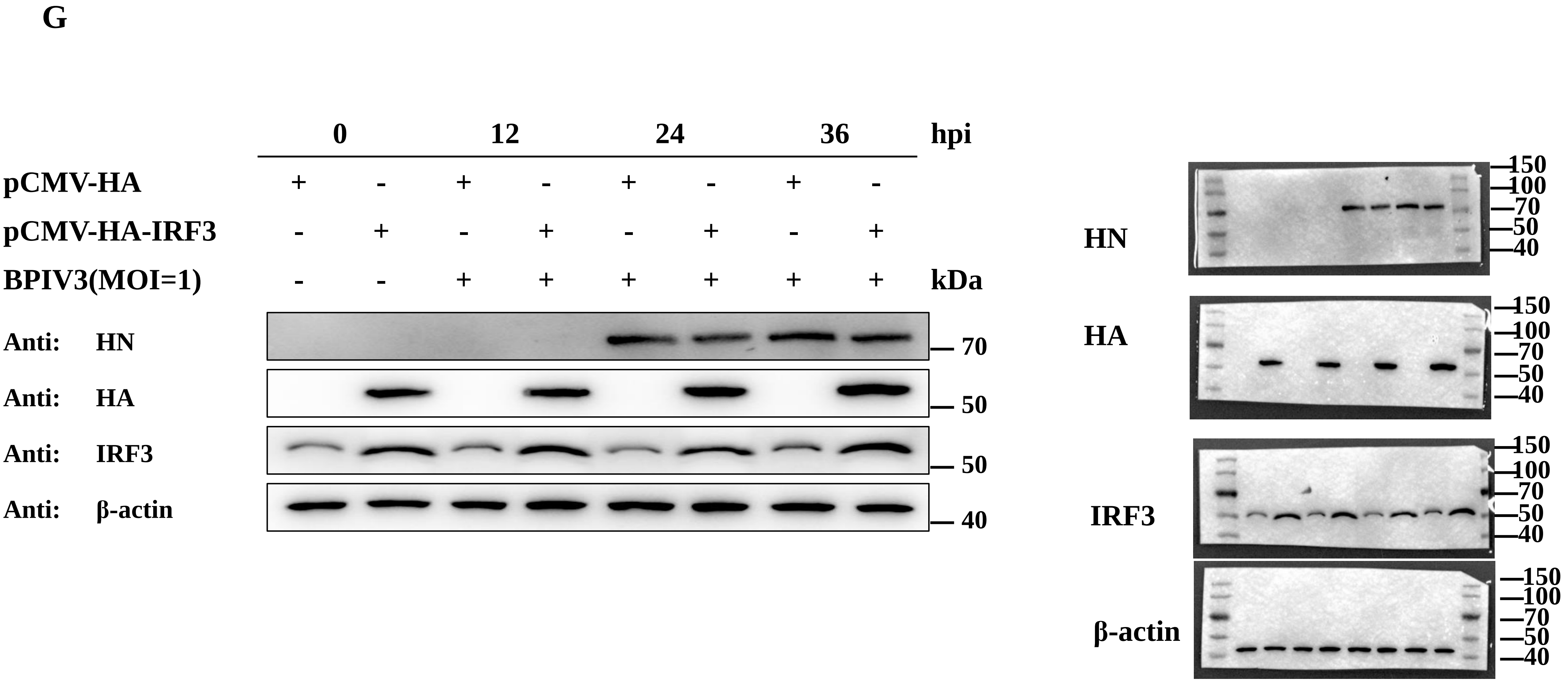

Supplementary Figure 5

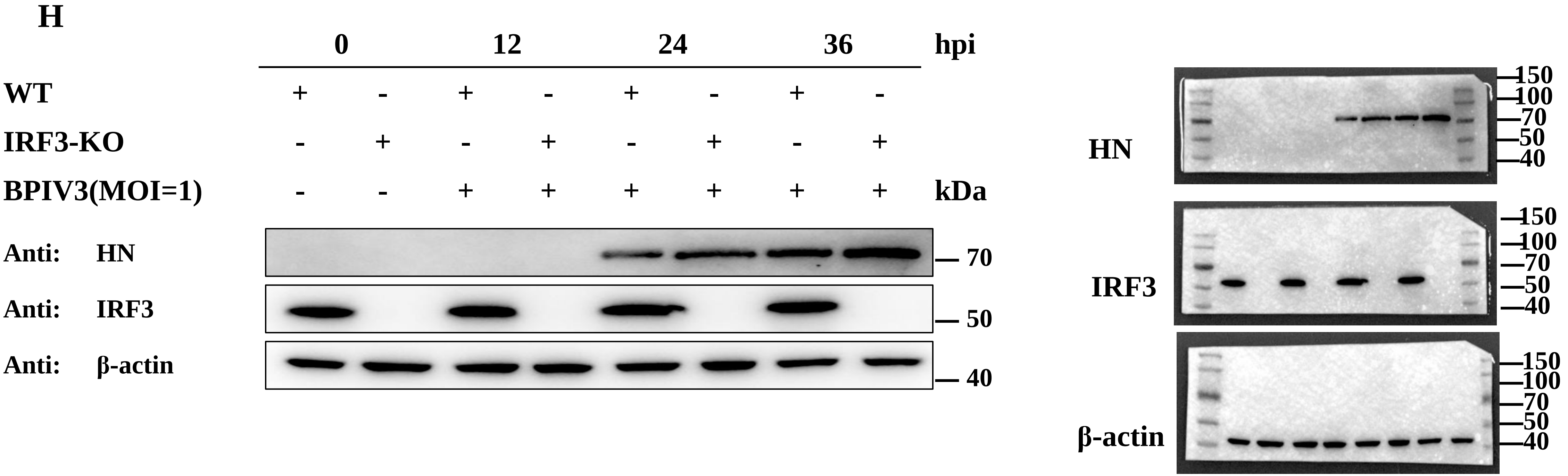

Supplementary Figure 6

Supplementary Figure 6

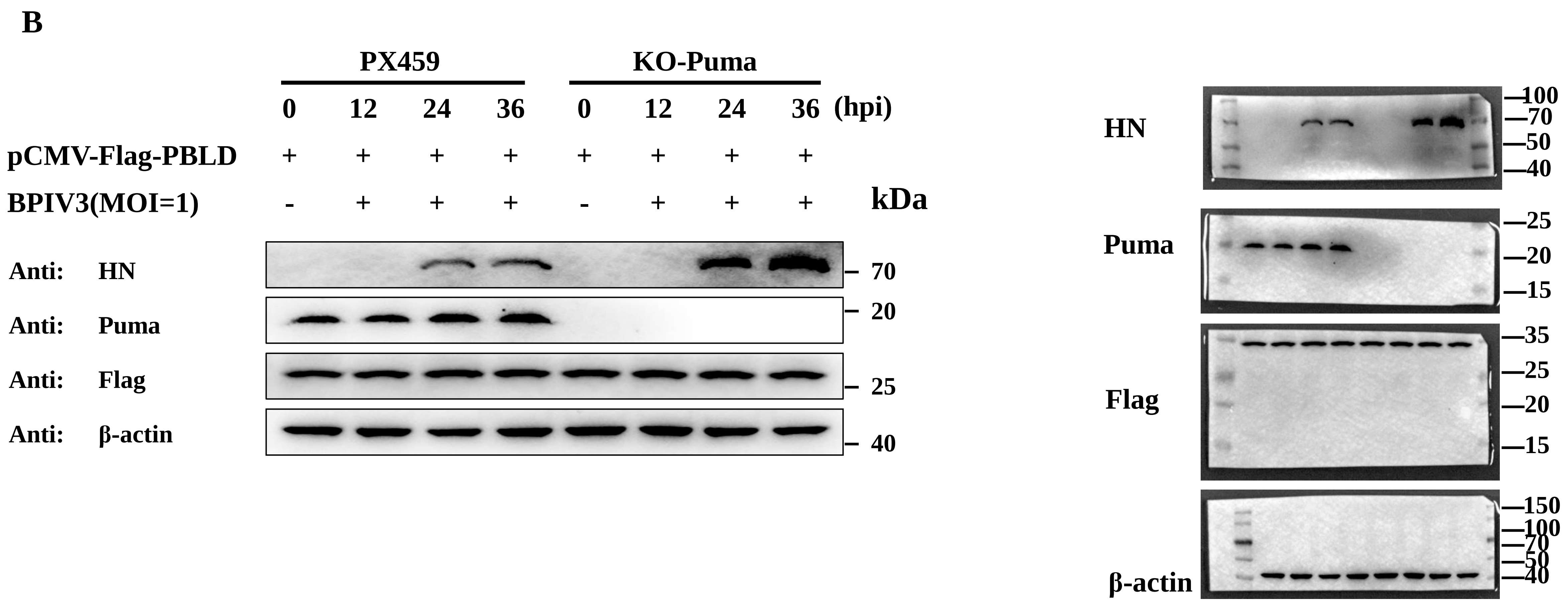

Supplementary Figure 6

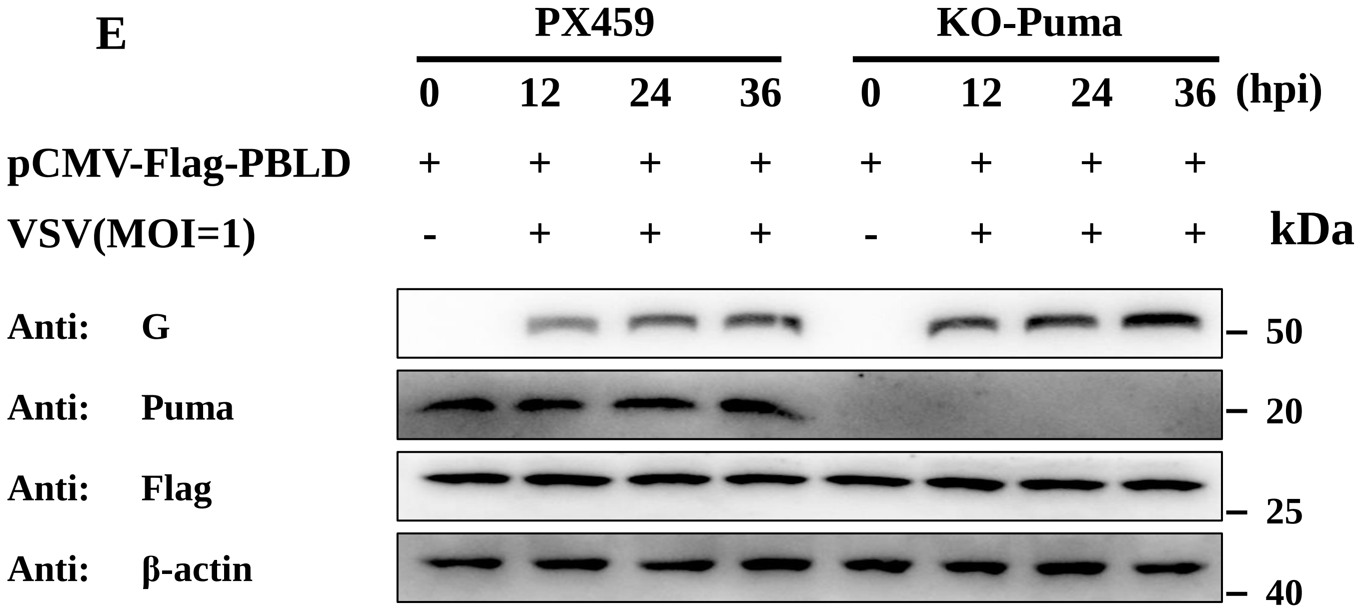

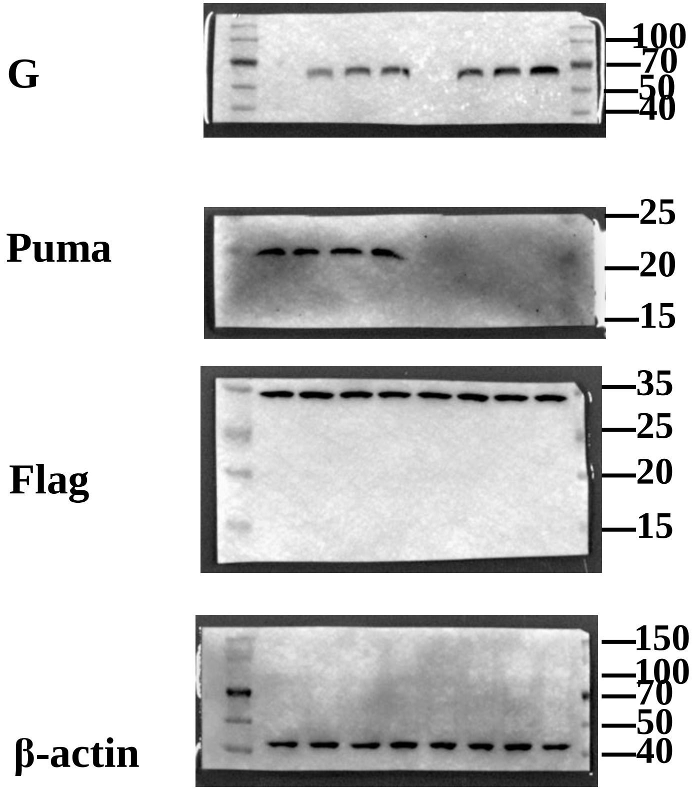

Supplementary Figure 6

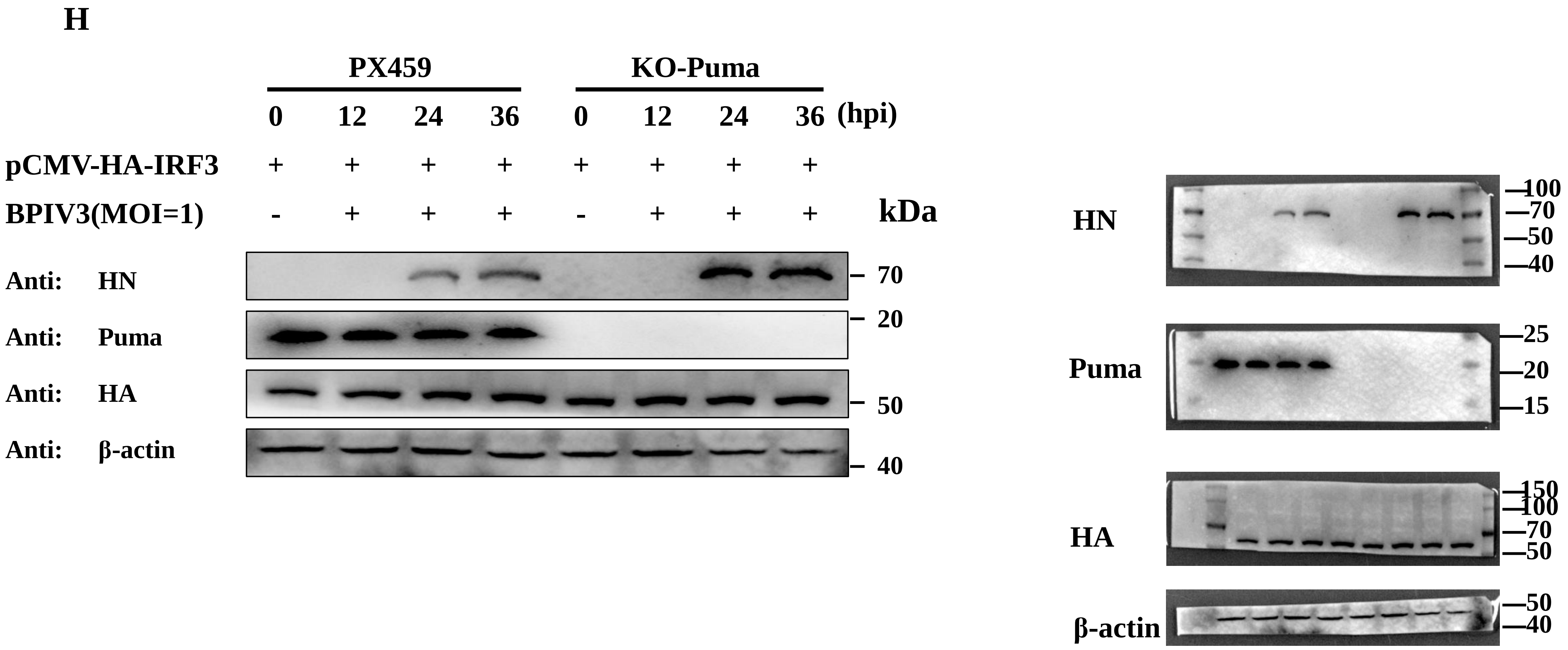

Supplementary Figure 6

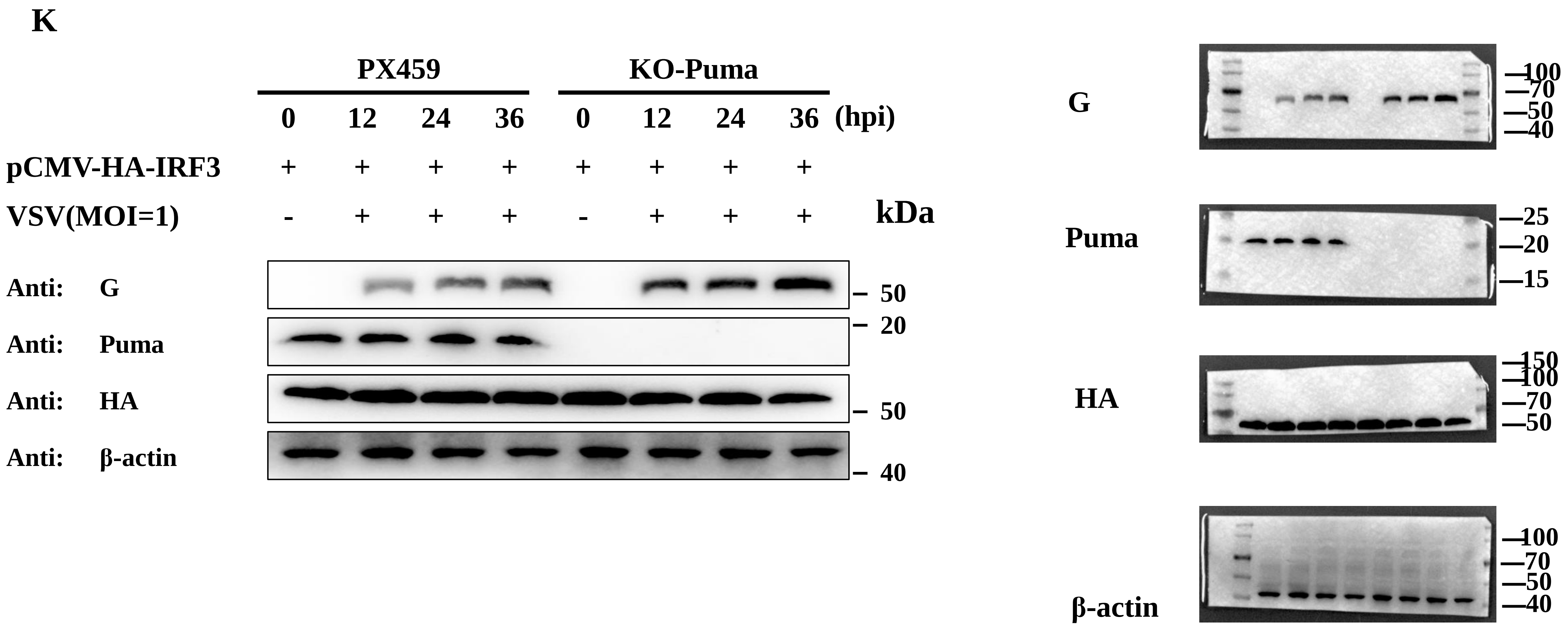

Supplementary Figure 7

Supplementary Figure 7

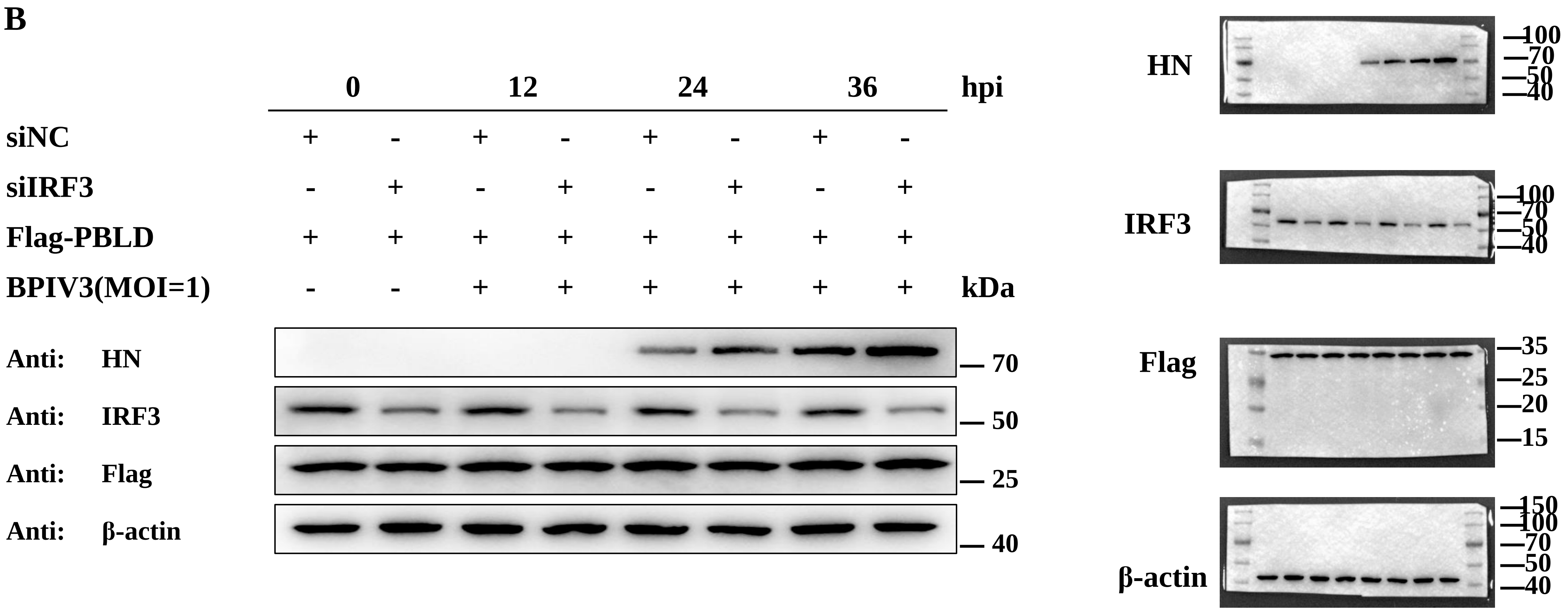

Supplementary Figure 7

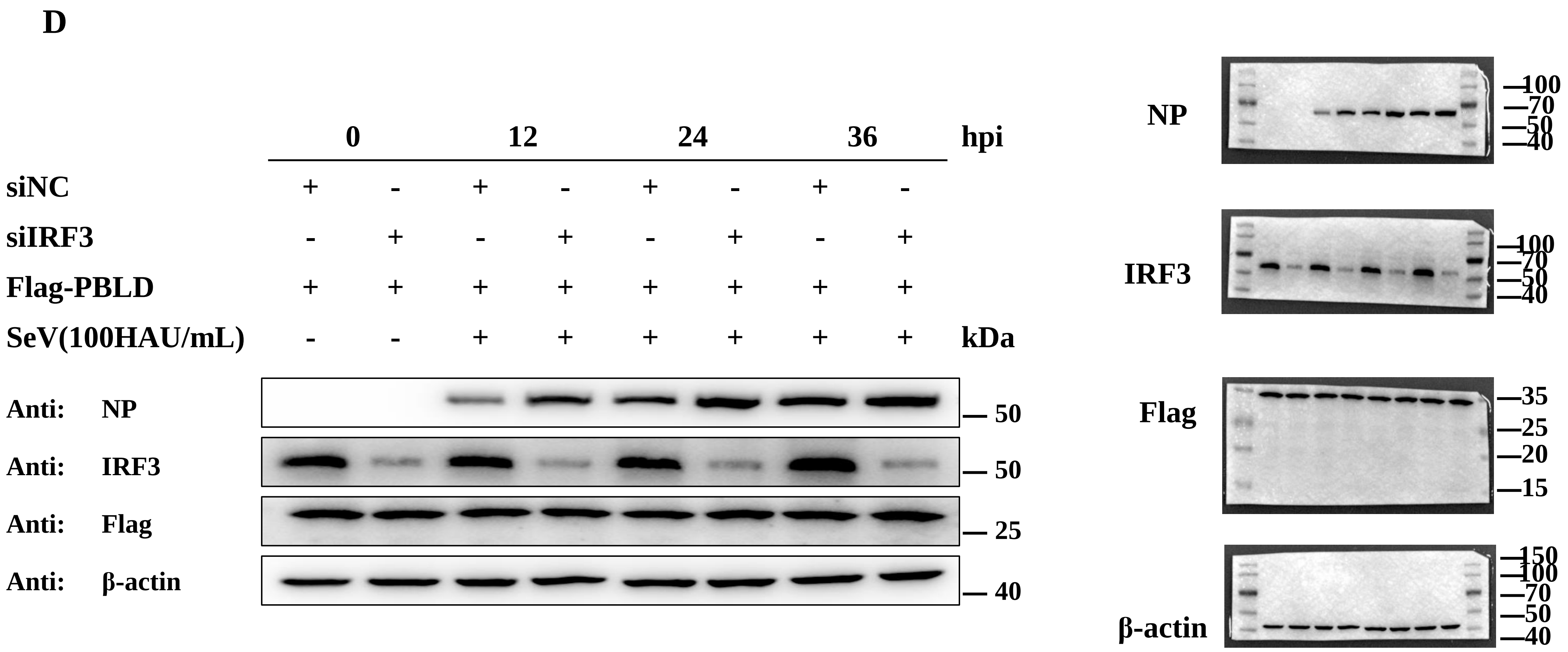

Supplementary Figure 8

Supplementary Figure 8

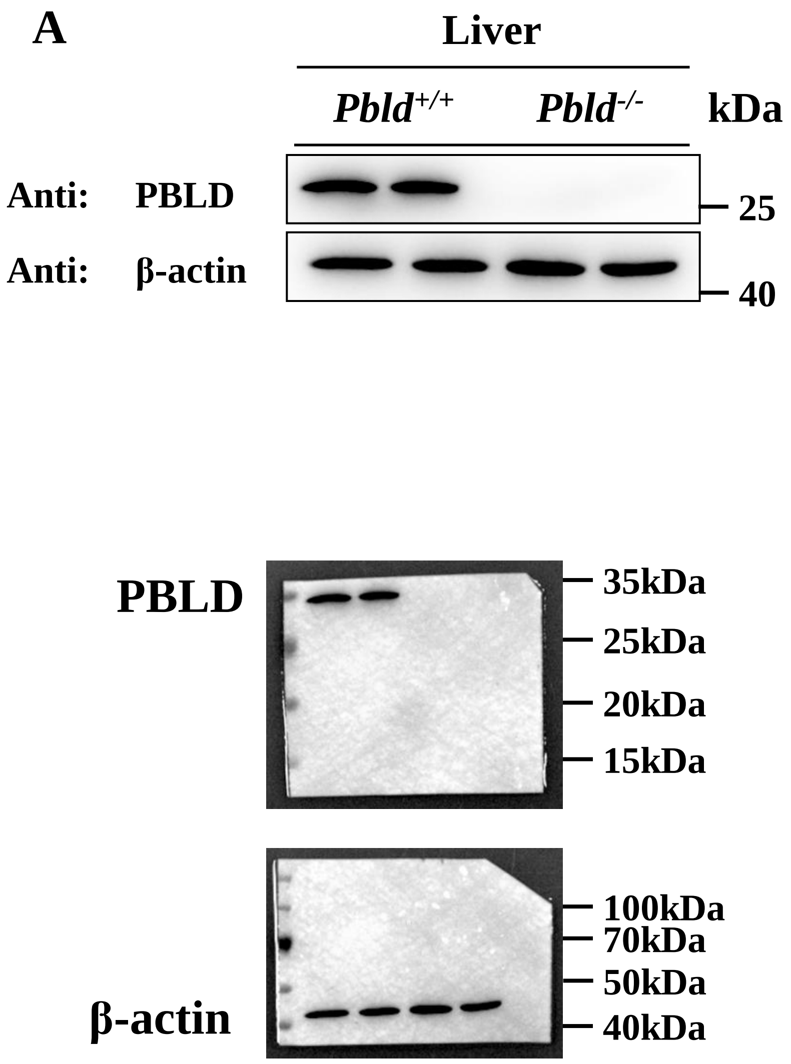

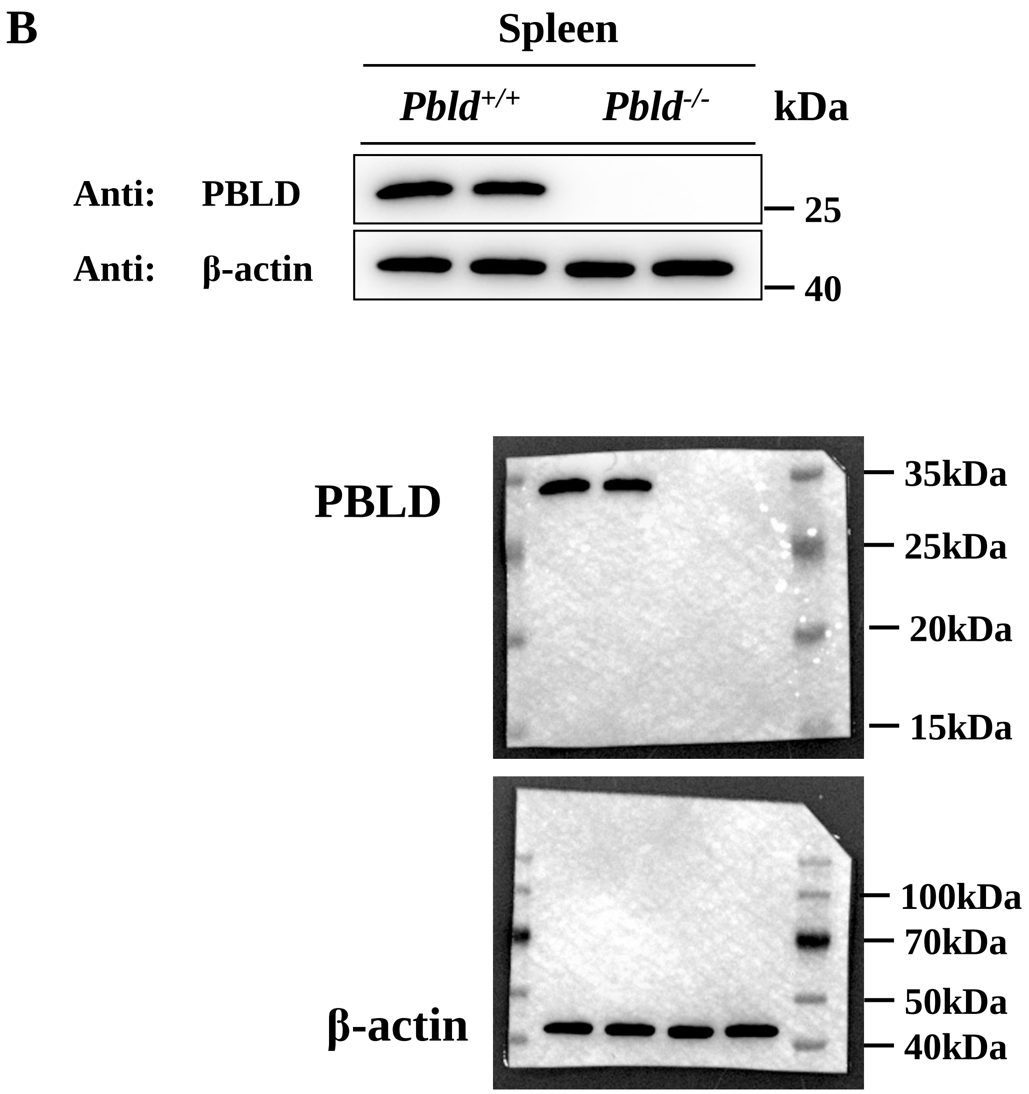

Supplementary Figure 8

C

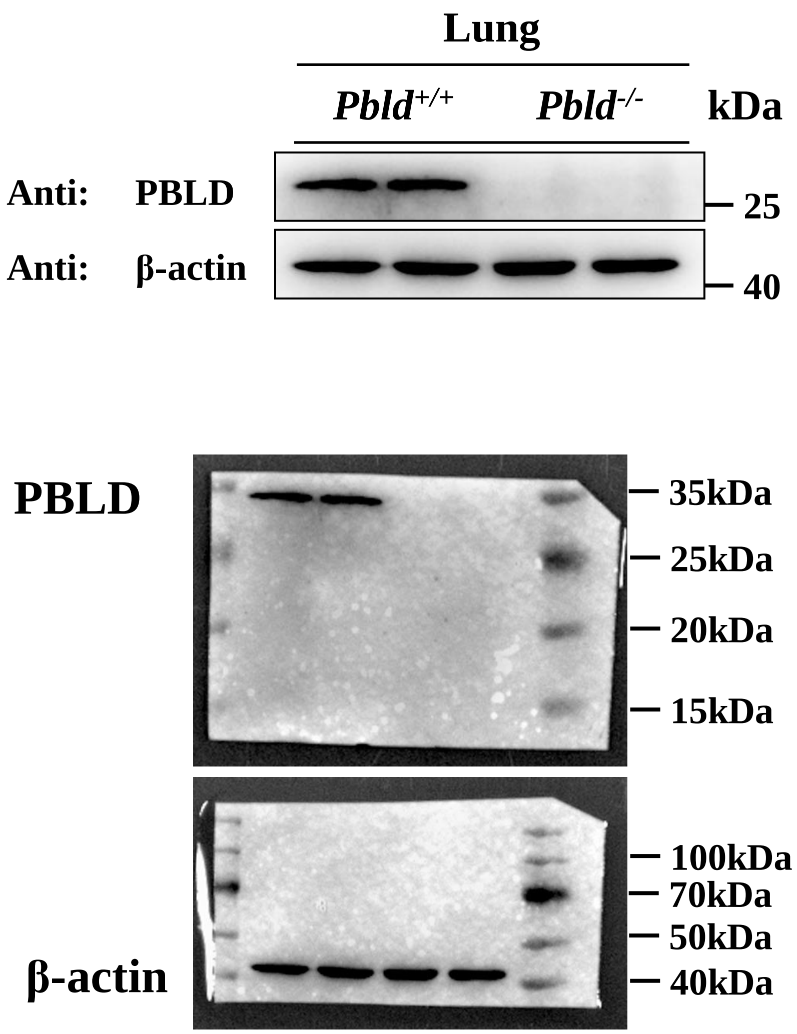

D

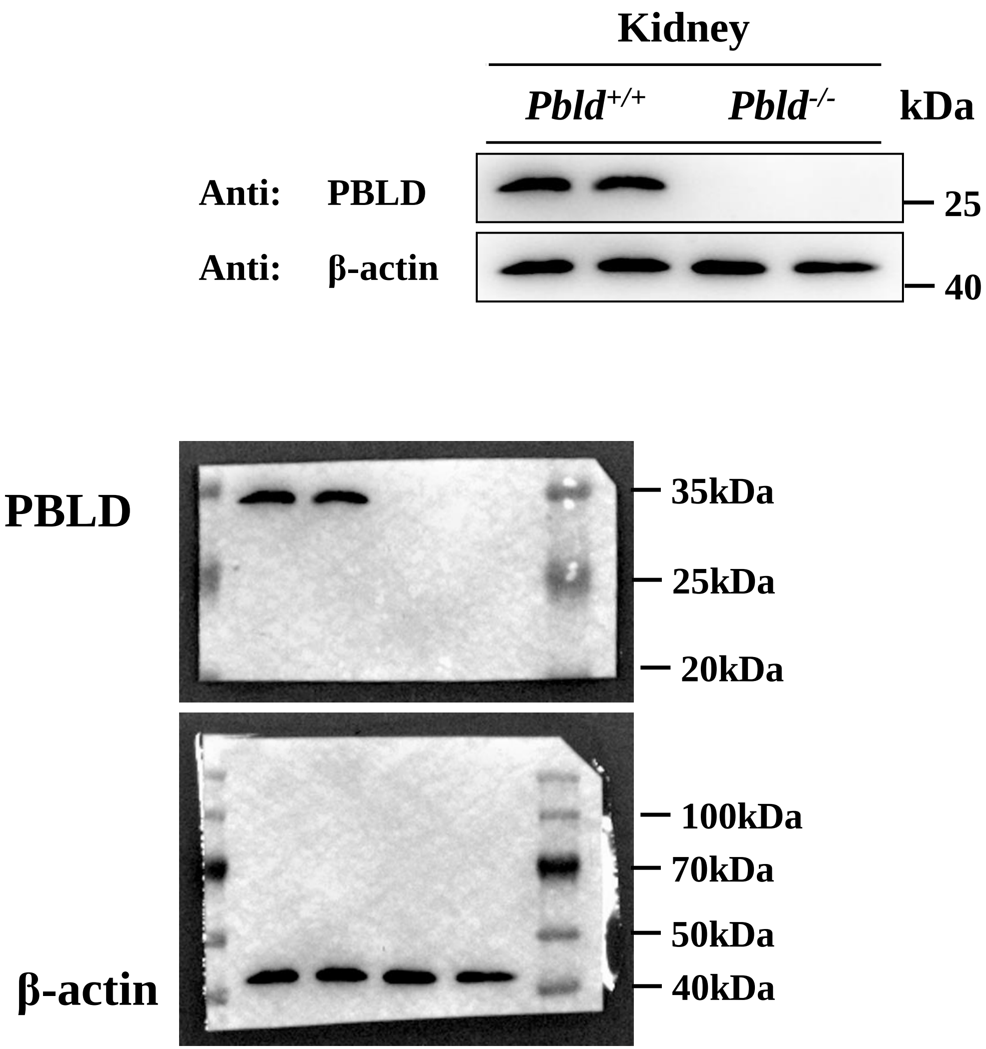

Supplementary Figure 9

Supplementary Figure 9

A

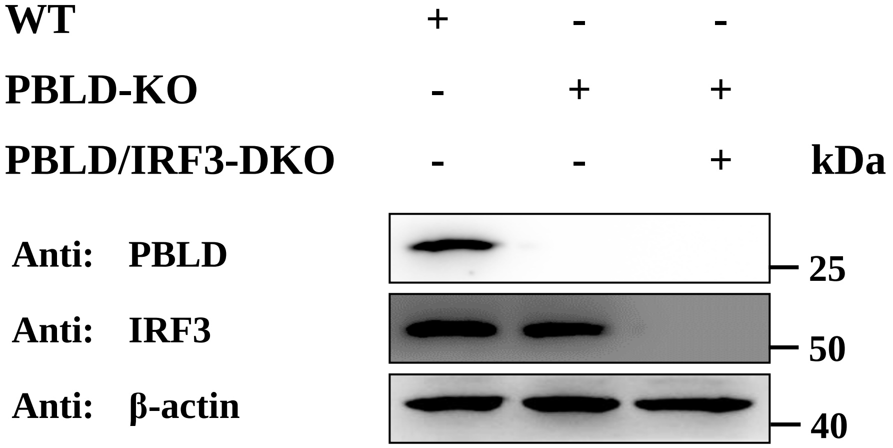

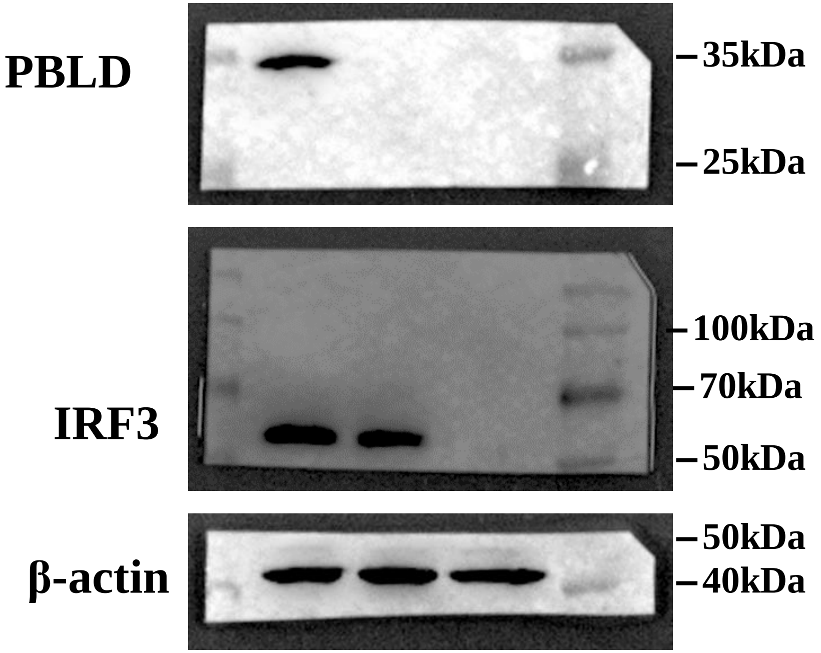

Supplement: Supplementary file 11 — original data [file 41419_2024_7083_MOESM11_ESM.pdf]
