# Supplementary material for: PBLD promotes IRF3 mediated the type I interferon (IFN-I) response and apoptosis to inhibit viral replication
Source: Cell Death Dis. 2024 Oct 3;15(10):727. doi: 10.1038/s41419-024-07083-w (PMC11450232; doi:10.1038/s41419-024-07083-w)
Supplement: Supplementary file 12 — Supplementary Table 1 [file 41419_2024_7083_MOESM12_ESM.docx]

**Supplementary Table1. The sequences used for genotyping of PBLD knock-out mice**

| **PCR Primers** | **Name** | **Sequences (5’-3’)** | **Gene type (Size)** | | |
| --- | --- | --- | --- | --- | --- |
|  |  |  | ***Pbld^-/-^*** | ***Pbld^+/-^*** | ***Pbld^+/+^*** |
| PCR Primers1 | F1: | GAGCTAGTCTCCGAGCTTTCTATC | 532bp | 532bp | No |
|  | R1: | CAAATTCACTGGCTGCATAGCTGC |  |  |  |
| PCR Primers2 | F2: | TTTCAAGCCACGTGGATCTAGCT | No | 621bp | 621bp |
|  | R1: | CAAATTCACTGGCTGCATAGCTGC |  |  |  |

**Note:** F, Forward Primer; R, Reverse Primer; bp, base pair; No, No band.
